# microRNA-25 drives immune checkpoint therapy resistance by repressing innate and humoral immunity via Syndecan-3

**DOI:** 10.1038/s41467-026-73339-y

**Published:** 2026-05-20

**Authors:** Zhouting Zhu, Wenyan Han, Yufei Deng, Zhaoyang Jia, Gulshanbir Baidwan, Lujing Wu, Shweta Jakhmola, Tongyun Wang, Dhenugen Logeswaran, Jing Wen, Amanda Y. Sun, Bill Bray, Na Li, Lingling Wang, Hui Hui, Jiaqian Wu, Sandip Pravin Patel, Tariq M. Rana

**Affiliations:** 1 Department of Cellular and Molecular Medicine, University of California San Diego, 9500 Gilman Drive, La Jolla, California, USA; 2 Graduate School of Biomedical Sciences, Sanford Burnham Prebys Medical Discovery Institute, 10901 North Torrey Pines Road, La Jolla, California, USA; 3 Moores Cancer Center, 3855 Health Sciences Drive, University of California San Diego, La Jolla, California, USA

**Keywords:** Tumour immunology, Non-coding RNAs

## Abstract

Immune checkpoint therapy (ICT) can induce durable tumor control but is limited by primary and acquired resistance. The mechanisms underlying immune-resistant tumor microenvironments (TMEs) remain incompletely understood. Here we show that deletion of microRNA-25 (miR-25) sensitizes tumors to ICT across multiple syngeneic mouse models. Single-cell transcriptomics reveals that miR-25 deficiency activates innate and humoral immunity by increasing major histocompatibility complex class II (MHC II) expression in tumor-associated macrophages (TAMs) and enhancing classical complement signaling in cancer-associated fibroblasts (CAFs). Complement activation shifts CAFs toward an inflammatory (iCAF) state, reduces suppressive crosstalk with TAMs, and promotes a pro-inflammatory TME. Mechanistically, miR-25 represses Syndecan-3 (SDC3) in response to interferon-γ (IFN-γ). Editing the miR-25 binding site in *Sdc3* restores SDC3 expression and overcomes resistance. These findings identify miR-25–mediated SDC3 repression as a driver of immune resistance and suggest strategies to convert immune-cold tumors into ICT-responsive hot tumors, offering avenues to enhance ICT.

## Abstract (web summary)

Immune checkpoint therapy (ICT) offers durable cancer control but is limited by resistance. This study shows that deleting miR-25 enhances ICT by activating innate and humoral immunity, remodeling CAF and macrophage interactions.

## Introduction

Immune checkpoint therapy (ICT) targeting cytotoxic T-lymphocyte antigen-4 (CTLA-4) and programmed death-1 (PD-1)/programmed death-ligand-1 (PD-L1) has transformed cancer treatment^1^. PD-1 inhibitors (nivolumab and pembrolizumab) and PD-L1 inhibitors (atezolizumab, avelumab, and durvalumab) are utilized as first-line or subsequent therapies for various cancers. However, their clinical benefits remain limited, with response rates to anti-PD-1/PD-L1 therapies averaging 20–30%, depending on the cancer type^2^. A substantial proportion of patients exhibits primary resistance, while some initial responders eventually develop acquired resistance^2^. Strong immunoediting and reduced tumor antigenicity diminish the efficacy of anti-PD-1 immunotherapy, even in young melanoma patients with robust immune systems^3^. Biomarkers distinguishing responders from non-responders have been identified, with high tumor mutation burden (TMB), the presence of immunogenic neoantigens, and mismatch repair (MMR) deficiency being reportedly associated with increased objective response rates^4^. A pro-inflammatory, anti-tumor immune response depends on the recognition of tumor cells by the host immune system; however, the molecular and cellular mechanisms underlying tumor immunogenicity remain insufficiently explored.

MicroRNAs (miRNAs or miRs) are small non-coding RNAs (~19–22 nucleotides) that regulate gene expression post-transcriptionally. They are processed from primary microRNAs (pri-miRNAs) into precursor microRNAs (pre-miRNAs) by Drosha and subsequently into mature microRNAs by Dicer^5,6^. Mature miRNAs are loaded onto the Argonaute-2 (AGO2) to form the miRNA-induced silencing complex (miRISC), which binds to untranslated regions (UTRs) of target mRNAs to promote mRNA degradation, a primary mechanism for gene silencing by miRNAs^7^. The miR-17 ~ 92 cluster (oncomiR-1) is a well-characterized oncogenic miRNA cluster, and its paralog miR-106b ~ 25 encodes miR-25, which is upregulated in melanoma cell lines and patient samples^8^. Beyond melanoma, miR-25 participates in diverse tumorigenic processes, including hypoxia-driven immunosuppression through repression of cGAS, a key innate DNA sensor^9^. However, whether miR-25 contributes to immune evasion and resistance to cancer immunotherapy remains unclear.

Here we identify miR-25 as a key regulator of immune resistance to ICT. miRNA profiling during anti-PD-1 therapy indicates miR-25 as selectively downregulated in tumors responsive to treatment, a finding functionally validated by CRISPR-Cas9–mediated *Mir25* knockout across three syngeneic tumor models. Notably, miR-25 deletion did not affect tumor growth in the absence of immunotherapy. Single-cell RNA sequencing (scRNA-seq) revealed that miR-25 deficiency activates innate and humoral immunity by increasing major histocompatibility complex class II (MHC II) expression in antigen-presenting tumor-associated macrophages (TAMs) and enhancing classical complement signaling in cancer-associated fibroblasts (CAFs). This is accompanied by a shift toward inflammatory CAFs (iCAFs), reduced immunosuppressive crosstalk with TAMs, and a pro-inflammatory tumor microenvironment (TME). These effects depend on interferon-γ (IFN-γ) and miRISC activity. Mechanistically, miR-25 represses the membrane proteoglycan Syndecan-3 (SDC3) under IFN-γ stimulation, thereby limiting innate immunosurveillance. Depletion of SDC3 abolished the therapeutic benefit of miR-25 deletion. Editing the miR-25 binding site in the *Sdc3* 3′ UTR restores SDC3 expression and phenocopies the therapeutic effect of miR-25 deletion, supporting SDC3 as a key functional target. These findings reveal a role of the tumor miR-25–SDC3 axis in enabling evasion from innate and humoral immunosurveillance and suggest therapeutic strategies to enhance ICT efficacy.

## Results

### miR-25 deficiency enhances tumor responses to immunotherapy

**Fig. 1:**
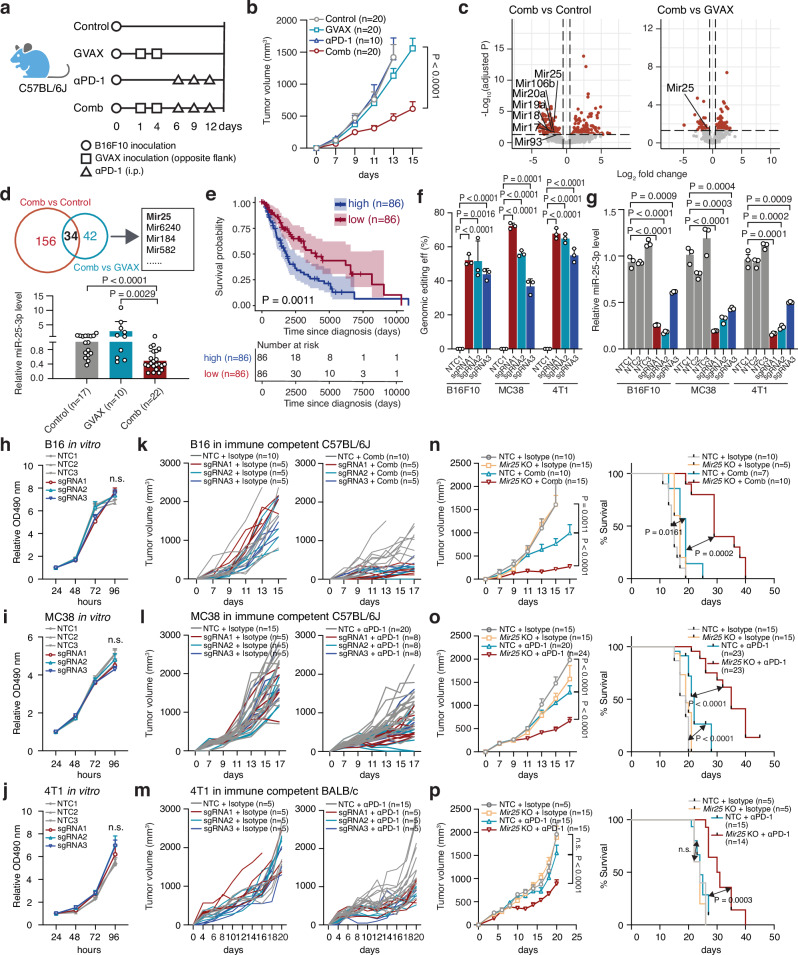
miR-25 deficiency enhances tumor responses to immune checkpoint therapy (ICT). **a** Schematic representation of the experimental design (created with BioRender.com). **b** Tumor growth curves in the B16F10 model (mean ± SEM). *n* indicates independent mice; exact sample sizes are shown in the figure. GVAX, granulocyte–macrophage colony-stimulating factor–secreting tumor cell vaccine; αPD-1, anti–programmed cell death protein 1; Comb, GVAX + αPD-1. **c** Volcano plot showing small RNA-seq analysis of B16F10 tumors. Sample size *n* = 3 per group; *n* indicates independent mice. **d** Top, Venn diagram showing overlap of downregulated miRNAs. Bottom, RT–qPCR validation of miR-25 expression (mean ± SD). *n* indicates independent mice; exact sample sizes are shown in the figure. Each point represents one tumor. **e** Kaplan–Meier survival analysis of TCGA-SKCM-TM patients stratified by miR-25 expression (high, *n* = 86 patients; low, *n* = 86 patients). Shaded areas represent 95% confidence intervals (CI). **f** TIDE analysis of predicted CRISPR editing efficiency (mean ± SD; *n* = 3 independent experiments). **g** RT–qPCR quantification of miR-25-3p expression comparing NTC (non-targeting control) and KO (*Mir25* knockout) cells (mean ± SD; *n* = 3 independent experiments). **h**–**j** In vitro proliferation assays in indicated tumor cell lines (mean ± SD; *n* = 6 independent experiments). **k**–**m** In vivo tumor growth of NTC and *Mir25* KO tumors under isotype or ICT treatment. Each line represents one mouse. *n* indicates independent mice; exact sample sizes are shown in the figure. **n**–**p** Tumor growth curves (left) and survival curves (right). Tumor growth (mean ± SEM). *n *indicates independent mice; exact sample sizes are shown in the figure. Upper *P* values compare NTC + treatment (Comb for B16; αPD-1 for MC38 and 4T1) and NTC + isotype. Lower *P* values compare *Mir25* KO + treatment and NTC + treatment. Statistical significance in (**b**, **h**–**j**, **n**–**p** tumor growth) was assessed using two-way ANOVA with Tukey’s multiple comparisons test. Statistical significance in (**d**, **f**, **g**) was assessed using two-sided Student’s t test. Survival in (**e**, **n**–**p** survival) was analyzed using the log-rank (Mantel–Cox) test. Source data are provided in the Source Data file.

To investigate the role of miRNAs in modulating responses to anti-PD-1 therapy, we utilized the B16F10 (B16) murine melanoma model, which generates poorly immunogenic tumors^10,11^. B16 cells were implanted subcutaneously into immunocompetent C57BL/6J mice, followed by granulocyte-macrophage colony-stimulating factor (GM-CSF)–secreting tumor cell vaccine (GVAX)^12–15^ vaccination and anti-PD-1 antibody treatment as indicated (Fig. 1a). Four treatment groups were analyzed: no treatment (Control), GVAX alone (GVAX), anti-PD-1 alone (αPD-1), and combination therapy (GVAX + αPD-1; Comb). Combination therapy significantly reduced tumor growth, whereas either monotherapy showed limited efficacy (Fig. 1b; Supplementary Fig. S1a).

Small RNA sequencing of tumors collected during therapy revealed downregulation of multiple members of the miR-17 ~ 92 cluster and its paralogs in response to treatment (Fig. 1c; Supplementary Dataset 1). Notably, miR-25 was significantly decreased in both Comb vs Control and Comb vs GVAX comparisons and exhibited the highest expression levels among candidates. This finding was validated by quantitative real-time PCR (qRT–PCR) (Fig. 1d; Supplementary Dataset 1). We then analyzed small RNA datasets from The Cancer Genome Atlas (TCGA). Among 352 skin cutaneous metastatic melanoma (SKCM, metastatic tumors, TM) patient samples, we categorized them into miR-25-low and miR-25-high expression groups (Supplementary Fig. S1b; Supplementary Dataset 2). Patients in the miR-25-low group demonstrated relatively prolonged survival, supporting the potential role of miR-25 in modulating immune responses and prognosis (Fig. 1e).

To determine the functional role of miR-25, we generated *Mir25* knockout (KO) tumor cell lines using CRISPR-Cas9. The *Mir25* locus encodes a stem-loop primary transcript processed by Drosha and Dicer to produce mature miR-25-3p, which is incorporated into AGO2-containing miRNA-induced silencing complexes (miRISCs). CRISPR-Cas9 editing introduces insertions and deletions (indels) within the *Mir25* stem-loop, thereby disrupting its processing and reducing mature miR-25 levels. To ensure efficient disruption, we designed three sgRNAs targeting distinct regions of the *Mir25* precursor, including the Dicer processing site (Supplementary Fig. S1c; Supplementary Dataset 3). Editing efficiency was confirmed by Sanger sequencing and TIDE^16^ analysis, showing up to 80% indel formation (Fig. 1f). Consistent with the editing efficiency, mature miR-25-3p levels were significantly reduced, as measured by qRT–PCR (Fig. 1g).

*Mir25* KO in the three syngeneic mouse cancer cell lines (B16, MC38, and 4T1) did not affect tumor cell proliferation in vitro compared to non-targeted control (NTC) (Fig. 1h–j). To further evaluate cell-intrinsic effects of miR-25 loss, we isolated single-cell clones with homozygous miR-25 deletion (Supplementary Fig. S1d; Supplementary Dataset 4). Homozygous deletion further reduced mature miR-25 levels relative to the previous bulk KO population (Supplementary Fig. S1e). Despite the inherent heterogeneity of cancer cells, we observed no significant defects in cell viability or morphology, which were further confirmed in three representative homozygous KO clones by assays of proliferation (Supplementary Fig. S1f, S1j, S1n), apoptosis (all below background rate 10%)^17^ (Supplementary Fig. S1g–h, S1k–l, S1o–p), and migration (Supplementary Fig. S1i, S1m, S1q). Similarly, miR-25-deficient tumors treated with isotype control antibodies exhibited tumor progression comparable to NTC tumors in vivo (Fig. 1k–m). However, under anti-PD-1 immunotherapy, *Mir25* KO tumor growth was significantly reduced relative to NTC, independent of the sgRNA or single-cell clone used (Fig. 1k–m; and Supplementary Fig. S1r; Supplementary Dataset 4). This effect was observed with GVAX plus αPD-1 therapy in B16 tumors, and with αPD-1 monotherapy in MC38 and 4T1 tumors. Because all sgRNAs yielded comparable reductions in mature miR-25-3p levels (Fig. 1g), the results were combined and referred to as *Mir25* KO. As a result, miR-25-deficient tumors displayed reduced tumor burden and improved survival compared with controls (Fig. 1n–p). Re-expression of *Mir25* in knockout cells restored miR-25 levels and abolished the enhanced therapeutic response, excluding potential off-target effects (Supplementary Fig. S1s–v; and Supplementary Dataset 4). Collectively, these data indicate that loss of miR-25 does not affect tumor cell–intrinsic properties but sensitizes tumors to immune checkpoint therapy.

### Single-cell RNA-seq reveals activation of innate and humoral immunity in miR-25-deficient tumors

**Fig. 2:**
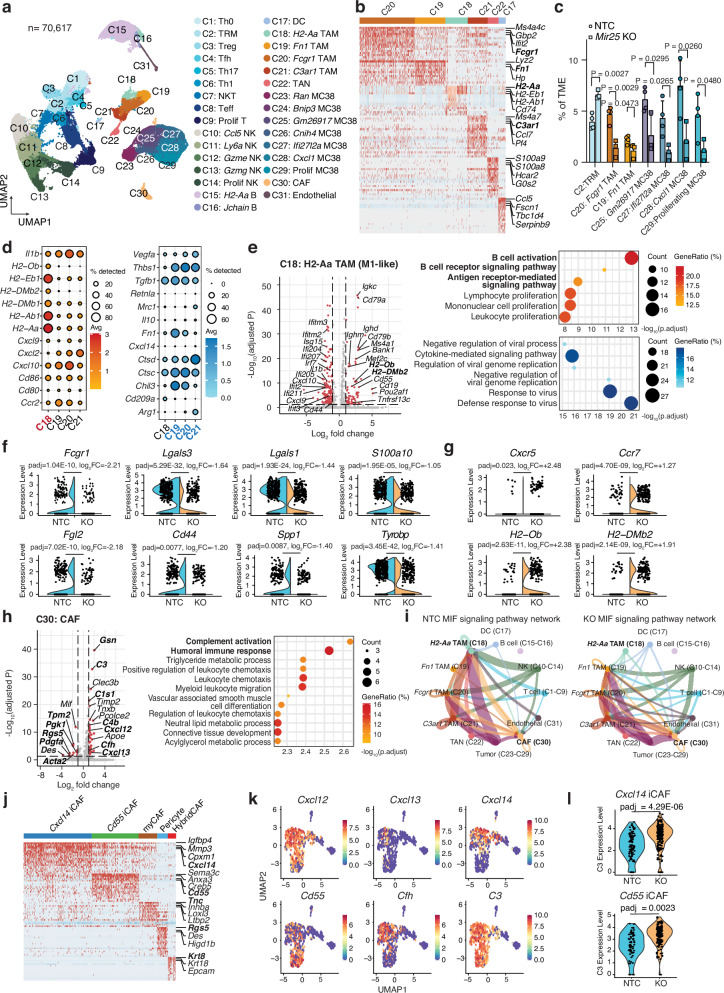
Single-cell RNA sequencing (scRNA-seq) reveals activation of innate and humoral immunity in the tumor microenvironment (TME). **a** UMAP visualization of MC38 tumors (NTC + αPD-1 and *Mir25* KO + αPD-1, day 17) showing annotated cellular subclusters. A total of 70,617 cells from independent mouse tumors (NTC, *n* = 4; *Mir25* KO, *n* = 3) are shown. Th0, T helper 0; TRM, tissue-resident memory T cell; Treg, regulatory T cell; Tfh, T follicular helper cell; Th17, T helper 17 cell; Th1, T helper 1 cell; NKT, natural killer T cell; Teff, effector T cell; Prolif T, proliferating T cell; DC, dendritic cell; TAM, tumor-associated macrophage; TAN, tumor-associated neutrophil; CAF, cancer-associated fibroblast. NTC, non-targeting control; KO, *Mir25* knockout. **b** Heatmap showing top marker genes defining myeloid subclusters. **c** Cellular composition changes between *Mir25* KO and NTC tumors (mean ± SD; NTC, *n* = 4 mice; *Mir25* KO, *n* = 3 mice). **d** Bubble plot showing expression of M1 and M2 macrophage markers across tumor-associated macrophage (TAM) subsets. **e** Analysis of C18 TAMs. Left, volcano plot of differentially expressed genes. Right, Gene Ontology (GO) enrichment analysis. **f**, **g** Expression of representative genes in C18 TAMs. Each point represents one cell from independent mice (NTC, *n* = 4 mice; *Mir2*5 KO, *n* = 3 mice). **h** Analysis of C30 cancer-associated fibroblasts (CAFs). **i** Circle diagram showing macrophage migration inhibitory factor (MIF)–mediated cell–cell communication. **j** Heatmap showing top marker genes defining CAF subclusters. **k** UMAP visualization of inflammatory cancer-associated fibroblast (iCAF) signature genes. Gene expression shown as log_10_(TPM + 1). Data derived from single-cell RNA-seq (*n* = 7 mice in total). **l** Differential expression of complement 3 (C3) in iCAF clusters. Each point represents one cell from independent mice (NTC, n = 4 mice; *Mir25* KO, *n* = 3 mice). Differential expression in (**e**–**h**, **l**) was performed using DESeq2 with two-sided Wald test and Benjamini–Hochberg correction. GO analysis in (**e**, **h**) was performed using clusterProfiler with Benjamini–Hochberg correction. Statistical significance in (**c**) was assessed using two-sided Student’s t test. Source data are provided in the Source Data file.

To assess the impact of miR-25 loss on the tumor microenvironment (TME), we performed single-cell RNA sequencing of MC38 tumors treated with anti-PD-1. After quality control, we obtained 70,617 transcriptomes from four NTC tumors and three *Mir25* KO tumors. Cell type annotation identified major immune and stromal populations, including T cells, B cells, natural killer (NK) cells, dendritic cells (DCs), tumor-associated macrophages (TAMs), tumor-associated neutrophils (TANs), cancer-associated fibroblasts (CAFs), tumor cells, and endothelial cells (Fig. 2a; Supplementary Fig. S2a–c; and Supplementary Dataset 5). T cells were further subdivided into nine clusters based on marker gene expression (Supplementary Fig. S2d), and myeloid populations included multiple TAM subsets as well as DCs and TANs (Fig. 2b; Supplementary Dataset 5). Notably, miR-25 deficiency was associated with an increase in tissue-resident memory T cells and a reduction in specific TAM and tumor cell populations (Fig. 2c). Despite the expansion of tissue-resident memory T cells, these cells exhibited only limited transcriptional changes, indicating that T cell alterations alone may not explain the observed tumor control (Supplementary Fig. S2e).

We therefore focused on TAMs, which showed marked compositional changes. Based on established scRNA-seq markers of macrophage polarization^18,19^, TAM subsets were classified into M1-like and M2-like populations. Cluster 18 (C18) exhibited high expression of major histocompatibility complex class II (MHC II) genes, including *H2-Eb1*, *H2-DMb2*, *H2-DMb1*, *H2-Ab1*, and *H2-Aa*, and was thus defined as antigen-presenting M1-like TAMs. In contrast, clusters C19 (*Fn1* TAM), C20 (*Fcgr1* TAM), and C21 (*C3ar1* TAM) expressed M2-associated markers such as *Thbs1*, *Tgfb1*, *Fn1*, and *Chil3*, consistent with M2-like phenotypes (Fig. 2d). Notably, the TAM populations that were reduced in miR-25-deficient tumors (C19 and C20) corresponded to M2-like subsets. However, these M2-like TAMs displayed only limited transcriptional changes (Supplementary Fig. S2f, g; and Supplementary Dataset 6), suggesting that their reduced abundance, rather than functional reprogramming, contributes to the observed phenotype. In contrast, M1-like TAMs (C18) exhibited extensive transcriptional remodeling. Genes associated with antigen presentation and B cell activation were upregulated, including MHC II components such as *H2-Ob* and *H2-DMb2*. Type I interferon–related genes (*Ifitm2*, *Ifitm3*, *Isg15*) were downregulated (Fig. 2e). In addition, multiple immunosuppressive or M2-associated genes, including *Fcgr1*, *Lgals3*, *Lgals1*, *S100a10*, *Fgl2*, *Cd44*, *Spp1*, and *Tyrobp*, were also reduced (Fig. 2f; Supplementary Dataset 6). Conversely, immune activation–associated genes, including *Cxcr5* and *Ccr7*, were upregulated (Fig. 2g; and Supplementary Dataset 6). CAFs exhibited increased expression of classical complement components, including *C1s1*, *C3*, and *C4b*. This was accompanied by a shift toward inflammatory CAFs (iCAFs) and reduced expression of genes associated with myofibroblastic CAFs (myCAFs) (*Tpm2*, *Pgk1*, *Rgs5*, *Pdgfa*, *Des*, and *Acta2*), indicating activation of humoral immune pathways in the miR-25-deficient TME (Fig. 2h; Supplementary Dataset 6 and 7).

To investigate tumor–immune communication in the *Mir25* KO TME, we analyzed cell–cell interaction networks using CellChat (http://www.cellchat.org)^20^. This analysis revealed enhanced complement signaling between CAFs and TAMs in *Mir25* KO tumors, with CAFs acting as a major source of complement signals directed toward M2-like macrophages (C19–C21) (Supplementary Fig. S2h; Supplementary Dataset 8). In parallel, MIF–CD74/CD44 signaling between CAFs and TAMs was reduced (Fig. 2i; Supplementary Dataset 8). Macrophage Migration Inhibitory Factor (MIF) binds to CD74, a cell-surface form of the MHC II–associated invariant chain, and forms a receptor complex with CD44 to promote macrophage recruitment and immunosuppressive signaling^21^. Consistently, *Mir25* KO tumors showed reduced expression of *Mif* in CAFs and *Cd44* in TAMs, supporting attenuation of this pathway (Fig. 2e, f, h; Supplementary Dataset 6).

To further examine how these CAF-derived signals shape tumor–immune interactions, we next sought to resolve CAF heterogeneity in the *Mir25* KO TME. CAFs were subset into five clusters based on marker gene expression. Two clusters corresponded to iCAFs, marked by *Cxcl14* and *Cd55*, whereas myCAFs and pericyte-like populations were defined by *Tnc* and *Rgs5* expression, respectively (Fig. 2j; Supplementary Fig. S2i; Supplementary Dataset 5). Notably, complement-related genes were preferentially enriched in iCAF populations characterized by *Cxcl12*, *Cxcl13*, *Cxcl14*, and *Ccl19* expression (Fig. 2k; Supplementary Fig. S2j). In particular, the central complement component C3 was selectively upregulated in the two iCAF clusters (Fig. 2l; Supplementary Dataset 6).

In summary, these findings suggest that miR-25 loss under anti-PD-1 therapy reprograms the TME by enhancing antigen-presenting M1-like macrophage activity and promoting complement-driven, iCAF-associated humoral responses. These coordinated changes are accompanied by reduced macrophage abundance and diminished immunosuppressive crosstalk, thereby establishing a more pro-inflammatory, immune-permissive TME.

### Complement activation induced by miR-25 loss is macrophage-dependent

**Fig. 3:**
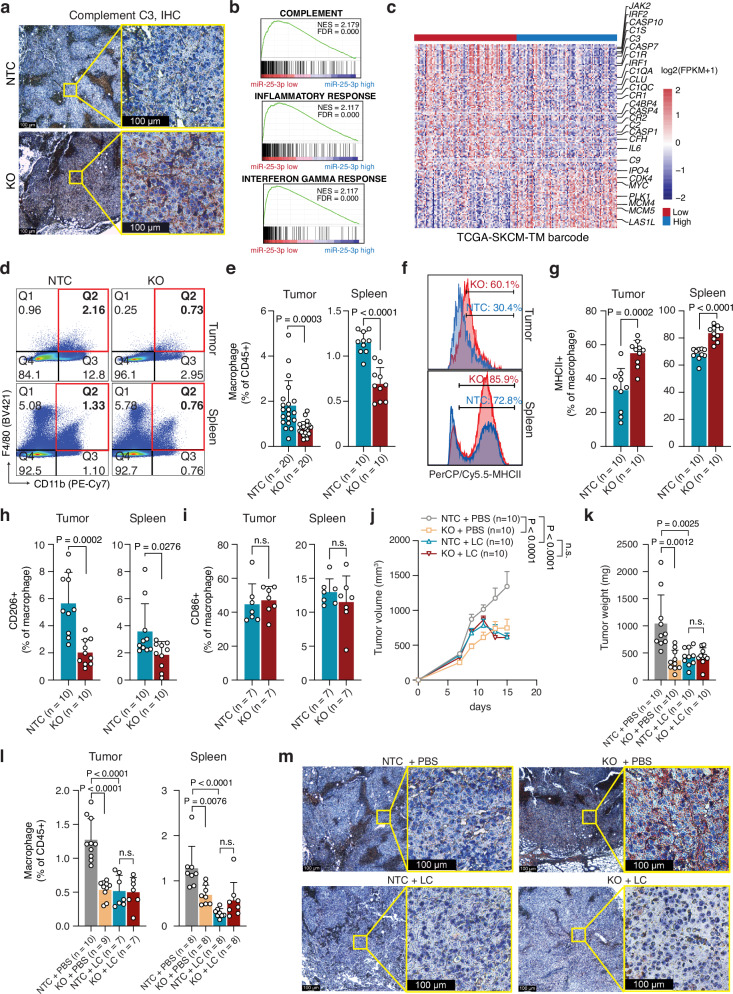
Complement activation induced by miR-25 deficiency is dependent on macrophages. **a** Representative immunohistochemistry (IHC) staining for complement 3 (C3) in B16F10 tumors (day 15). Scale bars, 100 μm. Experiments were repeated three times (*n* = 3 mice per group). NTC, non-targeting control; KO, *Mir25* knockout. **b** Gene set enrichment analysis (GSEA) comparing low (*n* = 90 patient tumors) versus high (*n* = 88 patient tumors) miR-25 expression in TCGA-SKCM-TM samples. NES, normalized enrichment score; FDR, false discovery rate. **c** Heatmap showing gene expression from enriched and downregulated pathways in TCGA-SKCM-TM samples (miR-25 high, *n* = 88 patient tumors; low, *n* = 90 patient tumors). Gene expression shown as log₂(FPKM + 1). **d** Representative flow cytometry gating of tumor and splenic macrophages (F4/80⁺CD11b⁺). **e** Flow cytometry quantification of tumor and splenic macrophages (mean ± SD). *n* indicates independent mice; exact sample sizes are shown in the figure. Each point represents one mouse. **f** Representative histogram of major histocompatibility complex class II (MHC II) expression in macrophages. **g**–**i** Flow cytometry quantification of MHC II (**g**), CD206 (**h**), and CD86 (**i**) in macrophages (mean ± SD). *n* indicates independent mice; exact sample sizes are shown in the figure. Each point represents one mouse. **j** Tumor growth curves of B16F10 tumors under macrophage depletion with GVAX + αPD-1 treatment (mean ± SEM). *n* indicates independent mice; exact sample sizes are shown in the figure. PBS phosphate-buffered saline; LC liposome clodronate. **k** Tumor weights from (**j**) (mean ± SD). *n* indicates independent mice; exact sample sizes are shown in the figure. Each point represents one mouse. **l** Flow cytometry quantification of tumor and splenic macrophages from (**j**) (mean ± SD). *n* indicates independent mice; exact sample sizes are shown in the figure. Each point represents one mouse. **m** Representative C3 IHC staining from (**j**). Scale bars, 100 μm. Experiments were repeated three times (*n* = 3 mice per group). Statistical significance in (**e**, **g**–**i**, **k**, **l**) was assessed using two-sided Student’s t test. Statistical significance in (**j**) was assessed using two-way ANOVA with Tukey’s multiple comparisons test. Source data are provided in the Source Data file.

To validate the innate and humoral immune activation observed in Fig. 2, we examined B16 *Mir25* KO tumors. Immunohistochemical staining for C3 revealed limited and localized deposition in control tumors, whereas *Mir25* KO tumors exhibited widespread C3 deposition throughout the tumor tissue, consistent with enhanced complement activation (Fig. 3a).

Transcriptomic analyses further supported immune activation upon miR-25 loss. Bulk RNA sequencing of B16 tumors and analysis of TCGA-SKCM datasets both showed enrichment of immune-related pathways in miR-25–low conditions (Supplementary Fig. S3a, b; Supplementary Dataset 9). Gene Set Enrichment Analysis (GSEA) of TCGA-SKCM samples identified complement activation, inflammatory response, and interferon-γ (IFN-γ) signaling as the top enriched pathways. In contrast, downregulated pathways included *MYC* target genes involved in cell proliferation, including *CDK4*, *MCM4* and *MCM5* (Fig. 3b, c; Supplementary Dataset 10). These findings suggest a potential negative correlation between miR-25 expression and immune infiltration and activation in melanoma.

We next assessed macrophage populations by flow cytometry (Supplementary Fig. S3c). *Mir25* KO tumors exhibited a reduced proportion of macrophages (Fig. 3d, e), accompanied by increased MHC II expression, indicating enhanced antigen-presenting capacity (Fig. 3f, g). Consistently, the M2-associated marker CD206 was decreased, suggesting a shift away from immunosuppressive macrophage states (Fig. 3h; Supplementary Fig. S3d). In contrast, the classical M1 marker CD86 expression remained unchanged (Fig. 3i; Supplementary Fig. S3d). Similar changes were observed in splenic macrophages.

To further investigate the functional role of macrophages, we utilized liposome clodronate (LC) to deplete macrophages in B16 tumor-bearing mice. LC was administered twice, starting on day 8, in combination with the original GVAX and anti-PD-1 immunotherapy regimen. Macrophage depletion reduced tumor growth in both control and *Mir25* KO groups and abolished the therapeutic advantage of *Mir25* KO tumors, resulting in comparable tumor burdens between groups (Fig. 3j, k; and Supplementary Fig. S3e). Efficient macrophage depletion was confirmed by flow cytometry, which showed reduced TAMs and splenic macrophages (Fig. 3l). Importantly, depletion of macrophages also markedly reduced C3 deposition within the tumor microenvironment (Fig. 3m). Together, these findings validate a miR-25 deficiency mechanism of innate and humoral immune responses in B16 melanoma and confirm that complement activation in the tumor microenvironment is dependent on macrophages.

### miR-25 induced effect is dependent on tumor IFN-γ response and miRISC complex

**Fig. 4:**
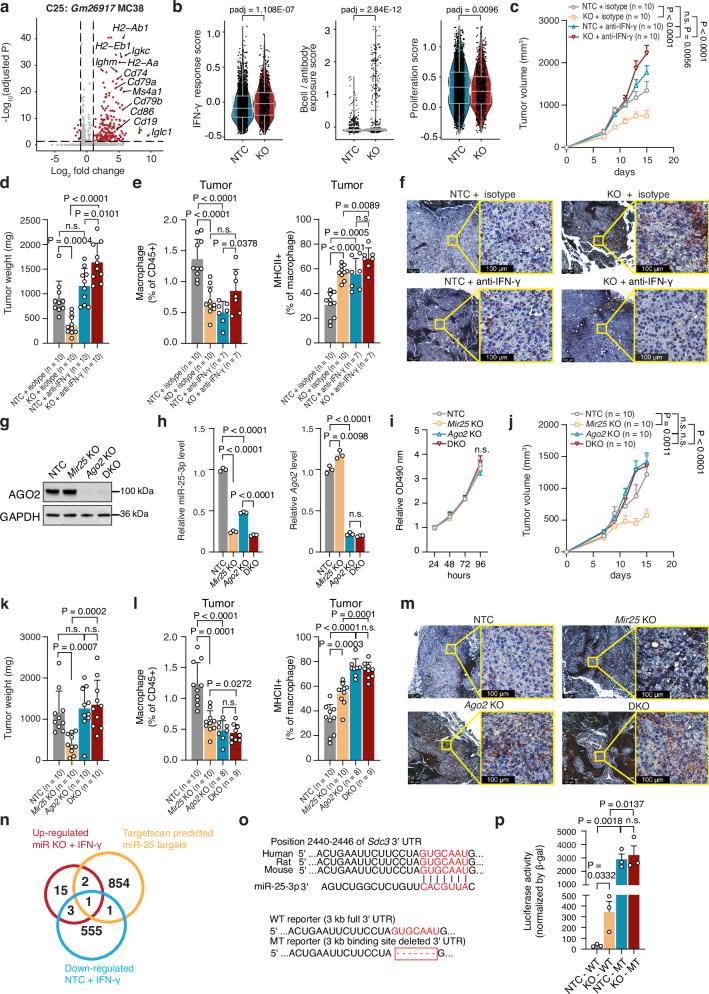
IFN-γ signaling and miRNA-induced silencing complex (miRISC) are required for miR-25–mediated immune regulation. **a** Volcano plot showing differentially expressed genes from cluster 25 (Fig. 2), comparing *Mir25* knockout (KO) and non-targeting control (NTC) tumors. **b** Violin plots showing IFN-γ response (*Ifng*, *Stat1*, *Irf1*, *Irf8*, *Cxcl9*, *Cxcl10*, *Gbp2*, *H2-Ab1*, *Cd274*, *Nos2*, *Tnf*, *Tap1*, *Ifngr1*), B-cell/antibody exposure (*Igkc*, *Ighm*, *Cd79a*, *Mzb1*, *Ms4a1*), and proliferation (*Mki67*, *Pcna*, *Ccn1*, *Cdk1*, *Top2a*, *Mcm3*, *Mcm7*, *Birc5*). Box plots show median (center), interquartile range (box), and whiskers (1.5 × IQR). Each point represents one cell from independent mice (NTC, *n* = 4 mice; *Mir25* KO, *n* = 3 mice). **c** Tumor growth curves under IFN-γ blockade with GVAX + αPD-1 treatment (mean ± SEM). *n* indicates independent mice; exact sample sizes are shown in the figure. **d** Tumor weights (mean ± SD). *n* indicates independent mice; exact sample sizes are shown in the figure. Each point represents one mouse. **e** Flow cytometry quantification of tumor-associated macrophages (TAMs, F4/80⁺CD11b⁺) (mean ± SD). *n* indicates independent mice; exact sample sizes are shown in the figure. Each point represents one mouse. **f** Representative C3 IHC staining from (**c**). Scale bars, 100 μm. Experiments were repeated three times (*n* = 3 mice per group). **g** Immunoblot analysis of AGO2 and GAPDH in B16F10 cells. Blots are representative of three independent experiments. DKO, *Mir25*/*Ago2* double knockout. **h** RT–qPCR quantification of miR-25-3p and *Ago2* (mean ± SD; *n* = 3 independent experiments). **i** In vitro proliferation assay (mean ± SD; *n* = 6 independent experiments). **j** Tumor growth curves with GVAX + αPD-1 treatment (mean ± SEM). *n* indicates independent mice; exact sample sizes are shown in the figure. **k** Tumor weights (mean ± SD). *n* indicates independent mice; exact sample sizes are shown in the figure. Each point represents one mouse. **l** Flow cytometry quantification of TAMs (mean ± SD). *n* indicates independent mice; exact sample sizes are shown in the figure. Each point represents one mouse. **m** Representative C3 IHC staining from (**j**). Scale bars, 100 μm. Experiments were repeated three times (*n* = 3 mice per group). **n** Venn diagram showing overlap among the indicated gene sets. **o** Schematic of *Sdc3* 3′ UTR luciferase reporter constructs: WT, wild-type 3′ UTR containing the miR-25 binding site; MT, binding site deleted. **p** Luciferase activity assay (mean ± SD; *n* = 3 independent experiments). Differential expression in (**a**) was analyzed using DESeq2 with a two-sided Wald test and Benjamini–Hochberg correction. Statistical significance in (**b**) was assessed by a two-sided Wilcoxon rank-sum test on sample-level aggregated data; (**c**, **i**, **j**) was assessed using two-way ANOVA with Tukey’s multiple comparisons test; (**d**, **e**, **h**, **k**, **l**, **p**) was assessed using two-sided Student’s t test. Source data are provided in the Source Data file.

To investigate the tumor-intrinsic mechanisms underlying the observed innate and humoral immune responses, we analyzed tumor cell clusters identified by scRNA-seq (C23–C29; Fig. 2a). Tumor identity was confirmed by marker expression and inferCNV (https://github.com/broadinstitute/inferCNV)^22^ analysis, which distinguished these clusters from non-tumor populations (Supplementary Fig. S4a, b). Among tumor clusters, only C25 exhibited substantial differential gene expression between *Mir25* KO and control tumors (Fig. 4a; and Supplementary Dataset 6). Because anti-PD-1 immunotherapy enhances IFN-γ secretion by activated T cells^23–25^, we next assessed tumor cell responsiveness to IFN-γ signaling. This IFN-γ response score was calculated using genes including *Ifng*, *Stat1*, *Irf1*, *Irf8*, *Cxcl9*, and *Cxcl10* (Supplementary Fig. S4c). Only C25 showed an elevated IFN-γ response in *Mir25* KO tumors (Fig. 4b, left), indicating enhanced sensitivity to immune-derived IFN-γ. Consistent with this, C25 revealed increased expression of antigen-presentation genes (*H2-Ab1, H2-Eb1, H2-Aa*), together with elevated B-cell/antibody exposure scores (Fig. 4b, middle), indicating activation of humoral immunity. We also quantified proliferative capacity using canonical proliferation markers (*Mki67, Pcna, Ccn1, Cdk1, Top2a, Mcm3, Mcm7, Birc5*), which revealed reduced proliferation scores in *Mir25* KO tumors (Fig. 4b, right). Since miR-25 deletion does not intrinsically affect tumor cell proliferation (Fig. 1i), these changes likely reflect immune-mediated suppression. These results suggest that miR-25 loss enhances tumor cell responsiveness to IFN-γ, linking tumor-intrinsic signaling to immune activation.

To determine the effect of IFN-γ response on anti-PD-1 immunotherapy, we depleted IFN-γ using an antibody starting on day 8. IFN-γ depletion reversed the tumor suppression effects of miR-25 deletion in vivo. *Mir25* KO tumor-bearing mice treated with combination therapy plus anti-IFN-γ antibody exhibited larger tumor volumes and heavier tumor weights compared to controls receiving the same treatment (Fig. 4c, d; Supplementary Fig. S4e). To explain this phenomenon, we conducted flow cytometry analysis on tumors and spleens. Interestingly, IFN-γ depletion resulted in reduced macrophage proportions in control tumors, whereas in the *Mir25* KO group with IFN-γ depletion, macrophage abundance was increased in both tumors and spleens (Fig. 4e; Supplementary Fig. S4f). Despite these opposite trends in macrophage frequency, IFN-γ depletion led to an overall upregulation of MHC II expression in tumor-associated macrophages in both the control and *Mir25* KO groups (Fig. 4e). In contrast, splenic macrophages exhibited reduced MHC II expression in *Mir25* KO tumor-bearing mice compared with controls following IFN-γ depletion (Supplementary Fig. S4f). Finally, we assessed the humoral immunity and complement activation within the tumor microenvironment. Consistent with the increased tumor burden and altered macrophage composition, C3 infiltration was reduced in IFN-γ depleted *Mir25* KO tumors, with fewer C3-positive regions detected by immunohistochemistry (Fig. 4f). Together, these findings demonstrate that the anti-tumor effects induced by miR-25 loss are dependent on the tumor’s IFN-γ response.

Mature microRNAs, Argonaute-2 (AGO2), and several other proteins are assembled into the miRISC complex to regulate target mRNAs, with AGO2 serving as the key protein of this complex^5,6^. AGO2 has been reported to promote tumor immune evasion by suppressing CXCL chemokines, interferon-stimulated genes (ISGs), and HLA molecules^26^. To determine whether miR-25 regulates its target mRNAs through the canonical miRNA–Ago2 pathway, we generated B16 *Ago2* knockout (*Ago2* KO) cells as well as *Mir25*/*Ago2* double knockout (DKO) cells (Fig. 4g–i). Notably, loss of *Ago2* abolished the tumor suppression observed in *Mir25* KO tumors, with *Ago2* KO and DKO tumors exhibiting growth comparable to control tumors under combination immunotherapy (Fig. 4j, k; Supplementary Fig. S4g). These results indicate that the anti-tumor effect of miR-25 loss is fully dependent on AGO2-mediated miRISC activity. Despite the loss of tumor suppression, several immune features associated with *Mir25* KO tumors were partially retained in *Ago2* KO tumors, consistent with the partial reduction of miR-25 levels upon *Ago2* loss (Fig. 4h). Similar to *Mir25* KO tumors, *Ago2* KO and DKO tumors showed reduced macrophage abundance together with increased MHC II expression in tumor-associated macrophages (Fig. 4l), accompanied by a modest increase in complement deposition (Fig. 4m). In contrast, splenic macrophages displayed a distinct pattern, with *Ago2* KO increasing macrophage abundance without affecting MHC II expression (Supplementary Fig. S4h), suggesting that systemic immune effects of AGO2 loss extend beyond miR-25 and likely involve additional miRNA pathways^26^.

Because our results indicate that the tumor phenotype associated with miR-25 deletion depends on both IFN-γ signaling and the canonical miRNA silencing machinery, we next sought to identify miR-25 target genes linking these pathways. To this end, we performed RNA-seq analysis of B16 cells treated with IFN-γ and compared gene expression between *Mir25* KO and control cells. This analysis identified 15 genes significantly upregulated in *Mir25* KO cells under IFN-γ stimulation (Fig. 4n; Supplementary Fig. S4i; Supplementary Dataset 11). Intersection with TargetScan (https://www.targetscan.org/)^27^ predictions narrowed these candidates to two potential direct targets, *Notch1* and *Sdc3*. We therefore examined whether miR-25 regulates these genes in response to IFN-γ. In control cells, *Sdc3* was reduced following IFN-γ treatment, whereas this repression was abolished in *Mir25* KO cells (Fig. 4n; Supplementary Fig. S4i, j), consistent with miR-25–dependent silencing. To test whether *Sdc3* is a direct miR-25 target, we performed luciferase reporter assays using the full-length *Sdc3* 3′ untranslated region (3′ UTR). IFN-γ treatment significantly reduced luciferase activity in control cells carrying the wild-type reporter, whereas this repression was lost in miR-25-deficient cells. Deletion of the predicted miR-25 binding site abolished the IFN-γ–dependent repression (Fig. 4o, p). Notably, in *Mir25* KO cells, luciferase activity remained lower with the wild-type reporter than with the binding site–deleted construct, suggesting that additional microRNAs may also contribute to *Sdc3* regulation (Fig. 4p). These results identify *Sdc3* as a direct miR-25 target and demonstrate that miR-25 restricts IFN-γ–responsive tumor immunity through AGO2-mediated post-transcriptional regulation of *Sdc3*.

### miR-25 regulates the tumor membrane proteoglycan Syndecan-3 to promote IFN-γ–dependent immune evasion

**Fig. 5:**
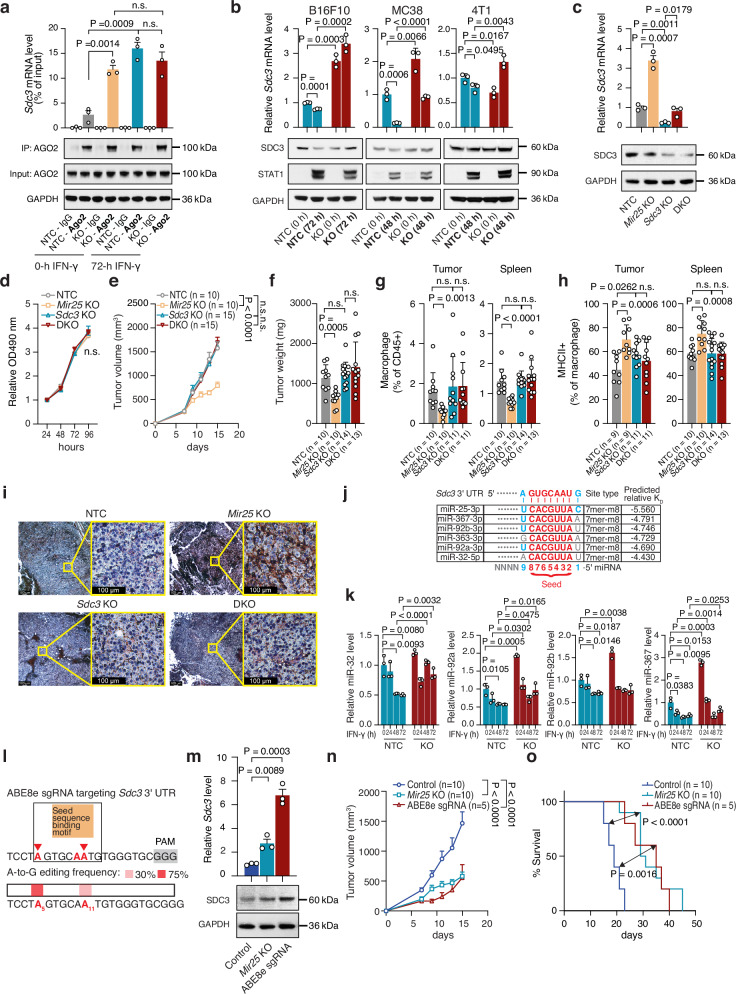
miR-25 regulates SDC3 to promote IFN-γ–dependent immune evasion. **a** RNA immunoprecipitation (RIP) assay showing enrichment of *Sdc3* transcripts in AGO2 complexes comparing *Mir25* knockout (KO) and non-targeting control (NTC) B16 cells (mean ± SD; *n* = 3 independent experiments). **b** RT–qPCR and immunoblot analysis of SDC3 and STAT1 expression following IFN-γ treatment. Representative blots from three experiments. RT–qPCR data are mean ± SD (*n* = 3 independent experiments). **c** RT–qPCR and immunoblot analysis of SDC3 expression in cells expressing indicated sgRNAs. Representative blots from three independent experiments. RT–qPCR data are mean ± SD (*n* = 3 independent experiments). DKO, *Mir25*/*Sdc3* double knockout. **d** In vitro proliferation assay (mean ± SD; *n* = 6 independent experiments). **e** Tumor growth curves of B16F10 tumors treated with GVAX + αPD-1 (mean ± SEM). *n* indicates independent mice; exact sample sizes are shown in the figure. **f** Tumor weights (mean ± SD). *n* indicates independent mice; exact sample sizes are shown in the figure. Each point represents one mouse. **g** Flow cytometry quantification of macrophages (F4/80⁺CD11b⁺) (mean ± SD). *n* indicates independent mice; exact sample sizes are shown in the figure. Each point represents one mouse. **h** Flow cytometry quantification of MHC II expression in macrophages (mean ± SD). *n* indicates independent mice; exact sample sizes are shown in the figure. Each point represents one mouse. **i** Representative C3 IHC staining. Scale bars, 100 μm. Experiments were repeated three times (*n* = 3 mice per group). **j** Predicted miR-25 family binding sites in the *Sdc3* 3′ UTR. Seed sequences shown in red; additional pairing shown in blue. Predicted relative dissociation constants (K_d) obtained from TargetScan. **k** RT–qPCR quantification of miR-25 family expression (mean ± SD; *n* = 3 independent experiments). **l** Schematic of adenine base editor 8e (ABE8e)–mediated editing targeting the *Sdc3* 3′ UTR. **m** RT–qPCR and immunoblot analysis of SDC3 expression after 72 h IFN-γ treatment. Representative blots from three experiments. Data are mean ± SD (*n* = 3 independent experiments). **n** Tumor growth curves of B16F10 tumors treated with GVAX + αPD-1 (mean ± SEM). *n* indicates independent mice; exact sample sizes are shown in the figure. **o** Kaplan–Meier survival analysis of mice bearing B16F10 tumors. *n* indicates independent mice; exact sample sizes are shown in the figure. Statistical significance in (**a**–**c**, **f**–**h**, **k**, **m**) was assessed using two-sided Student’s t test. Statistical significance in (**d**, **e**, **n**) was assessed using two-way ANOVA with Tukey’s multiple comparisons test. Survival in (**o**) was analyzed using log-rank (Mantel–Cox) test. Source data are provided in the Source Data file.

To determine whether miR-25 represses SDC3 through the miRISC complex, we performed AGO2 RNA immunoprecipitation (RIP) followed by qPCR together with transcript and protein analyses. At baseline, *Mir25* KO cells showed increased *Sdc3* mRNA and enhanced AGO2 association compared with controls, suggesting that additional endogenous miRNAs may bind the *Sdc3* 3′ UTR. Following IFN-γ stimulation, control cells exhibited increased AGO2 binding to *Sdc3* (Fig. 5a), accompanied by reduced *Sdc3* mRNA and protein levels, consistent with miR-25–mediated repression. In contrast, *Mir25* KO cells failed to show increased AGO2 association and instead accumulated *Sdc3* transcripts and protein (Fig. 5a, b; Supplementary Fig. S5a). These findings were validated in two additional murine cancer cell lines, MC38 and 4T1. IFN-γ reduced SDC3 expression in control cells but stabilized (MC38 at 48 h) or increased SDC3 (4T1 at 48 h) expression in *Mir25* KO cells (Fig. 5b; Supplementary Fig. S5b, c). Additionally, IFN-γ transiently suppressed miR-25 expression in B16 control cells, whereas IFN-γ increased miR-25 expression in MC38 and 4T1 cells (Supplementary Fig. S5d).

To assess the functional role of SDC3, we generated *Sdc3* knockout (KO) and *Mir25*/*Sdc3* double knockout (DKO) B16 cells (Fig. 5c; Supplementary Fig. S5e). While neither *Sdc3* KO nor DKO affected tumor cell proliferation in vitro (Fig. 5d), loss of SDC3 abolished the enhanced therapeutic response observed in *Mir25* KO tumors in vivo (Fig. 5e, f; Supplementary Fig. S5f). Consistent with this result, *Sdc3* KO and DKO reversed the reduction in macrophage abundance and eliminated the increase in MHC II–positive macrophages (Fig. 5g, h). The increase in C3 induced by *Mir25* KO was no longer observed in DKO tumors (Fig. 5i). These findings establish SDC3 as a critical downstream effector driving both the immune remodeling and therapeutic response associated with miR-25 loss.

These observations indicate that the *Sdc3* 3′ UTR may also be targeted by additional endogenous microRNAs (Fig. 4p, KO-WT vs. KO-MT; Fig. 5a–c, IFN-γ, 0 h). We therefore examined whether other members of the miR-25 family sharing the same seed sequence might compensate for miR-25 loss^28,29^. According to TargetScan predictions^27^, miR-25 displays the highest predicted binding affinity for *Sdc3* among six family members, featuring a 7mer-m8 site with additional base pairing at positions 1 and 9 (Fig. 5j). RT–qPCR analysis showed increased expression of miR-32, miR-92a, miR-92b and miR-367 in *Mir25* KO B16 cells at baseline (0 h without IFN-γ), indicating these miRNAs may exert even stronger inhibition effects on targets^30^ (Fig. 5k). Following IFN-γ stimulation, miR-32, miR-92a, and miR-367 remained elevated in *Mir25* KO cells, but this response declined between 24 and 72 h and did not prevent accumulation of SDC3 expression (Fig. 5k; and Supplementary Fig. S5a). These results indicate that miR-25 is the dominant regulator of SDC3 under IFN-γ conditions, consistent with the predicted binding affinity.

Finally, to directly test the functional importance of the miR-25–SDC3 interaction, we disrupted the miR-25 binding site within the *Sdc3* 3′ UTR using an adenine base editor ABE8e^31^ (Fig. 5l; and Supplementary Fig. S5g). Edited cells showed increased *Sdc3* expression compared to both control and *Mir25* KO following IFN-γ stimulation (Fig. 5m). When transplanted into mice, these cells exhibited enhanced responses to immunotherapy, with earlier tumor control and a magnitude comparable to that observed in *Mir25* KO tumors (Fig. 5n, o; and Supplementary Fig. S5h). These results demonstrate that disruption of the miR-25 binding site is sufficient to phenocopy miR-25 loss, establishing SDC3 as a key mediator of tumor immune evasion under anti-PD-1 therapy. Together, these findings define a miR-25–SDC3 axis that links IFN-γ signaling to tumor immune evasion during immune checkpoint therapy.

### miR-25–SDC3 axis may promote initial resistance to immunotherapy in human cancers

**Fig. 6:**
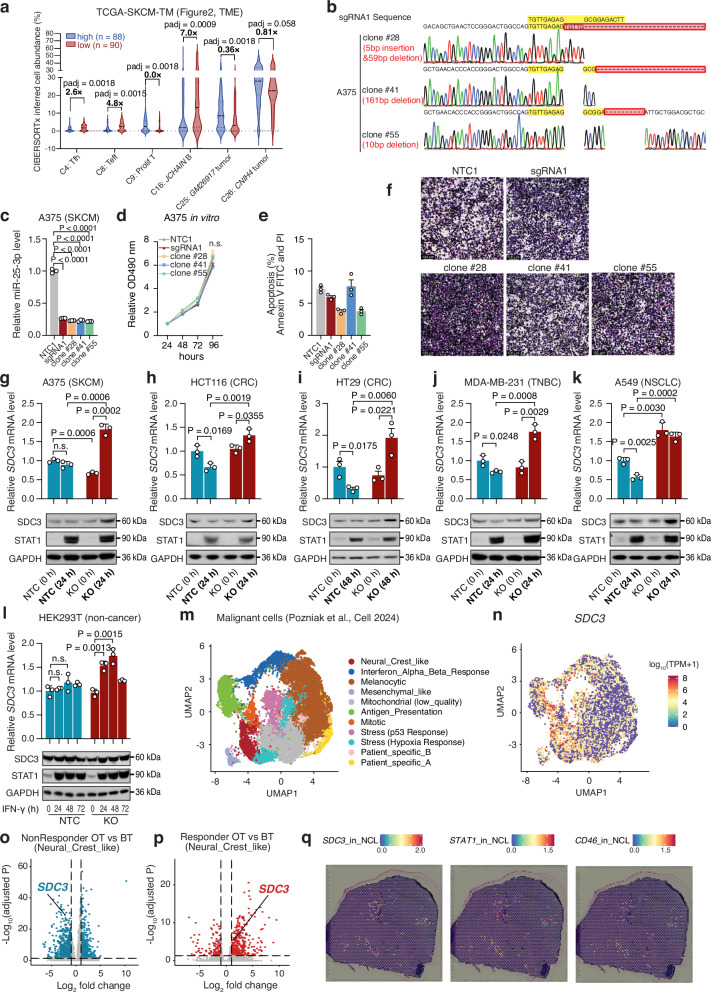
miR-25 represses SDC3 through an IFN-γ–dependent mechanism in human cancer. **a** Violin plots of CIBERSORTx-inferred cell abundance in TCGA-SKCM-TM tumors (miR-25 high, *n* = 88 patient tumors; low, *n* = 90 patient tumors). Fold change indicates median ratio (low vs. high). Tfh, T follicular helper cell; Teff, effector T cell; Prolif T, proliferating T cell. **b** Representative Sanger sequencing chromatograms of CRISPR-edited clones. **c** RT–qPCR quantification of mature miR-25-3p (mean ± SD; *n* = 3 independent experiments). **d** In vitro proliferation assay of A375 cells (mean ± SD; *n* = 6 independent experiments). **e** Apoptosis analysis in A375 cells (mean ± SD; *n* = 3 independent experiments). Q2 indicates late apoptotic cells. No statistical analysis performed due to minimal apoptosis. **f** Representative crystal violet staining of migrated A375 cells. Scale bars, 100 μm. Experiments were repeated three times. **g**–**l** RT–qPCR and immunoblot analysis of SDC3 and STAT1 comparing *MIR25* knockout (KO) and non-targeting control (NTC) cells. Representative blots from three independent experiments. Data are mean ± SD (*n* = 3 independent experiments). **m** UMAP of melanoma subclusters from Pozniak et al. (Cell, 2024). **n** Feature plot showing *SDC3* expression log_10_(TPM + 1). **o**, **p** Volcano plots comparing gene expression in neural-crest-like cells from non-responders (on-treatment (OT) vs. before treatment (BT)) (**o**) and responders (OT vs. BT) (**p**). *SDC3* highlighted. **q** Spatial transcriptomic visualization of *SDC3*, *STAT1*, and *CD46* expression in neural-crest-like melanoma cells (log-normalized values). Statistical significance in (**a)** was assessed using two-sided Mann–Whitney test with Benjamini–Hochberg correction. Statistical significance in (**d**) was assessed using two-way ANOVA with Tukey’s multiple comparisons test. Statistical significance in (**c**, **g**–**l**) was assessed using two-sided Student’s t test. Differential expression in (**o**, **p**) was performed using DESeq2 with two-sided Wald test and Benjamini–Hochberg correction. Source data are provided in the Source Data file.

To determine whether the miR-25–SDC3 axis is associated with changes in the human tumor microenvironment (TME), we performed CIBERSORTx^32^ deconvolution of TCGA-SKCM metastatic tumors using a single-cell–derived reference from our dataset (Fig. 2; reference data in Supplementary Dataset 12). Tumors with low miR-25 expression exhibited increased fractions of effector and humoral immune populations, including follicular helper T cells, effector T cells, and *JCHAIN* B cells, together with a marked reduction in proliferating T cells and tumor cell fractions (Fig. 6a). These results indicate that low miR-25 expression is associated with a more immune-active and less tumor-dominant TME in human melanoma, consistent with the immune-permissive state observed upon miR-25 loss in mice.

To assess miR-25 function, we generated *MIR25* knockout models across multiple human cell lines. Consistent with prior mouse genetics showing that deletion of the entire miR-106b ~ 25 cluster produces viable and fertile animals without overt abnormalities^29^, homozygous loss of miR-25 did not affect HEK293T viability (Supplementary Fig. S6a). Similarly, miR-25 loss did not affect cell viability, proliferation, migration, or apoptosis in either malignant (melanoma A375, colon cancer HCT116 and HT29, triple-negative breast cancer MDA-MB-231, non-small-cell lung cancer A549) or non-malignant cells (Fig. 6b–f; Supplementary Fig. S6a–j; Supplementary Dataset 4). These findings indicate that miR-25 is dispensable for tumor cell–intrinsic fitness, supporting a primary role in modulating tumor–immune interactions.

To determine whether miR-25–SDC3 regulation is conserved in human cancer, we examined SDC3 expression following IFN-γ stimulation. Across multiple human cancer cell lines, IFN-γ either failed to induce (A375) or reduced (HCT116, HT29, MDA-MB-231, and A549) SDC3 expression, whereas *MIR25* knockout attenuated this repression and enhanced SDC3 at both mRNA and protein levels (Fig. 6g–k). In non-malignant HEK293T cells, IFN-γ did not repress SDC3 mRNA expression and instead exhibited a modest increasing trend in control cells, with *MIR25* KO further enhancing SDC3 mRNA levels within 48 h. However, this effect was not reflected at the protein level, as no appreciable difference in SDC3 abundance was observed between control and *MIR25*-deficient cells following IFN-γ treatment (Fig. 6l). These findings indicate that miR-25 mediates IFN-γ–dependent repression of SDC3 across multiple human cancer cell lines and support a context-dependent, tumor-selective regulatory mechanism.

To assess the clinical relevance of SDC3, we analyzed published scRNA-seq datasets from melanoma patients treated with immune checkpoint therapy (ICT). Analysis of published datasets revealed an immune-resistance program in post-treatment malignant cells characterized by coordinated repression of *SDC3*, MHC class I antigen-presentation genes (*HLA-A*, *HLA-B*), and the complement component *C4A*^33^. A second independent dataset further identified malignant cells from pre-treatment (BT) and early on-treatment (OT) samples of distinct melanoma cell states^34^ (Fig. 6m). Within malignant cells, *SDC3* was highly expressed in antigen-presenting and neural-crest-like states (Fig. 6n). Differential expression analysis comparing ICT responders and non-responders revealed that *SDC3* decreased in neural-crest-like melanoma cells of non-responders (OT vs BT) but increased in responders (Fig. 6o, p; Supplementary Dataset 13). Notably, baseline *SDC3* expression (BT) was lower in responders than in non-responders (Supplementary Fig. S6k). Finally, spatial transcriptomics analysis of a pre-treatment immune-desert melanoma sample revealed mutually exclusive neural-crest-like and melanocytic regions^34,35^ (Fig. 6m; and Supplementary Fig. S6l, m). Within neural-crest-like tumor regions, *SDC3* spatially colocalized with *STAT1* and complement-related genes (*CD46*), consistent with IFN-driven signaling and complement regulation (Fig. 6q). These observations place SDC3 at the intersection of innate and humoral immune regulation in melanoma. Together, these findings define a miR-25–SDC3 axis that links IFN-γ signaling to complement regulation in human melanoma and implicate this pathway in early immune evasion and resistance to immunotherapy.

## Discussion

**Fig. 7:**
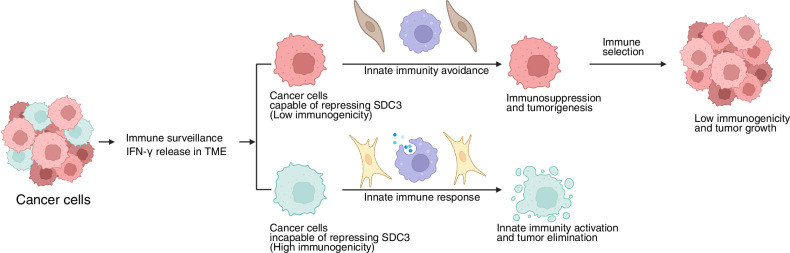
Proposed model of the miR-25–SDC3 regulatory axis in immune escape and immunotherapy resistance. Immune surveillance in the tumor microenvironment (TME) induces interferon-γ (IFN-γ) signaling, leading to suppression of SDC3 in tumor cells. Tumor cells capable of repressing SDC3 exhibit reduced immunogenicity, enabling evasion of innate immune responses and promoting immunosuppression and tumor progression. In contrast, tumor cells that fail to downregulate SDC3 remain immunogenic, triggering innate immune activation and tumor elimination. Through immune selection, SDC3-low tumor clones undergo clonal expansion and establish a stable immune-evasive state. Upon immune checkpoint therapy, this program is re-engaged, resulting in attenuated innate and humoral anti-tumor immunity and contributing to primary resistance (created with BioRender.com).

Here we show that tumor-intrinsic miR-25, a paralog of the miR-17 ~ 92 cluster, drives resistance to immune checkpoint therapy (ICT). Using scRNA-seq and flow cytometry, we demonstrate that the loss of miR-25 activates antigen-presenting macrophages and induces complement-associated inflammatory programs in CAFs, resulting in a more inflammatory tumor microenvironment (TME). Consistent with this, melanoma tumors with low miR-25 expression in TCGA cohorts exhibit increased complement activation and improved survival. Mechanistically, miR-25 represses SDC3 through the canonical miRNA silencing pathway in response to IFN-γ signaling. Disruption of this regulation restores SDC3 expression and enhances antitumor immunity. We propose that the miR-25–SDC3 regulatory program emerges during immune editing, where tumor cells unable to repress SDC3 are eliminated and SDC3-low clones are selected. This program may be re-engaged during immunotherapy and contribute to primary resistance to ICT by attenuating innate and humoral antitumor immunity (Fig. 7).

The miR-17 ~ 92 cluster was reported to be amplified in 70 cases of human B-cell lymphoma in 2004^36^. miR-17 ~ 92 cluster has been termed oncomiR-1 because of its well-documented oncogenic roles. miR-25, a member of the miR-106b ~ 25 cluster, is reported to be amplified in both malignancies and autoimmune diseases, underscoring its versatile roles in regulating diverse pathological processes^37^. Although previous studies have described oncogenic roles for miR-25^38^, our analyses across three syngeneic mouse tumor models and five human epithelial cancer cell lines revealed that neither mixed knockout populations nor homozygous *Mir25*/*MIR25* KO clones exhibited detectable changes in proliferation, apoptosis, or migration. This apparent lack of phenotype likely reflects functional redundancy within the miR-25 family, whereby loss of a single member is compensated by upregulation of seed-sharing paralogs^28^. Consistent with TargetScan predictions of binding affinity, miR-25 possesses the strongest binding to *Sdc3* among its family members and serves as the principal determinant of SDC3 repression under IFN-γ stimulation^27,39,40^. ABE8e–mediated base editing that introduced an A-to-G mutation within the miR-25 binding site effectively stabilized SDC3 expression and elicited an immune response comparable to that observed in *Mir25* KO tumors in vivo. Collectively, these findings indicate that loss of miR-25 does not affect cancer cell viability, and that the survival and transcriptional differences between miR-25-low and miR-25-high patient tumors (Figs. 1e, 3b, c) arise predominantly from SDC3–dependent modulation of the immune microenvironment rather than from cancer-cell-intrinsic effects.

TAMs are key regulators of immunosuppression in the TME, where tumor-derived signals drive macrophages toward an immunosuppressive M2-like phenotype associated with tumor progression and T cell dysfunction^41^. Targeting macrophage–mediated immunosuppression has therefore been proposed as a strategy to enhance responses to ICT^19,42^. In our models, macrophage depletion reduced tumor growth but also markedly decreased complement infiltration within tumors, suggesting that TAMs contribute to both immune suppression and immune activation in the TME. Consistent with this dual role, we identified a macrophage population expressing high levels of MHC II genes (Fig. 2, C18), indicative of antigen-presenting capacity. MHC II expression supports adaptive immune responses, whereas its loss limits antigen presentation to T cells^43,44^. MIF, a regulator of macrophage alternative activation, has been linked to immune suppression in tumors, and its silencing increases MHC II⁺ TAMs and immune infiltration without affecting tumor proliferation^45–48^. Our results suggest that loss of miR-25 in tumor cells may disrupt MIF–associated signaling by promoting inflammatory CAF states, thereby reducing macrophage recruitment through CD44–dependent signaling.

CAFs exhibit substantial heterogeneity and functional plasticity within the TME. Single-cell studies have identified multiple CAF states, including myCAFs, iCAFs, and antigen-presenting CAFs (apCAFs). Although these populations perform diverse functions, many CAF–TAM interactions are associated with immunosuppressive signaling that facilitates tumor progression^49,50^. In our study, tumor miR-25 deletion during immunotherapy was associated with a shift toward iCAF states and disruption of tumor-promoting TAM–CAF interactions. Complement activation and inflammatory chemokine expression in iCAFs coincided with reduced expression of myCAF and pericyte markers. These findings suggest that complement activation in iCAFs reflects downstream humoral immune responses driven by antigen-presenting TAMs, particularly the MHC II⁺ C18 macrophage population identified in our dataset, which may provide the immune activation context enabling complement-associated inflammatory programs in CAFs. However, the molecular link between SDC3–mediated regulation of TAM antigen presentation and complement activation in CAFs remains unclear. Because MHC II signaling is classically associated with CD4⁺ T cell activation^51^ rather than direct stromal regulation, the intermediate signals connecting antigen-presenting macrophages to CAF complement programs require further investigation. Consistent with this model, macrophage depletion markedly reduced C3 infiltration within tumors, indicating that complement activation depends on antigen-presenting TAMs. Notably, apCAFs were not detected in our dataset (Fig. 2j), further suggesting that complement activation in CAFs relies on specialized antigen-presenting cells rather than intrinsic antigen presentation by CAFs.

SDC3, a transmembrane proteoglycan of the Syndecan family, carries both heparan sulfate and chondroitin sulfate chains^52–54^. SDC3 is broadly expressed across human tissues, with enrichment in neuronal cells^55,56^ (Supplementary Fig. S7a), but has also been implicated in inflammatory regulation in non-neuronal contexts, acting as a pro-inflammatory mediator in skin and anti-inflammatory in cartilage^57^. scRNA-seq studies^58^ and our data detect *Sdc3* expression in immune populations, including macrophages, as well as moderate expression in NK cells, T cells, and tumor cells (Supplementary Fig. S7b). Recent studies have linked hypoxia to tumor progression and therapy resistance in melanoma, and TCGA analyses indicate that *SDC3* is upregulated in several solid tumors associated with hypoxic environments^59,60^. Our data suggest that miR-25 suppresses SDC3 as an early tumor-intrinsic response to immune pressure. Consistently, both mouse and human cancer cells exhibited selective SDC3 downregulation following IFN-γ stimulation. Notably, scRNA-seq analysis at a late treatment time point (day 17) did not detect increased *Sdc3* expression in *Mir25* knockout tumor clusters (Supplementary Fig. S7c), suggesting that SDC3 regulation occurs predominantly during early phases of immune activation. Interestingly, we also observed reduced *Sdc3* expression in the C18 TAM population (Supplementary Dataset 6), suggesting that SDC3 in TAMs may influence macrophage polarization or antigen presentation. Because SDC3 expression varies across cell types and may change dynamically during ICT, interpretation of bulk RNA-seq measurements remains challenging, particularly in clinical samples with heterogeneous cellular composition and sampling time points.

Our data suggest that SDC3, a proteoglycan typically associated with neuronal and extracellular matrix functions, is ectopically expressed in epithelial cancers. Notably, two additional genes, *Lamb1* and *L1cam*, were co-regulated with *Sdc3* following *Mir25* KO with IFN-γ treatment (Supplementary Fig. S4i, j). Both of these genes are well known for their roles in the nervous system^61,62^. Consistent with this pattern, tumors with low miR-25 expression exhibited enhanced immune activation and downregulation of synapse-related pathways (Supplementary Fig. S7d), suggesting that immune activation is coupled to suppression of neuronal-like transcriptional programs in epithelial tumors. This neuronal-associated program was further supported by human melanoma datasets, where *SDC3* marks neural-crest-like malignant cells (Fig. 6m–p). The presence of neuronal-associated gene expression may help explain why *Mir25* KO produced similar immune phenotypes across multiple epithelial tumor models. These findings raise the possibility that SDC3 functions as an atypical surface antigen in epithelial cancers, where its ectopic expression may promote immune activation through neuronal-like inflammatory signaling.

**Fig. 8:**
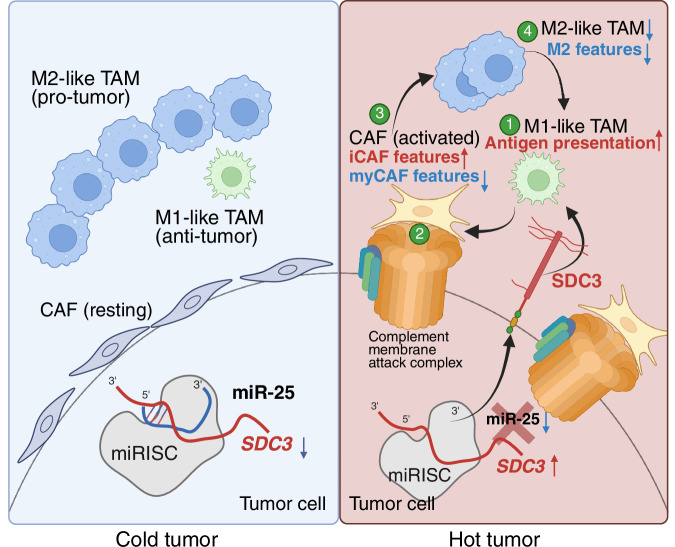
The miR-25–SDC3 axis regulates macrophage antigen presentation, complement activation, and cancer-associated fibroblast (CAF) states. In cold tumors (left), miR-25 represses SDC3 via miRNA-induced silencing complex (miRISC)–mediated silencing, leading to reduced macrophage antigen presentation, impaired classical complement activation, and maintenance of CAFs in a resting state, thereby promoting an immunosuppressive microenvironment enriched in M2-like tumor-associated macrophages (TAMs). In hot tumors (right), loss of miR-25 permits SDC3 accumulation, enhancing macrophage antigen presentation ① and activating the classical complement cascade with membrane attack complex (MAC) deposition ②. This is accompanied by cancer-associated fibroblasts (CAFs) activation toward inflammatory (iCAF) states, away from myofibroblastic (myCAF) states ③, reduced M2-like TAMs ④, and disruption of immunosuppressive macrophage–fibroblast interactions. Together, the miR-25–SDC3 axis acts as a switch controlling tumor immune state (created with BioRender.com).

In summary, our findings identify a tumor–stromal regulatory axis in which tumor-intrinsic miR-25 controls SDC3 expression to coordinate macrophage–fibroblast interactions in the TME (Fig. 8). This program contributes to early resistance to ICT and highlights the miR-25–SDC3 axis as a potential target for therapeutic intervention.

## Methods

### Ethics statement

All animal experiments were approved by the Institutional Animal Care and Use Committee (IACUC) of the University of California, San Diego, and were conducted in accordance with institutional and national guidelines. The maximal tumor size permitted by the IACUC was 2.0 cm in length, and this limit was not exceeded in any of the experiments.

### Cell lines and culture conditions

B16, 4T1, A375, HCT116, HT29, MDA-MB-231, A549, and HEK293T cell lines were obtained from the American Type Culture Collection (ATCC). MC38 cells were obtained from Kerafast. The B16-GM-CSF cell line was generously provided by Drs. Glenn Dranoff and Michael Dougan (Dana-Farber/Harvard Cancer Center). B16, MC38, A375, HCT116, MDA-MB-231, A549, and HEK293T cells were cultured in Dulbecco’s Modified Eagle Medium (DMEM), whereas 4T1 and HT29 cells were maintained in Roswell Park Memorial Institute (RPMI-1640) medium, each supplemented with 10% fetal bovine serum and 1% penicillin–streptomycin. Cells were maintained at 37 °C in a humidified atmosphere with 5% carbon dioxide (CO₂). Cells were routinely tested for mycoplasma contamination.

### CRISPR-Cas9–mediated knockout, base editing, and Mir25 overexpression

Knockout (KO) cell lines targeting *Mir25*, *Ago2*, or *Sdc3*, as well as non-targeting control (NTC) lines, were generated using CRISPR-Cas9–mediated genome editing. For each gene, at least two independent sgRNAs were designed and cloned into the lentiCRISPR v2 vector. sgRNA sequences are provided in Supplementary Dataset 3. For base editing of the *Sdc3* 3′ UTR, the adenine base editor ABE8e was expressed together with sgRNAs targeting the *Sdc3* 3′ UTR using lentiviral constructs. For miR-25 overexpression, the full-length *Mir25* genomic sequence with ~200 bp flanking regions was cloned into a lentiviral expression vector. Lentiviral particles were produced in HEK293T cells by co-transfection of transfer, packaging, and envelope plasmids. Viral supernatants were collected 48 h after transfection and used to transduce target cells. Transduced cells were selected with antibiotics to establish stable cell populations. Genome editing efficiency was assessed by PCR amplification of target loci followed by Sanger sequencing and analysis using TIDE or EdiR^63^. Editing was further validated by RT–qPCR, immunoblotting, or T7 endonuclease I assay.

### CRISPR-Cas9 RNP electroporation and single-cell clone isolation

Chemically modified sgRNAs were synthesized (GenScript) and complexed with recombinant Cas9 protein to form ribonucleoprotein (RNP) complexes. Human and mouse cancer cell lines were electroporated with RNP complexes using a 4D-Nucleofector system (Lonza)^64,65^. Cells were allowed to recover in complete medium following electroporation. Single-cell clones were isolated by limiting dilution and screened by PCR amplification of target loci followed by Sanger sequencing.

### Cell proliferation assay

Cell proliferation was measured using a colorimetric cell viability assay (Promega). Tumor cells were seeded at a density of 1,000 cells per well in 96-well plates. Cell proliferation was assessed at 24, 48, 72, and 96 h after seeding by measuring absorbance at 490 nm. Data are presented as fold change relative to the 24 h time point.

### Cell migration and apoptosis assay

For migration assays, cells were serum-starved for 24 h and then seeded into Transwell inserts (8-μm pore size, Corning) in serum-free medium. Medium supplemented with serum was added to the lower chamber as a chemoattractant. After 24 h, migrated cells were fixed and stained with crystal violet (Sigma-Aldrich). For apoptosis assays, cells were cultured under serum-deprived conditions for 24 h, followed by staining with Annexin V–FITC and propidium iodide (BioLegend). Apoptotic cells were analyzed by flow cytometry.

### Tumor challenge and treatments

Mice were housed under standard institutional conditions with a 12 h light/12 h dark cycle, controlled ambient temperature (20–24 °C), and relative humidity (40–60%). Female mice were used in all experiments to minimize variability associated with sex-dependent immune responses. Sex was considered in the study design; however, comparisons between sexes were not performed. C57BL/6J or BALB/c mice aged 9–12 weeks were used for all experiments. For the B16 melanoma model, 5 × 10^5^ B16 cells were injected subcutaneously into the left flank of C57BL/6J mice on day 0. Mice received 1 × 10^6^ irradiated B16-GM-CSF cells (GVAX) in the contralateral flank on days 1 and 4. Anti-PD-1 antibody or isotype control (rat IgG, Bio X Cell) was administered intraperitoneally at 10 mg kg⁻¹ on days 6, 9, and 12. For macrophage depletion experiments, mice received 200 μL liposome clodronate (Fisher Scientific) intravenously on days 8 and 11. For IFN-γ depletion, mice were treated with 10 μg anti-mouse IFN-γ antibody (clone XMG1.2, Bio X Cell) intraperitoneally on days 8 and 11. For the MC38 model, 5 × 10^5^ MC38 cells were injected subcutaneously into C57BL/6J mice, followed by anti-PD-1 antibody or isotype control treatment starting on day 9 until endpoint. For the 4T1 model, 2 × 10^6^ 4T1 cells were injected subcutaneously into BALB/c mice, followed by anti-PD-1 antibody or isotype control treatment starting on day 9 until endpoint. Tumor length and width were measured every 2 days using calipers, and tumor volume was calculated as (length × width^2^)/2. For survival analyses, mice were euthanized when tumors reached the humane endpoint defined by the approved institutional guidelines.

### Tumor dissociation and flow cytometry of immune cells

Tumors were mechanically dissociated and enzymatically digested using a tumor dissociation kit (Miltenyi Biotec). Spleens were minced and passed through a 70 μm cell strainer (Corning) to obtain single-cell suspensions. Red blood cells were lysed using RBC lysis buffer (BioLegend). Cells were incubated with Fc receptor–blocking antibody (BioLegend) and stained with a viability dye (BioLegend) to exclude dead cells, followed by staining with antibodies against cell surface markers. Cells were analyzed by flow cytometry. Compensation controls (BD Biosciences), fluorescence-minus-one (FMO) controls, and unstained controls were included to establish gating strategies. Gating strategies for immune cell subsets are shown in Supplementary Fig. S3. Antibody clone information is provided in the Reporting Summary.

### RNA isolation and RT–qPCR

Total RNA was isolated using column–based RNA extraction kits (Zymo Research). Complementary DNA (cDNA) was synthesized using reverse transcription kits for mRNA (Bio-Rad) or microRNA (Takara). Quantitative real-time PCR (RT–qPCR) was performed using SYBR Green Supermix (Bio-Rad) on a real-time PCR system (Applied Biosystems QuantStudio™ 7). Gene expression levels were normalized to *Gapdh*/*GAPDH* mRNA, and microRNA expression levels were normalized to U6 snRNA. Relative expression was calculated using the 2⁻ΔΔCt method. Primer sequences are provided in Supplementary Dataset 3.

### RNA sequencing (RNA-seq)

Total RNA was extracted from at least two biologically independent samples under the indicated experimental conditions. RNA-seq libraries were prepared using standard mRNA or small RNA library preparation kits (Illumina) and sequenced on a NovaSeq 6000 platform (Illumina) at the IGM Genomics Center, University of California, San Diego. Raw sequencing reads were assessed for quality control and processed to remove adapter sequences and low-quality bases. Clean reads were aligned to the mm10 reference genome, and gene-level counts were generated. Differential expression analysis was performed using DESeq2, and functional enrichment analysis was conducted using clusterProfiler.

### Single-cell RNA sequencing (scRNA-seq) and spatial transcriptomic analysis

Single-cell RNA sequencing was performed using the Chromium Single Cell 3′ platform (10x Genomics). Tumor tissues were dissociated into single-cell suspensions, and red blood cells and dead cells were removed to enrich for viable cells. Approximately 20,000 cells per sample were loaded for library preparation and sequencing. Raw sequencing data were processed using Cell Ranger and aligned to the mm10 mouse reference genome to generate gene–barcode matrices. Downstream analyses were performed using the Seurat R package. Cells were filtered to retain those with more than 100 and fewer than 2500 detected genes and less than 25% mitochondrial transcripts. Gene expression values were normalized followed by principal component analysis and clustering based on the first 50 principal components with a clustering resolution of 1. Cluster marker genes were identified using Seurat’s differential expression framework and defined as genes expressed in at least 25% of cells within a cluster. Spatial transcriptomic datasets were obtained from a previously published study and reanalyzed using the processed data and analytical framework described in the original publication^34^.

### InferCNV and cell–cell communication analysis

Chromosomal copy number variations (CNVs) were inferred using the inferCNV R package, with naïve T cells as the reference population. MC38 tumor cells were identified based on marker gene *Shisal2b* expression and analyzed to distinguish malignant from non-malignant populations^22^. Cell–cell communication analysis was performed using CellChat to infer intercellular signaling interactions based on a curated ligand–receptor interaction database. Sender and receiver cell types were defined according to annotated cell populations from whole-tumor scRNA-seq data. Overexpressed genes and significant ligand–receptor interactions were identified using standard criteria implemented in CellChat^20^.

### Western blot analysis

Cells were lysed in lysis buffer supplemented with protease inhibitors (Thermo Fisher Scientific). Lysates were clarified by centrifugation, and protein concentrations were determined using a BCA assay (Thermo Fisher Scientific). Equal amounts of protein were resolved by SDS–PAGE (Invitrogen) and transferred to polyvinylidene fluoride (PVDF) membranes (Bio-Rad). Membranes were blocked with 5% non-fat milk in Tris-buffered saline containing 0.1% Tween-20 (TBST) and incubated with primary antibodies overnight at 4 °C, followed by incubation with horseradish peroxidase (HRP)-conjugated secondary antibodies for 1 h at room temperature. Protein bands were detected using enhanced chemiluminescence by autoradiography films (RPI Research). Antibody information is provided in the Reporting Summary. Uncropped scans of all blots and gels are provided in the Source Data file.

### Immunohistochemistry

Paraffin-embedded tumor sections were deparaffinized, rehydrated, and subjected to antigen retrieval. Endogenous peroxidase activity was quenched, followed by blocking and incubation with primary antibodies overnight at 4 °C. Sections were incubated with biotinylated secondary antibodies and developed using a peroxidase-conjugated avidin–biotin complex (Vector Laboratories) followed by using an AEC (3-Amino-9-ethylcarbazole) chromogen substrate (Vector Laboratories). Sections were counterstained with hematoxylin (Sigma-Aldrich). Images were acquired using a light microscope at indicated magnifications. Multiple non-overlapping fields per sample were analyzed. Image acquisition and selection were performed in a blinded manner, and image processing was applied uniformly across all samples.

### RNA immunoprecipitation

RNA immunoprecipitation (RIP) followed by qPCR was performed with minor modifications^66^. Cells were lysed, and clarified lysates were precleared with protein A magnetic beads (Invitrogen). A fraction of the lysate was reserved as input. The remaining lysate was incubated overnight at 4 °C with an anti-AGO2 antibody (Abcam) under gentle rotation. Beads were washed three times with polysome lysis buffer and three additional times with lysis buffer containing 1 M urea (Sigma-Aldrich) to reduce nonspecific interactions. RNA was extracted from input and immunoprecipitated fractions using phenol–chloroform–isoamyl alcohol, reverse transcribed (Bio-Rad), and analyzed by qPCR to assess enrichment of target transcripts.

### Endogenous miRNA luciferase assay

A full-length *Sdc3* 3′ UTR containing conserved miR-25 binding sites was cloned into a luciferase reporter vector (Invitrogen). A deletion construct lacking the miR-25 binding sites was generated to assess binding specificity. B16 cells with functional miR-25 (NTC) or *Mir25* knockout were transfected with luciferase reporter constructs together with a β-galactosidase control plasmid (Invitrogen). Cells were treated with mouse IFN-γ (BioLegend) for 72 h. Luciferase activity was measured using a luciferase assay kit (Promega) and normalized to β-galactosidase activity.

### Gene expression analysis of human tumors

Small RNA-seq data from The Cancer Genome Atlas skin cutaneous melanoma (TCGA-SKCM) were analyzed to examine the association between miR-25 expression, patient survival, and downstream gene expression. Samples were stratified into high and low miR-25 expression groups based on quartile distribution. Survival analysis was performed to assess the association between miR-25 expression and patient outcomes. Corresponding gene expression data were used for differential expression analysis to identify differentially expressed genes between groups. Gene set enrichment analysis (GSEA) was performed to identify pathways associated with high or low miR-25 expression using gene sets from the Molecular Signatures Database (MSigDB), including Hallmark and Kyoto Encyclopedia of Genes and Genomes (KEGG).

### Estimated cell-type composition in TCGA-SKCM

Cell-type composition in TCGA-SKCM metastatic melanoma samples was estimated using CIBERSORTx to deconvolve bulk RNA-seq profiles and infer relative fractions of tumor, immune, stromal, and endothelial cell populations. Samples were stratified into high and low hsa-miR-25-3p expression groups, and corresponding bulk RNA-seq profiles were used as input. A custom signature matrix was generated from our whole-tumor scRNA-seq dataset (Fig. 2). Cell clusters were defined using Seurat, and representative marker genes from each cluster were used to construct the signature matrix. Mouse genes were mapped to human orthologs, and unmapped or duplicated genes were removed prior to analysis. Deconvolution was performed using the single-cell reference mode in CIBERSORTx, and results are reported as relative cell-type fractions. The finalized signature matrix is provided in Supplementary Dataset 12.

### Statistical analysis

All statistical analyses were performed using GraphPad Prism (GraphPad Software). The specific statistical tests used for each experiment are indicated in the corresponding figure legends. Data are presented as mean ± standard error of the mean (SEM) or mean ± standard deviation (SD), as indicated in the figure legends. Unless otherwise specified, statistical tests were two-sided. *P* < 0.05 was considered statistically significant. Differential expression analysis of RNA-seq data was performed using DESeq2. *P* values were adjusted for multiple testing using the Benjamini–Hochberg false discovery rate (FDR) correction. Genes with an absolute log₂ fold change (|log₂FC |) > 1 and an adjusted *P* value < 0.05 were considered significantly differentially expressed.

### Reporting summary

Further information on research design is available in the Nature Portfolio Reporting Summary linked to this article.

## Supplementary information

Supplementary Information

Description of Additional Supplementary Files

Supplementary Dataset 1

Supplementary Dataset 2

Supplementary Dataset 3

Supplementary Dataset 4

Supplementary Dataset 5

Supplementary Dataset 6

Supplementary Dataset 7

Supplementary Dataset 8

Supplementary Dataset 9

Supplementary Dataset 10

Supplementary Dataset 11

Supplementary Dataset 12

Supplementary Dataset 13

Reporting Summary

Transparent Peer Review file

## Source data

Source Data

## Data Availability

RNA-seq and scRNA-seq data generated in this study have been deposited in the Gene Expression Omnibus (GEO) under accession number GSE313080. Publicly available datasets used in this study include: single-cell and spatial transcriptomic datasets from Pozniak et al. (Cell, 2024) (https://rdr.kuleuven.be/dataset.xhtml?persistentId=doi:10.48804/GSAXBN); data from Jerby-Arnon et al. (Cell, 2018) (https://www.ncbi.nlm.nih.gov/geo/query/acc.cgi?acc=GSE115978); The Cancer Genome Atlas Skin Cutaneous Melanoma (TCGA-SKCM) dataset (https://portal.gdc.cancer.gov/projects/TCGA-SKCM); and protein expression data from the Human Protein Atlas (https://www.proteinatlas.org/), including *SDC3* (https://www.proteinatlas.org/ENSG00000162512-SDC3), *LAMB1* (https://www.proteinatlas.org/search/lamb1), and *L1CAM* (https://www.proteinatlas.org/ENSG00000198910-L1CAM). All other data supporting the findings of this study are included within the article, Supplementary Information, or Source Data files. Source data are provided with this paper.
