# Supplementary material for: microRNA-25 drives immune checkpoint therapy resistance by repressing innate and humoral immunity via Syndecan-3
Source: Nat Commun. 2026 May 20;17:6666. doi: 10.1038/s41467-026-73339-y (PMC13381856; doi:10.1038/s41467-026-73339-y)
Supplement: Supplementary file 1 — Supplementary Information [file 41467_2026_73339_MOESM1_ESM.pdf]

Supplementary information for

microRNA-25 drives immune checkpoint therapy resistance by repressing innate and humoral immunity via Syndecan-3

Zhouting Zhu[1,2], Wenyan Han[1], Yufei Deng[1], Zhaoyang Jia[1], Gulshanbir Baidwan[1], Lujing Wu[1], Shweta Jakhmola[1], Tongyun Wang[1], Dhenugen Logeswaran[1], Jing Wen[1], Amanda Y. Sun[1], Bill Bray[1], Na Li[1], Lingling Wang[1], Hui Hui[1], Jiaqian Wu[2], Sandip Pravin Patel[3], Tariq M. Rana[1,3*]

[1]Department of Cellular and Molecular Medicine, University of California San Diego, 9500 Gilman Drive, La Jolla, California 92093, USA
[2]Graduate School of Biomedical Sciences, Sanford Burnham Prebys Institute, 10901 North Torrey Pines Road, La Jolla, California 92037, USA
[3]Moores Cancer Center, 3855 Health Sciences Drive, University of California San Diego, La Jolla, California 92093, USA

*Correspondence: trana@ucsd.edu

Supplementary information includes:

Supplementary Figs. 1 to 7

Supplementary Figs

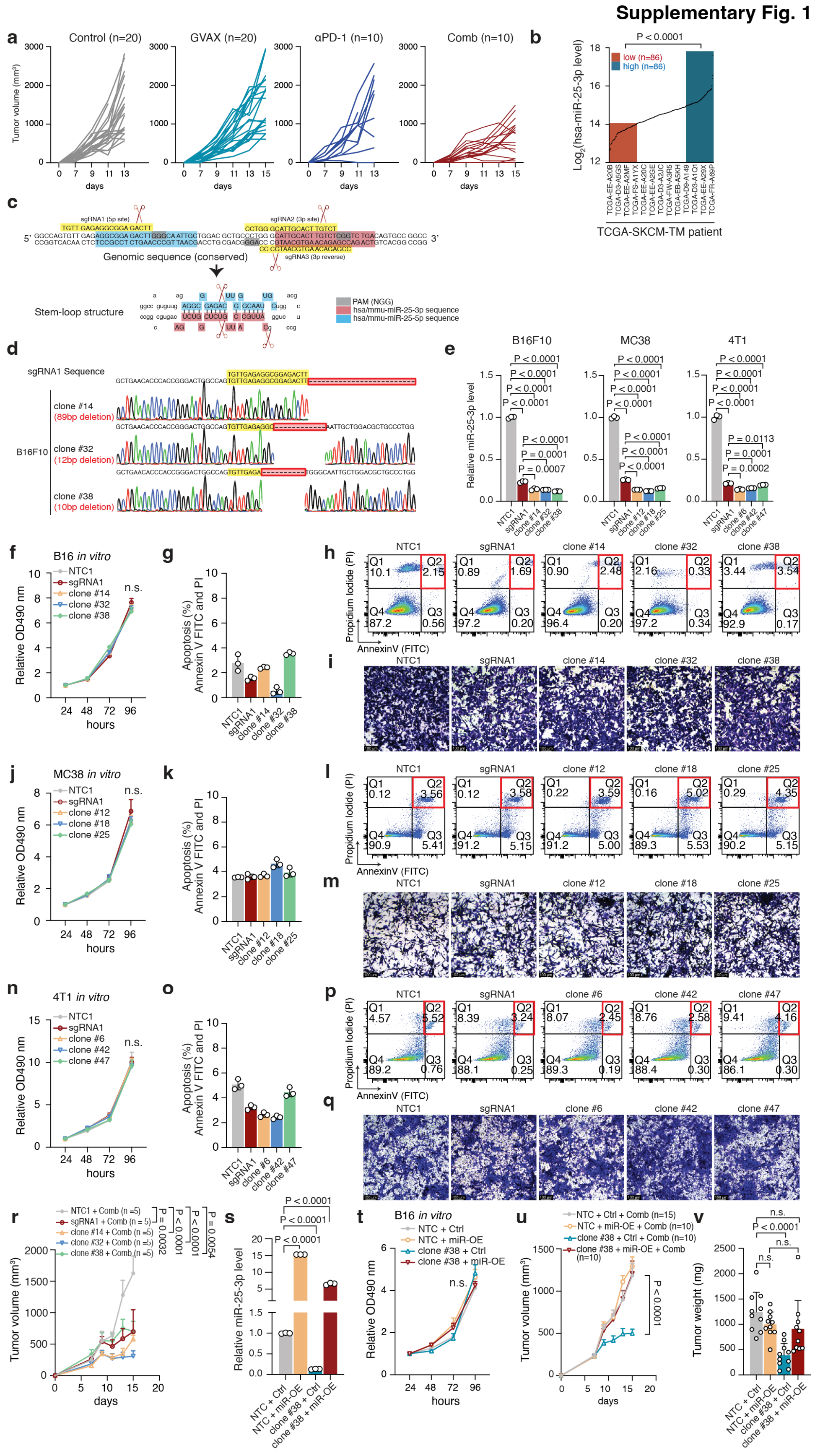
Supplementary Fig. 1
Control (n=20)
GVAX (n=20)
αPD-1 (n=10)
Comb (n=10)
Tumor volume (mm³)
days
P < 0.0001
low (n=86)
high (n=86)
TCGA-SKCM-TM patient
Genomic sequence (conserved)
Stem-loop structure
PAM (NGG)
hsa/mmu-miR-25-3p sequence
hsa/mmu-miR-25-5p sequence
sgRNA1 Sequence
clone #14
(89bp deletion)
clone #32
(12bp deletion)
clone #38
(10bp deletion)
B16F10
MC38
4T1
Relative miR-25-3p level
B16 in vitro
MC38 in vitro
4T1 in vitro
Relative OD490 nm
hours
n.s.
Apoptosis (%)
Annexin V FITC and PI
Propidium Iodide (PI)
AnnexinV (FITC)
NTC1
sgRNA1
Tumor volume (mm³)
Tumor weight (mg)
NTC + Ctrl
NTC + miR-OE
clone #38 + Ctrl
clone #38 + miR-OE

Supplementary Fig. 1: Validation of miR-25 perturbation and functional effects in tumor models

(a) Tumor growth curves of B16F10 tumors in mice. Each line represents one tumor from an individual mouse. n indicates independent mice; exact sample sizes are shown in the figure. **(b)** miR-25-3p expression in TCGA-SKCM-TM samples. n = 86 patients per group. Each point represents one patient. **(c)** Schematic of sgRNA targeting sites within the conserved miR-25 hairpin region in mouse and human (created with BioRender.com). **(d)** Representative Sanger sequencing chromatograms showing CRISPR–Cas9-induced deletions at the *Mir25* genomic locus. **(e)** RT–qPCR quantification of mature miR-25-3p in B16F10, MC38, and 4T1 cells. Data are mean ± SD from n = 3 independent experiments. **(f, j, n)** In vitro proliferation assays of B16F10 (f), MC38 (j), and 4T1 (n) cells. Data are mean ± SD from n = 6 independent experiments. **(g–h, k–l, o–p)** Apoptosis analyses in B16F10 (g, h), MC38 (k, l), and 4T1 (o, p) cells. Quantification of apoptotic cells (g, k, o) is presented as mean ± SD from n = 3 independent experiments. Q2 indicates late apoptotic cells. No statistical analysis was performed as apoptosis levels were minimal across conditions. **(i, m, q)** Representative crystal violet staining of migrated cells in B16F10 (i), MC38 (m), and 4T1 (q) cells. Experiments were independently repeated three times with similar results. Scale bars, 100 μm. **(r)** Tumor growth curves of B16F10 tumors from the indicated groups. Data are mean ± SEM. n indicates independent mice; exact sample sizes are shown in the figure. **(s)** RT–qPCR quantification of miR-25-3p expression in NTC1 and clone #38 cells transduced with control (Ctrl) or miR-25 overexpression (miR-OE) lentivirus. Data are mean ± SD from n = 3 independent experiments. **(t)** In vitro proliferation assays of the indicated cells. Data are mean ± SD from n = 6 independent experiments. **(u)** Tumor growth curves of the indicated groups under combination therapy. Data are mean ± SEM. n indicates independent mice; exact sample sizes are shown in the figure. **(v)** Tumor weights measured at the endpoint of the experiment shown in (u). Data are mean ± SD. n indicates independent mice (n = 10 per group). Each point represents one tumor from an individual mouse. Statistical significance in (b, e, s, v) was assessed using two-sided Student’s t test. Statistical significance in (f, j, n, r, t, u) was assessed using two-way ANOVA with Tukey’s multiple comparisons test. Source data are provided in the Source Data file.

Supplementary Fig. 2

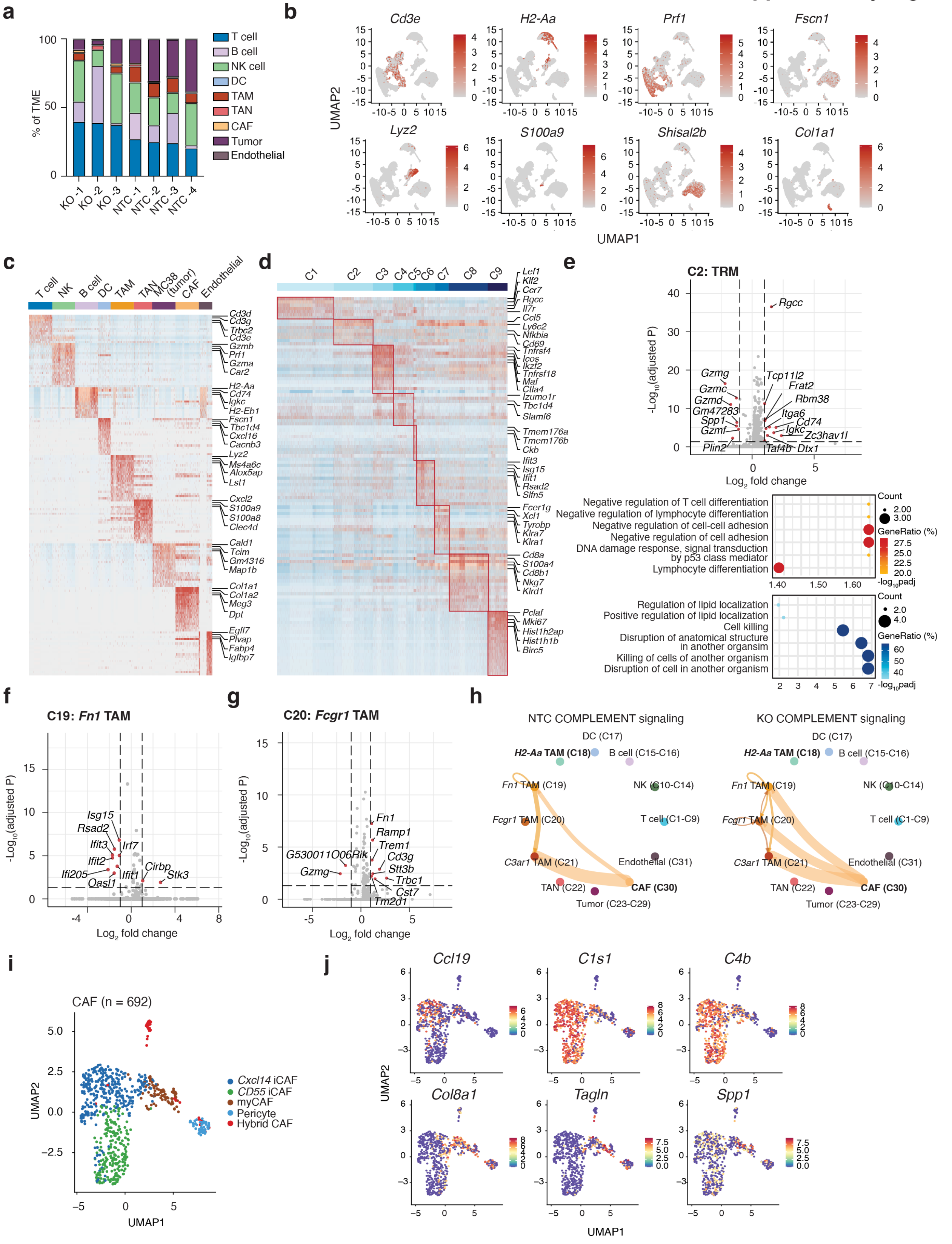

Supplementary Fig. 2: Single-cell characterization of immune and stromal populations in the tumor microenvironment

(a) Proportion of major cell types identified across scRNA-seq samples. Each bar represents one mouse. n = 3 tumors per group (*Mir25* KO) and n = 4 tumors per group (NTC*)*. **(b)** Feature plots showing expression of representative marker genes defining the major cell types shown in Fig. 2a. **(c)** Heatmap showing the top marker genes defining major cell populations identified in the scRNA-seq dataset. **(d)** Heatmap showing the top marker genes distinguishing T cell subclusters. **(e)** Analysis of tumor-resident memory T cells (C2) in *Mir25* KO tumors. Upper, volcano plot showing differentially expressed genes. Lower, Gene Ontology enrichment analysis showing upregulated (red) and downregulated (blue) pathways. **(f, g)** Volcano plot showing differentially expressed genes in C19 TAM (f) and C20 TAM (g) comparing *Mir25* KO and NTC tumors. **(h)** Circle diagram showing complement signaling interactions within the tumor microenvironment inferred based on ligand–receptor analysis. **(i)** UMAP visualization of CAFs and their annotated subclusters (n = 692 CAF cells). **(j)** UMAP visualization of CAFs showing expression of the indicated marker genes. Gene expression is shown as $\log_{10}$(TPM + 1). Differential expression analyses in (e–g) were performed using DESeq2 with a two-sided Wald test and Benjamini–Hochberg correction. Gene Ontology enrichment analyses were performed using clusterProfiler with Benjamini–Hochberg correction. Source data are provided in the Source Data file.

Supplementary Fig. 3

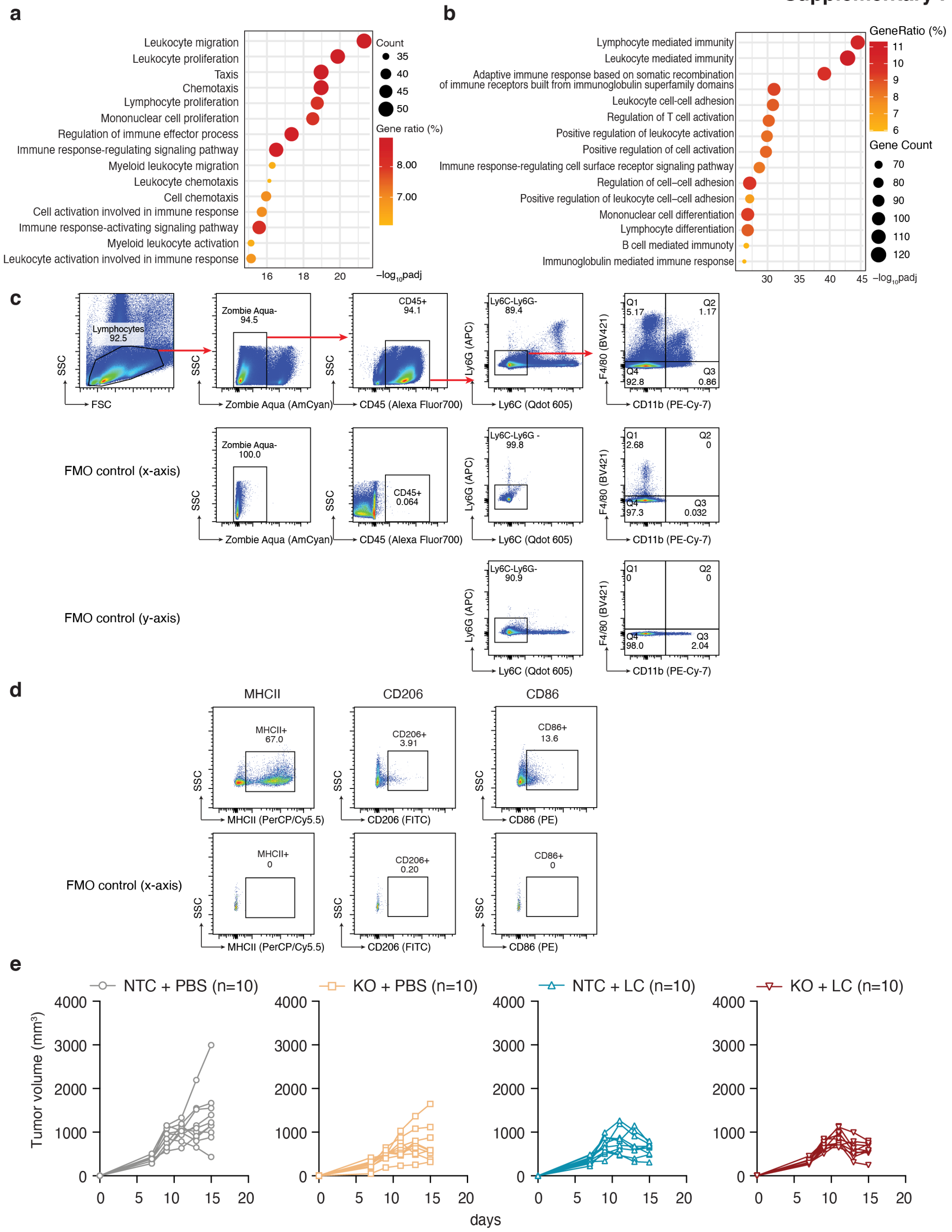

Supplementary Fig. 3: Macrophage characterization and functional analysis of miR-25–dependent tumor responses

(a, b) Gene Ontology analysis of differentially expressed genes in the tumor microenvironment of *Mir25* KO B16F10 tumors versus control tumors (a) and in miR-25 low- versus high-expressing TCGA-SKCM-TM samples (b). Upregulated pathways are shown in red. **(c)** Representative flow cytometry gating strategy for identifying macrophages. Fluorescence minus one (FMO) controls were used as negative controls. **(d)** Representative gating strategy for defining macrophage subpopulations based on MHCII, CD206, and CD86 expression. All gated cells were derived from the macrophage population defined in (c). FMO controls were used as negative controls. **(e)** Individual tumor growth curves of B16F10 tumors (NTC or KO) in mice treated with phosphate-buffered saline (PBS) or liposome clodronate (LC) in combination with GVAX and anti–PD-1 therapy. Each line represents one tumor from an individual mouse. n indicates independent mice; exact sample sizes are shown in the figure. NTC, non-targeting control; KO, *Mir25* knockout. Differential expression analysis in (a) was performed using DESeq2 with a two-sided Wald test, and in (b) using edgeR with a quasi-likelihood F-test (two-sided), with Benjamini–Hochberg correction for multiple comparisons. Gene Ontology enrichment analysis was performed using clusterProfiler with Benjamini–Hochberg correction. Source data are provided in the Source Data file.

Supplementary Fig. 4

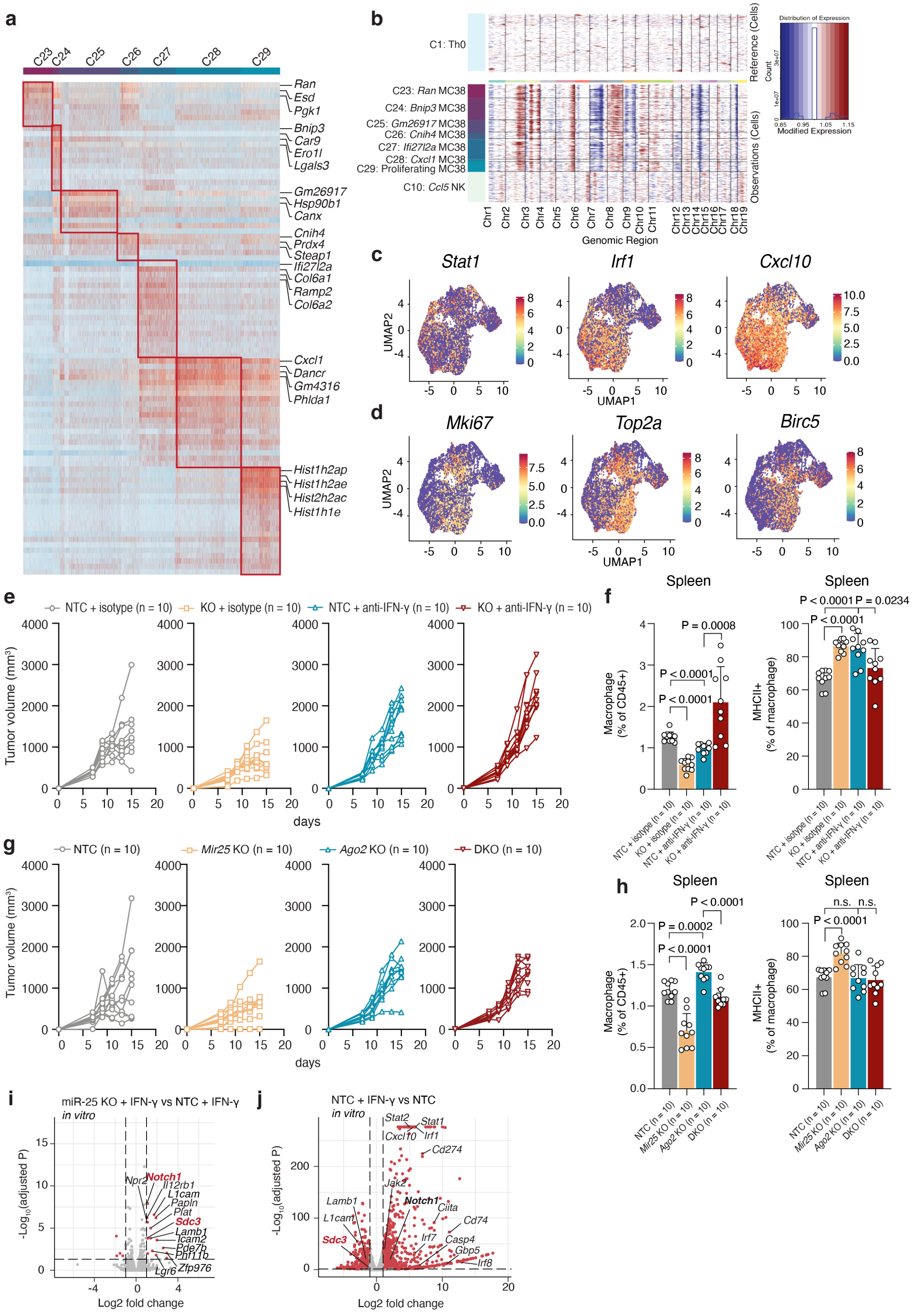

Supplementary Fig. 4: Tumor cell–intrinsic and IFN-γ–dependent responses associated with miR-25 deficiency

(a) Heatmap showing the top marker genes for MC38 tumor cell clusters (C23–C29) identified by scRNA-seq (Fig. 2). **(b)** Heatmap displaying inferred genomic copy-number variation (inferCNV) profiles across MC38 tumor cell clusters (C23–C29). Each row represents one tumor cell, and each column represents a genomic region along the indicated chromosomes. **(c, d)** UMAP visualization of representative IFN-γ response genes (c) and proliferation-related genes (d) across tumor cells. Gene expression is shown as $\log_{10}$(TPM + 1). **(e)** Individual tumor growth curves of B16F10 tumors in mice treated with isotype control or anti-IFN-γ in combination with GVAX and αPD-1 therapy. Each line represents one tumor from an individual mouse. n indicates independent mice; exact sample sizes are shown in the figure. NTC, non-targeting control; KO, *Mir25* knockout. **(f)** Flow cytometry quantification of splenic macrophages from the experiment shown in Fig. 4c. Left, total splenic macrophages; right, MHCII^{+} macrophages expressed as a proportion of total macrophages. Data are mean ± SD; each dot represents one mouse. n indicates independent mice; exact sample sizes are shown in the figure. **(g)** Individual tumor growth curves of B16F10 tumors with the indicated genotypes treated with combination therapy (GVAX + αPD-1). Each line represents one tumor from an individual mouse. n indicates independent mice; exact sample sizes are shown in the figure. DKO, *Mir25*/*Ago2* double knockout. **(h)** Flow cytometry quantification of splenic macrophages from the experiment shown in Fig. 4j. Left, total splenic macrophages; right, MHCII^{+} macrophages expressed as a proportion of total macrophages. Data are mean ± SD; each dot represents one mouse. n indicates independent mice; exact sample sizes are shown in the figure. **(i, j)** Volcano plots comparing gene expression in *Mir25* KO B16 cells treated with IFN-γ (n = 2 independent experiments) versus NTC cells (n = 3 independent experiments) under the same conditions (i), and NTC cells treated with IFN-γ (n = 3 independent experiments) versus untreated controls (n = 2 independent experiments) (j). Red dots indicate differentially expressed genes, and bolded gene names indicate TargetScan-predicted miR-25 targets. Statistical significance in (f, h) was assessed using two-sided Student's t test. Differential expression analysis in (i, j) was performed using DESeq2 with a two-sided Wald test and Benjamini–Hochberg correction. Source data are provided in the Source Data file.

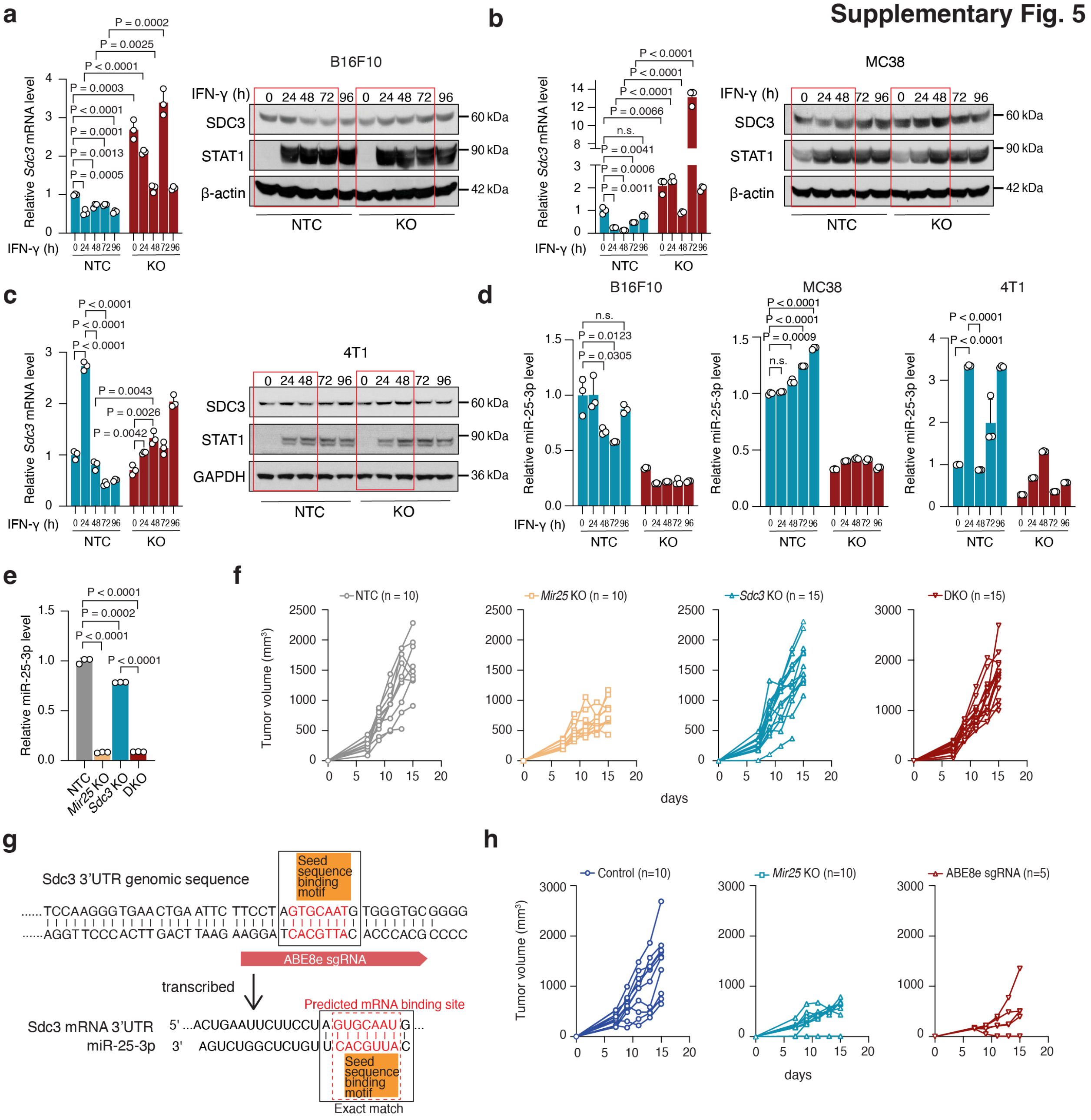

Supplementary Fig. 5: IFN-γ–dependent regulation of SDC3 and miR-25 and functional validation in tumor models

(a–c) RT–qPCR quantification (left) and representative immunoblots (right) of *Sdc3* mRNA and protein expression in B16F10 (a), MC38 (b), and 4T1 (c) cells following IFN-γ treatment at the indicated time points. Representative immunoblots from three independent experiments are shown. β-actin or GAPDH was used as a loading control. Data are mean ± SD from n = 3 independent experiments. NTC, non-targeting control; KO, *Mir25* knockout. **(d)** RT–qPCR

quantification of miR-25-3p expression in B16F10, MC38, and 4T1 cells following IFN-γ stimulation at the indicated time points. Data are mean ± SD from n = 3 independent experiments. **(e)** RT–qPCR quantification of miR-25-3p expression in B16 cells expressing the indicated sgRNAs. Data are mean ± SD from n = 3 independent experiments. DKO, *Mir25*/*Sdc3* double knockout. **(f)** Individual tumor growth curves of B16 tumors expressing the indicated sgRNAs treated with GVAX and anti–PD-1 therapy. Each line represents one tumor from an individual mouse. n indicates independent mice; exact sample sizes are shown in the figure. **(g)** Schematic representation of ABE8e-mediated base editing targeting the *Sdc3* 3′UTR seed-binding motif. **(h)** Individual tumor growth curves of B16 tumors expressing sgRNA constructs under GVAX and anti–PD-1 therapy. Each line represents one tumor from an individual mouse. n indicates independent mice; exact sample sizes are shown in the figure. Statistical significance in (a–e) was assessed using two-sided Student's t test. Source data are provided in the Source Data file.

Supplementary Fig. 6

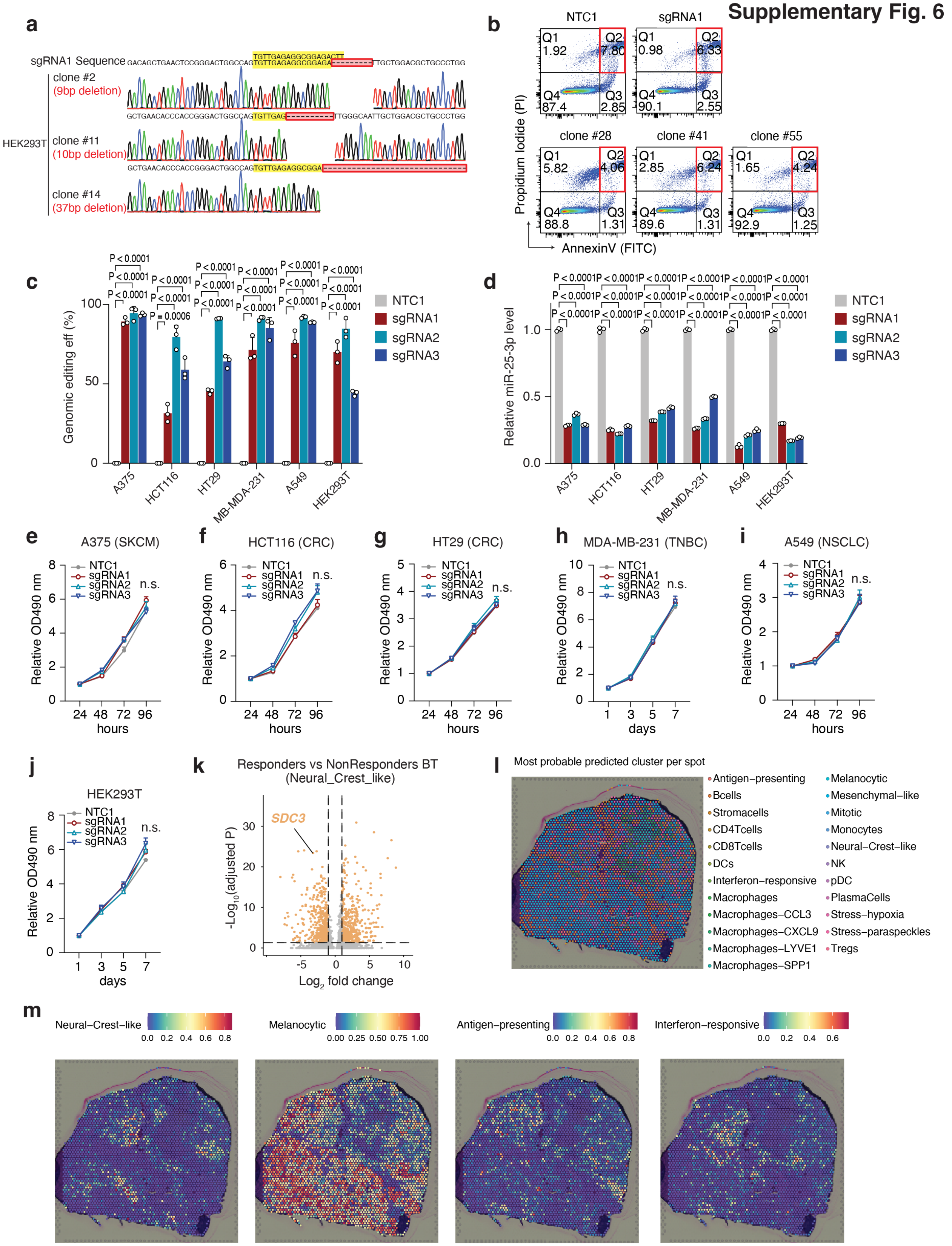

Supplementary Fig. 6: Validation of *MIR25* editing and analysis of human tumor datasets
(a) Sanger sequencing chromatograms showing CRISPR–Cas9 editing at the *MIR25* genomic locus in HEK293T clones. **(b)** Representative flow cytometry analysis of apoptosis in NTC and *MIR25* KO clones. $FITC^+PI^+$ cells indicate late apoptotic cells. Experiments were independently repeated three times with similar results. NTC, non-targeting control. **(c)** TIDE analysis of genome editing efficiency across human cancer cell lines and HEK293T cells. Data are mean ± SD from n = 3 independent experiments. **(d)** RT–qPCR quantification of miR-25-3p expression following transduction with three distinct sgRNAs. Data are mean ± SD from n = 3 independent experiments. **(e–j)** In vitro proliferation assays of NTC and MIR25-edited cells in A375 (e), HCT116 (f), HT29 (g), MDA-MB-231 (h), A549 (i), and HEK293T (j) cells. Data are mean ± SD from n = 6 independent experiments. **(k)** Volcano plot showing differentially expressed genes in neural crest–like cells from responders versus non-responders at baseline (BT), based on data from Pozniak et al. (Cell, 2024). *SDC3* is highlighted. **(l)** Spatial transcriptomic map of a melanoma sample (Visium platform), based on data from Pozniak et al. (Cell, 2024). **(m)** Spatial feature plots showing neural crest–like, melanocytic, antigen-presenting, and interferon-responsive signatures reproduced from the dataset in Pozniak et al. (Cell, 2024). Statistical significance in (c, d) was assessed using two-sided Student's t test. Statistical significance in (e–j) was assessed using two-way ANOVA with Tukey's multiple comparisons test. Differential expression analysis in (k) was performed using a two-sided Wald test with Benjamini–Hochberg correction. Source data are provided in the Source Data file.

Supplementary Fig. 7

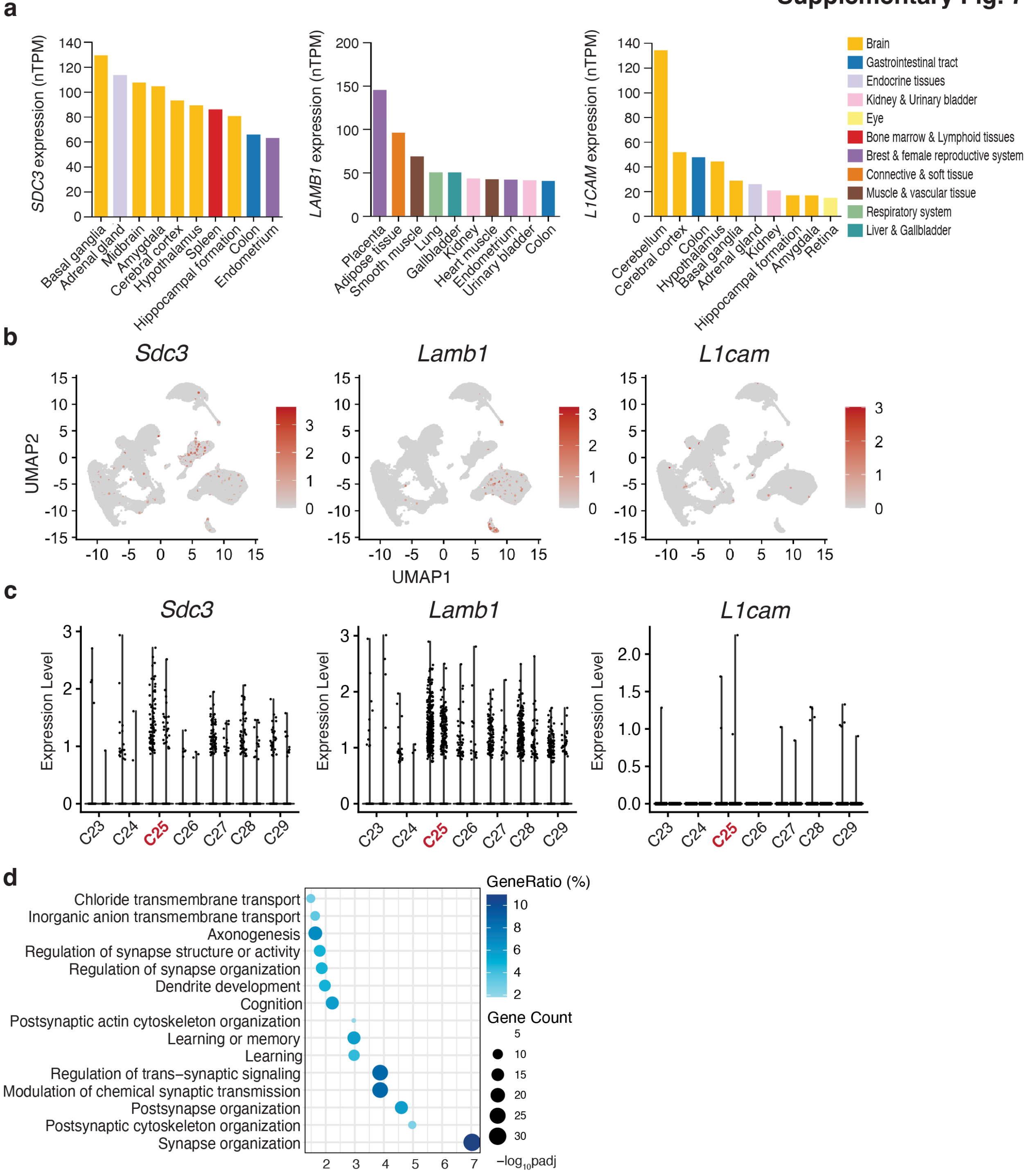

Supplementary Fig. 7: Expression patterns of SDC3-associated genes across tissues and tumor cell populations

(a) Top 10 human tissues with the highest *SDC3* (left), *LAMB1* (middle), and *L1CAM* (right) mRNA expression, based on data from the Human Protein Atlas. Tissues are color-coded by

origin. **(b)** Feature plots showing expression of *Sdc3*, *Lamb1*, and *L1cam* in the scRNA-seq dataset (Fig. 2a). Gene expression is shown as $\log_{10}$(TPM + 1). **(c)** Violin plots showing the expression of *Sdc3*, *Lamb1*, and *L1cam* across tumor cell clusters (C23–C29) in *Mir25* KO and control tumors. Within each cluster, the left violin represents NTC (non-targeting control) and the right represents *Mir25* KO. Each point corresponds to a single cell. Cluster C25 is highlighted. **(d)** Gene Ontology analysis of downregulated pathways in miR-25 low- versus high-expressing TCGA-SKCM-TM tumors. Downregulated pathways are shown in blue. Statistical significance in (c) was performed using DESeq2 with a two-sided Wald test and Benjamini–Hochberg correction and no statistical significance was observed. Differential expression analysis in (d) was performed using DESeq2 with a two-sided Wald test and Benjamini–Hochberg correction. Gene Ontology enrichment analysis was performed using clusterProfiler with Benjamini–Hochberg correction. Source data are provided in the Source Data file.
