# Supplementary material for: microRNA-25 drives immune checkpoint therapy resistance by repressing innate and humoral immunity via Syndecan-3
Source: Nat Commun. 2026 May 20;17:6666. doi: 10.1038/s41467-026-73339-y (PMC13381856; doi:10.1038/s41467-026-73339-y)
Supplement: Supplementary file 2 — Description of Additional Supplementary Files [file 41467_2026_73339_MOESM2_ESM.pdf]

Description of Additional Supplementary Files

Supplementary Dataset 1: Small RNA-seq differential expression analysis of tumors under different treatment conditions (n = 3 tumors per group).

Supplementary Dataset 2: hsa-miR-25-3p expression values in TCGA-SKCM-TM patient samples (n = 352 patients).

Supplementary Dataset 3: Sequences of sgRNAs and PCR primers used in this study.

Supplementary Dataset 4: Validation data for single-clone Mir25/MIR25 knockout cells.

Supplementary Dataset 5: Top marker genes defining each cell cluster from scRNA-seq analysis.

Supplementary Dataset 6: Differentially expressed genes in each scRNA-seq cluster (cells derived from independent tumors; NTC n = 4 tumors, KO n = 3 tumors).

Supplementary Dataset 7: Gene Ontology enrichment analysis results for scRNA-seq clusters.

Supplementary Dataset 8: Cell–cell communication analysis using CellChat.

Supplementary Dataset 9: Bulk RNA-seq differential expression and Gene Ontology analysis of B16 tumors and TCGA samples.

Supplementary Dataset 10: Normalized gene expression values of TCGA-SKCM-TM samples stratified by miR-25 expression.

Supplementary Dataset 11: In vitro RNA-seq analysis of B16 cells under indicated conditions.

Supplementary Dataset 12: Humanized gene signature matrix derived from MC38 scRNA-seq data.

Supplementary Dataset 13: Single-cell RNA-seq analysis of malignant cells from Pozniak et al. (Cell, 2024).
