# Supplementary material for: microRNA-25 drives immune checkpoint therapy resistance by repressing innate and humoral immunity via Syndecan-3
Source: Nat Commun. 2026 May 20;17:6666. doi: 10.1038/s41467-026-73339-y (PMC13381856; doi:10.1038/s41467-026-73339-y)
Supplement: Supplementary file 16 — Reporting Summary [file 41467_2026_73339_MOESM16_ESM.pdf]

nature portfolio

Corresponding author(s): Tariq M. Rana

Last updated by author(s): Mar 27, 2026

Reporting Summary

Nature Portfolio wishes to improve the reproducibility of the work that we publish. This form provides structure for consistency and transparency in reporting. For further information on Nature Portfolio policies, see our Editorial Policies and the Editorial Policy Checklist.

Please do not complete any field with "not applicable" or n/a. Refer to the help text for what text to use if an item is not relevant to your study.
For final submission: please carefully check your responses for accuracy; you will not be able to make changes later.

Statistics

For all statistical analyses, confirm that the following items are present in the figure legend, table legend, main text, or Methods section.

n/a	Confirmed	
☐	☒	The exact sample size (*n*) for each experimental group/condition, given as a discrete number and unit of measurement
☐	☒	A statement on whether measurements were taken from distinct samples or whether the same sample was measured repeatedly
☐	☒	The statistical test(s) used AND whether they are one- or two-sided<br>*Only common tests should be described solely by name; describe more complex techniques in the Methods section.*
☒	☐	A description of all covariates tested
☐	☒	A description of any assumptions or corrections, such as tests of normality and adjustment for multiple comparisons
☐	☒	A full description of the statistical parameters including central tendency (e.g. means) or other basic estimates (e.g. regression coefficient) AND variation (e.g. standard deviation) or associated estimates of uncertainty (e.g. confidence intervals)
☐	☒	For null hypothesis testing, the test statistic (e.g. *F*, *t*, *r*) with confidence intervals, effect sizes, degrees of freedom and *P* value noted<br>*Give P values as exact values whenever suitable.*
☒	☐	For Bayesian analysis, information on the choice of priors and Markov chain Monte Carlo settings
☒	☐	For hierarchical and complex designs, identification of the appropriate level for tests and full reporting of outcomes
☒	☐	Estimates of effect sizes (e.g. Cohen's *d*, Pearson's *r*), indicating how they were calculated

Our web collection on statistics for biologists contains articles on many of the points above.

Software and code

Policy information about availability of computer code

Data collection	Data collection for flow cytometry was performed using FlowJo (v10.8.1), and IHC images were acquired with ImageViewerG (v1.1.7). Absorbance measurements were obtained using a Synergy 2 BioTek plate reader. Quantitative real-time PCR (qRT-PCR) was conducted using iTaq Universal SYBR Green Supermix (Bio-Rad) on Roche LightCycler 480 II or QuantStudio7 Pro system. Computational analyses were conducted in R (v4.3.2) using RStudio (v2023.12.1+402), incorporating Seurat (v5.0.1) for single-cell RNA-seq analysis, inferCNV (v1.14.0) for copy number inference, CellChat (v1.6.1) for cell–cell communication, clusterProfiler (v4.10.0) for enrichment analysis, TCGAbiolinks (v2.30.3) for TCGA data mining, survminer (v0.4.9) for survival analysis, and limma (v3.58.1) for bulk transcriptomic comparisons. Bulk RNA-seq preprocessing employed FastQC (v0.12.1) for quality control, Trimmomatic (v0.40) for adapter trimming, HISAT2 (v2.2.1) for alignment, and DESeq2 (v1.42.0) for differential expression analysis. All analyses were performed using publicly available software packages with standard or previously published workflows.
Data analysis	Data analysis was performed using GraphPad Prism (v9) and R (v4.3.2).

For manuscripts utilizing custom algorithms or software that are central to the research but not yet described in published literature, software must be made available to editors and reviewers. We strongly encourage code deposition in a community repository (e.g. GitHub). See the Nature Portfolio guidelines for submitting code & software for further information.

Data

Policy information about availability of data

All manuscripts must include a data availability statement. This statement should provide the following information, where applicable:

- Accession codes, unique identifiers, or web links for publicly available datasets
- A description of any restrictions on data availability
- For clinical datasets or third party data, please ensure that the statement adheres to our policy

RNA-seq and scRNA-seq data generated in this study have been deposited in the Gene Expression Omnibus (GEO) under accession code GSE313080 [https://www.ncbi.nlm.nih.gov/geo/query/acc.cgi?acc=GSE313080]. Published single-cell and spatial transcriptomic datasets from Pozniak et al. (Cell, 2024) are available from the corresponding publications. Source data are provided with this paper. All other data supporting the findings of this study are included within the article, Supplementary Information, or Source Data files.

Research involving human participants, their data, or biological material

Policy information about studies with human participants or human data. See also policy information about sex, gender (identity/presentation), and sexual orientation and race, ethnicity and racism.

Reporting on sex and gender	Sex or gender was not considered in the study design.
Reporting on race, ethnicity, or other socially relevant groupings	Not applicable.
Population characteristics	Not applicable.
Recruitment	Not applicable.
Ethics oversight	Not applicable.

Note that full information on the approval of the study protocol must also be provided in the manuscript.

Field-specific reporting

Please select the one below that is the best fit for your research. If you are not sure, read the appropriate sections before making your selection.

☒ Life sciences ☐ Behavioural & social sciences ☐ Ecological, evolutionary & environmental sciences

For a reference copy of the document with all sections, see nature.com/documents/nr-reporting-summary-flat.pdf

Life sciences study design

All studies must disclose on these points even when the disclosure is negative.

Sample size	No formal statistical methods were used to predetermine sample size. Sample sizes for in vivo experiments were chosen based on standard practices in the field and prior studies to ensure sufficient statistical power and reproducibility. In vitro experiments were performed with at least three independent biological replicates. Sequencing-based experiments were conducted using sample sizes consistent with commonly accepted standards for RNA-seq and single-cell RNA-seq analyses. Exact sample sizes for all experiments are provided in the corresponding figure legends.
Data exclusions	No data was excluded.
Replication	All key experiments in this study were independently repeated at least three times with consistent results, including RT–qPCR, western blotting, flow cytometry, immunohistochemistry, RNA immunoprecipitation, and in vitro functional assays. In vivo tumor growth and survival experiments were performed using independent cohorts of mice with reproducible outcomes across experiments. No findings reported in this study failed to be replicated.
Randomization	Samples and animals were randomly assigned to experimental groups where applicable. For in vivo experiments, mice were randomly allocated to treatment groups to minimize bias. For in vitro experiments, cells were assigned to experimental conditions in parallel to ensure comparable starting conditions. No covariates requiring adjustment were identified for this study.
Blinding	Investigators were not blinded to group allocation during treatment administration. However, data collection and analysis were performed in a blinded manner where feasible.

Reporting for specific materials, systems and methods

We require information from authors about some types of materials, experimental systems and methods used in many studies. Here, indicate whether each material, system or method listed is relevant to your study. If you are not sure if a list item applies to your research, read the appropriate section before selecting a response.

Materials & experimental systems

n/a	Involved in the study	
☐	☒	Antibodies
☐	☒	Eukaryotic cell lines
☒	☐	Palaeontology and archaeology
☐	☒	Animals and other organisms
☒	☐	Clinical data
☒	☐	Dual use research of concern
☒	☐	Plants

Methods

n/a	Involved in the study	
☒	☐	ChIP-seq
☐	☒	Flow cytometry
☒	☐	MRI-based neuroimaging

Antibodies

Antibodies used	Flow cytometry antibodies were used as follows: CD45 (BioLegend, 103128), CD11b (Biolegend, 101216), Ly6G (Biolegend, 128016) , Ly6C (BioLegend, 128036), MHC-II (BioLegend, 107626), F4/80 (BioLegend, 123132), CD206(BioLegend, 141704), CD86 (BioLegend, 105008), and FITC Annexin V Apoptosis Detection Kit with PI (BioLegend, 640914).<br><br>Western blot antibodies were used as follows: SDC3 (10886-1-AP, Proteintech), AGO2 (ab186733, Abcam), STAT1 (9172, Cell Signaling Technology), β-Actin (4967, Cell Signaling Technology), and GAPDH (14C10, Cell Signaling Technology).<br><br>IHC antibodies were used as follows: C3 (PA5-21349, Thermo)
Validation	Antibody specificity was validated by the manufacturers, and where applicable, by expected band size in immunoblotting and appropriate staining patterns in flow cytometry and immunohistochemistry.

Eukaryotic cell lines

Policy information about cell lines and Sex and Gender in Research

Cell line source(s)	B16, 4T1, A375, HCT116, HT29, MDA-MB-231, A549, and HEK293T cell lines were obtained from the American Type Culture Collection (ATCC). MC38 cells were obtained from Kerafast. The B16-GM-CSF cell line was generously provided by Drs. Glenn Dranoff and Michael Dougan (Dana-Farber/Harvard Cancer Center).
Authentication	Cell lines obtained from commercial sources (e.g., ATCC) were authenticated by the providers using standard methods such as short tandem repeat (STR) profiling. Cell lines obtained from collaborators were authenticated by the providers as stated. No additional in-house authentication was performed.
Mycoplasma contamination	All cell lines tested negative for mycoplasma contamination.
Commonly misidentified lines (See ICLAC register)	No commonly misidentified cell lines were used.

Animals and other research organisms

Policy information about studies involving animals; ARRIVE guidelines recommended for reporting animal research, and Sex and Gender in Research

Laboratory animals	Mice were housed under standard institutional conditions with a 12 h light/12 h dark cycle, controlled ambient temperature (20–24° C), and relative humidity (40–60%). C57BL/6J mice were used for all experiments unless otherwise specified. BALB/c mice were used specifically for the experiments shown in Fig. 1m and 1p. All mice were obtained from established vendors as indicated in the Methods, and information on substrain, genetic background, species (mouse), sex (female), and age (9–12 weeks) has been clearly stated.
Wild animals	Not applicable.
Reporting on sex	Female mice were used in all experiments to minimize variability associated with sex-dependent immune responses.
Field-collected samples	Not applicable.
Ethics oversight	All animal studies were approved by the Institutional Animal Care and Use Committee (IACUC) of the University of California, San Diego (protocol S14123). Female C57BL/6J and BALB/c mice aged 9–12 weeks were used in this study.

Note that full information on the approval of the study protocol must also be provided in the manuscript.

Plants

Seed stocks	Not applicable.
Novel plant genotypes	Not applicable.
Authentication	Not applicable.

Flow Cytometry

Plots

Confirm that:

☒ The axis labels state the marker and fluorochrome used (e.g. CD4-FITC).

☒ The axis scales are clearly visible. Include numbers along axes only for bottom left plot of group (a 'group' is an analysis of identical markers).

☒ All plots are contour plots with outliers or pseudocolor plots.

☒ A numerical value for number of cells or percentage (with statistics) is provided.

Methodology

Sample preparation	Tumors were mechanically dissociated and enzymatically digested using the Mouse Tumor Dissociation Kit (Miltenyi Biotec) following the manufacturer's instructions. Spleens were collected, minced with a razor blade, and passed through a 70 µm cell strainer. Cells were washed in culture medium containing 10% fetal bovine serum (FBS; Gibco) and treated with ammonium chloride solution (STEMCELL) to lyse red blood cells (RBCs). Remaining cells were incubated with TruStain FcX anti-mouse CD16/32 antibodies (BioLegend) to block Fc receptors and stained with Zombie Aqua Live/Dead fixable dye (BioLegend) to exclude dead cells. Subsequently, cells were incubated in the dark on ice with antibody cocktails recognizing surface markers. After staining, cells were resuspended in FACS staining buffer and analyzed on a BD FACS Canto flow cytometer (SD CFAR Flow Cytometry Core). BD Compensation Beads (552845, BD Biosciences) were used to set fluorescence parameters. Fluorescence-minus-one (FMO) controls, unstained cells, and single-stained samples were included to establish gating strategies.
Instrument	BD FACS Canto (UCSD Flow Cytometry core)
Software	Data collection for flow cytometry experiments was performed with FlowJo (10.8.1).
Cell population abundance	A minimum of 20,000 live CD45+ cells were acquired per sample for immune profiling. For apoptosis assays, 10,000 cells were acquired per sample.
Gating strategy	Gating strategies for all experiments are provided in Supplementary Fig. 3 . Initial gating was performed based on forward and side scatter (FSC/SSC) to exclude debris, followed by doublet exclusion and viability gating.

☒ Tick this box to confirm that a figure exemplifying the gating strategy is provided in the Supplementary Information.

This checklist template is licensed under a Creative Commons Attribution 4.0 International License, which permits use, sharing, adaptation, distribution and reproduction in any medium or format, as long as you give appropriate credit to the original author(s) and the source, provide a link to the Creative Commons license, and indicate if changes were made. The images or other third party material in this article are included in the article's Creative Commons license, unless indicated otherwise in a credit line to the material. If material is not included in the article's Creative Commons license and your intended use is not permitted by statutory regulation or exceeds the permitted use, you will need to obtain permission directly from the copyright holder. To view a copy of this license, visit http://creativecommons.org/licenses/by/4.0/
