# Supplementary material for: microRNA-25 drives immune checkpoint therapy resistance by repressing innate and humoral immunity via Syndecan-3
Source: Nat Commun. 2026 May 20;17:6666. doi: 10.1038/s41467-026-73339-y (PMC13381856; doi:10.1038/s41467-026-73339-y)
Supplement: Supplementary file 17 — Transparent Peer Review file [file 41467_2026_73339_MOESM17_ESM.pdf]

nature portfolio

Peer Review File

microRNA-25 drives immune checkpoint therapy resistance by repressing innate and humoral immunity via Syndecan-3

Corresponding Author: Professor Tariq Rana

This file contains all reviewer reports in order by version, followed by all author rebuttals in order by version.

Version 0:

Reviewer comments:

Reviewer #1

(Remarks to the Author)
Please, see full review in the attached pdf file.

Reviewer #2

(Remarks to the Author)
In this manuscript, Zhu, et al. attempted to tackle the issue of therapeutic resistance to anti-PD-1 in various forms of cancer (melanoma, colorectal cancer, and triple negative breast cancer). They found that downregulation of miR-25 in tumor cells upon IFN-g treatment in vitro and anti-PD-1 therapy in vivo helped tumor cell escape from immunotherapy. Deletion of miR-25 by CRISPR-Cas9 in tumor cells improved therapeutic efficacy of anti-PD-1 by promoting innate immune sensing through augmented expression of Sdc3, a membrane proteoglycan that can be recognized by innate immunosurveillance. The authors attributed this to the reduction of tumor-associated macrophages (TAMs, even though they gained proinflammatory M1 features upon miR-25 KO and GVAX+anti-PD-1 treatment) and increased Nlrp3 inflammasome formation, the latter of which depended on TAMs, IFN-g release, and miRISC protein Ago2. Overall, this study illustrates that tumor cells turn off full-spectrum immune activation by downregulating miR-25 (and other microRNAs) after sensing IFN-g production upon anti-PD-1 therapy. Some major and minor concerns are as follows:

Major concerns:
1. While miR-25 was downregulated in tumor cells upon anti-PD-1 and IFN-g treatment, restoration/stabilization of miR-25 (but not deletion of miR-25) in tumor cells would be needed to unequivocally link miR-25 downregulation to anti-PD-1 resistance.
2. With the identification of miR-25 as an underlying mechanism of therapeutic resistance to anti-PD-1, how to target it to bolster therapeutic efficacy of anti-PD-1 was not described.
3. Was miR-25 different from other widely studied microRNAs, such as miR-19-92a cluster and miR-155, in terms of their roles in immune evasion?
4. While miR-25 deletion in tumor cells greatly increased the infiltration of CD45+ cells, the percentages of macrophages were actually decreased. This significant reduction of macrophages in miR-25 KO tumor cells (further reduced upon GVAX+anti-PD-1: GP) casted the benefit of the doubt on the role of macrophages in the process. Specifically, how to reconcile this macrophage reduction to its involvements in driving Nlrp3 inflammasome formation and innate immunity? It was counterintuitive that reduction of TAM (M1) drove miR-25 deficiency effects (greater response to anti-PD-1, etc.). Furthermore, while deletion of TAM with clodronate liposomes largely abolished miR-25 KO effects, this deletion was not overt in miR-25 KO tumors. Did deletion of macrophages abolish inflammasome formation and potentiation of adaptive immunity in the TME of miR-25 KO tumors?
5. There was a repression of interferon genes in macrophages from miR-25 KO tumors by αPD-1 therapy. Conversely, blocking IFN-g activated Mac in miR-25 KO tumors. These seemingly contradictory observations needed to be reconciled.
6. Ago2 was unchanged in miR-25 and miR17-92a deficient tumor cells, although the authors showed that Ago2/miR-25 DKO phenotypes were comparable to those in Ago2 KO alone (Figure 4c and s4d). This is sort of expected as without the essential component of RISC complex, miRNAs would not work. That said, Ago KO only partially abolished the phenotype of miR-25 KO, suggesting other mechanisms exist.
7. Bulk RNA-Seq and scRNA-seq should be done using the B16-F16 (the major tumor model); otherwise, it would be hard to connect, given that different treatments were used for B16-F10 (GVAX+anti-PD-1) and MC38 (only anti-PD-1). By the same

token, Fig. 4d RNA seq analysis should use in vivo RNA-seq datasets, as mixing in vivo RNA-seq data with in vitro RNA-seq data from miR-25 KO tumor cells treated with IFN-g or not introduced too many confounding factors.

Minor concerns:
1. Please clearly specify GVAX.
2. Line 449-450—"Our data indicates that anti-PD-1 can increase TAM infiltration of tumors, an activity regulated by tumor cells to achieve immunosenescence." But there were no data supporting this; instead, they showed that anti-PD-1 decreased TAM infiltration.
3. Please clarify "we found that IFN-γ depletion starting at day 8 also abolished miR-25-KO effects and increased the number of MHCII+ macrophages in spleen, indicating that IFN-γ is indispensable for TAM activation in the TME" (Line 456-458), as this writing suggested that IFN-g actually suppressed TAM activation.
4. Please explain "in the presence of IFN-γ, TAM recognition of tumor cell changes depending on miR-25 expression in tumor cells" (Line 461). Did the authors mean to say that TAM recognition of tumor cells needed miR-25 expression?
5. This is an overstatement "we show that miR-25-KO induces Sdc3 expression on tumor cells, converting immune 'cold' to 'hot' tumors based on Nlrp3 inflammasome formation in seen TAMs after ICB (Figure 6)." "in seen" appeared to be a typo.
6. Please double check the text to remove typos and grammatical errors.

Reviewer #3

(Remarks to the Author)
In this study, Zhu et al show that microRNAs miR-25 and miR-17-92 members modulate innate immunity and response to anti-PD1 immunotherapy. Using B16F10 as a murine model of melanoma, the authors find miR-25 and the miR-19/72 cluster downregulated in tumor cells upon GVAX and anti-PD1 treatment. CRISPR/Cas9 knockout of miR-25 combined with GVAX+PD1 or PD1 treatment in B16F10 melanoma, MC38 colorectal carcinoma, and 4T1 breast cancer significantly reduced tumor volume and enhanced survival relative to GVAX+PD1 or PD1 immunotherapy, respectively. miR-25 knockout alone did not alter tumor growth in vivo, supporting its specificity in regulating immunotherapy response. Similar effects were found by targeting components of miR-17-92. These results highlight the potential role suppressing miRNA can have in enhancing immunotherapy treatments.
Flow cytometry plus immunohistochemistry of B16F10 tumors demonstrated increased infiltration of CD45+ immune cells in the miR-25 KO GP group, an increase of cytotoxic Granzyme B CD8+ T cells, a reduction of immune-suppressive FOXP3+ CD4+ Tregs and CD206+ macrophages. Bulk RNA-sequencing and immunohistochemistry of miR-25 KO GP and miR-106a-KO tumors revealed upregulation of complement genes like C3 and innate immune pathways involving the pyroptotic phagosome, ultimately contributing to a pro-inflammatory microenvironment in the miR-25 KO GP group. Clodronate liposome injections abolished the reduced tumor volume observed in the miR-25 KO GP group, suggesting macrophages mediate miR-25's immunotherapeutic effects.
Single cell RNA-seq of MC38 cells showed that upon miR-25 inhibition, macrophage depletion plus polarization, CAF polarization, and T cell activation contributes to the immunotherapy response. Depletion of IFN-y abolished the reduced tumor growth observed upon miR-25 KO GP treatment in B16F10 cells and increased the number of MHC-II expressing M2 macrophages in the spleen, indicating M1 macrophage polarization following miR-25 KO requires IFN-y in the TME. Overlapping three B16F10 gene expression datasets with an algorithm for predicting miR-25 target genes uncovered Sdc3 as a downstream target of miR-25. miR-25 base-pairs with Sdc3, repressing Sdc3 expression through an interaction with Ago2 for RISC-dependent degradation in the presence of IFN-y. Additionally, miR-25 knockout increases transcript and protein levels of Sdc3 in B16F10, MC38, and 4T1 cells. This suggests that while IFN-y enhances degradation of Sdc3, higher basal mRNA and protein levels of Sdc3 due to miR-25 knockout enables cancer cells to maintain higher overall Sdc3 levels.
Small RNA sequencing of TCGA metastatic melanoma patients showed a ~2-fold difference in miR-25 levels, with miR-25 low tumors exhibiting increased phagosome pathway activity and complement plus IL6-JAK-STAT signaling. miR-25-low patients had reduced amounts of undifferentiated macrophages and higher delta gamma T cells, CD8 T cells, and CD4 memory activated T cells. mir-25 and miR-17/92 expression negatively correlated with innate immune genes related to complement activity. Furthermore, responders to PD1 immunotherapy showed lower levels of miR-25 and negatively correlated with complement gene expression.

Major issues
It is unclear how differentially expressed miRNAs in B16 tumors treated with GVAX and anti-PD1 vs control were prioritized for further analysis and why specifically they followed up on miR-25. Based on the volcano plot, these miRNA don't seem the most differentially expressed.

There are notable differences between the FACS and IF analyses of tumors, with the FACS showing reduced macrophage infiltration in the miR-25 KO mice and the IF showing similar numbers. FACS and IF analyses were only conducted in the B16 model. They should be repeated in at least another model. Similarly, there seem to be differences with the MC38 model, which by scRNAseq did show a reduction of M2 macrophages and an increased in gamma-delta T cells. Given the differences observed across models and techniques, it seems necessary to compare side-by-side the results by FACS and IF in B16 and MC38 cells.
Finally, the mechanistic findings (miR-25 modulation of Sdc3) appear disconnected from the phenotypic effects on complement, IFN levels, macrophage polarization, etc. Authors should do a better job at linking mechanism and immune effects by showing that Sdc3 modulation by miR-25 mediates (at least partially) those effects.

In sum, while the manuscript contains many interesting findings, some of the conclusions don't seem sufficiently supported

by data obtained in more than one model. The phenotypic effects of miR-25 KO in the tumor immune repertoire seem context/model dependent and it is hard to draw strong conclusions. In addition, the mechanistic connection between miR-25 and a single target (Sdc3) seems also a stretch, and should be further supported by clinical data.

Specific points that should be addressed:
Figure 1
1. Despite its spontaneous occurrence, B16F10 cells do not harbor clinically relevant melanoma mutations in the MAPK pathway or tumor suppressor genes. More relevant melanoma immunotherapy models are currently available and should be used to confirm the ability of miR-25 KO to enhance response to immune-therapy.
2. Despite some clinical indications that GVAX treatment could positively modulate immunotherapy, the apparent differences in PD1 response as observed in B16F10 are clinically rare, indicating GVAX may not be a valid model for making broad claims regarding immunotherapy.
3. In their small RNA sequencing analyses, the authors compared the GP (GVAX+PD1) group to an untreated control instead of the GVAX group. It is unclear if miR-25 and miR-19/72 cluster members are significantly downregulated in comparison to the GVAX group, therefore the authors can only make claims in regard to immunotherapy in the context of GVAX treatment and not solely immunotherapy, which is far less clinically relevant due to the clinically unproven benefit of GVAX.
a. miR-19 is the only member of its miRNA cluster whose fold change is halved while also achieving a significant p-value.
b. It is unclear whether the authors profiled any other hits that had a more meaningful fold change and significant p-value, to validate their screen.
4. Colorectal carcinoma tumors are typically responsive to immunotherapy if they are mismatch repair–deficient (dMMR) or possess microsatellite instability-high (MSI-H). MC38 cells are proficient in mismatch repair but respond to immunotherapy, suggesting they are poor models that do not accurately recapitulate colorectal carcinoma as seen in patients.
5. Injections of MC38 and 4T1 were not orthotopic but injected into the flank of mice. Figures 1f-h are missing error bars for the red lines. Tumor weight at endpoint should be shown to orthogonally validate a difference in tumor size between groups.
6. The authors demonstrated significant reduction of miR-25 level with each of three sgRNAs used individually, but chose to combine all three guides in vivo, increasing the likelihood of off-target effects. The sequence of the non-targeting guide RNA (sgNTC) used is unknown.
7. It is unclear if deletion of miR-17-92a family members in B16F10 altered proliferation of tumor cells, as that could explain the reduced tumor volume. Proliferation effects should be shown.
Figure 2
8. The voltage settings and gating strategy in S2a and S2b are cutoff and thus may not accurately capture all CD45+ cells. Additionally, the Granzyme B, FOXP3, and CD69 gating is lenient, defining positive cells with a signal strength barely above negative.
9. RNA-sequencing was performed on unsorted tumors, therefore it is unclear from which cell population the changes in innate immune signaling are coming and whether it is a feature of multiple cells and cell types or driven by a single cell population exhibiting much higher levels of immune genes/pathways. It is also unclear if these upregulated immune pathways are observed solely due to the presence of more CD45+ cells in the tumor. Some of the gene expression differences in Fig. 2g appear to be inconsistently high in one of the three miR-25 KO GP samples (Cfp, Tlr9, C1rl, Cfd, C3, Serping1, C1s1, Clu, Cfh).
10. F4/80 can also be a marker of monocytes and eosinophils. It is unclear why authors suggest C3+ cells do not interact with tumor cells.
11. There does not appear to be a significant difference in tumor mass between the miR-25 KO GP and miR-25 KO GP+LC groups, contrary to the obvious difference in tumor volume. This suggests that the tumor volume measurement does not accurately represent the differences in tumor volume, possibly due to the oblong shape of the tumors and a failure to integrate tumor height in the formula used to calculate tumor volume. This casts doubt on previous experiments that do not quantify tumor mass. This is further corroborated by the black and blue lines, where volume suggests a ~1.5x difference between tumor volume while tumor mass is nearly identical.
12. Despite efficient reduction of splenic and tumoral macrophages by LC treatment, the lack of difference in tumor mass in the yellow and red groups casts doubt whether miR-25 KO GP treatment works through macrophage infiltration. Additionally, without sequencing of the miR-25 KO GP tumors, suggesting that complement signaling has changed is slightly overreaching.
Figure 3
13. While they may be simply validating their results in another model when using MC38 for scRNA-seq, it should be explicitly stated if they attempted scRNA-seq on B16F10 and what was the outcome, if any, was. There are multiple published gene signatures that can be utilized to define M1 vs M2 macrophages. Categorizing a macrophage population as M1 because of a unique metabolic signature, and simply defining the rest as M2 is not appropriate.
14. Additionally, authors failed to recapitulate a difference in CD8 and CD4 cells in MC38 tumors as in B16F10, and did not mention if they observed a difference in delta gamma T cells in B16F10 or simply did not check. Decrease in dendritic cells was unaddressed. The difference in delta gamma T cells, while significant, is not particularly meaningful. Furthermore, the difference in MC38 cells by scRNA-seq is also marginally significant, suggesting that the reduced MC38 tumor volume in Figure 1 may be simply due to a lack of macrophages, while the number of tumor cells is relatively unchanged and the tumor remains cold. It is unclear if the differences observed across models are a result of using different approaches (FACS, IF and bulk RNAseq in B16 vs scRNA-seq in MC38); therefore using the same techniques and comparing models side by side seems necessary to extract solid phenotypic conclusions.
15. Decrease in macrophage interferon signaling during immunotherapy response inconsistent with what’s known in the literature and claims in Figure 4. Interferon gamma levels were unaddressed. Additionally, the complement signaling in the MC38 appears to come from fibroblasts instead of macrophages like in the B16F10.

16. Defining Atp6v0c and Tubb4b as regulators of BCR signaling seems overreaching.
Figure 4
17. Unclear if the activated splenic macrophages upon IFN-y depletion are also present in the tumor. Evidence for differential IFN-y signaling was not observed by scRNA-seq in the previous figure.
18. Unclear if the reduction in tumor growth upon Ago2 depletion can be simply explained by reduced proliferation of Ago2-depleted tumor cells. Proliferation effects should be shown. Ago2 sgRNA sequence and thus their specificity is unknown.
19. The authors did not mention whether Sdc3 expression was increased in in vivo MC38 miR-25 KO cells by scRNA-seq, this is important to validate. Besides miR-92, it does not appear other miRNAs of the cluster have complementarity to Sdc3 3'UTR and miR-363 does not appear to be a hit from the original screen in Figure 1. Luciferase reporter assays should be conducted using the entire Sdc3 3'UTR sequence and not just the putative binding site.
20. Ago2 levels should be profiled by qPCR and western blot in their 96hr timecourse to confirm that Ago2 levels do not proportionally increase alongside Sdc3 upon miR-25 KO, which would partially invalidate their findings. They appear consistent in the immunoprecipitation, but without a loading control.
Figure 5
21. It appears authors saw no differences by CIBERSORT in M1 and M2 macrophages like their murine models but instead chose to focus on M0 macrophages. The quantity of delta gamma T cells is very low and marginally different between low and high groups. miR-25 showed very weak negative correlation with complement genes.
22. There are very few responders in the immunotherapy dataset. Many of the nonresponders also had low miR-25 levels. Negative correlation with complement genes is not very convincing as some of the lowest expressing miR-25 patients had very low complement activity' the negative association is only clearly evident in one patient. miR-17HG appears positively correlated with miR-25, unlike in panel f.
23. It is still unclear where IFN-y is coming from in the in vivo murine models and whether it negatively correlates with miR-25 in patients. They should also examine if Sdc3 mRNA levels inversely correlate with miR-25 in patient samples to further confirm the relevance of the proposed model to human.

Reviewer #4

(Remarks to the Author)
• In this study, the authors analyzed the clinical and biological significance of the expression of miRNA-25 and its role in the response of anti-PD1 therapy in Melanoma cancer. To this end, they employed a mouse model of Melanoma. The authors validated some results in a mouse model of colon cancer and triple-negative breast cancer. The authors conclude that miR-25 and some family members of miR-17/92 were downregulated in melanoma tumors. The deletion of miRNA-25 impacts the response to anti-PD1 treatment by making tumors more sensitive to therapy. They also concluded that this was due to an induction of the innate immune response and the upregulation of the heparan sulfate proteoglycan Syndecan-3 and the miRNA complex in the presence of IFN-γ. Finally, through bioinformatics analysis using public databases, the authors observed that miRNA-25 expression impacts the survival of melanoma patients. Although this study is of clinical and biological relevance in the treatment of Melanoma, I consider some concerns must be addressed before considering the manuscript for publication.
Comments:
Major
- Figure 1c, why did the authors decide to make the comparison only for GVAX + αPD-1 (GP) vs Control? the fact that the other conditions do not significantly change tumor volume does not mean that there is no change in miRNA expression in the other conditions. It would be informative to know whether the changes in miRNA expression are really due to an additive effect of the two treatments or come from the effect of the αPD-1 treatment, or the effect of the factors generated by the GVAX inoculation. Please make comparisons for each group.
- Figure 2: Flow cytometry Gatings need to be improved (Figure S2). 1) Some populations are too far to the right and appear to fall out of the quadrant. 2) According to the methodology, the cells were incubated with Zombi Aqua to distinguish between live and dead cells. Please show the Gatings where it is shown that the analysis is being done on the live cells (Zombie Aqua-negative) as the they showed in Figure S2-C. Why did the authors decide to use CD24 (which is preferential for LT-B) as a marker for dendritic cells and not CD11c? 3) Probably the authors did not choose well an example of FOXP3+ cells because in the analysis they showed, there are no cells in that quadrant. The same for granzyme B. Overall, flow cytometry data is a fundamental part of the work and the analysis should be very strict.
- The CD206+ macrophages come from the F4/80 population, did the M1 population increase? Please, analyze M1 markers also to ensure that the M2 population of CD206+ macrophages is increasing.
- Figure 2 k and figure s2k-l, please corroborate the date by immunohistochemistry like in figure 2h.
- Figure 3: The authors mention that to know the composition of the microenvironment of the miR-35 KO GP tumors they did the colon cancer single-cell RNA sequencing (scRNA-Seq) model because it is more immunogenic. From Figures 1 to 5 except for Figure 3, all or most of it has been done in the melanoma model, even the validation of their model in the clinical part was done in melanoma patients. Regardless that the colon cancer model is more immunogenic, the authors should do the scRNA-Seq in the B16 melanoma model, to corroborate which immune populations are present in their model and are being regulated by both the miRNA and the anti-PD1 treatment.
- Figure 4, That's a lot of ideas in one figure that are not so conclusive. For example: Figure 4a: To test the hypothesis that miRNA-25 can regulate PAMP or DAMP expression in tumor cells by the release of IFN-γ due to anti-PD1 treatment, the authors depleted IFN-γ in their miR-25 KO GP mouse model. 1) I suggest first measuring blood IFN- γ concentration and protein levels by flow cytometry in miR-25 KO GP+anti-PD1 models and respective controls. 2) Inhibit IFN- γ and corroborate that it is inhibited by the above methods. My suggestion of points 1 and 2 is because many variables are being applied to the

model. Figure 4b: measure also by cytometry if the proportion of resident macrophages changes and the other populations that are severely affected by the effect of IFN- inhibition. Figure 4c: page 12, line 329 double KO of Ago2/miR-25 strongly suggests that miR-25 regulation of immunotherapy requires miRISC. This is not necessarily true because Ago2 KO alone decreases tumor size and the effect of double KO could be because other miRNAs may be being modified. Furthermore, no information is presented on how the immune system could be modified. In this case, it would be worthwhile to do an RNA-seq of these tumors (Ago2/miR-25 DKO and controls).
- Figure 4D: page 13, line 336. The authors said the only up-regulated gene common to all datasets was Syndecan-3. The authors observed that depending on the model analyzed, the treatment, and the time of the treatment, there are changes or not in the expression of Syndecan 3. Melanoma has been associated with hypoxia which it is known to regulate syndecan 3 (D'Aguanno, S., Mallone, F., Marenco, M. et al. Hypoxia-dependent drivers of melanoma progression. J Exp Clin Cancer Res 40, 159 (2021). Prieto-Fernández E, Egia-Mendikute L, Bosch A, García Del Río A, Jimenez-Lasheras B, Antoñana-Vildosola A, Lee SY, Palazon A. Hypoxia Promotes Syndecan-3 Expression in the Tumor Microenvironment. Front Immunol. 2020 Sep 30;11:586977.). I suggest in the analysis of Figure 4d, also including the RNA seq of NTC and NTC-GP to know if Sdc3 is overexpressed under these conditions. Sdc-1 expression in tissues could also be checked by immunohistochemistry in the models in Figure 4d plus the controls and associated with Figures 4f-k.
Minor
• Introduction:
- The introduction must be improved because is very general and should focus more on melanoma since most of the experiments were done on melanoma models, even the clinical data were done on melanoma patient database.
- On page 5 lines 87-93, it is not clear why the miR-17-92 family has an impact on tumorigenesis. The authors wrote only some examples of who these miRNAs are regulated by or who they regulate but it would be better if they could give more examples about the role of these miRNAs in tumorigenesis o what is known about miRNAs analyzed in melanoma.
• Methodology.
- Could the authors please explain why in each model (melanoma, colon, and breast) they injected different amounts of cells? Is it a general protocol, or is it something that they already standardized and if so, please provide the reference. Also, could you please explain why there is a big difference between the number of B16 cells vs. the number of GVAX injected?
- Add clodronate liposome concentration used. Although the authors described it in the figure legend, please write the IFN-γ concentration in the materials and methods as well.
- For intracellular cytometric staining, did the authors use a commercial kit? If so, which one? If not, indicate what was used for fixation and permeabilization.
- Were the analyses made from Zombie Aqua negative cells? did they follow the manufacturer's protocol for Zombie Aqua staining? Of the digested tumor, were all the cells used for cytometry, or was a specific concentration used?
- Please add the concentration or dilution of the antibodies used.
- For Luciferase assay please add the concentration of the cells.
- In general, unless the authors have already published it, please add the citation in the methodology, otherwise describe in more detail the steps followed in all the experiments.
• Discussion
- Page 16, line 429: Our analysis suggests that cancer cells… I consider that we should not write in general and rather direct the discussion towards melanoma since most of the experiments were done on this type of cancer.
- Page 16, line 444: CD68+ CD163-M1 TAMs. add a space between CD163- and M1 because it is not understood that CD163- refers to a negative population for that marker. As it is (CD163-M1) it is understood as if it is an M1 marker when in fact it is an M2.
- In general, I suggest a better discussion of the results. It seems that the results are described again but there is little discussion of them. for example, concerning Syndecan-3 it may have different roles depending on the type of cancer, as it influences multiple signaling pathways in a context-dependent manner.

Reviewer #5

(Remarks to the Author)
I co-reviewed this manuscript with one of the reviewers who provided the listed reports. This is part of the Nature Communications initiative to facilitate training in peer review and to provide appropriate recognition for Early Career Researchers who co-review manuscripts.

Reviewer #6

(Remarks to the Author)
"I co-reviewed this manuscript with one of the reviewers who provided the listed reports. This is part of the Nature Communications initiative to facilitate training in peer review and to provide appropriate recognition for Early Career Researchers who co-review manuscripts."

Reviewer #7

(Remarks to the Author)
I co-reviewed this manuscript with one of the reviewers who provided the listed reports. This is part of the Nature Communications initiative to facilitate training in peer review and to provide appropriate recognition for Early Career

Researchers who co-review manuscripts.

Version 1:

Reviewer comments:

Reviewer #1

(Remarks to the Author)
In my initial review, I raised four key concerns that, in my view, needed to be addressed to rule out the possibility that the authors' findings were not due to off-target effects on miR-25/miR-106.

(1) My first concern was whether the sgRNAs effectively depleted the mature miRNAs. I asked the authors to provide the sequences of the sgRNAs used in the knockout experiments and to isolate subclones in which homozygous targeting of the desired miRNAs could be confirmed. I now understand that the authors targeted either the terminal loop or the stem region of the precursor. However, they report being unable to isolate subclones with homozygous editing, despite describing 50–75% editing in the bulk population. Their explanation—that this reflects the "inherent plasticity of cancer cells"—is unconvincing. Does this mean that cells with edited miR-25 frequently revert to wild type? Or that loss of miR-25 is not tolerated by cancer cells? Neither explanation is persuasive, especially considering that mice lacking the entire miR-106b-25 locus are viable and appear phenotypically normal.

(2) My second concern was that the previous version of the manuscript reported that knockout of miR-106a resulted in the same phenotype as knockout of miR-25, despite the authors' own data showing that miR-106a is expressed at levels 10,000-fold lower than miR-25 in these cells. This raised the strong possibility that the observed phenotype was not due to loss of miRNA expression. In the revised manuscript, the authors acknowledge that miR-106a is barely expressed and have removed references to those experiments. However, this does not explain why knockout of a barely expressed miRNA would yield the same phenotype as knockout of a highly expressed paralog.

(3) A third concern was that RNA-seq data from the miR-25 knockout cell lines showed no evidence of preferential upregulation of predicted miR-25 targets. This type of analysis is the gold standard for assessing loss or gain of miRNA function, and the absence of coherent changes in target mRNA expression is a red flag. In their rebuttal, the authors agree that the RNA-seq data do not show de-repression of miR-25 targets but argue that this is expected because "miRNA-mediated repression mostly occurs through translation," citing two 2008 Nature papers by the Rajewsky and Bartel groups. This is incorrect. Both papers demonstrate that while translational inhibition can contribute to target repression, mRNA destabilization is the primary mechanism of miRNA-mediated gene silencing. For instance, the Baek paper states: "…our results show that mRNA destabilization can explain most of the endogenous miR-223-mediated repression." This was further confirmed in a later Molecular Cell paper ("mRNA destabilization is the dominant effect of mammalian microRNAs by the time substantial repression ensues"; Eichhorn et al., PMC4292926). The abstract of this paper clearly states: "…regardless of the miRNA, cell type, growth condition, or translational state, mRNA destabilization explains most (66% to >90%) miRNA-mediated repression," and "…[these results] also simplify future studies, dramatically extending the known contexts and time points for which monitoring mRNA changes captures most of the direct miRNA effects." Therefore, as I stated in my original review, the lack of target mRNA de-repression in miR-25 KO cells suggests that the knockout was ineffective, that miR-25 levels were too low to be functionally relevant in this cell line, or that other paralogs compensate for miR-25 loss.

(4) Finally, in light of these concerns, I requested that the authors perform a rescue experiment by reintroducing ectopic miR-25 into the miR-25 KO cells. In their rebuttal, the authors claim that this experiment cannot be performed because the sgRNAs remain expressed. This explanation is unconvincing. Transient expression of the sgRNAs or Cas9 could easily be used, or the mature miR-25 sequence could be expressed from a pri-miRNA backbone resistant to the sgRNAs.

In conclusion, the lack of mRNA target destabilization, the unexplained phenotypic similarity between miR-106a and miR-25 knockouts, the absence of de-repression of miR-25 targets, and the failure to perform rescue experiments leads me to conclude that as it stands the authors' interpretation is not sufficiently supported by the experimental evidence provided.

Reviewer #2

(Remarks to the Author)
While the practical means targeting miR-25(other than genetic manipulations of its expression in tumor cells) remain to be identified (if at all possible), the authors have addressed most of my other concerns by restructuring the manuscript and by providing new data. Their findings of miR-25 as a major driver of therapeutic resistance to anti-PD-1 and as a TME-remodeler are worth being considered for publication in Nature Communications. I have no other concerns.

Reviewer #3

(Remarks to the Author)
Overall, the authors have adequately addressed most of our previous concerns. However, there are some outstanding concerns that can be addressed mostly through editing of the manuscript.

9. RNA-sequencing was performed on unsorted tumors, therefore it is unclear from which cell population the

changes in innate immune signaling are coming and whether it is a feature of multiple cells and cell types or driven by a single cell population exhibiting much higher levels of immune genes/pathways. It is also unclear if these upregulated immune pathways are observed solely due to the presence of more CD45+ cells in the tumor. Some of the gene expression differences in Fig. 2g appear to be inconsistently high in one of the three miR-25 KO GP samples (Cfp, Tlr9, C1rl, Cfd, C3, Serping1, C1s1, Clu, Cfh).
Response:
(1) Cell population-specific changes.
To directly address this concern, we have restructured the manuscript to place greater emphasis on scRNA-seq analysis using the MC38 model, which forms the basis for our mechanistic insights (Figure 2).
Our scRNA-seq analysis clearly shows that classical complement pathway genes—including C1s1, C3, C4b, and Cfh (Figure 2H)—are primarily expressed by fibroblasts (C30), as indicated by strong expression of canonical fibroblast markers such as Col1a1 and Col1a2 (Figure S2C). This is accompanied by a marked upregulation of inflammatory CAF (iCAF) markers and a concomitant reduction in myofibroblastic CAF (myCAF) markers, suggesting a shift toward a pro-inflammatory CAF phenotype in miR-25–deficient tumors undergoing immune ICT.
Importantly, these complement-related gene changes are not attributable to increased immune infiltration (e.g., CD45+ cells), but rather reflect a tumor-intrinsic and stroma-intrinsic remodeling of the TME. This cell-type–specific insight strengthens our conclusion that miR-25 regulates immune evasion not just through immune cell recruitment, but also through modulation of stromal fibroblast function.
(2) Variability in innate immune gene expression among miR-25 KO GP tumors
We acknowledge the reviewer's concern about variability in innate immune gene expression across miR-25 KO GP samples. Inter-sample heterogeneity is an inherent feature of in vivo tumor models, particularly within immune-modulated settings. To further clarify this point and align the B16 model with our MC38 scRNA-seq findings, we conducted additional analyses, including immunohistochemistry for complement component C3 and flow cytometry for macrophage activation markers.
We have included the original B16 bulk RNA-seq dataset to Supplemental Figure S2A, where it is used to illustrate general innate immune activation rather than to draw mechanistic conclusions. The full differential expression results are available in Supplemental Table 8, sheet: "DEseq_bulk_B16_invivo."

The wording in the text suggests that the activation of antigen-receptor in M1-like macrophages (line 331) precedes and drives complement activation and humoral immunity in CAFs. Authors present evidence that both are occurring, and CellChat suggests that macrophages and fibroblasts are interacting, but the data does not demonstrate which activation event occurs first and whether it is necessary or sufficient to drive the second activation event. Examining the effect liposome clodronate treatment had on CAFs would strengthen this connection. Otherwise, the authors should temper the definitive tone of these conclusions.

12. Despite efficient reduction of splenic and tumoral macrophages by LC treatment, the lack of difference in tumor mass in the yellow and red groups casts doubt whether miR-25 KO GP treatment works through macrophage infiltration. Additionally, without sequencing of the miR-25 KO GP tumors, suggesting that complement signaling has changed is slightly overreaching.
Response:
We appreciate the reviewer's thoughtful suggestion and agree that additional validation is important to support our mechanistic conclusions. Many of the relevant points have been addressed in our response above to specific point 11.
To further clarify the role of macrophages in complement signaling, we performed immunohistochemistry (IHC) for complement component C3 in tumor sections. We observed a marked reduction in C3 deposition following macrophage depletion (Figure 3M), suggesting that complement activation—particularly C3 accumulation—is dependent on macrophages in our model and is downstream of miR-25 regulation.
Although we did not perform RNA-seq specifically on macrophage-depleted tumors, the combination of diminished C3 signal by IHC, effective macrophage depletion, and reduced tumor mass in these groups strongly supports the conclusion that macrophages play a central role in mediating the complement-driven immune remodeling observed in miR-25 KO tumors. We also updated Discussion section lines 603 – 621 to clarify this interpretation.

In Figure 3 the % of macrophages in the KO+LC group is equivalent to the percent in the KO+PBS in Figure 3L, yet Figure 3M shows a marked reduction of C3 in the KO+LC. There could be a phenotypic change in macrophage polarization due to LC treatment that alters complement activation in the CAFs that should be acknowledged.

15. Decrease in macrophage interferon signaling during immunotherapy response inconsistent with what's known in the literature and claims in Figure 4. Interferon gamma levels were unaddressed. Additionally, the complement signaling in the MC38 appears to come from fibroblasts instead of macrophages like in the B16F10.
Response:
(1) Inconsistency of macrophage interferon signaling and interferon gamma levels
In our dataset, we observed a downregulation of type I IFN–associated genes—including Ifitm2, Ifitm3, Isg15, Ifit2, among others—as well as decreased expression of M2 polarization–related genes in M1-like antigen-presenting macrophages (cluster C18) (Figure 2E). Type I interferons (e.g., Ifit2, Ifit3, Ifitm2, and Ifitm3) are classically known for their antiviral roles.

Although IFN-I has been used in solid tumor treatment since the 1980s, its clinical success has been limited. Recent studies suggest IFN-I has context-dependent effects in the tumor microenvironment (TME), and in some cases, may even support immunosuppressive programs. For example, IFN-I–treated monocytes have been shown to induce Arg1 expression (encoding arginase-1), which promotes a tumor-supportive phenotype in TAMs.

Based on these findings, we hypothesize that in our system, the downregulation of type I IFN genes in macrophages may reflect a shift away from suppressive, M2-like functions toward a more pro-inflammatory, antigen-presenting M1-like state in miR-25 KO tumors. In contrast, type II IFN (IFN-γ) plays a distinct and central role in our model by mediating tumor intrinsic resistance to ICT. Anti–PD-1 therapy enhances $CD8^+$ T cell–mediated cytotoxicity and elevates IFN-γ production, which promotes tumor cell killing. We found that miR-25 KO sensitize tumor cells to immune pressure in an IFN-γ–dependent manner (Figure 4A), a finding further supported by IFN-γ depletion experiments. IFN-γ depletion reverted the therapeutic effects of miR-25 KO, including suppression of tumor growth, activation of macrophages, and complement infiltration (Figures 4B – 4E). These effects were accompanied by reverted expression of Sdc3 in vitro without IFN-γ (Figures 5B, 5E, and S5A), supporting our model that miR-25 represses Sdc3 in an IFN-γ-dependent context, thereby facilitating immune recognition and clearance of tumor cells.

(2) Interferon gamma levels were unaddressed

In the revised manuscript, we addressed IFN-γ levels by analyzing tumor cell–specific IFN-γ response signatures using our scRNA-seq dataset (Figures 4A and S4D). To further validate the functional relevance of IFN-γ, we performed IFN-γ depletion experiments in vivo (Figure 4B – 4E), which reversed the therapeutic effects of miR-25 knockout. Additionally, in vitro analysis showed that Sdc3 expression was restored in the absence of IFN-γ (Figures 5B, 5E, and S5A), supporting the conclusion that IFN-γ directly influences miR-25–mediated tumor-intrinsic responses during immune checkpoint therapy.

(3) Complement signaling in B16

We appreciate the reviewer's insightful comment regarding the source of complement C3 in the B16 melanoma model. We do not claim that macrophages are the source of C3 in B16, as our IHC staining cannot definitively determine the cellular origin of C3. However, we observed highly consistent phenotypes in B16 tumors that mirror those in the MC38 model—including reduced M2-like macrophage populations, increased MHC II+ macrophages, and elevated complement C3 activation.

Given these similarities, we believe the most likely source of C3 in B16 tumors is also fibroblasts, as observed in the MC38 scRNA-seq data.

My concern with the IFNg blockade in vivo experiments is that the overall reduction in macrophages and the increase in MHC2+ macrophages resulted in increased tumor size in KO+a-IFNg, while previously it was argued that these characteristics helped facilitate the reduction in tumor size upon miR-25 KO in MC38 and B16F10. This seems to suggest that CAF polarization is the most prevalent driver of miR-25 KO. I agree that this indicates CAFs are likely the primary source of C3, but in Figure 4E (like in Figure 3M) the loss of C3 occurs between the KO + Isotype and KO + a-IFNg, while the two conditions possess the same amount of macrophages and MHC2+ macrophages. The (presumed M1-like) MHC2+ macrophages being equivalent between the two groups also suggests that antigen-receptor activation does not precede complement activation in the CAFs, as was mentioned earlier (line 331).

Lastly, in Figure 4K you describe the levels of C3 linked to the changes in TAMs, but the levels in 4J are comparable to 3L, but the C3 staining in 4K is much different than in 3M; it is therefore unclear how authors link C3 staining to macrophage level.

Figure 5

21. It appears authors saw no differences by CIBERSORT in M1 and M2 macrophages like their murine models but instead chose to focus on M0 macrophages. The quantity of delta gamma T cells is very low and marginally different between low and high groups. miR-25 showed very weak negative correlation with complement genes.

Response:

We thank the reviewer for the question. We have removed the CIBERSORT analysis from the revised manuscript for the following reasons:

(1) In our scRNA-seq dataset, antigen-presenting macrophages (M1-like) could not be confidently classified as classical M1 macrophages, as many canonical M1 markers remained unchanged (Figure 2D). This observation was further validated in the B16 melanoma miR-25 KO model, where we observed no significant changes in $CD86^+$ macrophages (Figure 3I). Therefore, the CIBERSORT-based estimation of macrophage infiltration may not accurately reflect the macrophage phenotypes present in our models.

(2) We also removed T cell population analysis from the CIBERSORT output, as the marker sets used by CIBERSORT may not reliably distinguish T cell subsets in our experimental context.

(3) Our current analysis is focused on miR-25, and its immune regulatory function. To this end, we highlight the strong negative correlation between miR-25 expression and complement gene signatures, as shown in our GSEA analysis (Figures 3B and 3C).

You coultd try inputting your particular macrophage and T cell gene signatures into CIBERSORT to see if the specific populations you saw by scRNA-seq are also differentially present in patient's data.

22. There are very few responders in the immunotherapy dataset. Many of the nonresponders also had low miR-25 levels. Negative correlation with complement genes is not very convincing as some of the lowest expressing miR-25 patients had very low complement activity' the negative association is only clearly evident in one patient. miR-17HG appears positively correlated with miR-25, unlike in panel f.

Response:
We thank the reviewer for raising the question regarding the immunotherapy dataset. Due to the limited number of available patient samples, we have removed this analysis from the revised manuscript. The small sample size precludes any reliable conclusions regarding the correlation between miR-25, Sdc3, and complement activation, and we believe its inclusion would not meet the rigor expected for clinical relevance.

It would be beneficial to see if there are any published scRNAseq datasets that can demonstrate miR-25 is reduced (or Sdc3 increased) specifically in tumor cells with increased immune infiltration or in responders to PD1/ICT (is this unique to PD1 monotherapy or applicable to CTLA4/LAG3 combo and PDL1 as well?). More evidence that the effects of miR-25 KO or Sdc3 upregulation are relevant in human patient samples or human cell lines would be beneficial. Is there evidence that miR-25 levels are higher during disease progression, or higher in tumor relative to normal tissue? The evidence presented suggests a phenotype in multiple distinct tumor types so it should be possible to find a clinical connection in at least one tumor type. For melanoma immunotherapy transcriptomics and single cell you can check Hugo et al., Cell 2016; Riaz et al., Cell 2017; Gide et al., Cell 2019; Jerby-Arnon et al., Cell 2018; Pozniak et al., Cell 2024.

23. It is still unclear where IFN-y is coming from in the in vivo murine models and whether it negatively correlates with miR-25 in patients. They should also examine if Sdc3 mRNA levels inversely correlate with miR-25 in patient samples to further confirm the relevance of the proposed model to human.
Response:
We appreciate the reviewer for raising these important questions.
(1) Regarding the source of IFN-γ
IFN-γ secretion is a hallmark of enhanced anti-tumor immunity. Anti–PD-1 immunotherapy promotes $CD8^+$ T cell–mediated cytotoxicity and leads to increased secretion of IFN-γ, which contributes to tumor cell killing 37-39
.
Our analysis of TCGA-SKCM-TM patient data revealed a negative correlation between miR-25 expression and the IFN-γ response signature, as shown by GSEA in Figure 3B.
(2) Sdc3 mRNA levels and correlation with miR-25 in patient samples
Sdc3 has historically been reported as a neuron-enriched gene, primarily expressed in the brain.
54,55 However,
recent scRNA-seq studies, 56 including our own dataset, have revealed strong Sdc3 expression in immune-related tissues such as the spleen and lymph nodes (Figures S6A and S6C), indicating a broader tissue distribution than previously appreciated.
Given the cellular heterogeneity of tumors—including malignant, immune, stromal, and potentially neuronal components—bulk RNA-seq data from patient samples may not accurately reflect tumor cell–intrinsic correlations between miR-25 and Sdc3. Therefore, such correlation analyses using bulk transcriptomic data must be interpreted with caution.
That said, in TCGA-SKCM-TM samples, tumors with low miR-25 expression showed enhanced immune activation (Figure S3B) and downregulation of synapse-related pathways (Figure S6D), suggesting the presence of a neuron-like gene signature in epithelial tumors. These findings may explain why miR-25 knockout consistently elicits immune-stimulatory effects across three epithelial cancer models—B16 (melanoma), MC38 (colon), and 4T1 (triple-negative breast cancer)—under immune checkpoint therapy.
We hypothesize that Sdc3, an atypical cell surface antigen in epithelial tumors, may trigger neuron-associated inflammatory signaling in the tumor microenvironment. However, the mechanisms underlying ectopic Sdc3 expression in epithelial cancers and its role in tumor progression, ECM remodeling, or metastasis remain to be elucidated. We also updated Discussion section lines 676 – 722 to clarify this interpretation.

A stronger connection from the mechanistic mouse work to either human cell line or patient data is one of the key deficiencies of the story, but the survival differences and upregulated immune pathway activation in miR-25 low samples does partially address this. Maybe authors could check if these characteristics are also present in Sdc3 low vs high samples? And are Sdc3 levels higher in miR-25 low samples? I understand it they may not be, due to the bulk nature of the sequencing data, but it would be interesting to see.

Reviewer #4

(Remarks to the Author)
The authors have appropriately addressed our previous comments.

Minor:
- Figure s5J is not described in the results section and was described as 4J in the rebuttal letter.
- the general references on syndecans (reviews) are over 30 years old. Admittedly, the content (citing the putative GAG attachment sites) is still valid, but the authors may want to consider replacing either reference 53 or 54 with a more recent review on syndecans, eg. an inflammation/immunity related one.

Reviewer #5

(Remarks to the Author)
I co-reviewed this manuscript with one of the reviewers who provided the listed reports. This is part of the Nature Communications initiative to facilitate training in peer review and to provide appropriate recognition for Early Career Researchers who co-review manuscripts.

Reviewer #6

(Remarks to the Author)
I co-reviewed this manuscript with one of the reviewers who provided the listed reports. This is part of the Nature Communications initiative to facilitate training in peer review and to provide appropriate recognition for Early Career Researchers who co-review manuscripts.

Reviewer #7

(Remarks to the Author)
I co-reviewed this manuscript with one of the reviewers who provided the listed reports. This is part of the Nature Communications initiative to facilitate training in peer review and to provide appropriate recognition for Early Career Researchers who co-review manuscripts.

Version 2:

Reviewer comments:

Reviewer #1

(Remarks to the Author)
I commend the authors for the extensive revision of the manuscript. The rescue experiments and the generation of miR-25 knockout clones are compelling and confirm the originally described phenotype, effectively addressing my most significant concern.

I remain puzzled by the previously reported results for miR-106, and I do not agree with the comment that RNA-seq is less accurate than RT-PCR for determining miRNA abundance.

One remaining point that should be addressed concerns the cumulative distribution functon(CDF) plots shown in Rebuttal Figure 5d. As currently presented, these plots cannot be interpreted because the x-axis spans an excessively broad range (from −10 to > +5 log fold change), which necessarily compresses any biologically meaningful differences; this is particularly problematic given that the highly significant KS test strongly suggests the presence of such differences. In general, the x-axis in these plots should be restricted to a narrower range (e.g., −2 to +2 log fold change) to allow proper visualization and interpretation. This issue should be corrected prior to publication.

Reviewer #3

(Remarks to the Author)
The revised version includes additional data analyses in response to prior concerns. However, some aspects of the model downstream of miR-25 remain unclear, particularly with regard to the crosstalk between specific macrophage populations and CAFs (e.g., which specific macrophage populations drive complement signaling in CAFs and their polarization to iCAFs). Nonetheless, given the extensive data already presented in this study regarding miR-25 modulation of Sdc3 in response to IFNγ in the context of ICB resistance, additional characterization could be the subject of future investigation.

Reviewer #5

(Remarks to the Author)
I co-reviewed this manuscript with one of the reviewers who provided the listed reports. This is part of the Nature Communications initiative to facilitate training in peer review and to provide appropriate recognition for Early Career Researchers who co-review manuscripts.

Open Access This Peer Review File is licensed under a Creative Commons Attribution 4.0 International License, which permits use, sharing, adaptation, distribution and reproduction in any medium or format, as long as you give appropriate credit to the original author(s) and the source, provide a link to the Creative Commons license, and indicate if changes were made.
In cases where reviewers are anonymous, credit should be given to 'Anonymous Referee' and the source.
The images or other third party material in this Peer Review File are included in the article's Creative Commons license, unless indicated otherwise in a credit line to the material. If material is not included in the article's Creative Commons license and your intended use is not permitted by statutory regulation or exceeds the permitted use, you will need to obtain permission directly from the copyright holder.
To view a copy of this license, visit https://creativecommons.org/licenses/by/4.0/

We thank the reviewers for their constructive criticism, which improved the quality of our manuscript. We have addressed the reviewers' question in the revised manuscript as described below in blue text. Cited references are presented at the end of all responses.

A summary of the comprehensive and rigorous revision of the study is outlined below.

1. Manuscript Restructuring for Improved Clarity and Flow
We reorganized the manuscript to enhance narrative coherence, with a clear progression from discovery to mechanism and cross-model validation.

2. Comprehensive re-analysis of RNA-seq datasets
All transcriptomic data—including small RNA-seq and single-cell RNA-seq (scRNA-seq)—were re-analyzed with increased statistical rigor. We clarified group comparisons to improve interpretability and ensured robust biological conclusions.

3. Repeated and Expanded In Vivo Functional Validation
All key in vivo experiments—including macrophage and IFN-γ depletion—were repeated, and tumor weights were added to confirm tumor volume findings.

4. Mechanistic Validation of the miR-25–Sdc3 Regulatory Axis
We conducted a comprehensive set of experiments to establish Sdc3 as a direct and functional target of miR-25, including:
(1) Repeated Western blotting
(2) RIP-qPCR to demonstrate RISC binding
(3) Luciferase assays using full-length Sdc3 3′UTR with site-directed mutagenesis in cells expressing endogenous miR-25
(4) CRISPR base editing of the predicted miR-25 site to confirm the specificity of regulation in vitro and in vivo

5. Expanded Discussion of Mechanistic and Translational Implications
The revised discussion provides a deeper interpretation of the roles of miR-25, tumor-associated macrophages (TAMs), cancer-associated fibroblasts (CAFs), and Sdc3 in shaping the tumor microenvironment and resistance to immune checkpoint therapy.

Reviewers' comments:

Reviewer #1 (Remarks to the Author): with expertise in miRNAs in cancer

Please, see full review in the attached pdf file.

In this manuscript, Zhu and colleagues report a novel function for miRNAs in the response of tumors to immunotherapy. Using a series of syngeneic mouse models and CRISPR-based KO of miRNAs, they propose that miR-25 and miR-106 facilitate tumor immune evasion. For miR-25, they propose that this effect is mediated, at least in part, by direct repression of Syndecan 3 (Sdc3). According to this model, loss of miR-25 function would result to derepression of Sdc3, in turn increasing macrophage recognition and facilitating both immune activation and clearing of tumor cells (Figure 6).
The manuscript is well written the data are generally well presented and the relevant scientific literature is cited. The topic of this work is potentially of clinical relevance and describes a previously unappreciated role for miRNAs in tumorigenesis.
However, a careful analysis of the primary data suggest that alternative explanations for the observed phenotype are possible and indicate that essential controls are missing.
In particular, the authors should address the following major concerns:

(1) Evidence for effective CRISPR-mediated loss of miR-25 and miR-106 function is insufficient

The authors describe the use of 3 sgRNAs for the inactivation of miR-25 and 3 sgRNA for the inactivation of miR-106, however the sequence of the sgRNAs is not reported in the manuscript (at least I couldn't find it) and therefore it is unclear where Cas9 is cleaving within the miRNAs and in particular in relation to its seed sequence, which is the key determinant of substrate specificity. It is important to note that CRISPR editing generally results in small insertions/deletions near the cleavage sites. If the site is far from the seed, these may have no effect on miRNA function (but possibly impair miRNA detection by RT-PCR). If, on the other hand, the site is within the seed, cleavage could result in changed substrate specificity rather than total loss of miRNA function (a neomorphic mutation). At a minimum, the authors should perform high-throughput sequencing of the edited alleles to conclusively establish the efficiency and the nature of the editing in the bulk population. Ideally, however, the authors should isolate subclones with confirmed homozygous loss of the miRNA (large deletion encompassing most of the seed) and repeat the in vivo experiments. As a related issue, in the main text it should be described which sgRNA(s) where in the end used for the in vivo experiments throughout the paper.

Response:

We appreciate this valuable suggestion. In response, we have updated the supplemental materials, and the sequences of the sgRNAs that we used to target miR-25, are now provided in the revised **Supplemental Table 3, sheet "Primers for sgRNAs."**

The rationale behind our sgRNA design is grounded in the principles of miRNA biogenesis. MicroRNAs are transcribed from genomic DNA by RNA polymerase II and initially form a hairpin-loop structure known as pri-miRNA. This structure is recognized and cleaved by Drosha, producing a pre-miRNA, which is then exported to the cytoplasm. In the cytoplasm, the RNase III enzyme Dicer cleaves the pre-miRNA hairpin to generate a miRNA: miRNA* duplex. In our study, only miR-25-3p is incorporated into the RNA-induced silencing complex (RISC) to carry out gene silencing functions **(Figure S1C)**.

Cas9 typically introduces double-stranded breaks three to four base pairs upstream of the PAM sequence. [1-3] As annotated in updated **Figure S1D**, sgRNA1 and sgRNA2 were designed to target and disrupt base-pairing within the core stem region of the pri-miR-25 hairpin, which is essential for proper miRNA processing. Although sgRNA3 targets a site outside the main stem, it is located within the terminal loop region, which plays a critical role in Dicer recognition and processing of the pre-miRNA into mature miR-25-3p. Given the positioning of all three sgRNAs, we expect that none of them would allow to produce functional mature miR-25-3p.

To validate CRISPR-Cas9-mediated knockout (KO), we sequenced the edited alleles and analyzed the results using TIDE (https://tide.nki.nl), a well-established tool for assessing genome editing efficiency. [4] TIDE is a robust method for determining the spectrum and frequency of insertions and deletions (indels) in heterogeneous cell populations following CRISPR/Cas9 editing. This approach has been used in numerous recent publications to evaluate editing efficiency. [5-9] Our analysis confirmed that CRISPR-Cas9-mediated KO of miR-25 resulted in an overall 50 –75% editing efficiency in the bulk population of cells **(Figure 1F)**. This level of editing correlates well with the reduction in mature miR-25 levels observed in the three mouse tumor cell lines, as measured by RT-qPCR **(Figure 1G)**.

In summary, we utilized three distinct sgRNAs to interfere miR-25 biogenesis, thereby inhibiting the production of functional miR-25-3p. Importantly, despite targeting different regions, each sgRNA induced a comparable phenotype across three syngeneic mouse cancer models **(Figures 1H – 1P)**. Therefore, these results demonstrate that the observed effects are unlikely due to unintended effects such as substrate alterations or neomorphic mutations.

We also appreciate the reviewer's suggestion to **isolate subclones with confirmed homozygous loss of miR-25**. In our attempts to generate single-cell clones using our lentivirus-based CRISPR-Cas9 system, we were unable to maintain stable homozygous deletions over time. We suspect this instability may be related to the inherent genomic plasticity of cancer cells. Nevertheless, given the high knockout efficiency achieved in the bulk population and the consistent in vivo phenotypes observed, we proceeded with bulk-edited cells for downstream experiments. We believe this approach effectively captures the functional consequences of miR-25 loss in our model.

(2) The choice of miR-106a and the resulting phenotype are puzzling

I am puzzled by the choice of the authors to knock-out miR-106a. According to their own primary data (Table 1), miR-106a is NOT down-regulated in response to immunotherapy. In fact is upregulated by approximately 8 fold (see table below). In addition, this miRNA is a member of the miR-106~363 cluster, located on the X chromosome and generally expressed at much lower levels than miR-106b~25 (which encodes miR25) and miR-17~92 (which encodes miR-92a). In fact, according to Table 1, the levels of miR-106a are extremely low compared to miR-25 (base mean of 9 vs base mean of 16235). It is highly unlikely that loss of a miRNA expressed at such low levels would have any phenotypic consequence, especially considering that its paralog miR-106b—which has the same seed and the same targets—is expressed nearly 1000 fold more in the same cells. In other words, the observation that its knockout has a phenotype identical to the phenotype observed upon KO of miR-25, which is expressed almost 10000 fold more, needs an explanation.

miRNA	baseMean	log2FoldChange	lfcSE	stat.	padj
Mir106a	9.11533862	2.9497855	0.64000287	4.60901914	0.00027335
Mir25	16235.4436	-0.6720933	0.24046043	-2.7950269	0.03590923
Mir106b	1727.95711	-0.4437295	0.1074967	-4.1278434	0.00110221

Response:

Thank you for raising this important point. We agree that it is a critical consideration and have reanalyzed the small RNA-seq data accordingly. As part of this reanalysis, we also refined our criteria across all RNA-seq analyses, applying a more stringent threshold: $|\log_2 FC| > 1$ and adjusted p-value (padj) < 0.05.

miR-106a was initially included to provide broader context across the miR-17-92 family and its paralogs. However, under the revised statistical criteria, miR-106a was no longer significantly differentially expressed. In contrast, several other members of the miR-17-92 and miR-106b-25 clusters—including miR-17, miR-18a, miR-19a, miR-20a, miR-93, and miR-106b—remained significantly downregulated. Notably, miR-25 was the only miRNA significantly and consistently downregulated in both the Combination (GVAX + anti-PD1) vs. Control and Combination vs. GVAX comparisons **(Figure 1C)**. Among all significantly downregulated miRNAs, miR-25 also exhibited the highest expression level, with a base mean of 17,315.96 **(Figure 1D; Supplemental Table 1, sheet "Comb_vs_GVAX")**.

To keep our findings focused and coherent, we chose to center all subsequent analyses and validations on miR-25. To validate the observed downregulation, we performed RT-qPCR on additional mouse tumor samples. These results confirmed that miR-25 is significantly decreased in tumors treated with combination therapy, particularly in those that responded to the treatment **(Figure 1D)**.

(3) The primary data provided show no evidence of loss of miR-25-mediated gene repression in miR25 KO cells (Table 4)

Deletion of abundantly expressed miRNAs invariably results in the preferential upregulation of their predicted targets and this can be easily detected by observing a shift to the right in the cumulative distribution of the log2FC of targets vs non-targets, as determined by RNASeq. I commend the authors for performing RNASeq analysis on RNAs extracted from miR-25-KO and NTC cells in vitro (table 4), but I was surprised that the authors didn't use this dataset to confirm the derepression of miR-25 targets.
I performed such analysis comparing the log2FC (miR-25KO vs NTC) of predicted conserved miR-25 targets (according to Targetscan) and background (all the other genes). As shown in the ecdf plot below, there is no sign of derepression of miR-25 targets in this dataset, suggesting that either the miR-25KO was partial, that miR-25 levels are not high enough to be functionally relevant in this cell line, or that miR-25 paralogs can compensate for loss of miR-25.

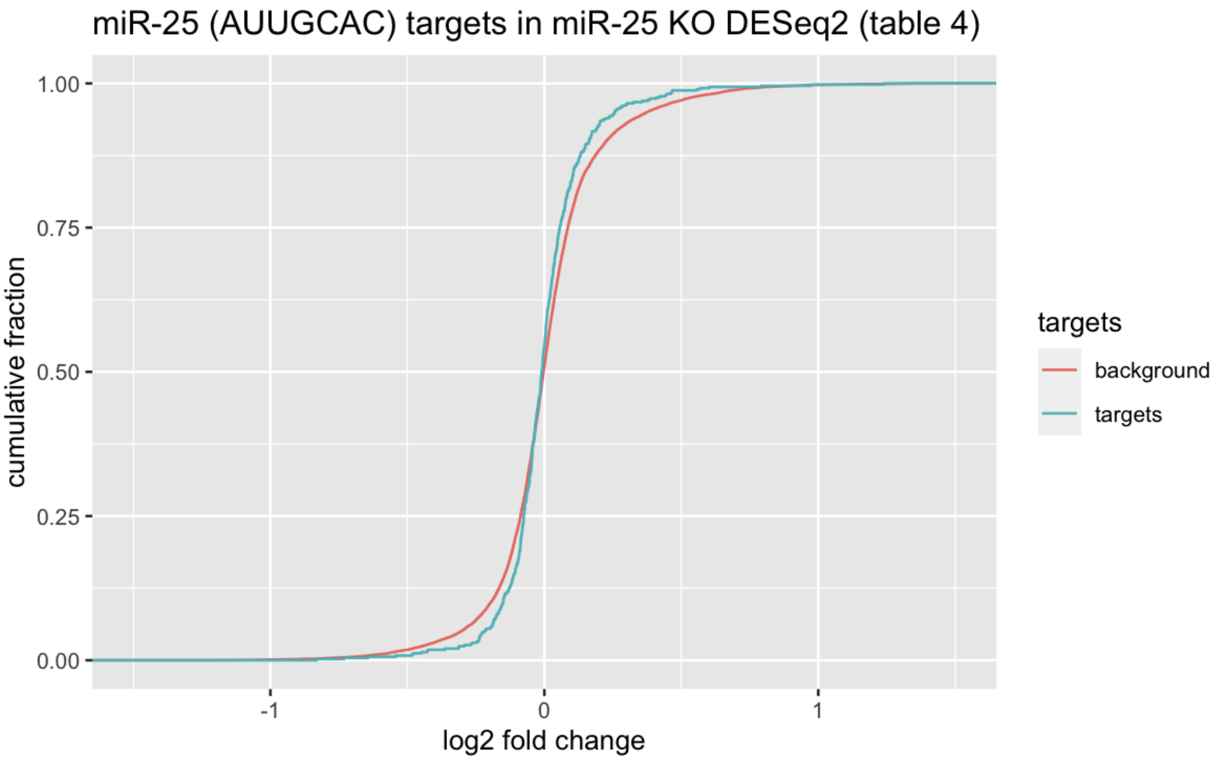

Response:

We thank the reviewer's thoughtful analysis and their effort to perform an independent cumulative distribution function (CDF) plot comparing predicted miR-25 targets and background genes. We agree that loss of an abundantly expressed miRNA often results in derepression of its predicted targets, typically seen as a rightward shift in the cumulative distribution of log_2 fold changes. However, we consider the lack of a clear derepression signature in our RNA-seq data may be due to the following factors.

First, it is well established that miRNA-mediated repression mostly occurs through translational repression. [10,11] Therefore, transcriptomic profiling alone may not fully capture the functional consequences of miR-25 depletion.

Second, miRNA activity is highly context-dependent, and its impact on gene expression can vary across different cell types and physiological conditions. The B16F10 cell line used in our study is a highly aggressive, genomically unstable, and poorly differentiated murine melanoma model, [12-14] which may exhibit different levels of miRNA activity. In line with this, we did not observe typical miR-25-loss phenotypes in cancer such as altered proliferation or apoptosis [15,16] in vitro **(Figure 1H)** or in vivo **(Figure 1K, left)**, despite confirmed CRISPR editing efficiency **(Figure 1F)** and reduced mature miR-25 expression levels **(Figure 1G)**. These findings suggest that, under basal conditions, miR-25 may noy strongly influence target mRNA abundance in this tumor context.

Taken together, we propose that miRNA regulation in highly immune-evasive, evolved cancer cells, such as B16, is strongly context-specific and may not necessarily manifest as widespread changes in transcript levels following miRNA knockout in original cells. In response to the reviewer's concern, we no longer rely on basal condition RNA-seq to infer miR-25 target. Instead, we have updated our in vitro RNA-seq analysis based on IFN-γ treatment in the revised **Supplemental Table 9** and present these findings in **Figures 4L, S4I and S4J**.

While we acknowledge the limitations inherent to transcriptome-level analyses, we believe that our complementary evidence—including confirmed CRISPR editing efficiency, reduced mature miR-25 expression, consistent in vivo phenotypes, and context-specific dependence on IFN-γ signaling—collectively support the functional relevance of miR-25 in our model.

(4) Rescue experiments should be performed

Given the uncertainty related to the loss of miR-activity, they authors should perform rescue experiments in which retroviruses encoding miR-25 are added back to miR-25KO cells. If the phenotype they observed is in fact mediated by loss of miR-25 function, it should be rescued by miR-25 add-back. This should also be done with respect to miR-106a, where the phenotype observed is even more surprising (see point 2).

Response:

Thank you for this great suggestion. However, we would like to note several technical limitations that may compromise the feasibility and interpretability of the approach.

First, effective overexpression of miRNAs using retroviral vectors requires incorporation of the full genomic sequence of the miRNA, which must then be correctly processed by the endogenous microRNA biogenesis machinery **(Figure S1C)**. In our study, the sgRNAs were specifically designed to target the core hairpin structure and/or Dicer processing sites of miR-25 **(Figure S1D)**. Consequently, Cas9-mediated cleavage remains active in miR-25 KO cells and may disrupt the maturation of any overexpressed retroviral miR-25 transcript, potentially leading to its misprocessing. This would limit the production of mature, functional miR-25-3p, thereby compromising the reliability of the rescue experiment.

We also considered alternative strategies, such as using synthetic pre-miR-25 (mimics) for RNA-level overexpression. However, these mimics lack sufficient stability to persist for the duration of in vivo experiments (typically over two weeks). Additionally, intratumoral injection of such mimics could introduce further complexity by impacting other cell types within the tumor microenvironment, including infiltrating immune cells, which would confound interpretation.

Importantly, we propose that the NTC (non-targeting control) control cells—expressing endogenous, abundant levels of miR-25-3p—already serve as a functional reference. These tumors do not display the phenotypes observed in miR-25 KO, further supporting the specificity of the effects attributed to miR-25 loss.

Given these considerations, we believe that traditional rescue experiments using retroviral or mimic-based reintroduction of miR-25 may not be technically feasible or biologically interpretable. Nevertheless, we fully agree with the importance of validating the specificity of the miRNA-targeting strategy. To that end, we have included multiple lines of supporting evidence throughout the manuscript, including CRISPR editing

confirmation, reduced mature miR-25 expression, in vivo functional data, and downstream target regulation by specific base editing **(Figures 5L – 5N)**.

(5) The validation of Sdc3 as a relevant miR-25 target is inconclusive
Line 342 (Figure 4e): The experiment reported here indicates that ectopic expression of miR25 can repress the Sdc3 3'UTR. But the real issue is whether the endogenous levels of miR25 present in these cells can do it and whether this effect is lost in miR-25KO cell.
To conclusively address this point, the authors should have performed the reporter assay in both WT and miR-25KO cells without adding exogenous miR-25.

Figure 4g/h/i: The included western blots are not congruent with the presented claims. It appears as if (i) B16 cells are showing increased levels at baseline in NTC vs miR-25 KO, (ii) MC38 cells are not showing any difference at baseline and 96 h in NTC vs miR-25 KO and iii) 4T1 cells are showing increased levels of Sdc3 in miR-25 KO vs NTC at all timepoints.

Response:

(1) Endogenous miR luciferase assay:

We fully agree with the reviewer that assessing the regulatory effect of endogenous miR-25 on Sdc3 is critical to validating it as a functional target. In response and incorporating suggestions from **Reviewer #3, specific point 19**, we constructed a firefly luciferase reporter plasmid containing the full-length Sdc3 3′ UTR **(Figure 4M)**. This reporter was transfected into B16 cells with or without endogenous miR-25 expression (i.e., NTC vs. miR-25 KO), followed by 72-hour IFN-γ treatment to simulate the immune-activated context in which Sdc3 regulation occurs. We observed approximately a 90% reduction in luciferase activity in miR-25–proficient cells (NTC-WT) compared to miR-25–deficient cells (KO-WT) **(Figure 4N)**, indicating that endogenous miR-25 is sufficient to repress Sdc3 expression under these conditions. Furthermore, when the predicted miR-25 binding site within the Sdc3 3′ UTR was deleted (mutant reporter: NTC-MT and KO-MT), this repression was abolished, confirming the specificity of the interaction **(Figure 4N)**.

These results provide strong functional evidence that endogenous miR-25 directly regulates Sdc3 in tumor cells, particularly in the presence of IFN-γ stimulation.

(2) Western blot results:

We repeated all Western blot experiments, including both the baseline (0-hour) and the most relevant post–IFN-γ stimulation time point for each cell line. These updated results are now presented in **Figures 5B – 5D** of the revised manuscript. For 4T1 cells, we repeated the Sdc3 Western blot across all time points and confirmed that 48 hours of IFN-γ treatment produces the most pronounced changes. These results are consistent with the corresponding Sdc3 mRNA levels by RT-qPCR **(Figure S5C)**.

Our central claim is that miR-25 regulates Sdc3 expression primarily in the context of immune activation, particularly under IFN-γ stimulation. At baseline (0 h, no IFN-γ), Sdc3 protein levels are not necessarily governed by miR-25, and thus, may not be consistently elevated in miR-25 KO cells. However, upon IFN-γ exposure, all three tumor cell lines—B16 (72 h), MC38 (48 h), and 4T1 (48 h)—show consistent upregulation of Sdc3 at both mRNA and protein levels in miR-25 KO cells compared to controls **(Figures 5B – 5D)**.

Minor points
Line 132 (Figure 1c) : Small RNA sequencing was performed in tumors post anti-PD1 therapy, hence miRNAs of the miR-17-92 family were downregulated in residual cancer cells that were refractory to the therapy. Wouldn’t an alternative interpretation suggest that cancer cells with high miR-17/92 levels are cleared more effectively in this system?**

Response:

We thank the reviewer for this thoughtful comment. It is indeed plausible that tumor cells with high miR-17/92 expression may be more effectively cleared by anti–PD-1 therapy, contributing to the observed downregulation in residual tumors. However, several lines of evidence support the alternative interpretation that miR-25 (a representative member of the miR-17/92 family and the paralogs) contributes to tumor immune evasion:

(1) In TCGA-SKCM patients, lower expression of miR-25 correlates with better survival **(Figures 1E and S1B)** and enhanced immune cell infiltration **(Figures 3B and 3C)**, consistent with an immunosuppressive role of miR-25.

(2) In our syngeneic mouse tumor models, CRISPR-Cas9–mediated miR-25 knockout enhances tumor sensitivity to anti–PD-1 therapy, further supporting its role in immune resistance **(Figures 1N, 1O and 1P)**.

Taken together, these data suggest that miR-25 may facilitate immune evasion, rather than being simply a marker of tumor cell clearance during therapy.

Line 137: The annotation of miRNA families should be consistent. It is not clear whether the miR-17/92 cluster or the miR-17-5p/20-5p/93-5p/106-5p seed family is referenced here.

Response: We thank the reviewer's attention to this important detail. To improve clarity and consistency, we have revised the description to explicitly refer to the miR-17-92 cluster and its paralogs **(lines 103, 104, 165)**.

Line 139 (Figure 1d): Were pre-miRNAs or mature miRNAs quantified in this experiment? If mature miRNAs were analyzed it should be stated if the -3p or -5p microRNA was quantified.

Response:

In our small RNA-seq analysis, reads were mapped to the mouse genome and annotated based on genomic coordinates defined in the GTF annotation file. This approach quantifies miRNA expression at the gene level, without distinguishing between pri-miRNA, pre-miRNA, or mature miRNA isoforms, nor between the -3p and -5p strands of mature miRNAs. As a result, the reported expression reflects cumulative read coverage across the annotated miRNA locus, rather than strand-specific mature miRNA levels.

According to miRNA biogenesis, only one strand is typically selected as the functional, RISC-loaded miRNA. [17-20] In the case of miR-25, the -3p strand (miR-25-3p) is the dominant and functionally active form, known to mediate translational repression and mRNA degradation.

To validate the expression of the functional strand, we performed RT-qPCR using primers specific to mature miR-25-3p **(Supplemental table 3, sheet "Primers for PCR")** on additional tumor samples **(Figure 1D)**. This confirms that miR-25-3p is the relevant and biologically active form in our study, particularly in the context of anti–PD-1 immunotherapy.

Line 351 (Figure 4f): I found this observation confusing. Why is more Sdc3 mRNA pulled down with IP-Ago2 in the miR-25 KO condition? If Sdc3 is a target of this microRNA, depletion of the microRNA would be expected to reduce the amount of Sdc3 that can be pulled down with Ago2 from cell lysates.

Response:

We agree that our initial explanation was unclear, and we appreciate the opportunity to clarify the data.

At baseline (IFN-γ at 0 h), miR-25 KO cells exhibit a 2.5-fold increase in total Sdc3 mRNA level in whole cell lysates **(Figure 5B, right panel qPCR, compare NTC 0 h and KO 0 h)**, accompanied by a 5-fold increase in Ago2-bound Sdc3 transcripts **(Figure 5A, upper panel qPCR, NTC Ago2 0 h and KO Ago2 0h, grey vs. yellow bars)** suggesting that binding of other endogenous miRNAs with similar seed sequences (e.g., miR-92a) to the Sdc3 3′UTR in the absence of miR-25.

Upon 72 hours of IFN-γ treatment, NTC cells exhibited a marked increase in Ago2-bound Sdc3 **(Figure 5A, upper panel qPCR, NTC Ago2 0 h and NTC Ago2 72h, grey vs. green bars)**, which was coupled with reduced total Sdc3 mRNA **(Figure 5B, right panel qPCR, compare NTC 0 h and NTC 72 h)** and protein levels **(Figure 5B, left panel western blotting, compare NTC 0 and NTC 72 h)**, consistent with miR-25–mediated repression under inflammatory conditions.

In contrast, miR-25 KO cells failed to show any additional increase in Ago2-bound Sdc3 after IFN-γ stimulation **(Figure 5A, upper panel qPCR, KO Ago2 0 h and KO Ago2 72 h, yellow vs. red bars)**, despite a continued rise in WCL Sdc3 mRNA **(Figure 5B, right panel qPCR, compare KO 0 h and KO 72 h)** and protein **(Figure 5B, left panel western blotting, compare KO 0 and KO 72 h)**. This decoupling between total mRNA abundance and Ago2 association supports a loss of IFN-γ–induced, miR-25–dependent repression.

In summary, other endogenous miRNAs may still associate with Ago2-bound Sdc3 transcripts at baseline of miR-25 KO, but they fail to mediate repression under immune-activated conditions IFN-γ stimulation. We have updated the **Results section lines 484 – 503** to reflect this explanation.

In conclusion, while the manuscript is clearly of great interest to the general readership of Nature Communication, the key findings are not supported by the provided evidence and the alternative explanation that the phenotype is caused by either off-target effect of the sgRNAs or by some other artifact cannot be excluded.

Reviewer #2 (Remarks to the Author): with expertise in cancer immunology

In this manuscript, Zhu, et al. attempted to tackle the issue of therapeutic resistance to anti-PD-1 in various forms of cancer (melanoma, colorectal cancer, and triple negative breast cancer). They found that downregulation of miR-25 in tumor cells upon IFN-g treatment in vitro and anti-PD-1 therapy in vivo helped tumor cell escape from immunotherapy. Deletion of miR-25 by CRISPR-Cas9 in tumor cells improved therapeutic efficacy of anti-PD-1 by promoting innate immune sensing through augmented expression of Sdc3, a membrane proteoglycan that can be recognized by innate immunosurveillance. The authors attributed this to the reduction of tumor-associated macrophages (TAMs, even though they gained proinflammatory M1 features upon miR-25 KO and GVAX+anti-PD-1 treatment) and increased Nlrp3 inflammasome formation, the latter of which depended on TAMs, IFN-g release, and miRISC protein Ago2. Overall, this study illustrates that tumor cells turn off full-spectrum immune activation by downregulating miR-25 (and other microRNAs) after sensing IFN-g production upon anti-PD-1 therapy. Some major and minor concerns are as follows:

Major concerns:
1. While miR-25 was downregulated in tumor cells upon anti-PD-1 and IFN-g treatment, restoration/stabilization of miR-25 (but not deletion of miR-25) in tumor cells would be needed to unequivocally link miR-25 downregulation to anti-PD-1 resistance.

Response:

We appreciate the reviewer's suggestion to perform restoration or stabilization of miR-25 to further validate its role. In our study, miR-25 is among the most abundantly expressed miRNAs in tumor cells **(Figures 1C and 1D)**, and its high expression has also been reported in melanoma cell lines and patient samples. [21,22]

As such, non-targeting control (NTC) cells—which retain endogenous miR-25-3p expression—serve as an appropriate physiological reference. These cells partially respond to combination immunotherapy and do not exhibit the immune-stimulatory phenotypes seen in miR-25 KO tumors, supporting the conclusion that loss of miR-25 contributes to enhanced therapy response.

Moreover, Sdc3 expression appears to be regulated through miRISC and may also be influenced by other miRNAs with similar seed sequences, such as miR-92a **(Figures 4N, 5A and 5B)**. Ectopic overexpression of miR-25 may artificially alter this balance, potentially introducing non-physiological or off-target effects.

Therefore, we believe that the combination of genetic knockout and appropriate NTC controls sufficiently demonstrates the tumor-intrinsic role of miR-25 in modulating response to immune checkpoint therapy.

2. With the identification of miR-25 as an underlying mechanism of therapeutic resistance to anti-PD-1, how to target it to bolster therapeutic efficacy of anti-PD-1 was not described.

Response:

Thank you for this important question. While miR-25 plays a clear role in mediating resistance to anti–PD-1 therapy in tumor cells, it potentially has context-dependent functions in other cell types, including lymphocytes. Given this potential for off-target immunomodulatory effects, we opted not to pursue direct miR-25 inhibition as a therapeutic strategy. Instead, as a more targeted approach, we explored disrupting the miR-25 binding site within the 3′ UTR of its downstream effector, Sdc3, using adenine base editing (ABE8e). This experiment, presented in **Figures 5L – 5N**, serves both as a functional validation of the miR-25–Sdc3 regulatory axis and as a proof-of-concept for genome-level therapeutic editing.

Using ABE8e, we achieved ~75% editing efficiency at site A5 and ~30% at site A11 **(Figures S5G and 5L)**, both of which resulted in significant upregulation of Sdc3 mRNA and protein in the presence of IFN-γ **(Figures S5H and 5M)**. These findings demonstrate that disrupting the miR-25 binding site is sufficient to relieve miR-25–mediated repression of Sdc3. Furthermore, we validated the in vivo efficacy of this base editing strategy

and found it comparable to miR-25 knockout in enhancing tumor sensitivity to anti–PD-1 therapy **(Figure 5N).** These results suggest that precise editing of key miRNA–mRNA interactions may offer a promising and more selective therapeutic alternative.

3. Was miR-25 different from other widely studied microRNAs, such as miR-19-92a cluster and miR-155, in terms of their roles in immune evasion?

Response:

We thank the reviewer for this thoughtful point. The roles of microRNAs in tumor immune evasion remain incompletely understood, in part due to the complexity of the tumor microenvironment (TME). The TME is a highly dynamic system composed not only of tumor cells but also infiltrating immune cells, stromal components, and soluble factors—all of which interact and collectively influence tumor progression, metastasis, and therapeutic response. [23] Because miRNAs are expressed across multiple components within the TME, their functional effects are often context-dependent and cell type–specific, making them challenging to study and therapeutically target with precision.

As previously noted in published work, the miR-17-92 cluster exhibits pleiotropic functions: while it promotes tumorigenesis in cancer cells, it also enhances T cell proliferation and survival. [24,25] Similarly, miR-155 has been shown to support tumor immune evasion, yet also plays a beneficial role in the immune system by preventing $CD8^+$ T cell exhaustion and senescence. [26,27] These dual and sometimes opposing roles make therapeutic targeting of these miRNAs complex.

Our study focuses on miR-25, a member of the oncomiR-1 paralogs, in the context of highly progressed, metastatic, and immune-evasive syngeneic mouse tumor models. These tumors can grow in immunocompetent hosts, suggesting selection for intrinsic mechanisms of immune escape. While miR-25 has previously been implicated in promoting tumorigenesis [28] and in hypoxia-induced immunosuppression via repression of cGAS, [29] our study identifies a previously uncharacterized role for miR-25 in mediating tumor immune evasion, which appears to be conditional upon immune activation. Specifically, miR-25 sensitizes tumors to therapy only in the context of immune checkpoint blockade in vivo **(Figures 1H – 1P),** and regulation of its downstream target, Sdc3, occurs only under IFN-γ stimulation in vitro **(Figures 5A – 5D)**. These findings underscore the immune-responsive and context-dependent nature of miR-25 function in cancer cells.

In summary, our findings provide new insight into how tumors may evolve under immune pressure, selecting for miRNA-driven mechanisms of immune escape, even in the setting of a fully functional immune system. We have also updated the **Discussion section lines 566 – 601** to reflect this explanation.

4. While miR-25 deletion in tumor cells greatly increased the infiltration of CD45+ cells, the percentages of macrophages were actually decreased. This significant reduction of macrophages in miR-25 KO tumor cells (further reduced upon GVAX+anti-PD-1: GP) casted the benefit of the doubt on the role of macrophages in the process. Specifically, how to reconcile this macrophage reduction to its involvements in driving Nlrp3 inflammasome formation and innate immunity? It was counterintuitive that reduction of TAM (M1) drove miR-25 deficiency effects (greater response to anti-PD-1, etc.). Furthermore, while deletion of TAM with clodronate liposomes largely abolished miR-25 KO effects, this deletion was not overt in miR-25 KO tumors. Did deletion of macrophages abolish inflammasome formation and potentiation of adaptive immunity in the TME of miR-25 KO tumors?

Response:

We thank the reviewer for this insightful question. In the revised manuscript, we have expanded and clarified our analysis through updated single-cell RNA-seq and flow cytometry validation, with particular focus on macrophage heterogeneity in the tumor microenvironment (TME).

To address the observed reduction in macrophages in miR-25 KO tumors, we interpret this finding in the context of distinct macrophage subpopulations. Our updated scRNA-seq analysis **(Figure 2)** shows a selective

decrease in two M2-like tumor-associated macrophage (TAM) clusters (C19 and C20), which express classic M2 markers such as Thbs1, Fn1, Ctsd, Ctsc, and Chil3. In contrast, the M1-like, antigen-presenting macrophage cluster (C18)—characterized by high expression of MHC II genes including H2-Ob, H2-Eb1, H2-DMb2, H2-DMb1, H2-Ab1, and H2-Aa—was not reduced **(Figure 2D)**. Instead, we observed a significant upregulation of MHC II molecules, particularly H2-Ob and H2-DMb2, in miR-25 KO tumors **(Figure 2E)**.

Furthermore, these M1-like TAMs (C18) also exhibited decreased expression of many immune suppressive genes, including Fcgr1, Lgals3, Lgals1, S100a10, Fgl2, Cd44, Spp1, and Tyrobp (**Figure 2F and Supplemental Table 5, sheet "C18_H2-Aa_TAM")**. This shift in gene expression suggests a polarization of antigen-presenting macrophages away from an M2-like state, driven by miR-25 loss in the tumor.

These findings were validated by flow cytometry in B16 melanoma, which showed decreased total macrophages **(Figures 3D and 3E)** and M2 macrophages **(Figure 3H)** but increased populations of MHC II+ macrophages in both the tumor and spleen **(Figures 3F and 3G)**. As M2-like macrophages typically comprise most of the TAM population, their selective depletion likely explains the overall reduction in macrophage percentages, even though M1-like macrophages are maintained or even enriched.

To directly test the central role of macrophages, we repeated macrophage depletion using clodronate liposomes. This depletion recapitulated the tumor-suppressive effect seen with miR-25 KO and abolished its additive benefit, confirming the critical involvement of macrophages **(Figures 3J and 3K)**. These findings are consistent with prior reports that targeting immunosuppressive macrophages can enhance responses to immune checkpoint therapy. [30,31] However, we also found that macrophage depletion markedly reduced complement infiltration **(Figure 3M)**, suggesting a potential impairment of humoral immunity. This reveals a key caveat: clodronate liposomes non-specifically deplete both M2-like and M1-like macrophages, including those with antigen-presenting capacity, which may inadvertently compromise adaptive immune responses.

In summary, our revised data support a model in which miR-25 KO selectively reduces immunosuppressive M2-like macrophages while preserving or enhancing M1-like, antigen-presenting macrophages, thereby reshaping the TME to favor immune activation. While clodronate-mediated macrophage depletion confirms their central role in miR-25–dependent tumor control, the non-selective nature of this approach likely contributes to the observed effects on innate immune antigen recognition and adaptive immunity, as the reviewer insightfully noted. We also updated **Discussion section lines 603 – 621** to clarify this interpretation.

5. There was a repression of interferon genes in macrophages from miR-25 KO tumors by αPD-1 therapy. Conversely, blocking IFN-g activated Mac in miR-25 KO tumors. These seemingly contradictory observations needed to be reconciled.

Response:

We thank the reviewer for carefully noting this apparent discrepancy in interferon (IFN) gene expression patterns. In our dataset, we observed a downregulation of type I IFN–associated genes—including Ifitm2, Ifitm3, Isg15, Ifit2, among others—as well as decreased expression of M2 polarization–related genes in M1-like antigen-presenting macrophages (cluster C18) **(Figure 2E)**. Type I interferons (e.g., Ifit2, Ifit3, Ifitm2, and Ifitm3) are classically known for their antiviral roles. [32,33] Although IFN-I has been used in solid tumor treatment since the 1980s, its clinical success has been limited. Recent studies suggest IFN-I has context-dependent effects in the tumor microenvironment (TME), and in some cases, may even support immunosuppressive programs. For example, IFN-I–treated monocytes have been shown to induce Arg1 expression (encoding arginase-1), which promotes a tumor-supportive phenotype in TAMs. [34]

Based on these findings, we hypothesize that in our system, the downregulation of type I IFN genes in macrophages may reflect a shift away from suppressive, M2-like functions toward a more pro-inflammatory, antigen-presenting M1-like state in miR-25 KO tumors.

In contrast, type II IFN (IFN-γ) plays a distinct and central role in our model by mediating tumor intrinsic resistance to ICT. [35,36] Anti–PD-1 therapy enhances $CD8^+$ T cell–mediated cytotoxicity and elevates IFN-γ production, which promotes tumor cell killing. [37-39] We found that miR-25 KO sensitize tumor cells to immune pressure in an IFN-γ–dependent manner **(Figure 4A)**, a finding further supported by IFN-γ depletion experiments. IFN-γ depletion reverted the therapeutic effects of miR-25 KO, including suppression of tumor

growth, activation of macrophages, and complement infiltration **(Figures 4B – 4E)**. These effects were accompanied by reverted expression of Sdc3 in vitro without IFN-γ **(Figures 5B, 5E, and S5A)**, supporting our model that miR-25 represses Sdc3 in an IFN-γ-dependent context, thereby facilitating immune recognition and clearance of tumor cells.

6. Ago2 was unchanged in miR-25 and miR17-92a deficient tumor cells, although the authors showed that Ago2/miR-25 DKO phenotypes were comparable to those in Ago2 KO alone (Figure 4c and s4d). This is sort of expected as without the essential component of RISC complex, miRNAs would not work. That said, Ago KO only partially abolished the phenotype of miR-25 KO, suggesting other mechanisms exist.

Response:

We thank the reviewer for their thoughtful question. Our goal in knocking out Ago2 was to determine whether miR-25 exerts its function through canonical RISC-mediated mechanisms. As expected, we found that the phenotypes of Ago2/miR-25 double knockout (DKO) tumors largely mirrored those observed with Ago2 knockout alone, both in vitro and in vivo **(Figures 4F– 4K)**, supporting the conclusion that miR-25 acts primarily through Ago2-dependent RISC activity.

Specifically, both Ago2 KO and Ago2/miR-25 DKO resulted in comparable decreases in total tumor-associated macrophages (TAMs), increased MHC II+ TAMs **(Figure 4J)**, and enhanced complement infiltration in tumors **(Figure 4K)**. However, tumor volumes and weights in the Ago2/miR-25 DKO group were not significantly different from NTC controls **(Figures 4H and 4I)**, suggesting that the tumor-suppressive phenotype observed with miR-25 KO alone was lost when Ago2 was also deleted.

Interestingly, Ago2 KO alone increased total macrophage numbers in the spleen—a phenotype reversed in the Ago2/miR-25 DKO group **(Figure S4H, left)**. Moreover, neither Ago2 KO nor Ago2/miR-25 DKO induced splenic MHC II+ macrophages to the extent seen in miR-25 KO alone **(Figure S4H, right)**, indicating that RISC inactivation only partially phenocopies miR-25 loss. These results suggest that Ago2, or potentially other microRNAs, may also play important roles in regulating tumor immune responses to checkpoint blockade.

As the reviewer insightfully noted, these findings imply that while miR-25 function is largely dependent on canonical RISC activity, Ago2 or additional microRNAs may contribute to the broader immune modulation observed in our model. We have updated the **Discussion section lines 584 – 593** to reflect this interpretation.

7. Bulk RNA-Seq and scRNA-seq should be done using the B16-F16 (the major tumor model); otherwise, it would be hard to connect, given that different treatments were used for B16-F10 (GVAX+anti-PD-1) and MC38 (only anti-PD-1). By the same token, Fig. 4d RNA seq analysis should use in vivo RNA-seq datasets, as mixing in vivo RNA-seq data with in vitro RNA-seq data from miR-25 KO tumor cells treated with IFN-g or not introduced too many confounding factors.

Response:

1. Mouse model discrepancy

We thank the reviewer for this insightful comment regarding the use of different tumor models and treatment regimens. We fully acknowledge that B16 (melanoma) and MC38 (colon carcinoma) are distinct models and were treated with different immunotherapy regimens—GVAX plus anti–PD-1 for B16, and anti–PD-1 alone for MC38.

We initially attempted single-cell RNA-seq (scRNA-seq) on B16 tumors; however, we encountered technical limitations due to poor viability. After dead cell removal, the percentage of live cells consistently fell below the quality control threshold of 90%, preventing us from generating reliable scRNA-seq data in this model. In contrast, MC38 tumors yielded high-quality viable cells, enabling us to successfully perform scRNA-seq.

Despite these differences, we observed consistent functional phenotypes across all three syngeneic tumor models—B16, MC38, and 4T1. Specifically, miR-25 knockout did not impact in vitro proliferation **(Figures 1H, 1I and 1J)**, but significantly enhanced response to anti–PD-1 therapy in vivo in all models **(Figures 1N – 1P)**.

Furthermore, the miR-25–dependent regulation of Sdc3 in response to IFN-γ was validated in all three tumor cell lines in vitro **(Figures 5B – 5D)**, supporting the broader relevance of this mechanism across epithelial cancers.

To improve clarity and emphasize logical coherence across models, we have restructured the Results section as follows:

Figure 1: Begins with phenotypic analysis of miR-25 KO across all three tumor models.

Figure 2: Presents MC38-based scRNA-seq analysis, which forms the mechanistic foundation of the study.

Figure 3: Aligns B16 tumor data and TCGA-SKCM human melanoma transcriptomes with the MC38 scRNA-seq findings.

Figures 4–5: Focus on in vivo functional validation of the mechanism (macrophage depletion, IFN-γ depletion, Ago2 KO, and Sdc3 targeting), all performed in the B16 model. The core regulatory axis (miR-25–Sdc3–IFN-γ) was also tested in MC38 and 4T1 cells in vitro to confirm generalizability.

This restructuring highlights mechanistic consistency across different tumor models and emphasizes key phenotypes observed, including reduced total macrophage infiltration, increased MHC II+ macrophage polarization, and enhanced complement activation.

2. Rationale for using in vitro RNA-seq

Our primary goal in this analysis was to isolate tumor cell–intrinsic effects of miR-25 knockout, independent of the confounding influence of immune and stromal cells within the tumor microenvironment (TME). The TME is a complex and heterogeneous ecosystem composed of multiple interacting cell types, whose collective gene expression can obscure tumor-specific regulatory effects. [23]

Our scRNA-seq data revealed that Sdc3 is expressed not only in tumor cells but also in various immune cell populations **(Figures S6A and S6C)**, making it difficult to attribute gene expression changes specifically to tumor cells in bulk in vivo datasets.

In addition, Sdc3 expression was not significantly increased in miR-25 KO tumor clusters at day 17 when performing scRNA-seq **(Figure S6B)**, likely due to the delayed sampling timepoint during treatment. Our in vitro data, however, showed that Sdc3 is stabilized within 24–48 hours of IFN-γ stimulation **(Figures 5C and S5B)**, suggesting that this regulation is time-dependent and may be most pronounced during early immune activation.

Therefore, we reasoned that an in vitro RNA-seq approach—using purified B16 tumor cells treated with or without IFN-γ—would provide a clearer window into direct, tumor cell–specific transcriptional responses under immune-activated conditions that mimic anti–PD-1–mediated inflammation **(Figure 4L)**.

That said, we fully agree that in vivo validation remains essential. We addressed this by target validation with in vivo functional experiments targeting Sdc3 via gene deletion or base editing **(Figures 5E – 5N)**, which further support the biological relevance of the miR-25–Sdc3 regulatory axis in the context of immune checkpoint therapy. We have also updated the **Discussion section from lines 676 – 704** reflect this interpretation.

Minor concerns:
1. Please clearly specify GVAX.

Response:

We thank the reviewer for this point and have now clarified the definition of GVAX in the revised manuscript. GVAX refers to irradiated B16 melanoma cells genetically engineered to secrete granulocyte-macrophage colony-stimulating factor (GM-CSF). This cell-based vaccine is used to stimulate dendritic cell recruitment and maturation, thereby eliciting an anti-tumor immune response against B16 melanoma. [40] We have added the reference work in **Results section line 154** and this clarification to the **Methods section from line 818 to 822**.

2. Line 449-450—"Our data indicates that anti-PD-1 can increase TAM infiltration of tumors, an activity regulated by tumor cells to achieve immunosenescence." But there were no data supporting this; instead, they showed that anti-PD-1 decreased TAM infiltration.

Response:

We thank the reviewer for pointing out this inconsistency. We have removed this statement from the Discussion section, as our data do not support the claim that anti–PD-1 therapy increases TAM infiltration.

3. Please clarify "we found that IFN-γ depletion starting at day 8 also abolished miR-25-KO effects and increased the number of MHCII+ macrophages in spleen, indicating that IFN-γ is indispensable for TAM activation in the TME" (Line 456-458), as this writing suggested that IFN-g actually suppressed TAM activation.

Response:

We have repeated the IFN-γ depletion experiments, as shown in **Figures 4B – 4E**. Our updated data confirm that IFN-γ is essential for mediating the effects of miR-25 knockout. Specifically, depletion of IFN-γ reverted the tumor-suppressive phenotype observed in miR-25 KO tumors **(Figures 4B and 4C)**, altered total macrophage infiltration and MHC II+ macrophage activation in both the tumor and spleen **(Figures 4D and S4F)**, and reduced complement C3 activation **(Figure 4E)**.

These findings suggest that IFN-γ plays a complex and context-dependent role in shaping the tumor microenvironment. While IFN-γ is primarily produced by effector $CD8^{+}$ T cells and is generally considered pro-inflammatory, our data indicate that in this setting, it may paradoxically contribute to immune suppression. Notably, IFN-γ depletion significantly reduced overall macrophage numbers but increased the proportion of MHC II+ macrophages in both tumor and spleen **(Figures 4D and S4F)**, suggesting that IFN-γ may suppress innate immune activation or macrophage antigen-presenting capacity under certain conditions.

We hope these revised figures and text provide a clearer explanation of the role of IFN-γ–induced resistance in our model.

4. Please explain "in the presence of IFN-γ, TAM recognition of tumor cell changes depending on miR-25 expression in tumor cells" (Line 461). Did the authors mean to say that TAM recognition of tumor cells needed miR-25 expression?

Response:

We thank the reviewer for pointing out the ambiguity in our previous statement.

In the updated text, we explain that miR-25 influences the expression of its downstream target, Sdc3, on the tumor cell surface, particularly under IFN-γ stimulation. This modulation of Sdc3 expression may, in turn, alter how TAMs interact with or recognize tumor cells in the tumor microenvironment. Therefore, we did not mean to suggest that TAM recognition requires miR-25 expression, but rather that miR-25 status in tumor cells modifies surface signaling (via Sdc3), which may influence TAM activation or polarization, especially in the context of IFN-γ–driven immune responses. We also updated **Discussion section lines 593 – 601** to clarify this interpretation.

5. This is an overstatement "we show that miR-25-KO induces Sdc3 expression on tumor cells, converting immune 'cold' to 'hot' tumors based on Nlrp3 inflammasome formation in seen TAMs after ICB (Figure 6)." "in seen" appeared to be a typo.

Response:

We thank the reviewer for pointing this out. We have carefully revised the relevant sentence and updated all associated figures. We now emphasize that miR-25 deficiency in tumor cells enhances both innate and

humoral immunity by promoting MHC class II expression in antigen-presenting, M1-like macrophages, and activating the classical complement cascade in inflammatory cancer-associated fibroblasts (iCAFs)—features that are well-recognized hallmarks of an immunologically active (or “hot”) tumor microenvironment. [41,42]

To clarify the biological significance, literature define "immune cold" tumors as those with low T cell infiltration, poor antigen presentation, and an overall immunosuppressive microenvironment—features that are often associated with resistance to ICT. In contrast, "immune hot" tumors exhibit high levels of immune cell infiltration, robust antigen presentation, and pro-inflammatory signaling, rendering them more responsive to ICT. [41,42] Further, we summarize these factors in synopsis figure.

In our study, miR-25–deficient tumors exhibit several hallmark features of immune hot tumors:

(1) Increased T cell infiltration **(Figure 2C)**,

(2) Upregulated MHC II expression in M1-like macrophages, indicating enhanced antigen presentation **(Figure 2E)**,

(3) Robust humoral immune activity through classical complement activation in inflammatory CAFs **(Figure 2H)**.

Taken together, these findings strongly align with the definition of immune "hot" tumors. Therefore, we believe it is justified to state that miR-25 deficiency reprograms the tumor microenvironment from immune-resistant "cold" to immune-responsive "hot", offering a mechanistically grounded and potentially actionable strategy to enhance ICT efficacy.

6. Please double check the text to remove typos and grammatical errors.

Response: Thank you for carefully reading the manuscript. We have thoroughly reviewed the text and have improved grammar, clarity, and overall readability throughout the manuscript.

Reviewer #3 (Remarks to the Author): with expertise in melanoma, miRNAs

In this study, Zhu et al show that microRNAs miR-25 and miR-17-92 members modulate innate immunity and response to anti-PD1 immunotherapy. Using B16F10 as a murine model of melanoma, the authors find miR-25 and the miR-19/72 cluster downregulated in tumor cells upon GVAX and anti-PD1 treatment. CRISPR/Cas9 knockout of miR-25 combined with GVAX+PD1 or PD1 treatment in B16F10 melanoma, MC38 colorectal carcinoma, and 4T1 breast cancer significantly reduced tumor volume and enhanced survival relative to GVAX+PD1 or PD1 immunotherapy, respectively. miR-25 knockout alone did not alter tumor growth in vivo, supporting its specificity in regulating immunotherapy response. Similar effects were found by targeting components of miR-17-92. These results highlight the potential role suppressing miRNA can have in enhancing immunotherapy treatments.
Flow cytometry plus immunohistochemistry of B16F10 tumors demonstrated increased infiltration of CD45+ immune cells in the miR-25 KO GP group, an increase of cytotoxic Granzyme B CD8+ T cells, a reduction of immune-suppressive FOXP3+ CD4+ Tregs and CD206+ macrophages. Bulk RNA-sequencing and immunohistochemistry of miR-25 KO GP and miR-106a-KO tumors revealed upregulation of complement genes like C3 and innate immune pathways involving the pyroptotic phagosome, ultimately contributing to a pro-inflammatory microenvironment in the miR-25 KO GP group. Clodronate liposome injections abolished the reduced tumor volume observed in the miR-25 KO GP group, suggesting macrophages mediate miR-25's immunotherapeutic effects.
Single cell RNA-seq of MC38 cells showed that upon miR-25 inhibition, macrophage depletion plus polarization, CAF polarization, and T cell activation contributes to the immunotherapy response. Depletion of IFN-y abolished the reduced tumor growth observed upon miR-25 KO GP treatment in B16F10 cells and increased the number of MHC-II expressing M2 macrophages in the spleen, indicating M1 macrophage polarization following miR-25 KO requires IFN-y in the TME. Overlapping three B16F10 gene expression datasets with an algorithm for predicting miR-25 target genes uncovered Sdc3 as a downstream target of miR-25. miR-25 base-pairs with Sdc3, repressing Sdc3 expression through an interaction with Ago2 for RISC-dependent degradation in the presence of IFN-y. Additionally, miR-25 knockout increases transcript and protein levels of Sdc3 in B16F10, MC38, and 4T1 cells. This suggests that while IFN-y enhances degradation of Sdc3, higher basal mRNA and protein levels of Sdc3 due to miR-25 knockout enables cancer cells to maintain higher overall Sdc3 levels.
Small RNA sequencing of TCGA metastatic melanoma patients showed a ~2-fold difference in miR-25 levels, with miR-25 low tumors exhibiting increased phagosome pathway activity and complement plus IL6-JAK-STAT signaling. miR-25-low patients had reduced amounts of undifferentiated macrophages and higher delta gamma T cells, CD8 T cells, and CD4 memory activated T cells. mir-25 and miR-17/92 expression negatively correlated with innate immune genes related to complement activity. Furthermore, responders to PD1 immunotherapy showed lower levels of miR-25 and negatively correlated with complement gene expression.

Major issues
It is unclear how differentially expressed miRNAs in B16 tumors treated with GVAX and anti-PD1 vs control were prioritized for further analysis and why specifically they followed up on miR-25. Based on the volcano plot, these miRNA don't seem the most differentially expressed.

There are notable differences between the FACS and IF analyses of tumors, with the FACS showing reduced macrophage infiltration in the miR-25 KO mice and the IF showing similar numbers. FACS and IF analyses were only conducted in the B16 model. They should be repeated in at least another model. Similarly, there seem to be differences with the MC38 model, which by scRNAseq did show a reduction of M2 macrophages and an increased in gamma-delta T cells. Given the differences observed across models and techniques, it seems necessary to compare side-by-side the results by FACS and IF in B16 and MC38 cells. Finally, the mechanistic findings (miR-25 modulation of Sdc3) appear disconnected from the phenotypic effects on complement, IFN levels, macrophage polarization, etc. Authors should do a better job at linking mechanism and immune effects by showing that Sdc3 modulation by miR-25 mediates (at least partially) those effects.

In sum, while the manuscript contains many interesting findings, some of the conclusions don't seem sufficiently supported by data obtained in more than one model. The phenotypic effects of miR-25 KO in the tumor immune repertoire seem context/model dependent and it is hard to draw strong conclusions. In addition,

the mechanistic connection between miR-25 and a single target (Sdc3) seems also a stretch, and should be further supported by clinical data.

Response:

We thank the reviewer for this constructive and thoughtful critique. In response, we have significantly revised the manuscript by including new experiments, bioinformatic and functional analyses, improving clarity, enhancing mechanistic linkage, and providing stronger cross-model support for our conclusions.

(1) Selection of miR-25:

The rationale for focusing on miR-25 has been updated. We reanalyzed the small RNA-seq data. As part of this reanalysis, we also refined our criteria across all RNA-seq analyses, applying a more stringent threshold: $|\log_2 FC| > 1$ and adjusted p-value (padj) < 0.05. Notably, miR-25 was the only miRNA significantly and consistently downregulated in both the Combination (GVAX + anti-PD1) vs. Control and Combination vs. GVAX comparisons **(Figure 1C)**. Among all significantly downregulated miRNAs, miR-25 also exhibited the highest expression level, with a base mean of 17,315.96 **(Figure 1D; Supplemental Table 1, sheet "Comb_vs_GVAX")**. To validate the observed downregulation, we performed RT-qPCR on additional mouse tumor samples. These results confirmed that miR-25 is significantly decreased in tumors treated with combination therapy, particularly in those that responded to the treatment **(Figure 1D)**.

(2) Model Comparison and Phenotypic Consistency:

We fully acknowledge that B16 (melanoma) and MC38 (colon carcinoma) are distinct models and were treated with different immunotherapy regimens—GVAX plus anti–PD-1 for B16, and anti–PD-1 alone for MC38.

We initially attempted single-cell RNA-seq (scRNA-seq) on B16 tumors; however, we encountered technical limitations due to poor viability. After dead cell removal, the percentage of live cells consistently fell below the quality control threshold of 90%, preventing us from generating reliable scRNA-seq data in this model. In contrast, MC38 tumors yielded high-quality viable cells, enabling us to successfully perform scRNA-seq.

Despite these differences, we observed consistent functional phenotypes across all three syngeneic tumor models—B16, MC38, and 4T1. Specifically, miR-25 knockout did not impact in vitro proliferation **(Figures 1H, 1I and 1J)**, but significantly enhanced response to anti–PD-1 therapy in vivo in all models **(Figures 1N – 1P)**. Furthermore, the miR-25–dependent regulation of Sdc3 in response to IFN-γ was validated in all three tumor cell lines in vitro **(Figures 5B – D)**, supporting the broader relevance of this mechanism across epithelial cancers.

To improve clarity and emphasize logical coherence across models, we have restructured the Results section as follows:

Figure 1: Begins with phenotypic analysis of miR-25 KO across all three tumor models.

Figure 2: Presents MC38-based scRNA-seq analysis, which forms the mechanistic foundation of the study.

Figure 3: Aligns B16 tumor data and TCGA-SKCM human melanoma transcriptomes with the MC38 scRNA-seq findings.

Figures 4–5: Focus on in vivo functional validation of the mechanism (macrophage depletion, IFN-γ depletion, Ago2 KO, and Sdc3 targeting), all performed in the B16 model. The core regulatory axis (miR-25–Sdc3–IFN-γ) was also tested in MC38 and 4T1 cells in vitro to confirm generalizability.

This restructuring highlights mechanistic consistency across different tumor models and emphasizes key phenotypes observed, including reduced total macrophage infiltration, increased MHC II+ macrophage polarization, and enhanced complement activation.

(3) Bridging Mechanism and Immune Effects:

We appreciate the reviewer's concern that the connection between miR-25/Sdc3 regulation and the observed immune phenotypes was not sufficiently articulated in the original manuscript. We have now addressed this directly through several key updates:

A. Demonstrating that Sdc3 regulation occurs downstream of IFN-γ, a cytokine enriched during anti–PD-1 therapy **(Figures 4 – 5)**

B. Showing that precise disruption of the miR-25 binding site in Sdc3's 3′UTR using ABE8e base editing mimics miR-25 KO and overcomes ICT resistance **(Figures 5L – 5N)**

C. Linking these molecular changes to functional immune responses, including macrophage activation and complement induction **(Figures 5G – 5K)**

D. Validating clinical relevance through analysis of TCGA-SKCM transcriptomic data, which further supports the conservation and translational significance of the miR-25 in human melanoma **(Figures 3B and 3C)**

(4) Clinical relevance of Sdc3 and immune response:

Please see response to **specific point 23** below.

Specific points that should be addressed:
Figure 1
1. Despite its spontaneous occurrence, B16F10 cells do not harbor clinically relevant melanoma mutations in the MAPK pathway or tumor suppressor genes. More relevant melanoma immunotherapy models are currently available and should be used to confirm the ability of miR-25 KO to enhance response to immune-therapy.

Response:

We thank the reviewer for this important comment. We fully acknowledge that the B16F10 melanoma model lacks common MAPK pathway mutations (e.g., BRAF or NRAS) found in human melanoma, and we agree that no single model can fully recapitulate the genetic and immunological landscape of human disease.

However, our rationale for choosing B16F10 was based on its well-established use as a highly immune-resistant and poorly immunogenic ("cold") tumor model, particularly in the context of ICT resistance. Specifically:

(1) B16F10 tumors are characterized by **low baseline T cell infiltration**, **high PD-L1 expression**, and **poor response to anti–PD-1/PD-L1 therapies**, mirroring features of ICB-refractory tumors in patients. [12,43]

(2) The B16F10 model has been foundational in identifying immune evasion pathways, testing combination immunotherapies, and modeling tumor–immune cell interactions. [44-48]

In our study, the immune-resistant nature of B16F10 made it an ideal model to test whether miR-25 knockout could reprogram the tumor microenvironment and overcome ICT resistance. Notably, we complemented our findings with two additional models (MC38 and 4T1) and observed consistent miR-25–dependent immune phenotypes across tumor types, further supporting the generalizability of our conclusions. While we recognize the value of using newer genetically engineered melanoma models, we believe that the B16F10 model remains a rigorous and widely accepted platform for preclinical ICT resistance research.

2. Despite some clinical indications that GVAX treatment could positively modulate immunotherapy, the apparent differences in PD1 response as observed in B16F10 are clinically rare, indicating GVAX may not be a valid model for making broad claims regarding immunotherapy.

Response:

We thank the reviewer for this valuable comment. While we agree that GVAX is not currently a standard clinical treatment, its use as a preclinical immune stimulant has been well established, particularly in the context of poorly immunogenic tumors such as B16F10 melanoma.

Our rationale for using GVAX (irradiated, GM-CSF–secreting tumor cells) in this study was not to model a clinically approved therapy, but rather to provide a tumor-specific, antigen-presenting cell–recruiting stimulus capable of eliciting immune pressure in an otherwise immune "cold" tumor model. [40] B16F10 tumors are notoriously resistant to ICT alone due to low T cell infiltration and antigen presentation. GVAX serves as a well-established immune-priming strategy that enhances dendritic cell recruitment, cross-presentation, and tumor-specific T cell activation, thereby allowing for a more rigorous test of tumor-intrinsic immune evasion mechanisms, [46,49,50] such as those regulated by miR-25.

Importantly, our conclusions do not rely solely on the GVAX-based model. We also demonstrated consistent effects of miR-25 knockout in GVAX-independent models, including MC38 (colon) and 4T1 (breast) cancer models, both of which respond to anti–PD-1 monotherapy. Together, these results support a GVAX-independent, tumor-intrinsic role for miR-25 in modulating ICT resistance across multiple epithelial tumor types.

3. In their small RNA sequencing analyses, the authors compared the GP (GVAX+PD1) group to an untreated control instead of the GVAX group. It is unclear if miR-25 and miR-19/72 cluster members are significantly downregulated in comparison to the GVAX group, therefore the authors can only make claims in regard to immunotherapy in the context of GVAX treatment and not solely immunotherapy, which is far less clinically relevant due to the clinically unproven benefit of GVAX.
a. miR-19 is the only member of its miRNA cluster whose fold change is halved while also achieving a significant p-value.
b. It is unclear whether the authors profiled any other hits that had a more meaningful fold change and significant p-value, to validate their screen.

Response:

We thank the reviewer for raising this important point. In response, we have re-analyzed our small RNA-seq dataset to include both Combination (GVAX + anti–PD-1) vs. Control (untreated) and Combination vs. GVAX-only comparisons, as now shown in **Figure 1C** of the revised manuscript. As part of this reanalysis, we also refined our criteria across all RNA-seq analyses, applying a more stringent threshold: $|\log_2 FC| > 1$ and adjusted p-value (padj) < 0.05. Our updated analysis confirms that while multiple members of the miR-17-92 cluster and its paralogs are significantly downregulated in the Combination vs. Control comparison, only miR-25 is significantly downregulated in both comparisons. In contrast, miR-19a, though it demonstrated a significant adjusted p-value in one comparison, did not meet significance in both comparisons and was therefore deprioritized **(Figure 1C)**.

4. Colorectal carcinoma tumors are typically responsive to immunotherapy if they are mismatch repair–deficient (dMMR) or possess microsatellite instability-high (MSI-H). MC38 cells are proficient in mismatch repair but respond to immunotherapy, suggesting they are poor models that do not accurately recapitulate colorectal carcinoma as seen in patients.

Response:

We thank the reviewer for this insightful comment. We fully agree the reviewer's point of colorectal carcinoma. While the MC38 cell line is mismatch repair–proficient (pMMR) and microsatellite stable (MSS), we selected this model intentionally because it remains one of the most widely used and well-characterized syngeneic tumor models for studying immune checkpoint therapy (ICT) response and tumor-intrinsic immune evasion.

MC38 tumors grow in fully immunocompetent mice, retain moderate immunogenicity, and respond to anti–PD-1/PD-L1 therapies, making them a well-established model for investigating the mechanisms underlying immunotherapy response. Notably, many recently published studies have used MC38 to uncover key pathways involved in immune resistance and therapeutic efficacy. [51-53]

In this study, we used MC38 in parallel with B16 (melanoma) and 4T1 (triple-negative breast cancer) to capture common, conserved immune resistance mechanisms across epithelial tumor types. Notably, miR-25 deficiency improved ICT efficacy in all three models, supporting the broader relevance of our findings. Future studies can validate our findings in other clinically relevant models.

5. Injections of MC38 and 4T1 were not orthotopic but injected into the flank of mice. Figures 1f-h are missing error bars for the red lines. Tumor weight at endpoint should be shown to orthogonally validate a difference in tumor size between groups.

Response:

We thank the reviewer for these valuable comments.

(1) Subcutaneous injection of the tumors

All three syngeneic tumor models used in our study—B16 (melanoma), MC38 (colon), and 4T1 (breast)—were injected subcutaneously into the flank of immunocompetent mice. While we acknowledge that orthotopic implantation offers a more anatomically relevant microenvironment, particularly for studying local invasion and metastasis, subcutaneous models remain widely used and well-established in preclinical immunotherapy research. They offer key advantages such as reproducible tumor growth, ease of tumor measurement, and consistent treatment delivery. Given that our primary goal was to investigate tumor-intrinsic mechanisms of immunotherapy resistance, subcutaneous models provided an appropriate and tractable platform for comparative analysis across tumor types. We have also provided recent published papers using MC38/4T1 injection on mice flanks. [51-53]

(2) Error bars in Figure 1N, 1O and 1P (original Figure 1f-h)

We appreciate the reviewer's observation regarding the error bars, particularly for the red line (miR-25 KOs + Combination) in **Figure 1N**. The small error bars in this group reflect the consistent and robust tumor suppression observed across replicates, as also shown by individual tumor growth curves **(Figure 1K, right)**. Nonetheless, we have re-verified the raw data and re-plotted the figures to ensure accurate error bar representation.

(3) Tumor weight

We repeated all in vivo functional validations and have now included tumor weight at endpoint **(Figures 3K, 4C, 4I, and 5H)**. We hope these revisions address the reviewer's concerns and enhance the overall clarity and rigor of our data presentation.

6. The authors demonstrated significant reduction of miR-25 level with each of three sgRNAs used individually, but chose to combine all three guides in vivo, increasing the likelihood of off-target effects. The sequence of the non-targeting guide RNA (sgNTC) used is unknown.

Response:

We appreciate the reviewer for raising this important point. We have now included the full list of all sgRNAs, including the non-targeting control (sgNTC), in the revised **Supplemental Table 3, sheet "Primers for sgRNAs"**.

To clarify the in vivo experimental design, we have updated **Figures 1H – 1P**. In our in vivo experiments, each sgRNA was tested individually in a separate mouse, and all three sgRNAs demonstrated comparable reductions in tumor growth compared to sgNTCs when combined with ICT **(Figures 1K, 1L, and 1M)**. These consistent results across three independently designed guides reduce the likelihood that our findings are due to off-target effects from any single sgRNA. Because the uniform therapeutic effect across all three sgRNAs, we combined the data from all miR-25 KO-treated mice into a single group (miR-25 KOs + Comb) or (miR-25 KOs + αPD-1) for downstream analysis and presentation **(Figures 1N, 1O and 1P)**. This approach improves clarity and statistical power while still reflecting the reproducibility of the phenotype across independently designed guides. We also updated **Result section lines 203 – 218** to clarify this sgRNA usage,

7. It is unclear if deletion of miR-17-92a family members in B16F10 altered proliferation of tumor cells, as that could explain the reduced tumor volume. Proliferation effects should be shown.

Response:

We thank the reviewer for raising this important point. In our updated analysis, we found that other members of the miR-17-92a family were not significantly downregulated when comparing combination therapy (GVAX + anti–PD-1) to GVAX monotherapy **(Figure 1C)**. As a result, we have decided to narrow the focus of this study exclusively to miR-25, which is the only member consistently and significantly downregulated across both comparisons and validated across models.

Accordingly, we have removed the experiments involving deletion of other miR-17-92a family members from the current manuscript. The potential roles of other family members may be addressed in a future, dedicated investigation.

Figure 2
8. The voltage settings and gating strategy in S2a and S2b are cutoff and thus may not accurately capture all CD45+ cells. Additionally, the Granzyme B, FOXP3, and CD69 gating is lenient, defining positive cells with a signal strength barely above negative.

Response:

Thank you for pointing out this important technical issue. In the revised manuscript, we have refined our flow cytometry analysis to focus specifically on macrophage populations, which represent the most relevant immune cell type based on our scRNA-seq and functional data.

To improve accuracy and clarity, we have:

(1) Updated our gating strategy to identify macrophages as live $CD45^{+}$ $Ly6C^{-}$ $Ly6G^{-}$ $CD11b^{+}$ $F4/80^{+}$ cells **(Figure S3C)**,

(2) Included additional functional markers such as MHC II, CD206, and CD86 **(Figure S3D)**, and

(3) Validated all gates using FMO (fluorescence minus one) controls to ensure rigor and reproducibility **(Figures S3C and S3D)**.

In response to the reviewer's concern regarding lenient gating of markers such as Granzyme B, FOXP3, and CD69, we have removed these markers from our updated analysis. Based on our transcriptomic and in vivo data, T cell and B cell effector markers were not significantly altered in miR-25–deficient tumors, and therefore were not central to our mechanistic focus. We believe these revisions enhance the technical robustness of our flow cytometry experiments and improve alignment with the central findings of the study.

9. RNA-sequencing was performed on unsorted tumors, therefore it is unclear from which cell population the changes in innate immune signaling are coming and whether it is a feature of multiple cells and cell types or driven by a single cell population exhibiting much higher levels of immune genes/pathways. It is also unclear if these upregulated immune pathways are observed solely due to the presence of more CD45+ cells in the tumor. Some of the gene expression differences in Fig. 2g appear to be inconsistently high in one of the three miR-25 KO GP samples (Cfp, Tlr9, C1rl, Cfd, C3, Serping1, C1s1, Clu, Cfh).

Response:

(1) Cell population-specific changes

To directly address this concern, we have restructured the manuscript to place greater emphasis on scRNA-seq analysis using the MC38 model, which forms the basis for our mechanistic insights **(Figure 2)**.

Our scRNA-seq analysis clearly shows that classical complement pathway genes—including C1s1, C3, C4b, and Cfh **(Figure 2H)**—are primarily expressed by fibroblasts (C30), as indicated by strong expression of canonical fibroblast markers such as Col1a1 and Col1a2 **(Figure S2C)**. This is accompanied by a marked upregulation of inflammatory CAF (iCAF) markers and a concomitant reduction in myofibroblastic CAF (myCAF) markers, suggesting a shift toward a pro-inflammatory CAF phenotype in miR-25–deficient tumors undergoing immune ICT.

Importantly, these complement-related gene changes are not attributable to increased immune infiltration (e.g., CD45+ cells), but rather reflect a tumor-intrinsic and stroma-intrinsic remodeling of the TME. This cell-type–specific insight strengthens our conclusion that miR-25 regulates immune evasion not just through immune cell recruitment, but also through modulation of stromal fibroblast function.

(2) Variability in innate immune gene expression among miR-25 KO GP tumors

We acknowledge the reviewer's concern about variability in innate immune gene expression across miR-25 KO GP samples. Inter-sample heterogeneity is an inherent feature of in vivo tumor models, particularly within immune-modulated settings. To further clarify this point and align the B16 model with our MC38 scRNA-seq findings, we conducted additional analyses, including immunohistochemistry for complement component C3 and flow cytometry for macrophage activation markers.

We have included the original B16 bulk RNA-seq dataset to **Supplemental Figure S2A**, where it is used to illustrate general innate immune activation rather than to draw mechanistic conclusions. The full differential expression results are available in **Supplemental Table 8, sheet: "DEseq_bulk_B16_invivo."**

10. F4/80 can also be a marker of monocytes and eosinophils. It is unclear why authors suggest C3+ cells do not interact with tumor cells.

Response:

(1) Macrophage gating strategy

In our revised gating panel ($CD45^+$ $Ly6C^-$ $Ly6G^-$ $CD11b^+$ $F4/80^+$),

A. Ly6C is used to **exclude monocytes**,

B. Ly6G to **exclude neutrophils**, and

C. The **Combination of CD11b and F4/80 identifies macrophages**.

We believe this co-expression pattern within a $Ly6C^-$ $Ly6G^-$ background offers a reliable and robust approach for distinguishing macrophages from other myeloid populations.

Furthermore, our scRNA-seq analysis independently identified consistent reductions in macrophage populations using established markers such as Fcgr1, Lyz2, and Ms4a4c, reinforcing the validity of both our flow cytometry and single-cell transcriptomic data for macrophage identification.

(2) $C3^+$ cells and tumor interaction

Our new CellChat analysis **(Figure S2H)** suggests that complement-mediated communication—specifically involving C3—is largely restricted to interactions between cancer-associated fibroblasts (CAFs) and tumor-associated macrophages (TAMs). While we did not observe a dominant direct interaction between $C3^+$ cells and tumor cells in this analysis, we identified strong expression of inflammatory CAF (iCAF) markers such as Cxcl12, Cxcl13, and Cxcl14, which have been implicated in anti-tumor immune responses. Therefore, we no longer conclude that $C3^+$ cells do not interact with tumor cells and have removed the corresponding sentence from the main text.

11. There does not appear to be a significant difference in tumor mass between the miR-25 KO GP and miR-25 KO GP+LC groups, contrary to the obvious difference in tumor volume. This suggests that the tumor volume measurement does not accurately represent the differences in tumor volume, possibly due to the oblong shape of the tumors and a failure to integrate tumor height in the formula used to calculate tumor volume. This casts doubt on previous experiments that do not quantify tumor mass. This is further corroborated by the black and blue lines, where volume suggests a ~1.5x difference between tumor volume while tumor mass is nearly identical.

Response:

We thank the reviewer for this important observation. To address concerns about the discrepancy between tumor volume and mass, we repeated all in vivo validation experiments and included endpoint tumor weights **(Figures 3K, 4C, 4I, and 5H)**.

For the macrophage depletion experiments (LC groups), we extended the observation period to day 15 for a more thorough assessment. In the updated dataset, macrophage depletion (NTC + LC) resulted in reductions in both tumor volume and tumor weight, with outcomes closely resembling those of the miR-25 KO + combination therapy group **(Figures 3J and 3K)**. These findings align with prior studies demonstrating that depleting macrophages can significantly hinder tumor progression in immune-evasive models. [30,31]

To further confirm effective macrophage depletion, we performed flow cytometry, which showed substantial reductions in both tumor-associated and splenic macrophages **(Figure 3L)**, reinforcing the functional relevance of this intervention. We believe these updates improve clarity regarding the tumor volume and mass measurements and strengthen the consistency and robustness of our in vivo findings.

12. Despite efficient reduction of splenic and tumoral macrophages by LC treatment, the lack of difference in tumor mass in the yellow and red groups casts doubt whether miR-25 KO GP treatment works through macrophage infiltration. Additionally, without sequencing of the miR-25 KO GP tumors, suggesting that complement signaling has changed is slightly overreaching.

Response:

We appreciate the reviewer's thoughtful suggestion and agree that additional validation is important to support our mechanistic conclusions. Many of the relevant points have been addressed in our response above to **specific point 11**.

To further clarify the role of macrophages in complement signaling, we performed immunohistochemistry (IHC) for complement component C3 in tumor sections. We observed a marked reduction in C3 deposition following macrophage depletion **(Figure 3M)**, suggesting that complement activation—particularly C3 accumulation—is dependent on macrophages in our model and is downstream of miR-25 regulation.

Although we did not perform RNA-seq specifically on macrophage-depleted tumors, the combination of diminished C3 signal by IHC, effective macrophage depletion, and reduced tumor mass in these groups strongly supports the conclusion that macrophages play a central role in mediating the complement-driven immune remodeling observed in miR-25 KO tumors. We also updated **Discussion section lines 603 – 621** to clarify this interpretation.

Figure 3
13. While they may be simply validating their results in another model when using MC38 for scRNA-seq, it should be explicitly stated if they attempted scRNA-seq on B16F10 and what was the outcome, if any, was. There are multiple published gene signatures that can be utilized to define M1 vs M2 macrophages. Categorizing a macrophage population as M1 because of a unique metabolic signature, and simply defining the rest as M2 is not appropriate.

Response:

(1) MC38 scRNA-seq and B16 validation

We have addressed the rationale for our choice of tumor models, including the use of MC38 for scRNA-seq and B16 for validation, in our response to **major points** above: **(2) Model Comparison and Phenotypic Consistency.**

(2) Macrophage markers used in scRNA-seq

In the revised manuscript, we have expanded and clarified our analysis through updated single-cell RNA-seq and flow cytometry validation, with particular focus on macrophage heterogeneity in the tumor microenvironment (TME).

To address the observed reduction in macrophages in miR-25 KO tumors, we interpret this finding in the context of distinct macrophage subpopulations. Our updated scRNA-seq analysis **(Figure 2)** shows a selective decrease in two M2-like tumor-associated macrophage (TAM) clusters (C19 and C20), which express classic M2 markers such as Thbs1, Fn1, Ctsd, Ctsc, and Chil3. In contrast, the M1-like, antigen-presenting macrophage cluster (C18)—characterized by high expression of MHC II genes including H2-Ob, H2-Eb1, H2-DMb2, H2-DMb1, H2-Ab1, and H2-Aa—was not reduced **(Figure 2D)**. Instead, we observed a significant upregulation of MHC II molecules, particularly H2-Ob and H2-DMb2, in miR-25 KO tumors **(Figure 2E)**.

Furthermore, these M1-like TAMs (C18) also exhibited decreased expression of many immune suppressive genes, including Fcgr1, Lgals3, Lgals1, S100a10, Fgl2, Cd44, Spp1, and Tyrobp (**Figure 2F and Supplemental Table 5, sheet "C18_H2-Aa_TAM")**. This shift in gene expression suggests a polarization of antigen-presenting macrophages away from an M2-like state, driven by miR-25 loss in the tumor.

These findings were validated by flow cytometry in B16 melanoma, which showed decreased total macrophages **(Figures 3D and 3E)** and M2 macrophages **(Figure 3H)** but increased populations of MHC II+ macrophages in both the tumor and spleen **(Figures 3F and 3G)**. As M2-like macrophages typically comprise most of the TAM population, their selective depletion likely explains the overall reduction in macrophage percentages, even though M1-like macrophages are maintained or even enriched.

14. Additionally, authors failed to recapitulate a difference in CD8 and CD4 cells in MC38 tumors as in B16F10, and did not mention if they observed a difference in delta gamma T cells in B16F10 or simply did not check. Decrease in dendritic cells was unaddressed. The difference in delta gamma T cells, while significant, is not particularly meaningful. Furthermore, the difference in MC38 cells by scRNA-seq is also marginally significant, suggesting that the reduced MC38 tumor volume in Figure 1 may be simply due to a lack of macrophages, while the number of tumor cells is relatively unchanged and the tumor remains cold. It is unclear if the differences observed across models are a result of using different approaches (FACS, IF and bulk RNAseq in B16 vs scRNA-seq in MC38); therefore using the same techniques and comparing models side by side seems necessary to extract solid phenotypic conclusions.

Response:

We thank the reviewer for raising these detailed questions. In response, we have re-analyzed the scRNA-seq dataset and provide the following clarifications:

(1) T Cell Populations

In our original submission, we reported a modest increase in γδ T cells. However, in our updated scRNA-seq analysis with improved clustering resolution, we identified nine distinct T cell clusters **(Figure S2D)**, each annotated with representative marker genes **(Supplemental Table 4, sheet "T_cell_subcluster")**.

Among these, C2 expresses tissue-resident and innate markers such as Ccl5, Ly6c2, and Cd69, and was annotated as tissue-resident memory T cells (TRMs). TRMs were significantly increased in miR-25–deficient tumors. However, we did not observe strong activation signatures or enriched effector pathways in these cells **(Figure S2E; Supplemental Table 5, sheet "C2_TRM")**. Given the concurrent reduction in tumor cell clusters (C25, C27 – C29) and M2-like macrophages (C19, C20), we interpret this increase in T cell proportion as a relative shift in composition, rather than a direct expansion or activation of T cells. Thus, we infer that T cells are not the primary mediators of the immune remodeling observed in the miR-25–deficient tumor microenvironment.

(2) Dendritic Cells (DCs)

DCs were detected in the scRNA-seq dataset, as shown in **Figure 2B** and annotated in **Supplemental Table 4, sheet "Myeloid_subcluster"**. We observed no significant difference in DC abundance or activation markers between NTC and miR-25 KO groups **(Supplemental Table 5, sheet "C17_DC")**. Given their low representation and lack of transcriptional modulation, we conclude that DCs are unlikely to be central players in the miR-25–dependent immune phenotype.

(3) Tumor Cells:

To improve tumor cell identification within the scRNA-seq data, we utilized both the MC38 tumor marker Shisal2b **(Figure S2B)** and inferCNV (https://github.com/broadinstitute/inferCNV) analysis to detect genomic amplifications/deletions consistent with tumor cells **(Figure S4B)**. This enabled us to confidently identify over 10,000 tumor cells divided into seven clusters. Among these, four clusters showed significant reductions in cell number in the miR-25 KO group **(Figure 2C)**. Coupled with increased T cell proportions **(Figure 2C)** and enhanced macrophage antigen presentation **(Figure 2E)**, these findings suggest that the miR-25 KO TME is transitioning from immune "cold" to "hot", even in the absence of dramatic increases in total CD8+ or CD4+ T cell counts.

15. Decrease in macrophage interferon signaling during immunotherapy response inconsistent with what's known in the literature and claims in Figure 4. Interferon gamma levels were unaddressed. Additionally, the complement signaling in the MC38 appears to come from fibroblasts instead of macrophages like in the B16F10.

Response:

(1) Inconsistency of macrophage interferon signaling and interferon gamma levels

In our dataset, we observed a downregulation of type I IFN–associated genes—including Ifitm2, Ifitm3, Isg15, Ifit2, among others—as well as decreased expression of M2 polarization–related genes in M1-like antigen-presenting macrophages (cluster C18) **(Figure 2E)**. Type I interferons (e.g., Ifit2, Ifit3, Ifitm2, and Ifitm3) are classically known for their antiviral roles. [32,33] Although IFN-I has been used in solid tumor treatment since the 1980s, its clinical success has been limited. Recent studies suggest IFN-I has context-dependent effects in the tumor microenvironment (TME), and in some cases, may even support immunosuppressive programs. For example, IFN-I–treated monocytes have been shown to induce Arg1 expression (encoding arginase-1), which promotes a tumor-supportive phenotype in TAMs. [34]

Based on these findings, we hypothesize that in our system, the downregulation of type I IFN genes in macrophages may reflect a shift away from suppressive, M2-like functions toward a more pro-inflammatory, antigen-presenting M1-like state in miR-25 KO tumors.

In contrast, type II IFN (IFN-γ) plays a distinct and central role in our model by mediating tumor intrinsic resistance to ICT. [35,36] Anti–PD-1 therapy enhances $CD8^+$ T cell–mediated cytotoxicity and elevates IFN-γ production, which promotes tumor cell killing. [37-39] We found that miR-25 KO sensitize tumor cells to immune pressure in an IFN-γ–dependent manner **(Figure 4A)**, a finding further supported by IFN-γ depletion experiments. IFN-γ depletion reverted the therapeutic effects of miR-25 KO, including suppression of tumor growth, activation of macrophages, and complement infiltration **(Figures 4B – 4E)**. These effects were accompanied by reverted expression of Sdc3 in vitro without IFN-γ **(Figures 5B, 5E, and S5A)**, supporting our model that miR-25 represses Sdc3 in an IFN-γ-dependent context, thereby facilitating immune recognition and clearance of tumor cells.

(2) Interferon gamma levels were unaddressed

In the revised manuscript, we addressed IFN-γ levels by analyzing tumor cell–specific IFN-γ response signatures using our scRNA-seq dataset **(Figures 4A and S4D)**. To further validate the functional relevance of IFN-γ, we performed IFN-γ depletion experiments in vivo **(Figure 4B – 4E)**, which reversed the therapeutic effects of miR-25 knockout. Additionally, in vitro analysis showed that Sdc3 expression was restored in the absence of IFN-γ **(Figures 5B, 5E, and S5A)**, supporting the conclusion that IFN-γ directly influences miR-25–mediated tumor-intrinsic responses during immune checkpoint therapy.

(3) Complement signaling in B16

We appreciate the reviewer's insightful comment regarding the source of complement C3 in the B16 melanoma model. We do not claim that macrophages are the source of C3 in B16, as our IHC staining cannot definitively determine the cellular origin of C3. However, we observed highly consistent phenotypes in B16 tumors that mirror those in the MC38 model—including reduced M2-like macrophage populations, increased MHC II+ macrophages, and elevated complement C3 activation.

Given these similarities, we believe the most likely source of C3 in B16 tumors is also fibroblasts, as observed in the MC38 scRNA-seq data.

16. Defining Atp6v0c and Tubb4b as regulators of BCR signaling seems overreaching.

Response:

We thank the reviewer for pointing out the overinterpretation in our original submission. In the revised manuscript, we have removed the TCR and BCR signaling, as our updated data do not support a central role for T or B cells in the immune remodeling observed in miR-25–deficient tumors.

Figure 4
17. Unclear if the activated splenic macrophages upon IFN-y depletion are also present in the tumor. Evidence for differential IFN-y signaling was not observed by scRNA-seq in the previous figure.

Response:

We appreciate the reviewer's question. In response, we have repeated the IFN-γ depletion assays, and the updated results are now shown in **Figures 4B – 4E** and **S4E – S4F**. Notably, we confirmed that activated (MHC II+) macrophages remain present in tumors following IFN-γ depletion **(Figure 4D, right)**, indicating an IFN-γ dependent repression of macrophage antigen presentation.

Additionally, we have now included an assessment of IFN-γ signaling activity in tumor cells, based on transcriptional profiling, in **Figure 4A** and **Figure S4D**, to better capture the impact of IFN-γ in the miR-25–deficient context.

18. Unclear if the reduction in tumor growth upon Ago2 depletion can be simply explained by reduced proliferation of Ago2-depleted tumor cells. Proliferation effects should be shown. Ago2 sgRNA sequence and thus their specificity is unknown.

Response:

Thank you for the question. We have now included the sequences of the Ago2-targeting sgRNAs in the revised **Supplemental Table 3, sheet "Primers for sgRNAs"**.

Additionally, we performed a detailed evaluation of the Ago2 depletion experiments, including in vitro proliferation assays. The results are now shown in **Figure 4G**, confirming that there's no intrinsic differences in proliferation of Ago2 KO and DKO.

19. The authors did not mention whether Sdc3 expression was increased in in vivo MC38 miR-25 KO cells by scRNA-seq, this is important to validate. Besides miR-92, it does not appear other miRNAs of the cluster have complementarity to Sdc3 3'UTR and miR-363 does not appear to be a hit from the original screen in Figure 1. Luciferase reporter assays should be conducted using the entire Sdc3 3'UTR sequence and not just the putative binding site.

Response: We thank reviewer for raising these important questions,

(1) In vivo MC38 miR-25 KO Sdc3 expression

Sdc3 expression was not significantly increased in miR-25 KO tumor clusters at day 17 when performing scRNA-seq **(Figure S6B)**, likely due to the delayed sampling timepoint during treatment. Our in vitro data, however, showed that Sdc3 is stabilized within 24–48 hours of IFN-γ stimulation **(Figures 5C and S5B)**, suggesting that this regulation is time dependent and may be most pronounced during early immune activation. We also updated **Discussion section lines 676 – 704** to clarify this interpretation.

(2) Other miRNAs in the miR-17-92a family and Sdc3 3′UTR complementarity

We have re-analyzed our small RNA-seq dataset to include both Combination (GVAX + anti–PD-1) vs. Control (untreated) and Combination vs. GVAX-only comparisons, as now shown in **Figure 1C** of the revised

manuscript. Our updated analysis confirms that while multiple members of the miR-17-92 cluster and its paralogs are significantly downregulated in the Combination vs. Control comparison, only miR-25 is significantly downregulated in both comparisons. Therefore, we focused our studies on mR-25.

(3) Luciferase reporter assay

We reconstructed the luciferase reporter plasmid using the full-length Sdc3 3′UTR (3 kb). The updated results are now included in **Figures 4M and 4N**.

20. Ago2 levels should be profiled by qPCR and western blot in their 96hr timecourse to confirm that Ago2 levels do not proportionally increase alongside Sdc3 upon miR-25 KO, which would partially invalidate their findings. They appear consistent in the immunoprecipitation, but without a loading control.

Response:

We thank the reviewer for the suggestion regarding mechanistic validation of Sdc3 regulation by the miRISC complex. In our model, Ago2 protein functions as the core component of the miRISC machinery involved in miR-25–mediated regulation of Sdc3, as validated in **Figures 4F – 4K**. Accordingly, we used Ago2 protein levels, rather than Ago2 mRNA expression, to assess miRISC abundance. In addition, we have included the loading control (Gapdh) for the RIP-qPCR in **Figure 5A**, confirming that equal amounts of protein were loaded and that Ago2 expression levels are not significantly altered in association with changes in Sdc3 expression.

Figure 5

21. It appears authors saw no differences by CIBERSORT in M1 and M2 macrophages like their murine models but instead chose to focus on M0 macrophages. The quantity of delta gamma T cells is very low and marginally different between low and high groups. miR-25 showed very weak negative correlation with complement genes.

Response:

We thank the reviewer for the question. We have removed the CIBERSORT analysis from the revised manuscript for the following reasons:

(1) In our scRNA-seq dataset, antigen-presenting macrophages (M1-like) could not be confidently classified as classical M1 macrophages, as many canonical M1 markers remained unchanged **(Figure 2D)**. This observation was further validated in the B16 melanoma miR-25 KO model, where we observed no significant changes in $CD86^+$ macrophages **(Figure 3I)**. Therefore, the CIBERSORT-based estimation of macrophage infiltration may not accurately reflect the macrophage phenotypes present in our models.

(2) We also removed T cell population analysis from the CIBERSORT output, as the marker sets used by CIBERSORT may not reliably distinguish T cell subsets in our experimental context.

(3) Our current analysis is focused on miR-25, and its immune regulatory function. To this end, we highlight the strong negative correlation between miR-25 expression and complement gene signatures, as shown in our GSEA analysis **(Figures 3B and 3C)**.

22. There are very few responders in the immunotherapy dataset. Many of the nonresponders also had low miR-25 levels. Negative correlation with complement genes is not very convincing as some of the lowest expressing miR-25 patients had very low complement activity' the negative association is only clearly evident in one patient. miR-17HG appears positively correlated with miR-25, unlike in panel f.

Response:

We thank the reviewer for raising the question regarding the immunotherapy dataset. Due to the limited number of available patient samples, we have removed this analysis from the revised manuscript. The small sample size precludes any reliable conclusions regarding the correlation between miR-25, Sdc3, and complement activation, and we believe its inclusion would not meet the rigor expected for clinical relevance.

23. It is still unclear where IFN-y is coming from in the in vivo murine models and whether it negatively correlates with miR-25 in patients. They should also examine if Sdc3 mRNA levels inversely correlate with miR-25 in patient samples to further confirm the relevance of the proposed model to human.

Response:

We appreciate the reviewer for raising these important questions.

(1) Regarding the source of IFN-γ

IFN-γ secretion is a hallmark of enhanced anti-tumor immunity. Anti–PD-1 immunotherapy promotes $CD8^{+}$ T cell–mediated cytotoxicity and leads to increased secretion of IFN-γ, which contributes to tumor cell killing [37-39]. Our analysis of TCGA-SKCM-TM patient data revealed a negative correlation between miR-25 expression and the IFN-γ response signature, as shown by GSEA in **Figure 3B**.

(2) Sdc3 mRNA levels and correlation with miR-25 in patient samples

Sdc3 has historically been reported as a neuron-enriched gene, primarily expressed in the brain. [54,55] However, recent scRNA-seq studies, [56] including our own dataset, have revealed strong Sdc3 expression in immune-related tissues such as the spleen and lymph nodes **(Figures S6A and S6C)**, indicating a broader tissue distribution than previously appreciated.

Given the cellular heterogeneity of tumors—including malignant, immune, stromal, and potentially neuronal components—bulk RNA-seq data from patient samples may not accurately reflect tumor cell–intrinsic correlations between miR-25 and Sdc3. Therefore, such correlation analyses using bulk transcriptomic data must be interpreted with caution.

That said, in TCGA-SKCM-TM samples, tumors with low miR-25 expression showed enhanced immune activation **(Figure S3B)** and downregulation of synapse-related pathways **(Figure S6D)**, suggesting the presence of a neuron-like gene signature in epithelial tumors. These findings may explain why miR-25 knockout consistently elicits immune-stimulatory effects across three epithelial cancer models—B16 (melanoma), MC38 (colon), and 4T1 (triple-negative breast cancer)—under immune checkpoint therapy.

We hypothesize that Sdc3, an atypical cell surface antigen in epithelial tumors, may trigger neuron-associated inflammatory signaling in the tumor microenvironment. However, the mechanisms underlying ectopic Sdc3 expression in epithelial cancers and its role in tumor progression, ECM remodeling, or metastasis remain to be elucidated. We also updated **Discussion section lines 676 – 722** to clarify this interpretation.

Reviewer #4 (Remarks to the Author): with expertise in cancer, miRNAs, syndecan

• In this study, the authors analyzed the clinical and biological significance of the expression of miRNA-25 and its role in the response of anti-PD1 therapy in Melanoma cancer. To this end, they employed a mouse model of Melanoma. The authors validated some results in a mouse model of colon cancer and triple-negative breast cancer. The authors conclude that miR-25 and some family members of miR-17/92 were downregulated in melanoma tumors. The deletion of miRNA-25 impacts the response to anti-PD1 treatment by making tumors more sensitive to therapy. They also concluded that this was due to an induction of the innate immune response and the upregulation of the heparan sulfate proteoglycan Syndecan-3 and the miRNA complex in the presence of IFN-γ. Finally, through bioinformatics analysis using public databases, the authors observed that miRNA-25 expression impacts the survival of melanoma patients. Although this study is of clinical and biological relevance in the treatment of Melanoma, I consider some concerns must be addressed before considering the manuscript for publication.
Comments:
Major
- Figure 1c, why did the authors decide to make the comparison only for GVAX + αPD-1 (GP) vs Control? the fact that the other conditions do not significantly change tumor volume does not mean that there is no change in miRNA expression in the other conditions. It would be informative to know whether the changes in miRNA expression are really due to an additive effect of the two treatments or come from the effect of the αPD-1 treatment, or the effect of the factors generated by the GVAX inoculation. Please make comparisons for each group.

Response:

We thank the reviewer for raising this important point. In response, we have re-analyzed our small RNA-seq dataset to include both Combination (GVAX + anti–PD-1) vs. Control (untreated) and Combination vs. GVAX-only comparisons, as now shown in **Figure 1C** of the revised manuscript. Our updated analysis confirms that while multiple members of the miR-17-92 cluster and its paralogs are significantly downregulated in the Combination vs. Control comparison, only miR-25 is significantly downregulated in both comparisons **(Figure 1C)**.

- Figure 2: Flow cytometry Gatings need to be improved (Figure S2). 1) Some populations are too far to the right and appear to fall out of the quadrant. 2) According to the methodology, the cells were incubated with Zombi Aqua to distinguish between live and dead cells. Please show the Gatings where it is shown that the analysis is being done on the live cells (Zombie Aqua-negative) as the they showed in Figure S2-C. Why did the authors decide to use CD24 (which is preferential for LT-B) as a marker for dendritic cells and not CD11c? 3) Probably the authors did not choose well an example of FOXP3+ cells because in the analysis they showed, there are no cells in that quadrant. The same for granzyme B. Overall, flow cytometry data is a fundamental part of the work and the analysis should be very strict.
- The CD206+ macrophages come from the F4/80 population, did the M1 population increase? Please, analyze M1 markers also to ensure that the M2 population of CD206+ macrophages is increasing.

Response:

We thank the reviewer for the detailed and thoughtful suggestions. Below, we address each point specifically:

(1) Flow cytometry gating and marker usage

In the revised manuscript, we refined our flow cytometry strategy to focus specifically on macrophage populations, which were identified as key regulators based on our scRNA-seq and in vivo functional data. To improve the accuracy and clarity of the gating:

(A) Macrophages were defined as live $CD45^+$ $Ly6C^-$ $Ly6G^-$ $CD11b^+$ $F4/80^+$ cells **(Figure S3C)**,

(B) We included additional markers for macrophage functionality, including MHC II, CD206, and CD86 **(Figure S3D)**.

(C) All gating steps were validated using FMO (fluorescence minus one) controls, ensuring specificity and reproducibility **(Figures S3C and S3D)**.

(2) Live cell gating with Zombie Aqua

As correctly noted, we used Zombie Aqua to distinguish live from dead cells. Zombie Aqua–negative cells were gated as live, as shown in the updated **Figure S3C (Zombie Aqua FMO control)**. BV510-labeled compensation beads were used as a positive control to identify Zombie Aqua fluorescence and ensure accurate gating.

(3) Dendritic cell populations

Although dendritic cells (DCs) were present in the scRNA-seq data **(Figure 2B; Supplemental Table 4, sheet "Myeloid_subcluster")**, we observed no significant differences in abundance or activation between NTC and miR-25 KO groups **(Supplemental Table 5, sheet "C17_DC")**. Given their low representation and minimal transcriptional changes, we conclude that DCs are not key players in the miR-25–mediated immune remodeling observed in our model.

(4) Intracellular staining (FOXP3 and Granzyme B)

We acknowledge the reviewer's concern regarding the FOXP3 and Granzyme B plots in our original submission. In the revised manuscript, we removed these intracellular markers from our analysis. Our transcriptomic and in vivo data consistently showed that T and B cell effector markers were not significantly altered in miR-25–deficient tumors, supporting our decision to focus on macrophage populations.

(5) M1 and M2 macrophage analysis

Using scRNA-seq, we identified an M1-like, antigen-presenting TAM population (Cluster C18, H2-Aa+ TAMs), that maintained stable cell numbers **(Figures 2A – 2D)** but showed significant upregulation of MHC II-related genes, including H2-Ob and H2-DMb2 **(Figure 2E)**. This polarization was further validated by flow cytometry in B16 tumors and spleens **(Figure 3F)**. Notably, classical M1 markers such as CD86 did not change significantly in either in scRNA-seq or in flow cytometry **(Figure 3I)**.

In contrast, M2-like macrophages (CD206+) were consistently reduced in both platforms **(Figures 2B – 2D, and 3H)**, indicating a shift away from an immunosuppressive phenotype.

In conclusion, although total macrophages and M2-like macrophages decrease, MHC II+ antigen-presenting M1-like macrophages increase in our model.

- Figure 2 k and figure s2k-l, please corroborate the date by immunohistochemistry like in figure 2h.

Response:

We have included the sample harvesting dates for all relevant analyses in the updated figure legends.

- Figure 3: The authors mention that to know the composition of the microenvironment of the miR-35 KO GP tumors they did the colon cancer single-cell RNA sequencing (scRNA-Seq) model because it is more immunogenic. From Figures 1 to 5 except for Figure 3, all or most of it has been done in the melanoma model, even the validation of their model in the clinical part was done in melanoma patients. Regardless that the colon cancer model is more immunogenic, the authors should do the scRNA-Seq in the B16 melanoma model, to corroborate which immune populations are present in their model and are being regulated by both the miRNA and the anti-PD1 treatment.

Response:

We fully acknowledge that B16 (melanoma) and MC38 (colon carcinoma) are distinct models and were treated with different immunotherapy regimens—GVAX plus anti–PD-1 for B16, and anti–PD-1 alone for MC38.

We initially attempted single-cell RNA-seq (scRNA-seq) on B16 tumors; however, we encountered technical limitations due to poor viability. After dead cell removal, the percentage of live cells consistently fell below the quality control threshold of 90%, preventing us from generating reliable scRNA-seq data in this model. In contrast, MC38 tumors yielded high-quality viable cells, enabling us to successfully perform scRNA-seq.

Despite these differences, we observed consistent functional phenotypes across all three syngeneic tumor models—B16, MC38, and 4T1. Specifically, miR-25 knockout did not impact in vitro proliferation **(Figures 1H, 1I and 1J)**, but significantly enhanced response to anti–PD-1 therapy in vivo in all models **(Figures 1H – 1P)**. Furthermore, the miR-25–dependent regulation of Sdc3 in response to IFN-γ was validated in all three tumor cell lines in vitro **(Figures 5B – 5D)**, supporting the broader relevance of this mechanism across epithelial cancers.

To improve clarity and emphasize logical coherence across models, we have restructured the Results section as follows:

Figure 1: Begins with phenotypic analysis of miR-25 KO across all three tumor models.

Figure 2: Presents MC38-based scRNA-seq analysis, which forms the mechanistic foundation of the study.

Figure 3: Aligns B16 tumor data and TCGA-SKCM human melanoma transcriptomes with the MC38 scRNA-seq findings.

Figures 4–5: Focus on in vivo functional validation of the mechanism (macrophage depletion, IFN-γ depletion, Ago2 KO, and Sdc3 targeting), all performed in the B16 model. The core regulatory axis (miR-25–Sdc3–IFN-γ) was also tested in MC38 and 4T1 cells in vitro to confirm generalizability.

This restructuring highlights mechanistic consistency across different tumor models and emphasizes key phenotypes observed, including reduced total macrophage infiltration, increased MHC II+ macrophage polarization, and enhanced complement activation.

- Figure 4, That's a lot of ideas in one figure that are not so conclusive. For example: Figure 4a: To test the hypothesis that miRNA-25 can regulate PAMP or DAMP expression in tumor cells by the release of IFN-γ due to anti-PD1 treatment, the authors depleted IFN-γ in their miR-25 KO GP mouse model. 1) I suggest first measuring blood IFN- γ concentration and protein levels by flow cytometry in miR-25 KO GP+anti-PD1 models and respective controls. 2) Inhibit IFN- γ and corroborate that it is inhibited by the above methods. My suggestion of points 1 and 2 is because many variables are being applied to the model. Figure 4b: measure also by cytometry if the proportion of resident macrophages changes and the other populations that are severely affected by the effect of IFN- inhibition. Figure 4c: page 12, line 329 double KO of Ago2/miR-25 strongly suggests that miR-25 regulation of immunotherapy requires miRISC. This is not necessarily true because Ago2 KO alone decreases tumor size and the effect of double KO could be because other miRNAs may be being modified. Furthermore, no information is presented on how the immune system could be modified. In this case, it would be worthwhile to do an RNA-seq of these tumors (Ago2/miR-25 DKO and controls).

Response:

We thank the reviewer for the thoughtful questions and suggestions regarding our in vivo validation in the context of immunotherapy. Below are our detailed responses:

(1) IFN-γ concentration and validation of IFN-γ depletion

We assessed tumor cell IFN-γ signaling using scRNA-seq data by calculating the expression of key pathway genes, including Ifng, Stat1, Irf1, Irf8, Irf9 and Cxcl10 **(Figures 4A and S4D)**. This provides a direct readout of IFN-γ response within tumor cells, rather than blood levels.

To further validate the role of IFN-γ, we repeated the IFN-γ depletion experiment using a higher antibody dose (10 µg/mouse, compared to 1.25 µg/mouse in the original submission). The updated data are shown in **Figures 4B – 4E**. IFN-γ depletion in miR-25 KO tumors under combination immunotherapy reversed the tumor-

suppressive effects, resulting in increased tumor volume **(Figures 4B and C)**, elevated macrophage infiltration **(Figure 4D, left)**, and diminished complement C3 deposition **(Figure 4E)**.

Consistently, in vitro analysis using B16 cells showed that in the absence of IFN-γ, miR-25 KO cells displayed lower Sdc3 protein levels compared to controls **(Figures 5B, 5E, and S5A)**. These results support an IFN-γ–dependent regulation of Sdc3, which helps explain our in vivo findings **(Figures 4B – 4E)**, where Sdc3 expression inversely correlates with macrophage infiltration and positively correlates with complement activation. These data cross validated each other and confirmed central role of IFN-γ signaling in our model.

(2) Ago2 depletion

In the revised manuscript, we have repeated and expanded the Ago2 depletion experiments, as shown in **Figures 4F– 4K**. We confirmed Ago2 knockdown **(Figures 4F and 4G)** and analyzed the immune consequences of Ago2 single KO and Ago2/miR-25 double KO (DKO) **(Figures 4J, 4K and S4H)**.

- Figure 4D: page 13, line 336. The authors said the only up-regulated gene common to all datasets was Syndecan-3. The authors observed that depending on the model analyzed, the treatment, and the time of the treatment, there are changes or not in the expression of Syndecan 3. Melanoma has been associated with hypoxia which it is known to regulate syndecan 3 (D'Aguanno, S., Mallone, F., Marenco, M. et al. Hypoxia-dependent drivers of melanoma progression. J Exp Clin Cancer Res 40, 159 (2021). Prieto-Fernández E, Egia-Mendikute L, Bosch A, García Del Río A, Jimenez-Lasheras B, Antoñana-Vildosola A, Lee SY, Palazon A. Hypoxia Promotes Syndecan-3 Expression in the Tumor Microenvironment. Front Immunol. 2020 Sep 30;11:586977.). I suggest in the analysis of Figure 4d, also including the RNA seq of NTC and NTC-GP to know if Sdc3 is overexpressed under these conditions. Sdc-1 expression in tissues could also be checked by immunohistochemistry in the models in Figure 4d plus the controls and associated with Figures 4f-k.

Response:

We thank the reviewer for the thoughtful questions regarding Sdc3 expression dynamics and the potential role of other Syndecan family members, such as Sdc1, in the TME. We have updated **Discussion section on Sdc3 in recent cancer research (lines 685 – 689)**. We address the following questions specifically:

(1) Overlapping in vivo bulk RNA-seq data of NTC and NTC-GP

We fully agree that incorporating a comparison between NTC-GP and NTC bulk RNA-seq would be ideal to further evaluate potential miR-25 targets, including Sdc3. As expected, we anticipate that Sdc3 expression would be elevated in the NTC-GP group due to immune activation. However, given the complexity of the tumor microenvironment (TME), we cannot exclude the possibility that tumor cell–derived Sdc3 expression may be confounded or masked by expression from infiltrating immune or stromal cells **(as interpreted in lines 676 – 704)**.

To address this limitation, we utilized IFN-γ–treated control tumor cells (NTC) in vitro, which endogenously express miR-25. This controlled setting allowed us to more directly assess tumor cell–intrinsic regulation while also simulating the inflammatory conditions experienced by tumor cells under immune pressure. As shown in **Figure 4J**, IFN-γ treatment led to a significant repression of Sdc3 mRNA levels, supporting the model that miR-25 contributes to Sdc3 suppression in the context of immune activation.

(2) Measurement on other Syndecan family members

We did not observe consistent or robust expression of other Syndecan family members (e.g., Sdc1) in our RNA-seq datasets, and Sdc1 is not predicted to be a direct miR-25 target. Therefore, we did not pursue further validation by immunohistochemistry in this context.

Minor

• Introduction:

- The introduction must be improved because is very general and should focus more on melanoma since most of the experiments were done on melanoma models, even the clinical data were done on melanoma patient database.

Response:

Thank you for the suggestion. While many of our in vivo experiments were conducted using the B16 melanoma model, we also included side-by-side analyses of miR-25 KO effects under immune checkpoint therapy in three epithelial cancer models—B16 (melanoma), MC38 (colon carcinoma), and 4T1 (breast cancer) **(Figures 1H – 1P)**.

We identified Sdc3 as a shared downstream target of miR-25 across all three tumor models **(Figures 5B – 5D and S5A – S5C)**. Since all three models represent epithelial cancers, and because Sdc3 is not typically expressed in epithelial tissues, we hypothesize that Sdc3 may act as an ectopic, neuron-related surface antigen that triggers innate and humoral immune responses in the context of ICT.

For this reason, although melanoma is a central focus of our study, we have kept the broader epithelial cancer context in the Introduction to reflect the potential universality of the miR-25–Sdc3 regulatory axis.

- On page 5 lines 87-93, it is not clear why the miR-17-92 family has an impact on tumorigenesis. The authors wrote only some examples of who these miRNAs are regulated by or who they regulate but it would be better if they could give more examples about the role of these miRNAs in tumorigenesis o what is known about miRNAs analyzed in melanoma.

Response:

The miR-17-92 cluster, also known as OncomiR-1, has been widely recognized for its oncogenic role in various cancers, including melanoma. This cluster promotes tumorigenesis by targeting tumor suppressors and regulating genes involved in cell cycle progression and apoptosis.

In melanoma specifically, miR-17 has been shown to support the maintenance of melanoma stemness [57], while miR-25, a paralog within the miR-106b-25 cluster, is reported to enhance melanoma cell migration by targeting DKK3 and modulating the WNT/β-catenin pathway [58]. Additionally, miR-25 is found to be consistently upregulated in melanoma cell lines and patient-derived samples [21,22].

Beyond melanoma, miR-25 has been broadly implicated in various tumorigenic processes [28], including hypoxia-induced immunosuppression via repression of cGAS, a key innate immune DNA sensor [29].

We have incorporated these points into the revised Introduction **(lines 105 –117)** to better articulate the rationale for investigating the miR-17-92 family, particularly miR-25, in melanoma tumorigenesis and immune evasion.

• Methodology.

- Could the authors please explain why in each model (melanoma, colon, and breast) they injected different amounts of cells? Is it a general protocol, or is it something that they already standardized and if so, please provide the reference. Also, could you please explain why there is a big difference between the number of B16 cells vs. the number of GVAX injected?

Response:

We thank the reviewer for raising this important question. The cell numbers used for each tumor model (B16, MC38, and 4T1) were based on well-established protocols from published literature and our lab's prior experience.

Specifically, the injection doses for B16 and GVAX were adapted from a Nature paper, [46] which also provided guidance on irradiation parameters and the number of GVAX cells required for effective immune stimulation. The MC38 cell number was based on a protocol established by our lab and aligned with the 2023 EMBO Journal publication. [59] For 4T1, we referred to a 2014 PNAS study [60] and adjusted the cell number downward to optimize the observation window and remain within UCSD IACUC guidelines.

These references and rationale have now been included in the revised **Methods section** for clarity.

- Add clodronate liposome concentration used. Although the authors described it in the figure legend, please write the IFN-γ concentration in the materials and methods as well.

Response:

We use commercialized clodronate liposome purchased from Fisher Scientific (Cat# CLD-8909-5ML) and intravenously injected 200 µL per mouse each time. For the IFN-γ depletion, we use 10 µg/mouse of rat anti-mouse IFN-γ antibody (clone XMG1.2, Bio X Cell) intraperitoneally. We have also updated in the revised **Methods section** for clarity.

- For intracellular cytometric staining, did the authors use a commercial kit? If so, which one? If not, indicate what was used for fixation and permeabilization.

Response:

In our original submission, we used an intracellular staining kit from BioLegend. However, in the revised manuscript, we have narrowed our focus to macrophage populations (for the surface marker analysis) and have therefore removed intracellular markers such as FOXP3 and Granzyme B from our analysis, which have been updated in the manuscript.

- Were the analyses made from Zombie Aqua negative cells? did they follow the manufacturer's protocol for Zombie Aqua staining? Of the digested tumor, were all the cells used for cytometry, or was a specific concentration used?

Response:

Yes, all analyses were performed on Zombie Aqua–negative (live) cells. We followed the manufacturer's protocol provided by BioLegend for Zombie Aqua staining. Regarding sample preparation, we used all cells from the digested tumor, including debris and dead cells, in the initial staining. However, during flow cytometry analysis, we applied standard gating strategies to exclude debris and dead cells, ensuring that only viable cells were included in downstream analysis **(Figure S3C)**.

- Please add the concentration or dilution of the antibodies used.

Response:

We have updated the antibody list using in the manuscript in **Method section Tumor Dissociation and Flow Cytometry of Immune Cells**. Each antibody was diluted followed by manufacturer's instruction.

- For Luciferase assay please add the concentration of the cells.

Response: We seeded 100 cell/well in a 96-well pate and after 24 hours we transfected the luciferase reporter plasmid and β-galactosidase expressing plasmid as a control. We have also updated the **Method section Endogenous miR Luciferase Assay**.

- In general, unless the authors have already published it, please add the citation in the methodology, otherwise describe in more detail the steps followed in all the experiments.

• Discussion
- Page 16, line 429: Our analysis suggests that cancer cells… I consider that we should not write in general and rather direct the discussion towards melanoma since most of the experiments were done on this type of cancer.

Response: We thank the reviewer for pointing out the issue in Discussion section. This concern was addressed in the **first minor concern above on introduction part**.

- Page 16, line 444: CD68+ CD163-M1 TAMs. add a space between CD163- and M1 because it is not understood that CD163- refers to a negative population for that marker. As it is (CD163-M1) it is understood as if it is an M1 marker when in fact it is an M2.

Response:

We thank the reviewer for pointing out the ambiguity in the phrasing. In the revised Discussion section, we have removed the reference to CD163, as it is no longer a central marker for distinguishing macrophage populations in our study. Instead, we have refined our discussion of M1/M2 macrophage polarization based on our scRNA-seq findings, including changes in MHC II expression, Mif signaling, and the interaction between TAMs and CAFs **(lines 603 – 674)**.

- In general, I suggest a better discussion of the results. It seems that the results are described again but there is little discussion of them. for example, concerning Syndecan-3 it may have different roles depending on the type of cancer, as it influences multiple signaling pathways in a context-dependent manner.

Response: We appreciate this suggestion. We have added a dedicated paragraph in the **Discussion section that more thoroughly explores the role of Syndecan-3 (lines 676 – 722)**.

Reviewer #5 (Remarks to the Author):

I co-reviewed this manuscript with one of the reviewers who provided the listed reports. This is part of the Nature Communications initiative to facilitate training in peer review and to provide appropriate recognition for Early Career Researchers who co-review manuscripts.

Reviewer #6 (Remarks to the Author):

I co-reviewed this manuscript with one of the reviewers who provided the listed reports. This is part of the Nature Communications initiative to facilitate training in peer review and to provide appropriate recognition for Early Career Researchers who co-review manuscripts.

Reviewer #7 (Remarks to the Author):

I co-reviewed this manuscript with one of the reviewers who provided the listed reports. This is part of the Nature Communications initiative to facilitate training in peer review and to provide appropriate recognition for Early Career Researchers who co-review manuscripts.

References

1 Doench, J. G. *et al.* Rational design of highly active sgRNAs for CRISPR-Cas9-mediated gene inactivation. *Nat Biotechnol* **32**, 1262-1267 (2014). https://doi.org/10.1038/nbt.3026

2 Ran, F. A. *et al.* Genome engineering using the CRISPR-Cas9 system. *Nat Protoc* **8**, 2281-2308 (2013). https://doi.org/10.1038/nprot.2013.143

3 Hsu, P. D. *et al.* DNA targeting specificity of RNA-guided Cas9 nucleases. *Nat Biotechnol* **31**, 827-832 (2013). https://doi.org/10.1038/nbt.2647

4 Brinkman, E. K., Chen, T., Amendola, M. & van Steensel, B. Easy quantitative assessment of genome editing by sequence trace decomposition. *Nucleic Acids Res* **42**, e168 (2014). https://doi.org/10.1093/nar/gku936

5 Zhao, X. *et al.* Human Neonatal Fc Receptor Is the Cellular Uncoating Receptor for Enterovirus B. *Cell* **177**, 1553-1565 e1516 (2019). https://doi.org/10.1016/j.cell.2019.04.035

6 Reina-Campos, M. *et al.* Metabolic programs of T cell tissue residency empower tumour immunity. *Nature* **621**, 179-187 (2023). https://doi.org/10.1038/s41586-023-06483-w

7 Levesque, S., Cosentino, A., Verma, A., Genovese, P. & Bauer, D. E. Enhancing prime editing in hematopoietic stem and progenitor cells by modulating nucleotide metabolism. *Nat Biotechnol* (2024). https://doi.org/10.1038/s41587-024-02266-4

8 Annunziato, S. *et al.* Comparative oncogenomics identifies combinations of driver genes and drug targets in BRCA1-mutated breast cancer. *Nat Commun* **10**, 397 (2019). https://doi.org/10.1038/s41467-019-08301-2

9 Lee, B. *et al.* Nanoparticle delivery of CRISPR into the brain rescues a mouse model of fragile X syndrome from exaggerated repetitive behaviours. *Nat Biomed Eng* **2**, 497-507 (2018). https://doi.org/10.1038/s41551-018-0252-8

10 Baek, D. *et al.* The impact of microRNAs on protein output. *Nature* **455**, 64-71 (2008). https://doi.org/10.1038/nature07242

11 Selbach, M. *et al.* Widespread changes in protein synthesis induced by microRNAs. *Nature* **455**, 58-63 (2008). https://doi.org/10.1038/nature07228

12 Wosko, T. J., Ferrara, D. T. & Sartori, L. S. Histological comparison of the B16 melanoma and its F1 variant. *Cancer Lett* **24**, 57-63 (1984). https://doi.org/10.1016/0304-3835(84)90080-6

13 Cruz, P. *et al.* Unraveling the Role of JMJD1B in Genome Stability and the Malignancy of Melanomas. *Int J Mol Sci* **25** (2024). https://doi.org/10.3390/ijms251910689

14 Martinez-Vicente, I. *et al.* Mahogunin Ring Finger 1 Is Required for Genomic Stability and Modulates the Malignant Phenotype of Melanoma Cells. *Cancers (Basel)* **12** (2020). https://doi.org/10.3390/cancers12102840

15 Peng, G. *et al.* miR-25 promotes glioblastoma cell proliferation and invasion by directly targeting NEFL. *Mol Cell Biochem* **409**, 103-111 (2015). https://doi.org/10.1007/s11010-015-2516-x

16 Petrocca, F. *et al.* E2F1-regulated microRNAs impair TGFbeta-dependent cell-cycle arrest and apoptosis in gastric cancer. *Cancer Cell* **13**, 272-286 (2008). https://doi.org/10.1016/j.ccr.2008.02.013

17 Bartel, D. P. MicroRNAs: target recognition and regulatory functions. *Cell* **136**, 215-233 (2009). https://doi.org/10.1016/j.cell.2009.01.002

18 Khvorova, A., Reynolds, A. & Jayasena, S. D. Functional siRNAs and miRNAs exhibit strand bias. *Cell* **115**, 209-216 (2003). https://doi.org/10.1016/s0092-8674(03)00801-8

19 Okamura, K. *et al.* The regulatory activity of microRNA* species has substantial influence on microRNA and 3' UTR evolution. *Nat Struct Mol Biol* **15**, 354-363 (2008). https://doi.org/10.1038/nsmb.1409

20 Medley, J. C., Panzade, G. & Zinovyeva, A. Y. microRNA strand selection: Unwinding the rules. *Wiley Interdiscip Rev RNA* **12**, e1627 (2021). https://doi.org/10.1002/wrna.1627

21 Philippidou, D. *et al.* Signatures of microRNAs and selected microRNA target genes in human melanoma. *Cancer Res* **70**, 4163-4173 (2010). https://doi.org/10.1158/0008-5472.CAN-09-4512

22 Igoucheva, O. & Alexeev, V. MicroRNA-dependent regulation of cKit in cutaneous melanoma. *Biochem Biophys Res Commun* **379**, 790-794 (2009). https://doi.org/10.1016/j.bbrc.2008.12.152

23 de Visser, K. E. & Joyce, J. A. The evolving tumor microenvironment: From cancer initiation to metastatic outgrowth. *Cancer Cell* **41**, 374-403 (2023). https://doi.org/10.1016/j.ccell.2023.02.016

24 Mogilyansky, E. & Rigoutsos, I. The miR-17/92 cluster: a comprehensive update on its genomics, genetics, functions and increasingly important and numerous roles in health and disease. *Cell Death Differ* **20**, 1603-1614 (2013). https://doi.org/10.1038/cdd.2013.125

25 Liao, K. *et al.* Critical roles of the miR-17 approximately 92 family in thymocyte development, leukemogenesis, and autoimmunity. *Cell Rep* **43**, 114261 (2024). https://doi.org/10.1016/j.celrep.2024.114261

26 Wang, J. *et al.* Breast cancer cell-derived microRNA-155 suppresses tumor progression via enhancing immune cell recruitment and antitumor function. *J Clin Invest* **132** (2022). https://doi.org/10.1172/JCI157248

27 Kalkusova, K., Taborska, P., Stakheev, D. & Smrz, D. The Role of miR-155 in Antitumor Immunity. *Cancers (Basel)* **14** (2022). https://doi.org/10.3390/cancers14215414

28 Sarkozy, M., Kahan, Z. & Csont, T. A myriad of roles of miR-25 in health and disease. *Oncotarget* **9**, 21580-21612 (2018). https://doi.org/10.18632/oncotarget.24662

29 Wu, M. Z. *et al.* miR-25/93 mediates hypoxia-induced immunosuppression by repressing cGAS. *Nat Cell Biol* **19**, 1286-1296 (2017). https://doi.org/10.1038/ncb3615

30 Zhang, L. *et al.* Loss of fragile site-associated tumor suppressor promotes antitumor immunity via macrophage polarization. *Nat Commun* **12**, 4300 (2021). https://doi.org/10.1038/s41467-021-24610-x

31 Chen, D. *et al.* Chloroquine modulates antitumor immune response by resetting tumor-associated macrophages toward M1 phenotype. *Nat Commun* **9**, 873 (2018). https://doi.org/10.1038/s41467-018-03225-9

32 Ivashkiv, L. B. & Donlin, L. T. Regulation of type I interferon responses. *Nat Rev Immunol* **14**, 36-49 (2014). https://doi.org/10.1038/nri3581

33 Platanias, L. C. Mechanisms of type-I- and type-II-interferon-mediated signalling. *Nat Rev Immunol* **5**, 375-386 (2005). https://doi.org/10.1038/nri1604

34 Tong, Y. *et al.* Concomitant type I IFN and M-CSF signaling reprograms monocyte differentiation and drives pro-tumoral arginase production. *EBioMedicine* **39**, 132-144 (2019). https://doi.org/10.1016/j.ebiom.2018.11.062

35 Spranger, S. *et al.* Up-regulation of PD-L1, IDO, and T(regs) in the melanoma tumor microenvironment is driven by CD8(+) T cells. *Sci Transl Med* **5**, 200ra116 (2013). https://doi.org/10.1126/scitranslmed.3006504

36 Yu, M. *et al.* Interferon-gamma induces tumor resistance to anti-PD-1 immunotherapy by promoting YAP phase separation. *Mol Cell* **81**, 1216-1230 e1219 (2021). https://doi.org/10.1016/j.molcel.2021.01.010

37 Ayers, M. *et al.* IFN-gamma-related mRNA profile predicts clinical response to PD-1 blockade. *J Clin Invest* **127**, 2930-2940 (2017). https://doi.org/10.1172/JCI91190

38 Grasso, C. S. *et al.* Conserved Interferon-gamma Signaling Drives Clinical Response to Immune Checkpoint Blockade Therapy in Melanoma. *Cancer Cell* **38**, 500-515 e503 (2020). https://doi.org/10.1016/j.ccell.2020.08.005

39 Minn, A. J. & Wherry, E. J. Combination Cancer Therapies with Immune Checkpoint Blockade: Convergence on Interferon Signaling. *Cell* **165**, 272-275 (2016). https://doi.org/10.1016/j.cell.2016.03.031
40 Dranoff, G. *et al.* Vaccination with irradiated tumor cells engineered to secrete murine granulocyte-macrophage colony-stimulating factor stimulates potent, specific, and long-lasting anti-tumor immunity. *Proc Natl Acad Sci U S A* **90**, 3539-3543 (1993). https://doi.org/10.1073/pnas.90.8.3539
41 Wu, B., Zhang, B., Li, B., Wu, H. & Jiang, M. Cold and hot tumors: from molecular mechanisms to targeted therapy. *Signal Transduct Target Ther* **9**, 274 (2024). https://doi.org/10.1038/s41392-024-01979-x
42 Duan, Q., Zhang, H., Zheng, J. & Zhang, L. Turning Cold into Hot: Firing up the Tumor Microenvironment. *Trends Cancer* **6**, 605-618 (2020). https://doi.org/10.1016/j.trecan.2020.02.022
43 Bohm, W. *et al.* T cell-mediated, IFN-gamma-facilitated rejection of murine B16 melanomas. *J Immunol* **161**, 897-908 (1998).
44 Balood, M. *et al.* Nociceptor neurons affect cancer immunosurveillance. *Nature* **611**, 405-412 (2022). https://doi.org/10.1038/s41586-022-05374-w
45 Badrinath, S. *et al.* A vaccine targeting resistant tumours by dual T cell plus NK cell attack. *Nature* **606**, 992-998 (2022). https://doi.org/10.1038/s41586-022-04772-4
46 Manguso, R. T. *et al.* In vivo CRISPR screening identifies Ptpn2 as a cancer immunotherapy target. *Nature* **547**, 413-418 (2017). https://doi.org/10.1038/nature23270
47 Redenti, A. *et al.* Probiotic neoantigen delivery vectors for precision cancer immunotherapy. *Nature* **635**, 453-461 (2024). https://doi.org/10.1038/s41586-024-08033-4
48 Feng, B. *et al.* The type 2 cytokine Fc-IL-4 revitalizes exhausted CD8(+) T cells against cancer. *Nature* **634**, 712-720 (2024). https://doi.org/10.1038/s41586-024-07962-4
49 Baumgartner, C. K. *et al.* The PTPN2/PTPN1 inhibitor ABBV-CLS-484 unleashes potent anti-tumour immunity. *Nature* **622**, 850-862 (2023). https://doi.org/10.1038/s41586-023-06575-7
50 Li, N. *et al.* ALKBH5 regulates anti-PD-1 therapy response by modulating lactate and suppressive immune cell accumulation in tumor microenvironment. *Proc Natl Acad Sci U S A* **117**, 20159-20170 (2020). https://doi.org/10.1073/pnas.1918986117
51 Yuan, S. *et al.* VDAC2 loss elicits tumour destruction and inflammation for cancer therapy. *Nature* (2025). https://doi.org/10.1038/s41586-025-08732-6
52 Guo, C. *et al.* SLC38A2 and glutamine signalling in cDC1s dictate anti-tumour immunity. *Nature* **620**, 200-208 (2023). https://doi.org/10.1038/s41586-023-06299-8
53 Breart, B. *et al.* IL-27 elicits a cytotoxic CD8(+) T cell program to enforce tumour control. *Nature* **639**, 746-753 (2025). https://doi.org/10.1038/s41586-024-08510-w
54 Carey, D. J. Syndecans: multifunctional cell-surface co-receptors. *Biochem J* **327 (Pt 1)**, 1-16 (1997). https://doi.org/10.1042/bj3270001
55 Bernfield, M. *et al.* Biology of the syndecans: a family of transmembrane heparan sulfate proteoglycans. *Annu Rev Cell Biol* **8**, 365-393 (1992). https://doi.org/10.1146/annurev.cb.08.110192.002053
56 Zhang, L. *et al.* Single-Cell Analyses Inform Mechanisms of Myeloid-Targeted Therapies in Colon Cancer. *Cell* **181**, 442-459 e429 (2020). https://doi.org/10.1016/j.cell.2020.03.048
57 Zhang, Y., Yang, X., Cui, Y. & Zhang, X. Suppression of RNA editing by miR-17 inhibits the stemness of melanoma stem cells. *Mol Ther Nucleic Acids* **27**, 439-455 (2022). https://doi.org/10.1016/j.omtn.2021.12.021
58 Huo, J. *et al.* Upregulated MicroRNA-25 Mediates the Migration of Melanoma Cells by Targeting DKK3 through the WNT/beta-Catenin Pathway. *Int J Mol Sci* **17** (2016). https://doi.org/10.3390/ijms17111124

59 Wang, L. *et al.* Role of PCIF1-mediated 5'-cap N6-methyladeonsine mRNA methylation in colorectal cancer and anti-PD-1 immunotherapy. *EMBO J* **42**, e111673 (2023). https://doi.org/10.15252/embj.2022111673

60 Kim, K. *et al.* Eradication of metastatic mouse cancers resistant to immune checkpoint blockade by suppression of myeloid-derived cells. *Proc Natl Acad Sci U S A* **111**, 11774-11779 (2014). https://doi.org/10.1073/pnas.1410626111

We thank all the reviewers for their insightful comments and constructive suggestions. In response, we have substantially revised and strengthened the manuscript according to their recommendations. Below, we provide a detailed, point-by-point response outlining how each concern has been addressed. Reviewers' comments are shown in black, and our responses are provided in blue. For convenience, we have organized key results and analyses into rebuttal figures and refer to the corresponding main-text figures.

Reviewers' comments:
Reviewer #1 (Remarks to the Author):

In my initial review, I raised four key concerns that, in my view, needed to be addressed to rule out the possibility that the authors' findings were not due to off-target effects on miR-25/miR-106.
(1) My first concern was whether the sgRNAs effectively depleted the mature miRNAs. I asked the authors to provide the sequences of the sgRNAs used in the knockout experiments and to isolate subclones in which homozygous targeting of the desired miRNAs could be confirmed. I now understand that the authors targeted either the terminal loop or the stem region of the precursor. However, they report being unable to isolate subclones with homozygous editing, despite describing 50–75% editing in the bulk population. Their explanation—that this reflects the "inherent plasticity of cancer cells"—is unconvincing. Does this mean that cells with edited miR-25 frequently revert to wild type? Or that loss of miR-25 is not tolerated by cancer cells? Neither explanation is persuasive, especially considering that mice lacking the entire miR-106b-25 locus are viable and appear phenotypically normal.

Thank you for this important point and we fully appreciate the concern regarding whether homozygous deletion of miR-25 is tolerated by cancer cells—a key question underlying our study. We have now addressed this issue comprehensively from three perspectives.

(1) Isolation of homozygous miR-25 knockout clones in cancer and non-cancer cell lines

We generated single-cell clones from three murine cancer lines (B16, MC38, and 4T1) and two human lines (A375, melanoma; HEK293T, non-cancerous). We successfully obtained homozygous miR-25 knockout clones, as confirmed by Sanger sequencing **(Rebuttal Figs. 1a–1e; full sequencing results in Supplementary Table 4)**. All single-cell clones—both cancerous and non-cancerous—remained viable after miR-25 deletion. This finding aligns with prior evidence showing that deletion of the entire miR-106b-25 cluster in mice yields viable and fertile offspring without overt abnormalities (Ventura et al., Cell, 2008; PMID: 18329372; strain: Mirc3^tm1.1Tyj/J, JAX#008460).

Quantitative PCR analysis further confirmed robust suppression of mature miR-25-3p in most homozygous knockout clones relative to mixed populations **(Rebuttal Fig. 1f)**. In A375, no additional decrease was detected compared with pooled knockout, which already exhibited >90–100% genomic editing efficiency **(main-text Fig.S6C)**. In this case, the pooled knockout effectively represents a functional homozygous knockout.

In our initial pooled knockout experiments, the observed 50–75% editing efficiency in some lines reflects heterogeneous sgRNA cutting and clonal mosaicism rather than reversion or negative selection. Indeed, homozygous clones can be isolated and maintained over multiple passages without genotypic reversion, confirming that loss of miR-25 is stably tolerated.

<u>These data are now presented in current Figs. S1D, S1E, 6B, 6C, S6A, and Supplementary Table 4.</u>

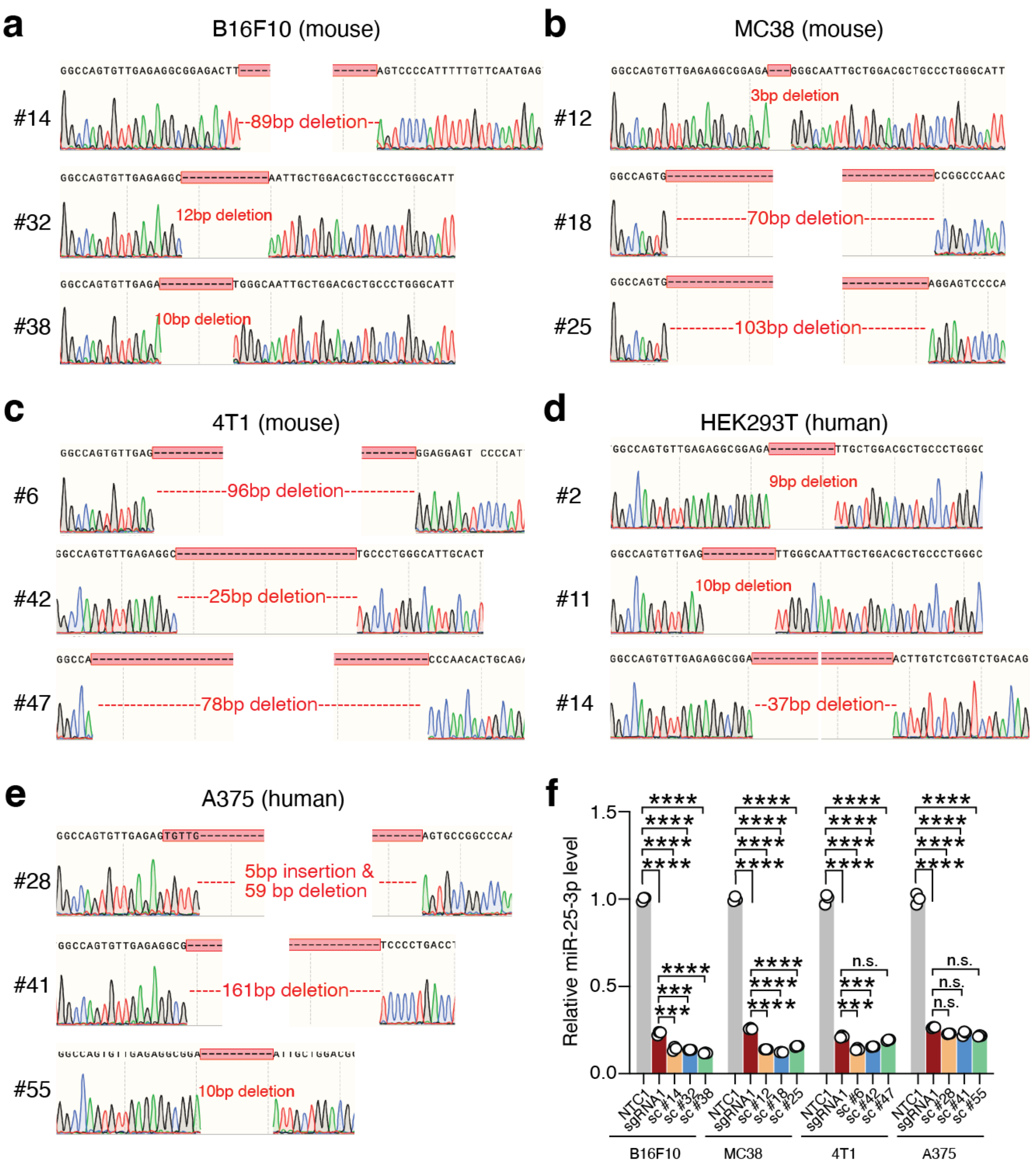

Rebuttal Figure 1. Validation of homozygous miR-25 knockout in murine and human cell lines

Representative Sanger sequencing chromatograms showing CRISPR–Cas9–induced deletions or insertions in the miR-25 genomic locus across multiple single-cell clones.

(a) B16F10 (mouse melanoma), (b) MC38 (mouse colorectal carcinoma), (c) 4T1 (mouse breast carcinoma), (d) HEK293T (human embryonic kidney), and (e) A375 (human melanoma). Red boxes and dashed lines denote deletion or insertion regions, with corresponding base-pair (bp) lengths indicated. All clones shown represent confirmed homozygous knockout alleles.

*(f) RT–qPCR analysis of mature miR-25-3p expression in control (NTC1), pooled knockout (sgRNA1), and three independent homozygous knockout clones from B16, MC38, 4T1, and A375. Data represent mean ± SEM (n = 3). Statistical significance was determined by two-tailed Student's t-test (***P < 0.001; ****P < 0.0001).*

(2) Viability and functional tolerance of miR-25 loss in murine cancer cells

We next assessed whether homozygous loss of miR-25 affects cancer cell fitness. In B16 melanoma cells—the principal tumor model of our study—neither sgRNA transduction nor homozygous deletion altered cell proliferation, apoptosis (<10% in all cases), or migration following serum starvation and 24-hour recovery **(Rebuttal Figs. 2a-2d)**. Similar results were obtained in MC38 and 4T1 cell lines **(Rebuttal Figs. 2e-2l)**. These findings reproduce our prior pooled-KO results showing no change in in vitro or in vivo viability **(main-text Figs. 1H–1J and 1K–1P, isotype controls)**.

To determine whether the homozygous knockouts recapitulate the immunotherapy phenotype, we transplanted single-clone B16 miR-25 KOs into C57BL/6 mice and treated with GVAX + anti-PD-1. These clones exhibited comparable sensitization to combination therapy as pooled knockouts **(Rebuttal Figs. 2m-2n)**. Collectively, these data confirm that CRISPR–Cas9-mediated homozygous deletion of miR-25 is well tolerated and faithfully reproduces the phenotypes observed in pooled knockouts.

<u>These data are now presented in current Figs. S1F-S1R and Supplementary Table 4.</u>

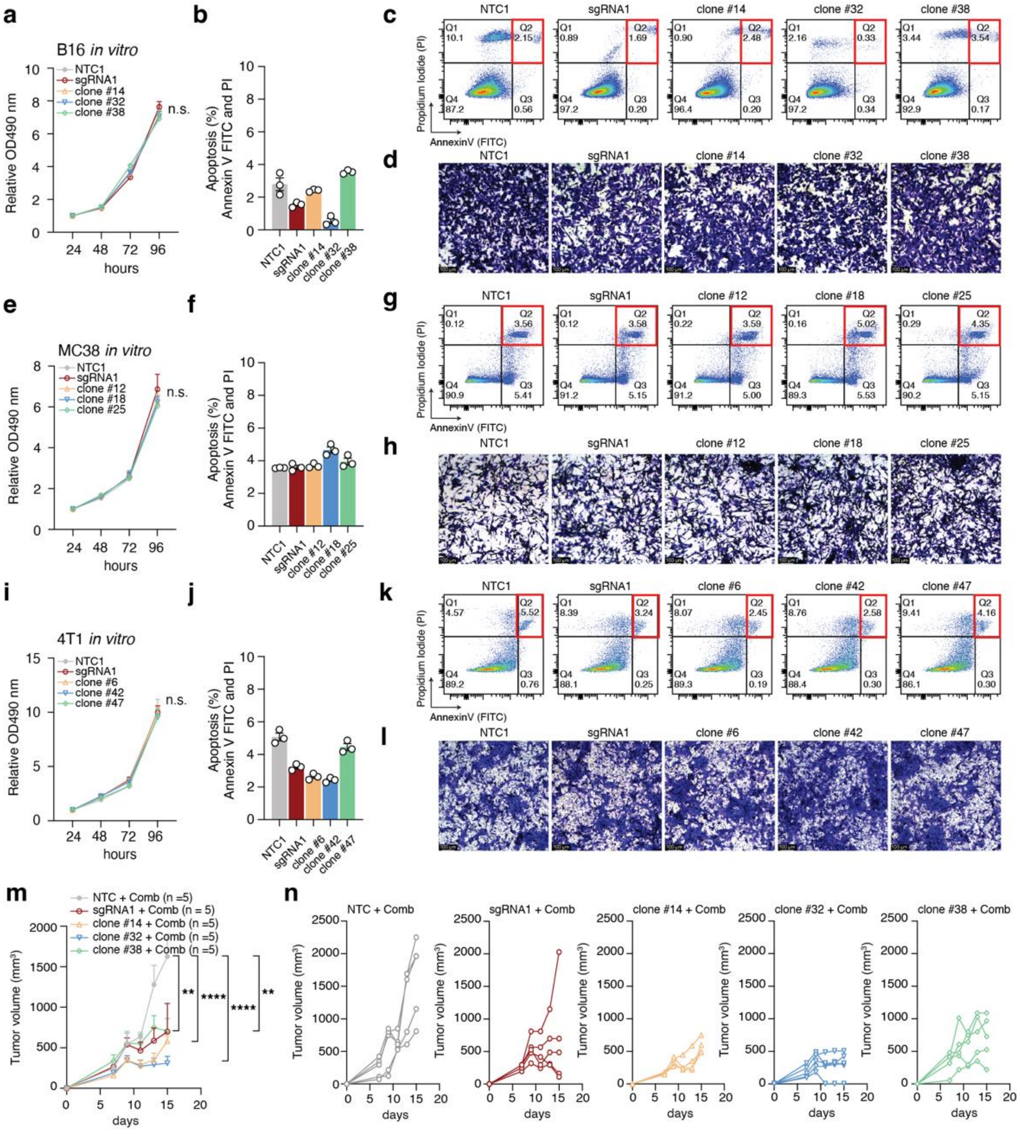

Rebuttal Figure 2. Homozygous miR-25 knockout fully recapitulates the phenotypes observed in pooled knockouts in vitro and in vivo
(a) In vitro proliferation of B16F10 cells measured by MTS assay over 96 hours. Data represent mean ± SEM (n = 6) and were analyzed by two-way ANOVA.
(b, c) Flow-cytometric analysis of apoptosis in B16F10 cells using Annexin V–FITC and propidium iodide (PI) staining. FITC $^+$ PI $^+$ double-positive cells were defined as late apoptotic (Q2).
(d) Representative crystal-violet staining of cell migration in the indicated B16F10 cells. Scale bars, 100 µm.
(e) In vitro proliferation of MC38 cells measured by MTS assay.
(f, g) Apoptosis analysis of MC38 cells as described above.
(h) Representative crystal-violet staining of MC38 cell migration.
(i) In vitro proliferation of 4T1 cells measured by MTS assay.
(j, k) Apoptosis analysis of 4T1 cells.
(l) Representative crystal-violet staining of 4T1 cell migration.
*(m) Tumor growth curves of B16F10 tumors derived from control (NTC1), pooled knockout (sgRNA1), and three homozygous knockout clones (#14, #32, #38) following GVAX + anti-PD-1 combination therapy. Data are shown as mean ± SEM (n = 5 mice per group). Statistical significance was determined by two-way ANOVA (**P < 0.01; ****P < 0.0001).*
(n) Individual tumor growth plots corresponding to panel (m).

(3) Viability of miR-25 knockout in human cells

We further tested whether homozygous miR-25 knockout affects human cell viability. Both pooled and single-clone knockouts in A375 showed no measurable change in proliferation, apoptosis, or migration **(Rebuttal Figs. 3a-3d)**.

We also generated miR-25 knockouts in additional human cancer cell lines (HCT116, HT29, MDA-MB-231, A549) and in HEK293T cells. In all cases, sgRNAs achieved adequate editing efficiency and significantly reduced mature miR-25-3p expression **(Rebuttal Figs. 3e-3f)**. None exhibited detectable viability or proliferation defects **(Rebuttal Figs. 3g-3l)**, indicating miR-25 knockout is tolerated in human cell lines. These data are now presented in current Figs. 6C-6F and S6B-S6J.

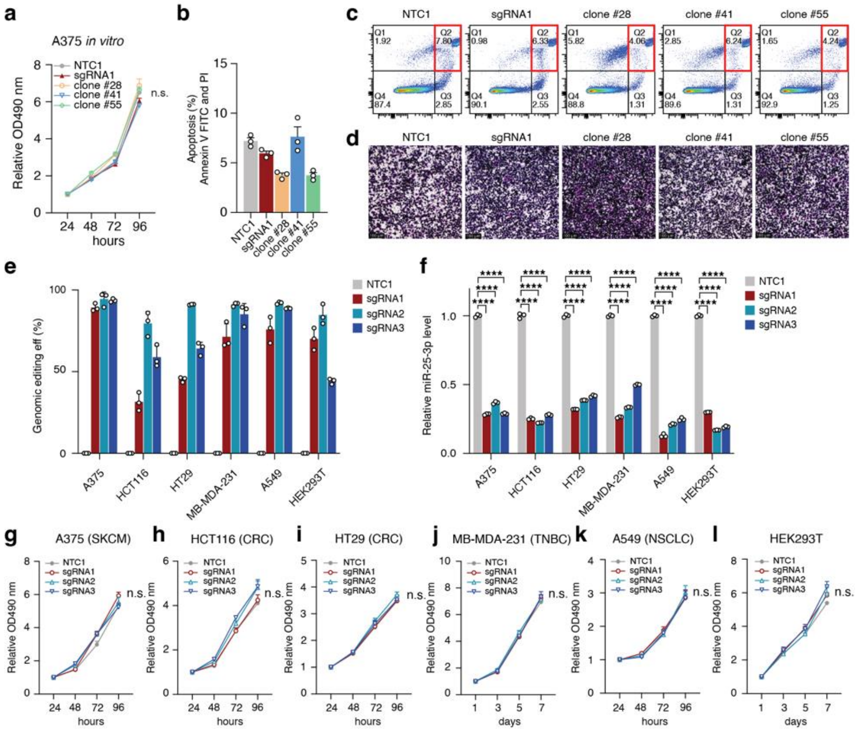

Rebuttal Figure 3. Homozygous and pooled miR-25 knockouts are well tolerated in human cancer and non-cancer cell lines
(a) In vitro proliferation of A375 cells measured by MTS assay over 96 hours. Data represent mean ± SEM (n = 6) and were analyzed by two-way ANOVA.
(b, c) Flow-cytometric analysis of apoptosis in A375 cells using Annexin V–FITC and propidium iodide (PI) staining. $FITC^+ PI^+$ double-positive cells were defined as late apoptotic (Q2).
(d) Representative crystal-violet staining showing cell migration of the indicated A375 clones. Scale bars, 100 µm.
(e) Predicted CRISPR editing efficiency (TIDE analysis) in A375 (SKCM), HCT116 (CRC), HT29 (CRC), MDA-MB-231 (TNBC), A549 (NSCLC), and HEK293T (human embryonic kidney) cell lines.
*(f) RT–qPCR analysis of mature miR-25-3p expression in the indicated cell lines transduced with different sgRNAs. Data represent mean ± SEM (n = 3). Statistical significance was determined by two-tailed Student's t-test (***P < 0.001; ****P < 0.0001).*
(g–l) In vitro proliferation of the indicated human cell lines measured by MTS assay over 96 hours. Data represent mean ± SEM (n = 6) and were analyzed by two-way ANOVA.

Together, these data demonstrate that homozygous loss of miR-25 is viable, stable, and phenotypically neutral in both murine and human cells. The consistent viability of homozygous clones across multiple models excludes the possibility of reversion or intolerance. Therefore, the phenotypic differences observed between miR-25–low and miR-25–high TCGA-SKCM-TM tumors **(main-text Figs. 1E, 3B, 3C)** are best explained by microenvironmental remodeling and immune modulation, rather than by intrinsic effects on cancer cell survival. We have also updated the manuscript discussion section (lines 671-691) to include an expanded description and discussion of the phenotypic consequences of miR-25 loss in cancer cells.

(2) My second concern was that the previous version of the manuscript reported that knockout of miR-106a resulted in the same phenotype as knockout of miR-25, despite the authors' own data showing that miR-106a is expressed at levels 10,000-fold lower than miR-25 in these cells. This raised the strong possibility that the observed phenotype was not due to loss of miRNA expression. In the revised manuscript, the authors acknowledge that miR-106a is barely expressed and have removed references to those experiments. However, this does not explain why knockout of a barely expressed miRNA would yield the same phenotype as knockout of a highly expressed paralog.

Thank you for raising this important point. In the revised manuscript, we have removed all miR-106a knockout experiments and no longer rely on miR-106a to support any conclusion. The current study now focuses exclusively on miR-25, and none of our mechanistic or phenotypic claims depend on miR-106a.

We agree that miR-106a is expressed at much lower levels than miR-25 in our small RNA-seq data, with read counts of approximately 10 **(Rebuttal Fig. 4a)**. In the previous version of the manuscript, miR-106a knockout appeared to partially phenocopy miR-25 knockout, which was indeed confusing. We appreciate the opportunity to clarify the likely basis for this observation.

First, as detailed in our responses to Points 1 and 4, we rigorously validated the specificity of our CRISPR–Cas9 miR-25 knockout using both (i) independent homozygous single-cell clones and (ii) a miR-25 rescue experiment that fully reversed the phenotype. These results demonstrate that the observed phenotype is specifically attributable to loss of miR-25 rather than to off-target editing or unintended disruption of other miRNAs.

Quantitative analyses indicate that the expression difference between miR-25 and miR-106a is variable and does not consistently reach a ~10,000-fold magnitude, for the following reasons:

(1) Small RNA-seq read counts do not directly reflect true biological miRNA abundance.

Although miR-106a appears at comparatively low read counts in our bulk small RNA-seq dataset **(Rebuttal Fig. 4a)**, sequencing-based abundance is strongly influenced by sequence- and structure-

dependent adapter ligation efficiency, PCR saturation, and overall library composition. Therefore, read counts do not represent an absolute intracellular copy number (Jayaprakash et al., Nucleic Acids Res., 2011; PMID: 21890899; Zhuang et al., Nucleic Acids Res., 2012; PMID: 22241775; Raabe et al., Nucleic Acids Res., 2014; PMID: 24198247). Consistent with this limitation, RT–qPCR analysis in B16 tumors shows that miR-106a is readily detectable with Ct values only modestly higher than those of miR-25, indicating a far smaller abundance difference than suggested by sequencing alone **(Rebuttal Fig. 4b)**.

(2) TCGA-SKCM analysis reveals lower but variable expression of miR-106a.

miR-106 has been described as functionally conserved across species (Meenhuis et al., Blood, 2011; PMID: 21628417). We have already provided extensive validation of miR-25 conservation and function **(main-text, Fig. 6)**. We therefore focused our comparative analysis on human miR-25 and miR-106a, for which sufficient sample numbers are available.

In the TCGA-SKCM-TM cohort, miR-25 expression ranges approximately from $\log_2$ 12–18, whereas miR-106a ranges from $\log_2$ 0–10 **(Rebuttal Fig. 4c)**. A true 10,000-fold difference would require a $\log_2$ gap of 13.3 units. Using within-sample comparisons, miR-25 consistently exceeded miR-106a across all tumors with a median difference of 9.4 $\log_2$ units (680-fold), with substantial inter-tumoral variability **(range: 3.8–14.5 $\log_2$ units; Rebuttal Fig. 4d)**. A uniform 10,000-fold difference (13.3) was not observed. These data indicate that miR-106a is substantially, but variably, lower than miR-25 in human melanoma samples, highlighting pronounced inter-tumoral heterogeneity.

Consistent with this variability observed in human tumors, the low apparent miR-106a read counts in our bulk tumor small RNA-seq dataset likely reflect not only its comparatively lower expression, but also sample-dependent variation and cellular heterogeneity. Bulk tumor profiling represents a mixture of tumor cells, immune cells, and stromal components, which may further dilute miR-106a signals relative to miR-25. Taken together, these analyses demonstrate that although miR-106a is consistently expressed at lower levels than miR-25, its relative abundance varies substantially across tumors, and bulk sequencing measurements reflect both biological heterogeneity and cellular composition rather than a uniform, fixed expression difference.

(3) miR-106a may be biologically relevant in melanoma, but through a mechanism distinct from miR-25.

Despite its lower apparent abundance, miR-106a expression significantly stratifies overall survival **(Rebuttal Fig. 4e)** in the TCGA-SKCM cohort and is associated with a coherent transcriptional program enriched for epidermal differentiation, keratinization, and extracellular matrix organization **(Rebuttal Fig. 4f)**. These data suggest that miR-106a may play a distinct role in melanoma biology, separate from the immune-regulatory function of miR-25.

However, we note that this interpretation is based on correlative analyses, and comprehensive functional validation—including single-clone knockout, rescue experiments, single-cell RNA-seq, and detailed mechanistic studies—was not performed in the current manuscript. We originally examined miR-106a to comprehensively survey the miR-17~92 family. We acknowledge that inclusion of these data without deeper validation in the first version of the manuscript was premature, and we apologize for this oversight.

Taken together, these orthogonal lines of evidence support that the phenotypes reported in the revised manuscript are specifically driven by miR-25 loss. miR-106a is no longer used to support any mechanistic conclusion, and any potential role of miR-106a in melanoma is now clearly separated from the central focus of this work.

a

Supplemental table 1. small RNA-seq

	baseMean	log2FoldCh	lfcSE	stat	pvalue	padj
Mir106a	11.12274	1.498116	0.983037	1.523967	0.127517	0.637644
Mir25	20410.34	-1.824178	0.378522	-4.819215	1.44E-06	0.000297

b

B16 tumor#	miR-25 (Ct)	Average miR-25 (Ct)	U6 (Ct)	U6 (Ct)
Tumor 317	19.65	19.63	12.28	12.27667
	19.62		12.27	
	19.62		12.28	
Tumor 318	19.72	19.23666667	10.69	10.68
	18.72		10.69	
	19.27		10.66	
Tumor 319	19.63	19.62333333	11.34	11.39333
	19.58		11.42	
	19.66		11.42	
Tumor 320	21.51	21.42333333	13.31	13.36
	21.33		13.42	
	21.43		13.35	
Tumor 321	20.9	20.87333333	12.21	12.24667
	20.85		12.27	
	20.87		12.26	

B16 tumor#	miR-106a (Ct)	Average miR-106a (Ct)	U6 (Ct)	U6 (Ct)
Tumor 317	21.04	21.03666667	12.28	12.27667
	21.05		12.27	
	21.02		12.28	
Tumor 318	20.28	20.26666667	10.69	10.68
	20.25		10.69	
	20.27		10.66	
Tumor 319	21.15	21.15666667	11.34	11.39333
	21.09		11.42	
	21.23		11.42	
Tumor 320	21.65	21.65	13.31	13.36
	21.69		13.42	
	21.61		13.35	
Tumor 321	22.52	22.53	12.21	12.24667
	22.53		12.27	
	22.54		12.26	

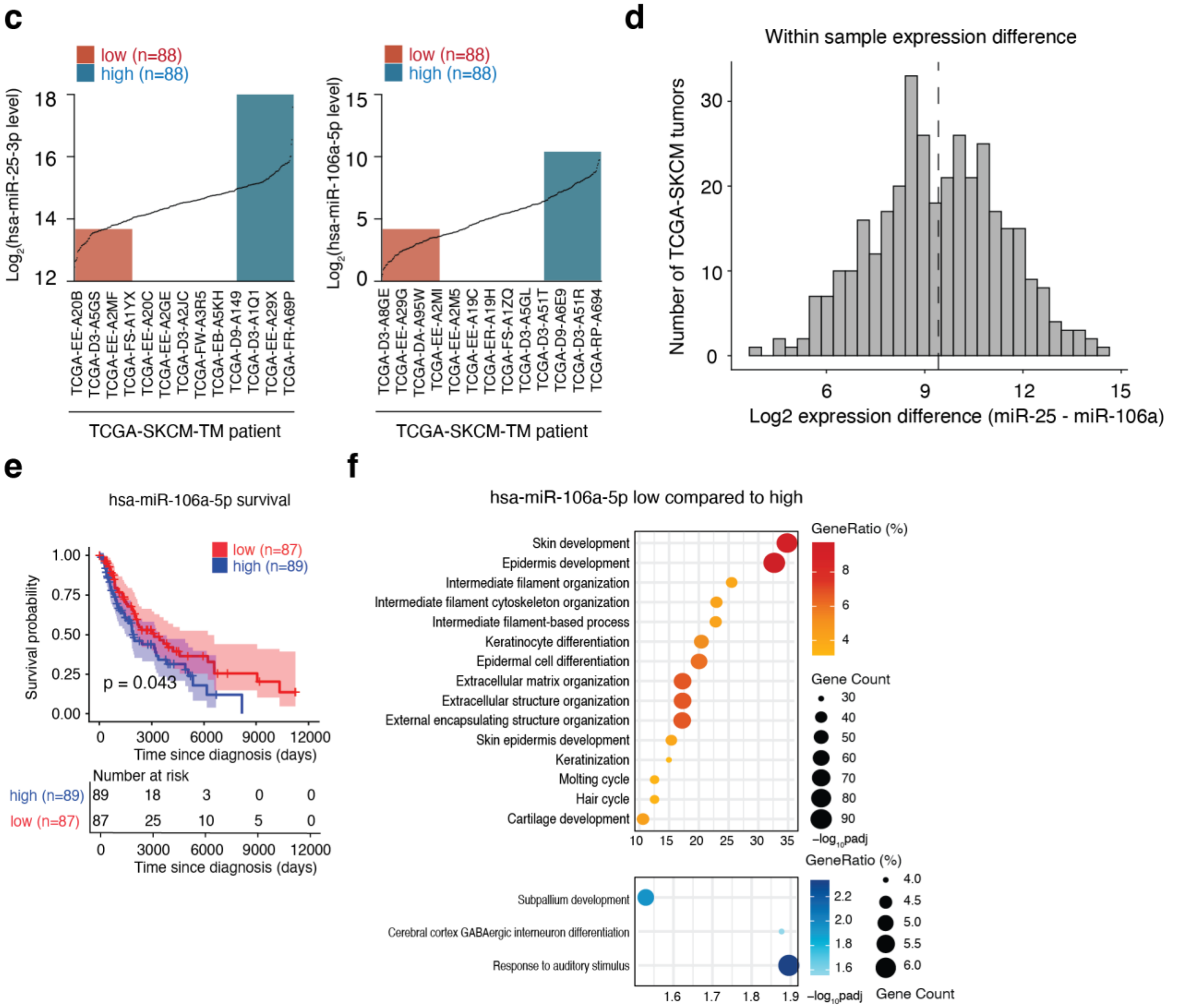

Rebuttal Figure 4. Reassessment of miR-106a expression and biological relevance in melanoma

(a) Small RNA-seq quantification of miR-25 and miR-106a in B16 tumors (combination treatment vs control; data from Supplementary Table 1).
(b) RT–qPCR Ct values for miR-25 and miR-106a in independent B16F10 tumors. U6 was used as an internal control.
(c) Expression distribution of miR-25-3p and miR-106a-5p across TCGA-SKCM metastatic tumors (n = 352 patients).
(d) Distribution of within-sample log_2 expression differences between miR-25 and miR-106a (miR-25 − miR-106a) across TCGA-SKCM metastatic tumors.
(e) Kaplan–Meier overall survival analysis of TCGA-SKCM patients stratified by high versus low miR-106a-5p expression. P values were determined using the log-rank (Mantel–Cox) test.
(f) Gene ontology (GO) enrichment analysis comparing tumors with low versus high miR-106a-5p expression, revealing biological programs distinct from those associated with miR-25.

(3) A third concern was that RNA-seq data from the miR-25 knockout cell lines showed no evidence of preferential upregulation of predicted miR-25 targets. This type of analysis is the gold standard for assessing loss or gain of miRNA function, and the absence of coherent changes in target mRNA expression is a red flag. In their rebuttal, the authors agree that the RNA-seq data do not show de-repression of miR-25 targets but argue that this is expected because "miRNA-mediated repression mostly occurs through translation," citing two 2008 Nature papers by the Rajewsky and Bartel groups. This is incorrect. Both papers demonstrate that while translational inhibition can contribute to target repression, mRNA destabilization is the primary mechanism of miRNA-mediated gene silencing. For instance, the Baek paper states: "…our results show that mRNA destabilization can explain most of the endogenous miR-223-mediated repression." This was further confirmed in a later Molecular Cell paper ("mRNA destabilization is the dominant effect of mammalian microRNAs by the time substantial repression ensues"; Eichhorn et al., PMC4292926). The abstract of this paper clearly states: "…regardless of the miRNA, cell type, growth condition, or translational state, mRNA destabilization explains most (66% to >90%) miRNA-mediated repression," and "…[these results] also simplify future studies, dramatically extending the known contexts and time points for which monitoring mRNA changes captures most of the direct miRNA effects." Therefore, as I stated in my original review, the lack of target mRNA de-repression in miR-25 KO cells suggests that the knockout was ineffective, that miR-25 levels were too low to be functionally relevant in this cell line, or that other paralogs compensate for miR-25 loss.

Thank you for the feedback and explanation of microRNA-directed mRNA regulation. We apologize for the oversight and agree that mRNA destabilization is the dominant mechanism for mammalian microRNA-mediated repression of gene expression. We have included a reference in the text that discusses miRNA biogenesis, evolution and mechanisms (main-text reference #7; PMID: 29570994). Revised text from line 101 - 107: Mature miRNAs are subsequently loaded onto the Argonaute 2 (Ago2) protein to form the miRNA-induced silencing complex (miRISC), which binds to untranslated regions (UTR) of target mRNAs to promote mRNA degradation, which is the primary mechanism for gene silencing by miRNAs.

As noted in our responses to Points 1 and 4 above, we have now validated the specificity of our CRISPR–Cas9 miR-25 knockout using both (i) homozygous single-cell clones and (ii) a miR-25 rescue experiment that fully reversed the phenotype. These data demonstrate that the observed phenotype is specifically attributable to the loss of miR-25, not to off-target editing or loss of other miRNAs.

In addition, miR-25 is expressed at high and functionally meaningful levels in B16 cells. As noted in Point 3, while miR-106a is extremely low, miR-25 reaches an average of 20410.34 counts in our small RNA-seq dataset (Supplementary Table 1), excluding insufficient abundance as a cause for the absence of a cumulative-distribution shift. We fully agree, however, that other paralogs may compensate for miR-25 loss as shown in our results presented in Fig 5M. Below, we address the cumulative-shift question in three parts.

(1) Compensatory effects among seed-sharing miRNAs explain the absence of cumulative-shift curves

Family redundancy among seed-sharing miRNAs is extensively documented. As highlighted by Victor Ambros (Ambros, Curr. Biol. 2010; PMID: 20656201): "**What happens when one deletes the gene for a**

worm microRNA other than lin-4 or let-7? Practically nothing… Part of the reason for the apparent irrelevance of an individual microRNA gene is family redundancy… simultaneous loss of two or more members of the let-7 family can cause severe defects." This is further supported by Ventura et al. (Cell, 2008; PMID: 18329372), who demonstrated that miRNAs with identical seeds compensate for one another at the functional level. The miR-17~92 / miR-106b~25 / miR-106a~363 clusters are canonical examples of such redundancy.

Early indications of compensation came from our endogenous luciferase reporter assay using a firefly luciferase reporter containing the full-length Sdc3 3′UTR **(Rebuttal Fig. 5a)**. In B16 cells with intact miR-25, luciferase activity was reduced by ~90% after IFN-γ incubation, whereas this repression was abolished when the Sdc3 binding site was deleted. Remarkably, in miR-25 knockout cells, luciferase activity remained ~90% lower for the wild-type Sdc3 3′UTR compared to the mutant 3′UTR **(Rebuttal Fig. 5b, yellow and red bars)**, indicating that additional microRNAs may regulate Sdc3.This indicates that additional seed-sharing miRNAs continue to repress Sdc3 in the absence of miR-25. This interpretation is supported by Ago2 RIP-qPCR, showing markedly increased Ago2-bound Sdc3 in the miR-25-KO background at baseline (0 h IFN-γ) **(Rebuttal Fig. 5c, grey and yellow bars)**. This explains why Sdc3 protein rebounds in miR-25-deficient cells without IFN-γ **(main-text Figs. 5B and 5E)** and clarifies the opposite behavior observed during IFN-γ depletion in vivo **(main-text Figs. 4C–4F)**.

To directly test this phenomenon, we performed RNA-seq on three independently derived homozygous miR-25 KO clones **(#14, #32, #38; Supplemental Table 10)**. As the reviewer noted previously, none of these clones displayed preferential derepression of predicted miR-25 targets **(TargetScan 8.0 ≥1 site; 854 genes; Rebuttal Fig. 5d)**. Thus, the lack of cumulative shift is a reproducible biological phenomenon, not a technical artifact or insufficient knockout.

We further examined previously published work. In Eichhorn et al. (Mol Cell, 2014), cumulative shifts were measured under miRNA transfection or knockout of miRNAs without seed-sharing paralogs. In contrast (i) miR-25 is endogenously highly expressed, (ii) deletion is genomic and stable, and (iii) miR-25 shares its seed sequence with five highly expressed paralogs (miR-32/92a/92b/363/367) **(Rebuttal Fig. 5e)**. These differences may explain why cumulative-shift curves seen in Eichhorn et al. do not directly apply to our system. They also induced miR-1 in 3T3 cells (seed-shared with miR-206), which differs fundamentally from a stable CRISPR knockout of a high-abundance miRNA.

Consistent with our observations, Ma et al. (BMC Genomics, 2018; PMID: 297643374; Figure 4c) demonstrated that target mRNA stabilization following knockout is masked when multiple highly expressed paralogs co-target the same transcripts. RT–qPCR in our study confirms robust upregulation of miR-32, miR-92a, miR-92b, and miR-367 in miR-25-KO cells at 0 h IFN-γ, explaining the restoration of Sdc3 levels and the unchanged cumulative distribution. Upon IFN-γ stimulation, miR-32, miR-92a, and miR-367 remain elevated **(Rebuttal Fig. 5f)**, lowering Sdc3 mRNA relative to 0 h, but compensation diminishes by 24–72 h, at which point these paralogs fail to repress Sdc3 **(Rebuttal Fig. 5g)**.

Thus, while we fully agree that mRNA destabilization is the dominant mechanism of miRNA action, our results support this model: Sdc3 mRNA destabilization is clearly mediated by miR-25 under IFN-γ stimulation, and deletion of miR-25 increases Sdc3 mRNA and protein in multiple cancer cell lines **(main-text Fig. 5B–5D and Fig. 6G–K, with IFN-γ stimulation)**.

(2) miR-25 has the highest binding affinity within the family, and paralogs cannot fully compensate under IFN-γ

TargetScan 8.0 predicts that miR-25 has the strongest binding affinity for the Sdc3 3′UTR among all six seed-sharing family members **(Rebuttal Fig. 4e)**. Although these paralogs are upregulated in miR-25-deficient cells, they do not fully restore Sdc3 repression following IFN-γ stimulation. This behavior is fully consistent with McGeary et al, (Science, 2019; PMID: 31806698), who demonstrated that "**binding-site affinity is the major determinant of miRNA-mediated repression.**"

Accordingly, no clear cumulative-distribution shift is observed at baseline because highly expressed family members compensate for miR-25 loss. Under IFN-γ stimulation, however, miR-25 becomes the dominant regulator due to its superior binding affinity, and Sdc3 expression rises sharply in knockout cells **(Rebuttal Fig. 5g)**. This mechanistically explains both the molecular phenotype and the enhanced in vivo immune response observed upon miR-25 deletion.

(3) Base-editing of the endogenous Sdc3 3′UTR phenocopies miR-25 KO and provides definitive mechanistic proof

As David Bartel emphasized (Cell, 2018; PMID 29570994), "**The most definitive approve for isolating and confirming the importance of a particular target is to disrupt the miRNA-binding site(s) within the endogenous target gene and examine the extent to which the resulting de-repression of that target phenocopies the miRNA knockout.**" Accordingly, we used ABE8e to mutate the Sdc3 miR-25 binding motif (A→G at positions 3 and 9). Editing efficiency was high **(Rebuttal Fig. 5h–5i)**, and both Sdc3 mRNA and protein increased under IFN-γ stimulation **(Rebuttal Fig. 5j)**. In vivo, these Sdc3-UTR–edited tumors responded earlier and with comparable magnitude to miR-25 KO under combination therapy **(Rebuttal Fig. 5k–5l)**.

This confirms that:
(i) miR-25 represses Sdc3 in an IFN-γ–dependent manner,
(ii) paralog compensation delays but does not eliminate the functional consequences of miR-25 loss, and
(iii) the miR-25–Sdc3 3′UTR interaction is the primary regulatory axis driving the phenotype.

Collectively, these results demonstrate that despite the absence of large-scale mRNA target derepression, miR-25 knockout is complete, functional, and biologically meaningful. The cumulative-curve behavior is fully explained by paralog compensation and binding-affinity hierarchy, rather than insufficient editing or low miR-25 expression.

These data are now presented in the current Figs. 4O, 4P, 5A, 5L–5Q, and S5A, S5G–S5H. We have also updated the manuscript (lines 679–691) to include a detailed description and discussion of the compensatory effects observed among miR-25 family members.

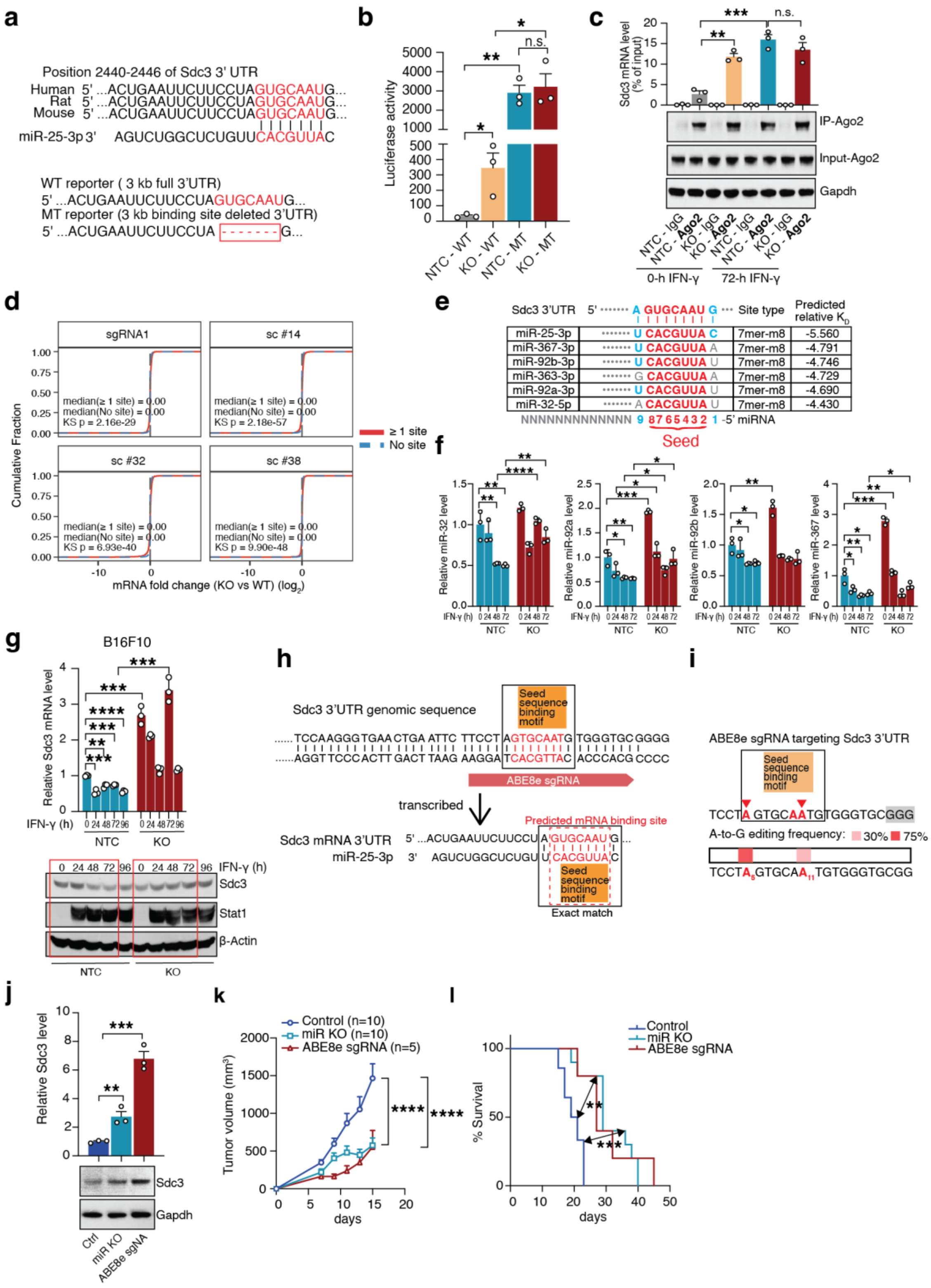

Rebuttal Figure 5. Paralog compensation masks cumulative-curve shifts but miR-25 becomes the dominant regulator of Sdc3 under IFN-γ stimulation

(a) Predicted miR-25 binding site within the Sdc3 3'UTR, as identified by TargetScan. Below: schematic of the DNA fragment cloned into the luciferase reporter plasmid, showing the wild-type (WT) sequence and the mutant (MT) sequence with the miR-25 binding site deleted.
*(b) Firefly luciferase activity in B16F10 cells transfected with the constructs described in (a). Data are mean ± SEM (n = 3). P-values: *P < 0.05; **P < 0.01 by two-tailed Student's t-test.*
*(c) RNA immunoprecipitation (RIP) analysis in B16F10 cells. Top: RT–qPCR quantification of Sdc3 transcripts in anti-Ago2 versus control IgG immunoprecipitates. Bottom: immunoblot of Ago2 in the corresponding RIP samples. Data are mean ± SEM (n = 3). P-values: **P < 0.01; ***P < 0.001 by Student's t-test.*
(d) Cumulative distribution plots of mRNA fold-changes (KO vs WT) for predicted miR-25 target genes in B16 miR-25 knockout clones. Genes with ≥1 predicted miR-25 site (n = 854) were compared with control genes lacking any site (n = 46,793). Fold-changes were normalized to the median expression of control genes.
(e) Canonical binding sites of miR-25-3p, miR-32-5p, miR-92b-3p, miR-367-3p, miR-363-3p, and miR-92a-3p within the Sdc3 3'UTR. The 6-nt seed region (positions 2–7) and additional pairing to nucleotide 8 are highlighted in red; flanking base pairing is shown in blue. Family members are ordered by predicted binding affinity.
*(f) RT–qPCR analysis of miR-32, miR-92a, miR-92b, and miR-367 expression in NTC and miR-25-KO B16 cells following IFN-γ stimulation (0–72 h). Data are mean ± SEM (n = 3). P-values: *P < 0.05; **P < 0.01; ***P < 0.001 by Student's t-test.*
*(g) Top: immunoblot analysis of Sdc3 protein in B16F10 cells at indicated time points following IFN-γ treatment. Bottom: RT–qPCR quantification of Sdc3 mRNA from matching samples. Data are mean ± SEM (n = 3). P-values: **P < 0.01; ***P < 0.001; ****P < 0.0001 by Student's t-test.*
(h) Schematic of the ABE8e adenine base-editing strategy targeting the Sdc3 3'UTR seed-binding motif within the genomic locus.
(i) Schematic of base-editing design and editing efficiency of the ABE8e sgRNA targeting the Sdc3 3'UTR. A-to-G editing frequencies at predicted positions are indicated.
*(j) Top: RT–qPCR quantification of Sdc3 mRNA in B16F10 cells transduced with the indicated sgRNAs after 72 h of IFN-γ treatment. Bottom: representative immunoblot of Sdc3 protein in the same samples. Data are mean ± SEM (n = 3). P-values: **P < 0.01; ***P < 0.001 by Student's t-test.*
*(k) In vivo tumor growth curves of B16 tumors treated with combination immunotherapy (GVAX + anti-PD-1). Data are mean ± SEM for each group. ****P < 0.0001 by two-way ANOVA.*
*(l) Kaplan–Meier survival curves corresponding to (k). P-values: **P < 0.01; ***P < 0.001 by log-rank (Mantel–Cox) test.*

(4) Finally, in light of these concerns, I requested that the authors perform a rescue experiment by reintroducing ectopic miR-25 into the miR-25 KO cells. In their rebuttal, the authors claim that this experiment cannot be performed because the sgRNAs remain expressed. This explanation is unconvincing. Transient expression of the sgRNAs or Cas9 could easily be used, or the mature miR-25 sequence could be expressed from a pri-miRNA backbone resistant to the sgRNAs.

We agree that in our first-round revision, our technical approach was limited, and we appreciate the reviewer's suggestion to directly validate the specificity of the CRISPR–Cas9 miR-25 knockout. In the revised study, we have now performed the requested rescue experiment.

To address this concern, we first generated miR-25 knockout B16 cells using transient CRISPR editing, achieved by electrotransfection of Cas9–sgRNA RNP complexes, followed by isolation of single-cell clones as described in Point 1. This strategy ensured that neither Cas9 nor sgRNAs remained expressed, thereby permitting functional rescue.

For miR-25 re-expression, we cloned the genomic miR-25 sequence—including ~100–200 bp of flanking regions—into the pLVX-IRES-puro lentiviral vector. Transduction of this construct resulted in robust miR-25 overexpression (~15-fold increase in B16 WT cells) and successful restoration of miR-25 expression in the miR-25 knockout single clone #38 **(~6-fold increase in the rescue group; Rebuttal Fig. 6a)**.

Importantly, re-expression of miR-25 did not alter in vitro proliferation, as all four groups exhibited comparable growth rates **(Rebuttal Fig. 6b)**. However, when transplanted into mice and treated with GVAX plus anti–PD-1, re-expression of miR-25 fully restored tumor growth to levels indistinguishable from the control group **(Rebuttal Figs. 6c–6e)**. Thus, the tumor growth inhibition observed in miR-25 knockout tumors is specifically attributable to loss of miR-25 rather than off-target effects of CRISPR editing.

These data are now included in manuscript Figure S1 and Supplementary Table 4.

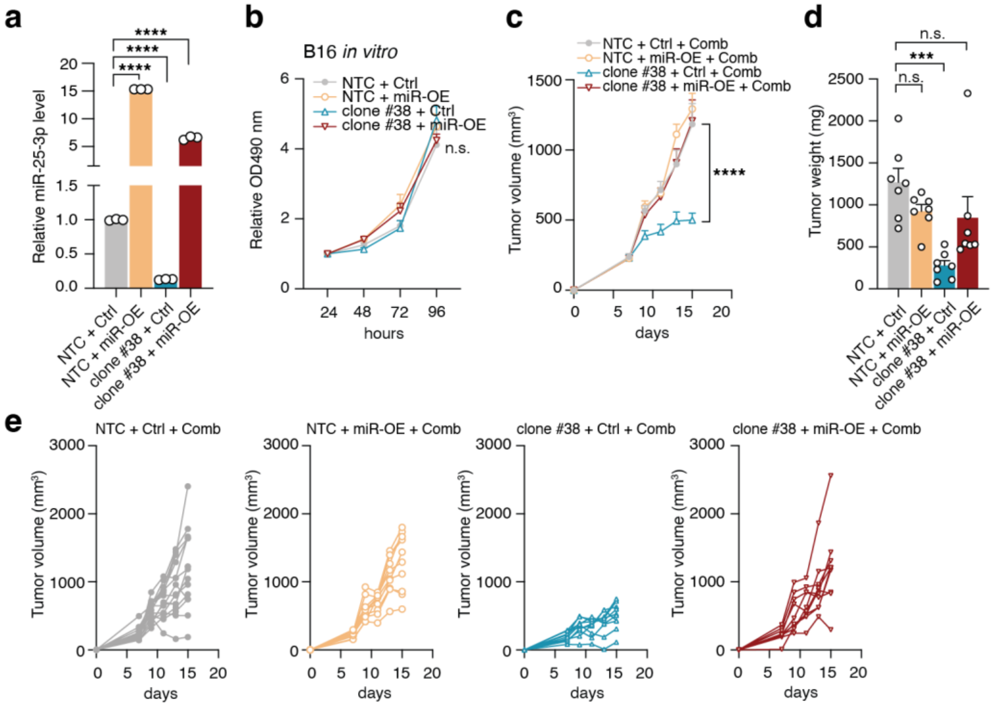

Rebuttal Figure 6. miR-25 re-expression and combination therapy experiments in B16 tumors.
*(a) RT–qPCR quantification of miR-25-3p expression in NTC and miR-25 KO clone #38 cells stably transduced with control or miR-25 overexpression (OE) lentivirus. ****P < 0.0001, by Student's t-test.*
(b) In vitro proliferation of B16 NTC and clone #38 cells following miR-25 overexpression measured by MTS assay. Data are presented as mean ± SEM, n = 6, analyzed by two-way ANOVA.
*(c) Tumor growth curves of B16 NTC and clone #38 cells treated with GVAX plus anti–PD-1 combination therapy (Comb) with or without miR-25 overexpression. Data are presented as mean ± SEM; n = 10–15 mice per group. ****P < 0.0001, two-way ANOVA.*
*(d) Tumor weights on day 15 from (c). Data are presented as mean ± SD (n = 7) per group. *** P < 0.001 by Student's t-tests. Each dot represents one mouse.*
(e) Individual tumor growth for each treatment group corresponding to panel (c).

In conclusion, the lack of mRNA target destabilization, the unexplained phenotypic similarity between miR-106a and miR-25 knockouts, the absence of de-repression of miR-25 targets, and the failure to perform rescue experiments leads me to conclude that as it stands the authors' interpretation is not sufficiently supported by the experimental evidence provided.

With our homozygous knockout validation, comprehensive analyses of mRNA target validation, clarification of functional redundancy among microRNAs, and successful completion of rescue experiments, we respectfully invite the reviewers to reconsider their conclusion. We believe that these additional data and analyses substantially strengthen our study and now provide robust experimental evidence supporting our interpretation.

Reviewer #2 (Remarks to the Author):

While the practical means targeting miR-25(other than genetic manipulations of its expression in tumor cells) remain to be identified (if at all possible), the authors have addressed most of my other concerns by restructuring the manuscript and by providing new data. Their findings of miR-25 as a major driver of therapeutic resistance to anti-PD-1 and as a TME-remodeler are worth being considered for publication in Nature Communications. I have no other concerns.

We thank the reviewer for the thoughtful and encouraging comments during the review process. Your feedback has greatly improved the clarity and impact of our work. We are currently designing practical gene-editing strategies aimed at targeting either SDC3 or members of the miR-25 family to further explore their therapeutic potential.

Reviewer #3 (Remarks to the Author):

Overall, the authors have adequately addressed most of our previous concerns. However, there are some outstanding concerns that can be addressed mostly through editing of the manuscript.
1. The wording in the text suggests that the activation of antigen-receptor in M1-like macrophages (line 331) precedes and drives complement activation and humoral immunity in CAFs. Authors present evidence that both are occurring, and CellChat suggests that macrophages and fibroblasts are interacting, but the data does not demonstrate which activation event occurs first and whether it is necessary or sufficient to drive the second activation event. Examining the effect liposome clodronate treatment had on CAFs would strengthen this connection. Otherwise, the authors should temper the definitive tone of these conclusions.

We fully agree with the reviewer that our current CellChat analysis does not establish the temporal sequence or causal relationship between M1-like macrophage activation and complement activation in CAFs. We also recognize that this limitation arises in part from the scope of the CellChat database and the broader knowledge gaps regarding innate immune regulation of complement. To examine this more carefully, we conducted an in-depth review of the CellChat ligand–receptor database. Within the COMPLEMENT signaling pathway, C3 is the only annotated ligand (sender), interacting with receptors such as C3AR1, CR2, ITGAM–ITGB2, and ITGAX–ITGB2 (receivers). For MHC-II–associated interactions, which we use as markers of M1-like macrophage activation, the CellChat database lists only two ligand–receptor pairs: H2-OB–CD4 and H2-DMB2–CD4. Full CellChat lists are provided in **Supplementary Table 8**. These findings emphasize that CellChat does not comprehensively capture innate–adaptive immune transitions or the full repertoire of macrophage–CAF communication pathways relevant to our model.

To assess whether complement activation in CAFs depends on macrophages, we analyzed tumor sections from mice treated with liposome clodronate (LC). In miR-25-knockout tumors, C3 staining (particularly within the intercellular regions between nuclei and the pale cytosolic tumor cells) was markedly diminished after LC treatment, returning to levels comparable to NTC + PBS and NTC + LC controls. These results indicate that macrophages are required for CAF-mediated C3 activation in our system. We have updated manuscript Figure 3M to provide an improved visualization of complement deposition **(Rebuttal Fig. 7)**.

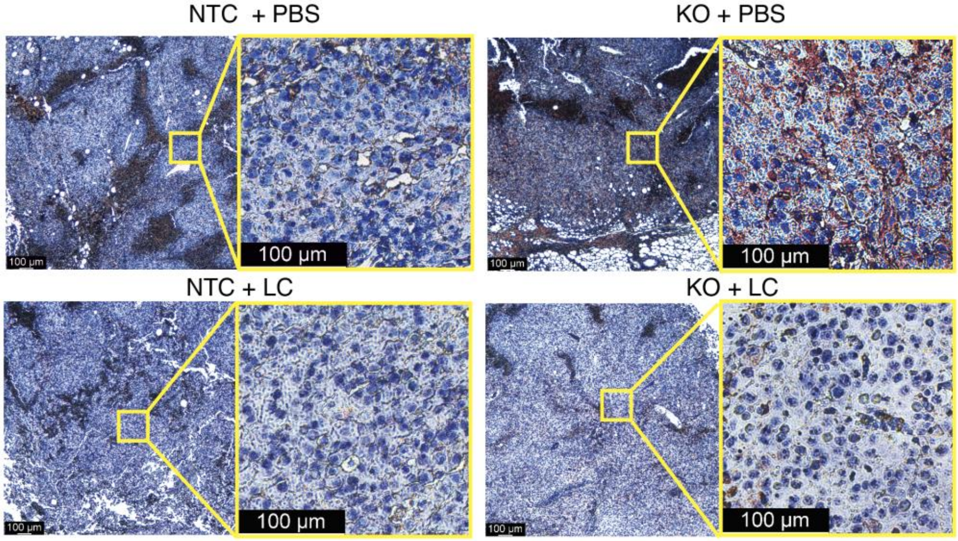

Rebuttal Figure 7. Immunohistochemical (IHC) staining of complement C3 in B16 tumors treated with liposome clodronate in combination with anti-PD-1 + GVAX.
Representative tumor sections from non-targeting control (NTC) and miR-25 knockout (KO) tumors treated with either PBS or liposome clodronate (LC), in combination with anti-PD-1 + GVAX. C3 staining (brown) indicates complement activation. Insets show high-magnification regions (yellow boxes) corresponding to the areas outlined in the low-magnification panels. Scale bars, 100 µm.

2. In Figure 3 the % of macrophages in the KO+LC group is equivalent to the percent in the KO+PBS in Figure 3L, yet Figure 3M shows a marked reduction of C3 in the KO+LC. There could be a phenotypic change in macrophage polarization due to LC treatment that alters complement activation in the CAFs that should be acknowledged.

Thank you for this insightful comment. Clodronate is encapsulated within liposomes that are selectively phagocytosed by macrophages. Once internalized, clodronate is released into the cytoplasm and induces apoptosis. Non-phagocytic cells (including fibroblasts, endothelial cells, and lymphocytes) are not directly affected. In tumor settings, LC treatment preferentially depletes M2-like tumor-associated macrophages (TAMs), thereby reducing immunosuppression and often increasing $CD8^+$ T-cell infiltration while skewing the immune environment toward an M1-like state (Zeisberger et al., Br J Cancer, 2006; PMID: 16832418).

Our data are consistent with these reports, as macrophage depletion significantly inhibited tumor growth across treatment groups. However, we also observed that LC treatment markedly reduced complement activation in miR-25 knockout tumors as shown in Point 1, despite overall macrophage frequencies appearing similar between KO+LC and KO+PBS in Figure 3L. This suggests that LC may alter the composition or polarization state of the remaining macrophages rather than simply reducing their total numbers.

Based on these findings, we hypothesize that LC-mediated depletion may preferentially eliminate not only immunosuppressive M2 macrophages, but also a subset of antigen-presenting M1-like macrophages that are required to sustain CAF-driven C3 activation and the associated humoral immune response. This interpretation is supported by Chen et al. (Nature Communications, 2018; PMID: 29717137), who showed that LC treatment abrogated the anti-tumor efficacy of chloroquine in B16 melanoma—a response shown to depend on M1 macrophages.

We have now incorporated this consideration into the revised Discussion (manuscript lines 703-713), acknowledging that LC treatment may reprogram or selectively deplete macrophage subsets in a manner that impacts CAF complement activation.

3. My concern with the IFNg blockade in vivo experiments is that the overall reduction in macrophages and the increase in MHC2+ macrophages resulted in increased tumor size in KO+a-IFNg, while previously it was argued that these characteristics helped facilitate the reduction in tumor size upon miR-25 KO in MC38 and B16F10. This seems to suggest that CAF polarization is the most prevalent driver of miR-25 KO. I agree that this indicates CAFs are likely the primary source of C3, but in Figure 4E (like in Figure 3M) the loss of C3 occurs between the KO + Isotype and KO + a-IFNg, while the two conditions possess the same amount of macrophages and MHC2+ macrophages. The (presumed M1-like) MHC2+ macrophages being equivalent between the two groups also suggests that antigen-receptor activation does not precede complement activation in the CAFs, as was mentioned earlier (line 331).

Lastly, in Figure 4K you describe the levels of C3 linked to the changes in TAMs, but the levels in 4J are comparable to 3L, but the C3 staining in 4K is much different than in 3M; it is therefore unclear how authors link C3 staining to macrophage level.

Thank you for these thoughtful comments regarding the effects of IFN-γ blockade, Ago2 depletion, and their relationship to macrophage polarization and complement activation.

In our model, miR-25 knockout increases expression of the tumor membrane protein Sdc3, which promotes activation of M1-like macrophages and subsequently induces CAF-mediated complement activation, while simultaneously weakening the pro-tumor MIF-driven TAM–CAF axis. Because we have already demonstrated that CAF complement activation requires macrophages (as shown in the LC experiment), we interpret the $MHCII^+$ macrophages as an upstream component in this pathway.

Importantly, the abundance of $MHCII^+$ macrophages itself does not change **(main-text Fig. 2C)**; therefore, overall macrophage percentage alone may not directly reflect the level of innate immune activation. Instead, the total macrophage abundance observed across conditions likely represents a

downstream consequence of complement-mediated humoral regulation within the TME **(Rebuttal Fig. 8)**, rather than the initiating immune event.

Regarding the IFN-γ blockade experiment, systemic neutralization of IFN-γ removes all IFN-γ signaling within the tumor microenvironment. This derepresses IFN-γ–regulated tumor membrane proteins (including Sdc3) resulting in an overall increase in MHCII^{+} macrophages **(main-text Fig. 4E, right)**. Among the derepressed proteins, Sdc3 is particularly associated with humoral activation and drives C3 expression in CAFs **(main-text Fig. 4F)**, which subsequently modulates total macrophage abundance **(main-text Fig. 4E, left)**. Consistent with this mechanism, we later confirmed that Sdc3 expression is restored by other miR-25-family paralogs in the absence of IFN-γ **(main-text Figs. 5B and 5E)**, matching the observed phenotype under IFN-γ blockade.

Similarly, Ago2 depletion removes not only miR-25 but also a broad set of functional microRNAs, several of which contribute to antitumor immune activation. Wang et al. (Cell Reports, 2025; PMID: 40106436) reported that Ago2 promotes tumor immune evasion by suppressing CXCL chemokines, ISGs, and HLA molecules. Despite this broad impact, we still observed immunologic changes specifically consistent with miR-25 loss, including enhanced MHCII^{+} macrophage activation **(main-text Fig. 4L, right)**, increased C3 staining **(main-text Fig. 4M)**, and a reduction in total macrophage abundance **(main-text Fig. 4L, left)**. These changes do not necessarily correlate with tumor size but instead highlight a canonical microRNA-mediated regulatory axis through which miR-25 shapes the tumor immune microenvironment.

Together, the IFN-γ blockade and Ago2 depletion studies provide in vivo mechanistic insights into how miR-25 regulates tumor-intrinsic immune signaling. As suggested, we have revised the manuscript to more clearly delineate the order of immune activation events and updated the synopsis figure to reflect these clarifications.

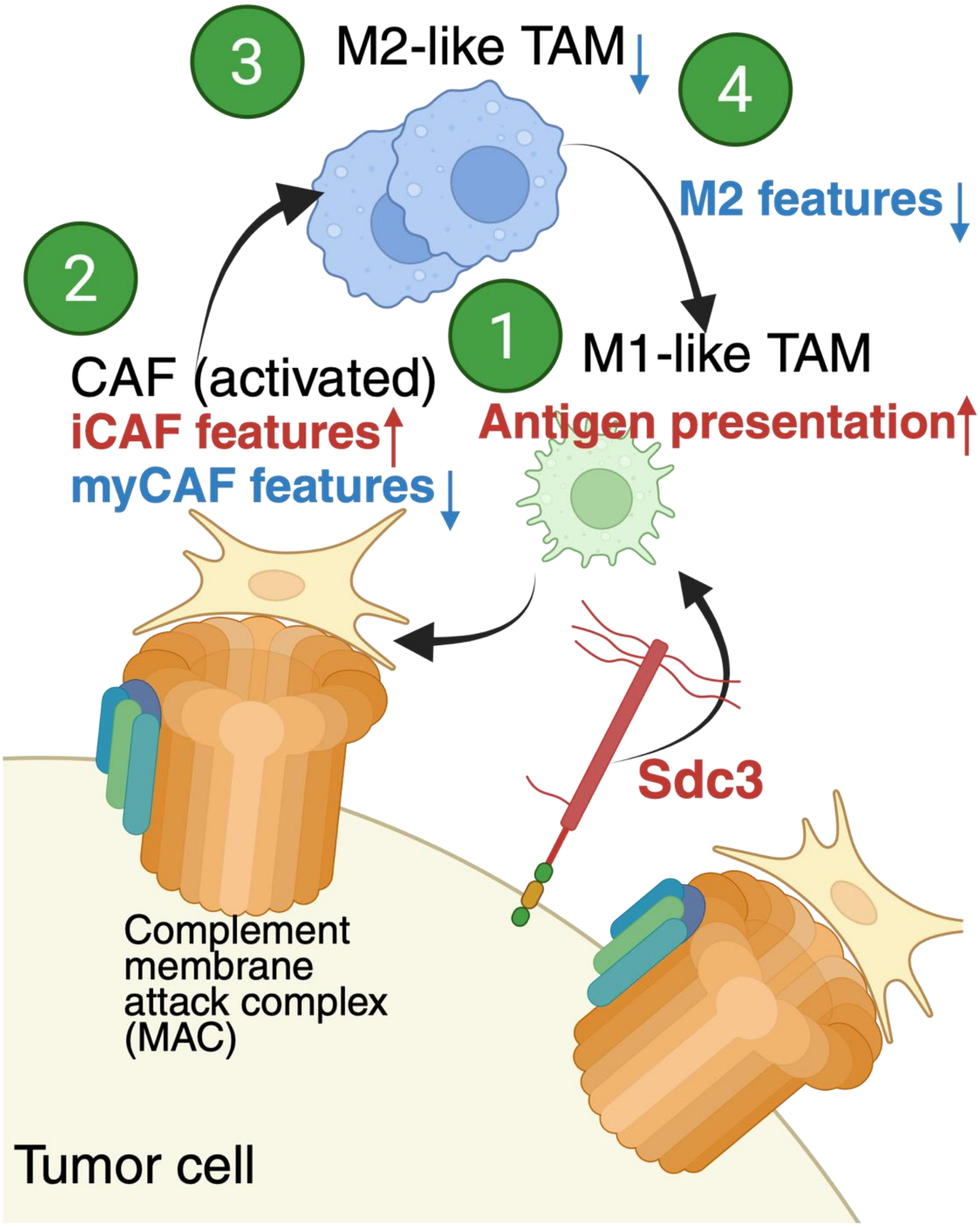

Rebuttal Figure 8. Proposed model of the miR-25–SDC3–CAF–macrophage regulatory axis.
Loss of miR-25 increases Sdc3 expression on tumor cells, enhancing antigen presentation and promoting M1-like macrophage activation. Activated macrophages induce CAF complement activation (C3/MAC), shift iCAF features↑ / myCAF features↓, and suppress M2 macrophage population, thereby strengthening anti-tumor humoral and innate immunity.

4. You could try inputting your particular macrophage and T cell gene signatures into CIBERSORT to see if the specific populations you saw by scRNA-seq are also differentially present in patient's data.

Thank you for this constructive suggestion. We performed the recommended analysis by applying CIBERSORTx to the TCGA-SKCM–TM bulk RNA-seq dataset using a single-cell–derived signature matrix generated from our whole-tumor atlas, which includes tumor, immune, stromal, and endothelial cell populations.

CIBERSORTx deconvolution revealed that low–miR-25 tumors were enriched for several immune populations consistent with an active anti-tumor microenvironment, including follicular helper T cells (C4),

Th1 cells (C6), effector T cells (C8), and Ccl5⁺ NK cells (C10). A particularly strong enrichment was observed for Jchain⁺ B cells (C16), which aligns with the humoral immune activation described in our model. Conversely, melanoma tumor-cell fractions were reduced in the C25 (Gm26917⁺ tumor) and C26 (Cnih4⁺ tumor) clusters **(Rebuttal Figure 9)**.

Together, these results indicate that reduced miR-25 expression in metastatic melanoma is associated with a tumor microenvironment enriched for Th1/cytotoxic and humoral immune populations, consistent with diminished tumor-cell abundance and relief of immune suppression mediated by the miR-25–SDC3 axis. <u>This new analysis is included in manuscript Figure 6A, and the single-cell–derived signature matrix has been added as Supplementary Table 11 in the revised manuscript.</u>

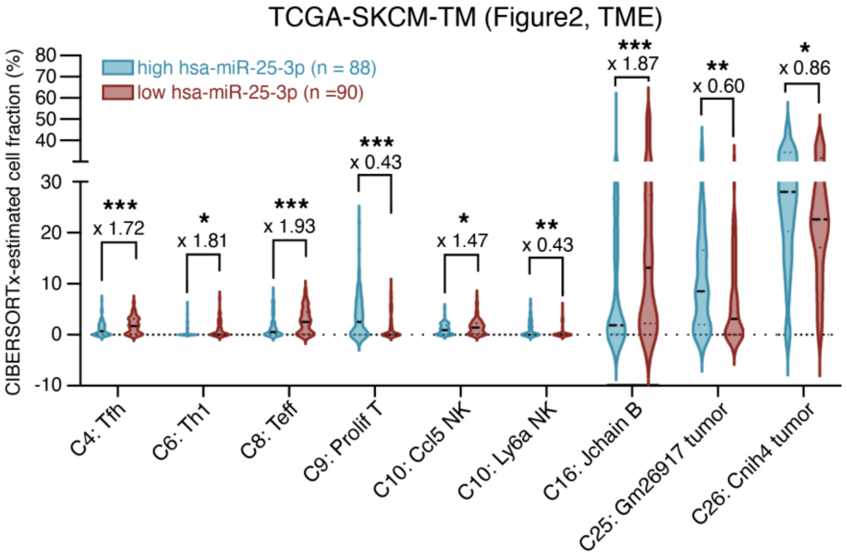

Rebuttal Figure 9. CIBERSORTx analysis of TCGA-SKCM-TM bulk RNA-seq data using a single-cell–derived signature matrix.
*Violin plots showing the relative abundance of indicated cell types from the TCGA-SKCM-TM cohort, comparing tumors with high or low miR-25-3p expression. Cell-type proportions were estimated using CIBERSORTx. * $P < 0.05$, ** $P < 0.01$, *** $P < 0.001$ by Student's t-test.*

5. It would be beneficial to see if there are any published scRNAseq datasets that can demonstrate miR-25 is reduced (or Sdc3 increased) specifically in tumor cells with increased immune infiltration or in responders to PD1/ICT (is this unique to PD1 monotherapy or applicable to CTLA4/LAG3 combo and PDL1 as well?). More evidence that the effects of miR-25 KO or Sdc3 upregulation are relevant in human patient samples or human cell lines would be beneficial. Is there evidence that miR-25 levels are higher during disease progression, or higher in tumor relative to normal tissue? The evidence presented suggests a phenotype in multiple distinct tumor types so it should be possible to find a clinical connection in at least one tumor type. For melanoma immunotherapy transcriptomics and single cell you can check Hugo et al., Cell 2016; Riaz et al., Cell 2017; Gide et al., Cell 2019; Jerby-Arnon et al., Cell 2018; Pozniak et al., Cell 2024.

Thank you for the insightful suggestions. We will answer the question from the two aspects,

(1) Bulk and single-cell RNA-seq evidence for miR-25/SDC3 in immunotherapy datasets

We investigated the datasets recommended by the reviewer. Among the three bulk RNA-seq studies (Hugo et al., Cell 2016; Riaz et al., Cell 2017; Gide et al., Cell 2019), only Riaz et al. (2017; PMID: 29033130) provides microRNA expression data, which we had previously analyzed in our original submission. In this cohort, we observed a potential association between miR-25 expression and complement/innate immune activation **(Rebuttal Fig. 10a)**. However, the limited sample size and mixed

cellular origin intrinsic to bulk RNA-seq prevented this trend from reaching statistical significance between responders and non-responders **(Rebuttal Fig. 10b)**. Likewise, SDC3 expression did not differ significantly between groups, likely because it is expressed in both tumor and immune compartments, obscuring tumor-specific changes in bulk profiles **(Rebuttal Fig. 10c)**.

To overcome these limitations, we focused on two single-cell datasets (Jerby-Arnon et al., Cell 2018; Pozniak et al., Cell 2024) that allow precise separation of malignant, immune, and stromal populations. Notably, Jerby-Arnon et al. (2018; PMID: 30388455) identified SDC3 as a repressed gene within their malignant-cell “immune resistance program” (PMID: 30388455; Table S4), alongside reduced antigen presentation (B2M, CTSB, HLA-A/B/C, TAPBP), IFN-γ signaling, complement response (CD59, C4A), and immune modulators (CD47, CD58).

In Pozniak et al. (Cell 2024; PMID: 38181739), our re-analysis confirmed that SDC3 is highly expressed in malignant cells, particularly within antigen-presenting and neural-crest–like melanoma subpopulations **(Rebuttal Fig. 10d)**. In this dataset, “BT” denotes pre-treatment baseline samples and “OT” denotes on-treatment biopsies collected early during immune checkpoint therapy. Within neural-crest–like melanoma cells, SDC3 expression decreased in non-responders (OT vs. BT) but increased in responders, with baseline SDC3 levels (BT) being lower in responders compared to non-responders **(Rebuttal Fig. 10e)**. Furthermore, spatial transcriptomic analysis of a pre-treatment immune-absent melanoma specimen revealed colocalization of SDC3 with STAT1 and CD46 in neural-crest–like regions, consistent with IFN-driven signaling (STAT1) and complement regulation (CD46). These observations support a role for SDC3 at the interface of innate and humoral immunity in human melanoma **(Rebuttal Figs. 10f and 10g)**.

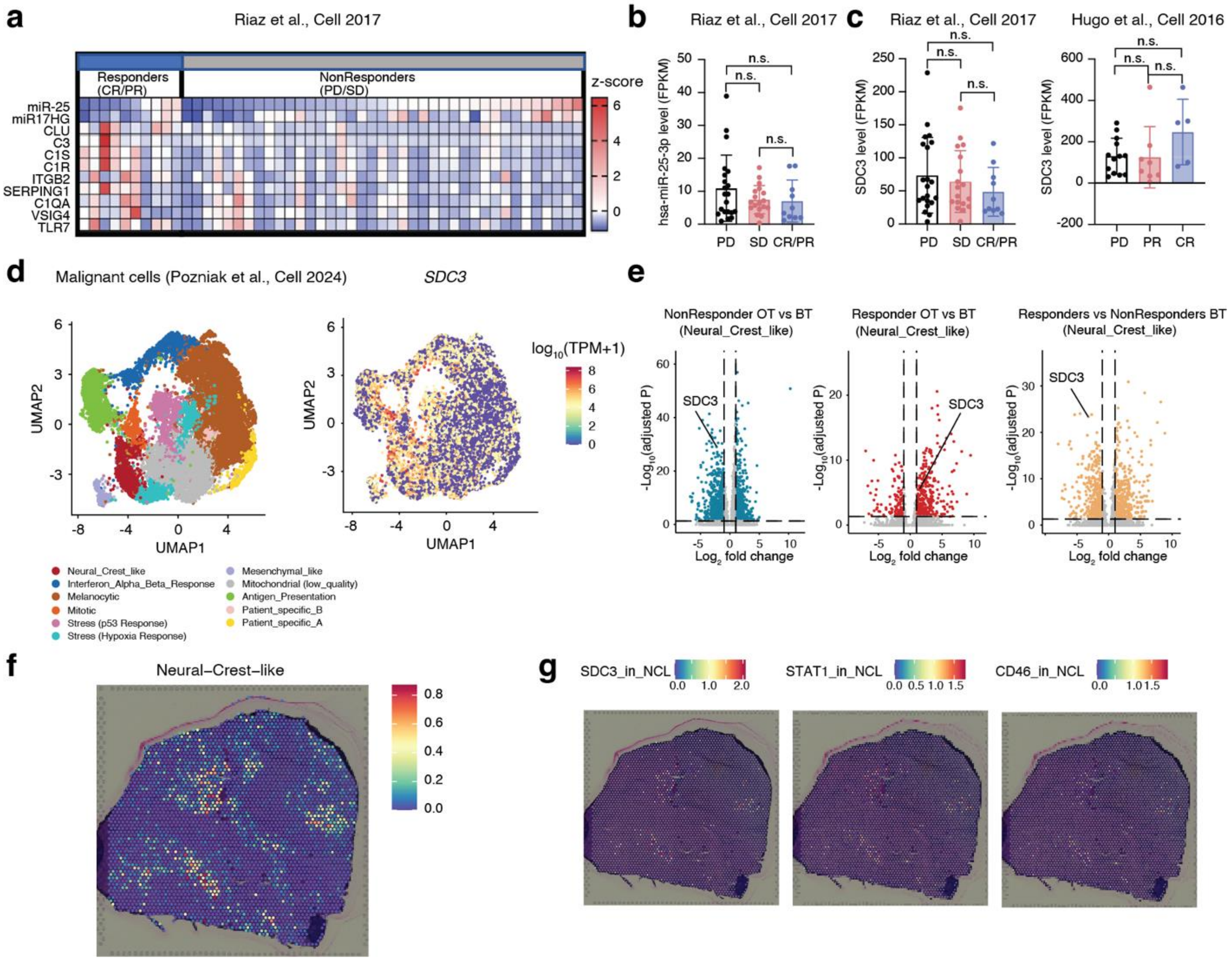

Rebuttal Figure 10. Analysis of miR-25 and SDC3 expression in human melanoma immunotherapy datasets.

(a–c) Bulk RNA-seq analysis of miR-25 and complement/innate immune signatures from Riaz et al., Cell 2017 and Hugo et al., Cell 2016.
(d) UMAP visualization of malignant cells from Pozniak et al., Cell 2024, showing SDC3 expression across malignant clusters.
(e) Volcano plots showing differential gene expression in neural-crest-like tumor cells from non-responders and responders comparing on-treatment (OT) versus baseline (BT) samples, or between responders and non-responders before treatment (BT) from the human melanoma scRNA-seq dataset. SDC3 is highlighted.
(f) Spatial transcriptomic map showing cell-type predictions in patient sample Melanoma2_1 (Visium).
(g) Spatial transcriptomic visualization of SDC3, STAT1, and CD46 expression within the neural-crest-like (NCL) region of one treatment naïve human melanoma tissue sections.
Abbreviations: CR, complete response; PR, partial response; SD, stable disease; PD, progressive disease; OT, on treatment; BT, before treatment; NCL, neural-crest-like.

(2) miR-25/SDC3 expression across tumor versus normal tissues and cancer stages

Consistent with previous reports (Philippidou et al., Cancer Res 2010; PMID: 20442294), miR-25 is broadly upregulated across human cancers and correlates with disease progression. In our TCGA-SKCM analysis, miR-25 expression displayed an upward trend from stage 0 to stage III, although the change did not reach statistical significance **(Rebuttal Fig. 11a)**. Nevertheless, this trend aligns with the previously reported association between miR-25 and tumor progression.

To further assess the relevance of the miR-25–SDC3 axis in human cancer, we examined five human cancer cell lines (A375, HCT116, HT29, MDA-MB-231, A549) and one non–cancer-derived line (HEK293T). Across all cancer cell lines, miR-25 knockout followed by IFN-γ stimulation consistently increased SDC3 expression, whereas in control (WT) cancer cells, SDC3 expression either failed to be induced (A375) or was selectively repressed (HCT116, HT29, MDA-MB-231, A549) at both the mRNA and protein levels **(Rebuttal Figs. 11b–11g)**. In contrast, HEK293T cells exhibited a slight IFN-γ–induced increase in SDC3 expression in both WT and miR-25 KO conditions, indicating that cancer cells, but not normal cells, actively suppress SDC3 under inflammatory conditions.

These results demonstrate that human cancer cells recapitulate the miR-25–SDC3 regulatory axis observed in our murine models, including IFN-γ–dependent repression of SDC3, which will potentially downregulate antigen-presentation machinery (MHC-II). Together, these findings support a conserved, tumor-intrinsic role for miR-25 in promoting immune evasion by suppressing SDC3 and dampening innate and adaptive immune activation in human cancers.

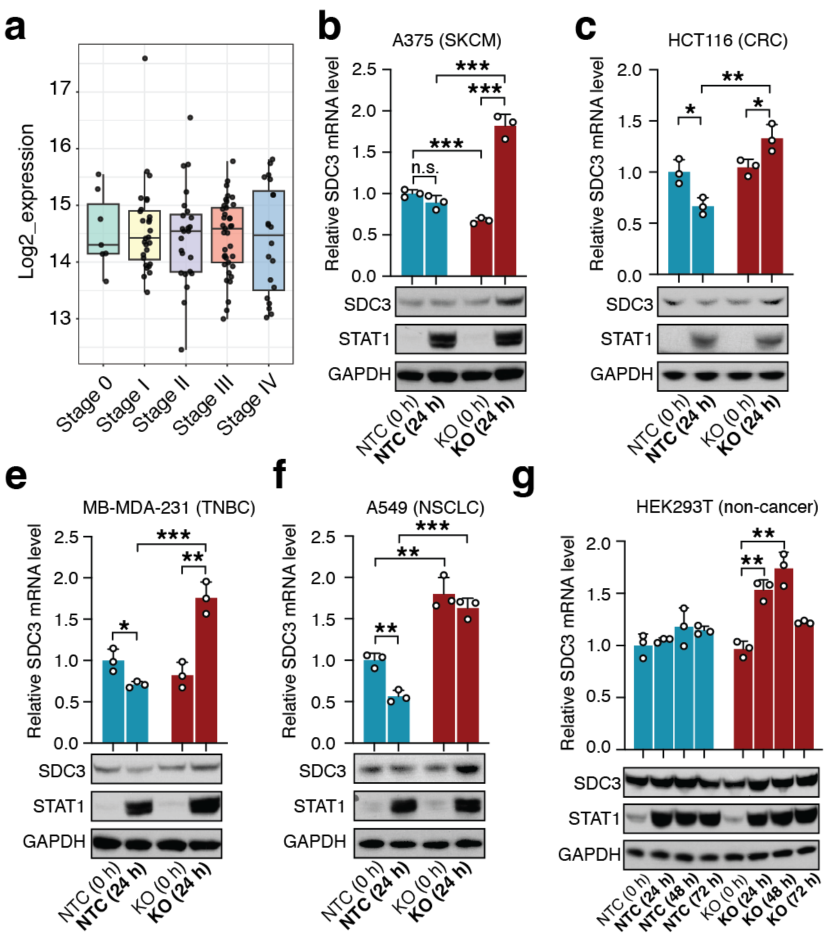

Rebuttal Figure 11. miR-25 knockout increases SDC3 expression upon IFN-γ stimulation in multiple human cancer cell lines.
(a) TCGA-SKCM analysis showing miR-25 expression across tumor stages (Stage 0–IV).
*(b–f) RT–qPCR and immunoblotting analysis of SDC3 and STAT1 expression in A375 (SKCM), HCT116 and HT29 (CRC), MB-MDA-231 (TNBC), and A549 (NSCLC) cell lines following IFN-γ stimulation. GAPDH served as a loading control. Data are presented as mean ± SD (n = 3). * $P < 0.05$, ** $P < 0.01$, *** $P < 0.001$ by Student's t-test.*
(g) RT–qPCR and immunoblotting of SDC3 and STAT1 in non-cancerous HEK293T cells treated with IFN-γ as in (b-f).

All these new data are presented in Figure 6, and the DEG list from Pozniak et al., Cell 2024 is provided as Supplementary Table 12 in the revised manuscript.

6. A stronger connection from the mechanistic mouse work to either human cell line or patient data is one of the key deficiencies of the story, but the survival differences and upregulated immune pathway activation in miR-25 low samples does partially address this. Maybe authors could check if these characteristics are also present in Sdc3 low vs high samples? And are Sdc3 levels higher in miR-25 low samples? I understand it they may not be, due to the bulk nature of the sequencing data, but it would be interesting to see.

Thank you for the comment regarding the connection between our mechanistic mouse data and human patient datasets. Mechanistic validation in human samples and cell lines has been addressed in our response to Point 5.

To evaluate whether SDC3 levels support the same trends observed for miR-25, we first examined SDC3 expression in miR-25–high versus miR-25–low TCGA-SKCM-TM samples. As anticipated given the bulk

nature of the dataset, this comparison did not reveal a clear positive correlation **(Rebuttal Fig. 12a)**. We then assessed patient outcomes stratified by SDC3 expression across multiple TCGA cancer types. Interestingly, higher SDC3 expression correlated with improved survival in several cancers—including melanoma (SKCM-TP) and uveal melanoma (UVM)—but correlated with poorer outcomes in others **(Rebuttal Figs. 12b–12e)**. In most cases, however, the differences did not reach statistical significance.

Together, these analyses suggest that while miR-25–low tumors consistently exhibit enhanced immune activation and improved survival, the interpretation of SDC3 expression from bulk RNA-seq is limited by the mixed cellular composition of tumor samples. In contrast, the tumor cell–specific SDC3 signal identified through single-cell and spatial transcriptomic analyses **(Rebuttal Fig. 10)**, together with our in vitro data from purified human cancer cell lines **(Rebuttal Fig. 11)**, provides a clearer and more biologically relevant link between SDC3, tumor-intrinsic immune regulation, and patient outcomes.

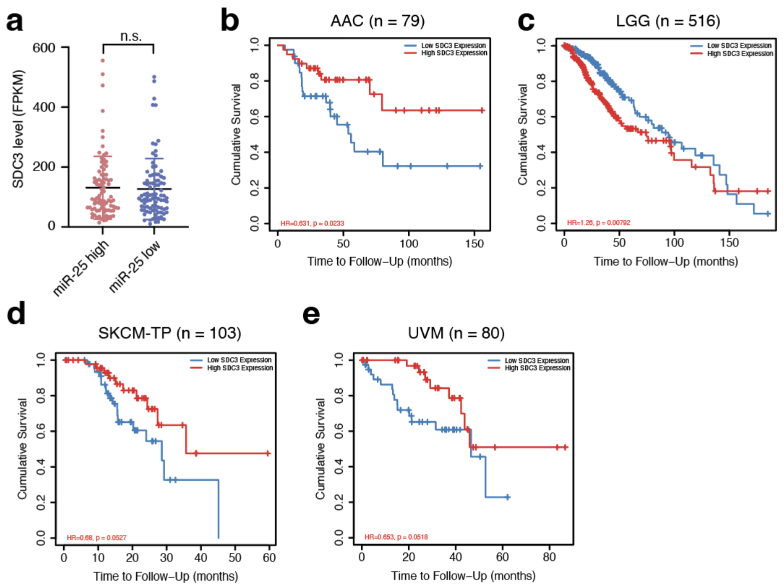

Rebuttal Figure 12. Analysis of SDC3 expression and patient outcome across TCGA cancer types.
(a) Comparison of SDC3 expression between miR-25 high and miR-25 low TCGA-SKCM-TM samples.
(b–e) Kaplan–Meier survival analyses of patients stratified by SDC3 expression (top 50% vs. bottom 50%) in ACC (adrenocortical carcinoma), LGG (lower-grade glioma), SKCM-TP (primary melanoma), and UVM (uveal melanoma) cohorts.
Abbreviations: SKCM, skin cutaneous melanoma; TM, tumor metastasis; TP, tumor primary; ACC, adrenocortical carcinoma; LGG, lower-grade glioma; UVM, uveal melanoma.

Reviewer #4 (Remarks to the Author):

The authors have appropriately addressed our previous comments.

Minor:
- Figure s5J is not described in the results section and was described as 4J in the rebuttal letter.

We thank the reviewer for carefully checking all figures, the rebuttal letter, and the manuscript. The reviewer may be referring to manuscript Figure S4J, which was not described in the Results section and was incorrectly cited as Figure 4J in the first-round rebuttal letter. We have updated Figure 4 with the revised panel order and now **describe the current manuscript Figures 4N and S4J in line 483.**

- the general references on syndecans (reviews) are over 30 years old. Admittedly, the content (citing the putative GAG attachment sites) is still valid, but the authors may want to consider replacing either reference 53 or 54 with a more recent review on syndecans, eg. an inflammation/immunity related one.

We appreciate the reviewer's careful examination of our references. In response, **we have added a recent review on syndecans in inflammation (Sandeep Gopal, Frontiers in Immunology, 2020; PMID: 32133006) as the current manuscript reference #58.**

Reviewer #5 (Remarks to the Author):

I co-reviewed this manuscript with one of the reviewers who provided the listed reports. This is part of the Nature Communications initiative to facilitate training in peer review and to provide appropriate recognition for Early Career Researchers who co-review manuscripts.

Reviewer #6 (Remarks to the Author):

I co-reviewed this manuscript with one of the reviewers who provided the listed reports. This is part of the Nature Communications initiative to facilitate training in peer review and to provide appropriate recognition for Early Career Researchers who co-review manuscripts.

Reviewer #7 (Remarks to the Author):

I co-reviewed this manuscript with one of the reviewers who provided the listed reports. This is part of the Nature Communications initiative to facilitate training in peer review and to provide appropriate recognition for Early Career Researchers who co-review manuscripts.

We thank the reviewers for their continued engagement, thoughtful evaluations, and constructive suggestions throughout the review process. Below, we provide a detailed, point-by-point response to all the remaining comments raised. Reviewers' comments are shown in black, and our responses are provided in blue.

REVIEWERS' COMMENTS

Reviewer #1 (Remarks to the Author):

I commend the authors for the extensive revision of the manuscript. The rescue experiments and the generation of miR-25 knockout clones are compelling and confirm the originally described phenotype, effectively addressing my most significant concern.

I remain puzzled by the previously reported results for miR-106, and I do not agree with the comment that RNA-seq is less accurate than RT-PCR for determining miRNA abundance.

One remaining point that should be addressed concerns the cumulative distribution function (CDF) plots shown in Rebuttal Figure 5d. As currently presented, these plots cannot be interpreted because the x-axis spans an excessively broad range (from −10 to > +5 log fold change), which necessarily compresses any biologically meaningful differences; this is particularly problematic given that the highly significant KS test strongly suggests the presence of such differences. In general, the x-axis in these plots should be restricted to a narrower range (e.g., −2 to +2 log fold change) to allow proper visualization and interpretation. This issue should be corrected prior to publication.

Thank you for the positive evaluation of the revised manuscript and for recognizing that the additional rescue experiments and independent knockout clones have addressed the major concerns.

Thank you also for your comments regarding miR-106a and the comparison between RNA-seq and RT–qPCR measurements. We agree that sequencing-based and PCR-based approaches each have their own technical considerations, and we have revised the text to clarify this point more carefully, avoiding any overstatement regarding relative accuracy.

Regarding the remaining concern about the cumulative distribution function (CDF) plots in Rebuttal Fig. 5d, we agree that the previously displayed x-axis range was excessively broad and limited interpretability. In the revised version, we have restricted the x-axis to a narrower and more appropriate range (−2 to +2 $\log_2$ fold change), which allows clearer visualization of the distribution shifts (updated figure shown below). Using the updated plots, we did not observe a global shift in the distribution of predicted miR-25 target genes compared with non-target genes. Although the Kolmogorov–Smirnov test detected statistically significant differences, the effect size was minimal, indicating only subtle transcriptome-level changes. This is consistent with functional compensation by miRNAs with shared seed sequences **(main-text Fig. 5k)**.

Thank you for this helpful suggestion, which has improved the clarity and interpretability of the data.

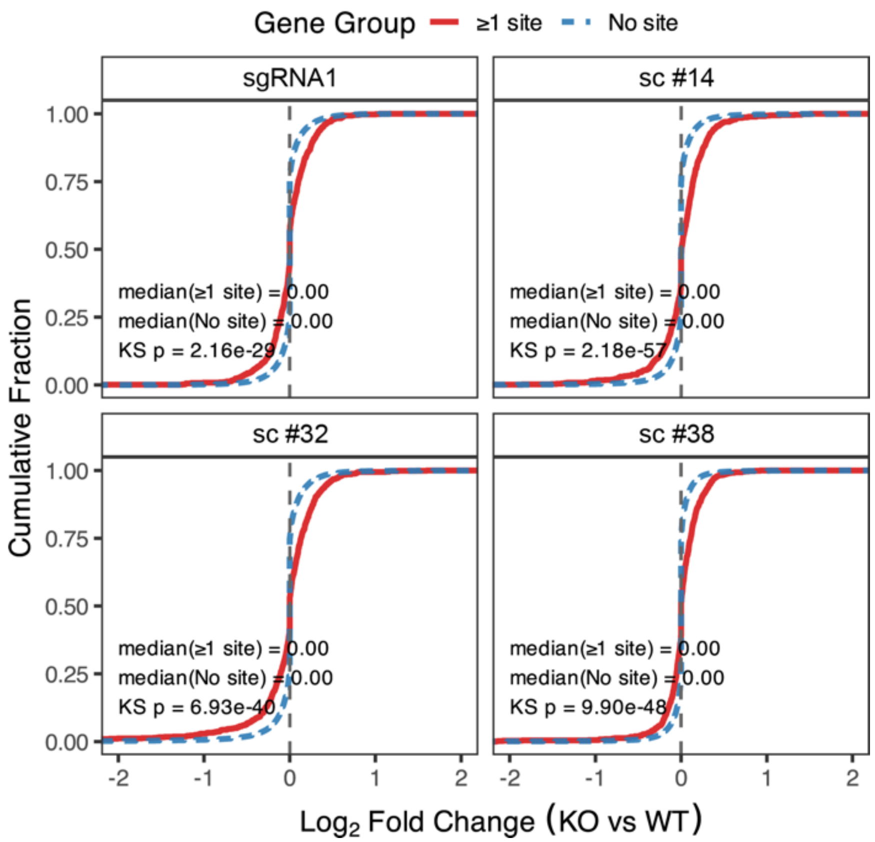

Cumulative distribution function (CDF) plots of log_2 fold changes (KO vs WT) for genes with ≥1 predicted miR-25 target site compared with genes lacking any predicted site across independent Mir25 knockout conditions. Log_2 fold changes were estimated using DESeq2 with shrinkage (ashr). Median log_2 fold changes for each group and Kolmogorov–Smirnov (KS) test P values are indicated within each panel.

Reviewer #3 (Remarks to the Author):

The revised version includes additional data analyses in response to prior concerns. However, some aspects of the model downstream of miR-25 remain unclear, particularly with regard to the crosstalk between specific macrophage populations and CAFs (e.g., which specific macrophage populations drive complement signaling in CAFs and their polarization to iCAFs). Nonetheless, given the extensive data already presented in this study regarding miR-25 modulation of Sdc3 in response to IFNγ in the context of ICB resistance, additional characterization could be the subject of future investigation.

Thank you for this thoughtful and constructive comment. We agree that the downstream mechanisms linking miR-25–SDC3 signaling to macrophage–CAF crosstalk remain incompletely understood, particularly with respect to the specific macrophage subsets involved and the intermediate signals that drive complement activation in CAFs.

To address this point, we have revised the Discussion to explicitly acknowledge this limitation and to better clarify our current working model. In the revised manuscript), we now state:

> These findings suggest that complement activation in iCAFs reflects downstream humoral immune responses driven by antigen-presenting TAMs, particularly the MHC II^+ C18 macrophage population identified in our dataset, which may provide the immune activation context enabling complement-associated inflammatory programs in CAFs. However, the molecular link between SDC3-mediated regulation of TAM antigen presentation and complement activation in CAFs remains unclear. Because

MHC II signaling is classically associated with $CD4^+$ T-cell activation (Axelrod et al., Clin Cancer Res, 2019; PMID: 30463850) rather than direct stromal regulation, the intermediate signals connecting antigen-presenting macrophages to CAF complement programs require further investigation. Consistent with this model, macrophage depletion markedly reduced C3 infiltration within tumors, indicating that complement activation depends on antigen-presenting TAMs.

Thank you for highlighting this point, which has helped us further refine the conceptual framework of our study and improve the clarity of the manuscript.

Reviewer #5 (Remarks to the Author):

I co-reviewed this manuscript with one of the reviewers who provided the listed reports. This is part of the Nature Communications initiative to facilitate training in peer review and to provide appropriate recognition for Early Career Researchers who co-review manuscripts.
